# Gene Expression Profiles Associated with Brain Aging are Altered in Schizophrenia

**DOI:** 10.1038/s41598-019-42308-5

**Published:** 2019-04-11

**Authors:** Sarven Sabunciyan

**Affiliations:** Department of Pediatrics, Johns Hopkins University, Baltimore, MD 21287 USA

## Abstract

Existence of aging associated transcriptional differences in the schizophrenia brain was investigated in RNA sequencing data from 610 postmortem Dorso-Lateral Pre-Frontal Cortex (DLPFC) samples in the CommondMind Consortium (CMC) and the psychENCODE cohorts. This analysis discovered that the trajectory of gene expression changes that occur during brain aging differed between schizophrenia cases and unaffected controls. Mainly, the identified gene expression differences between the diagnosis groups shrank in magnitude following 60 years of age. A differential expression analysis restricted to the 40 to 60 year age group identified 556 statistically significant loci that replicated and had highly consistent gene expression fold changes in the two cohorts. An interaction between age and diagnosis in the wider psychENCODE cohort was also detected. Gene set enrichment analysis discovered disruptions in mitochondria, RNA splicing and phosphoprotein gene pathways. The identified differentially expressed genes in the two cohorts were also significantly enriched in genomic regions associated with schizophrenia although no enrichment was observed for differentially expressed genes identified in the 40 to 60 year age group. This work implicates disruptions to the normal brain aging processes in the pathology of schizophrenia and demonstrates the need for age stratification in schizophrenia postmortem brain gene expression studies.

## Introduction

Gene expression in the postmortem schizophrenia brain has been extensively characterized by microarray and next generation sequencing studies^1–6^. The consensus from these studies is that there are many subtle expression differences in individual genes that likely alter the functioning of various gene pathways in schizophrenia^2,4,7,8^. The standard analysis approach employed by these studies assumes that gene expression and age have a linear relationship. Thus, appropriate statistical methods are used to adjust for the effect of age on gene expression. However, epidemiological studies consistently find excess early mortality^8,9^ in disease. In addition, studies that estimate brain age based on neuroanatomical structures^10^ and integrity of the white matter^11,12^ have found accelerated aging in schizophrenia. As transcriptional changes are likely to accompany neuroanatomical changes in the brain, the relationship between age and disease is likely to be more complex than assumed in current analysis pipelines. Therefore, in this work publicly available RNA sequencing data was reanalyzed to determine whether the transcriptome of the aging brain differs between unaffected controls and schizophrenia cases.

## Results

### Gene expression profiles in the aging schizophrenia brain is altered

**Figure 1:**
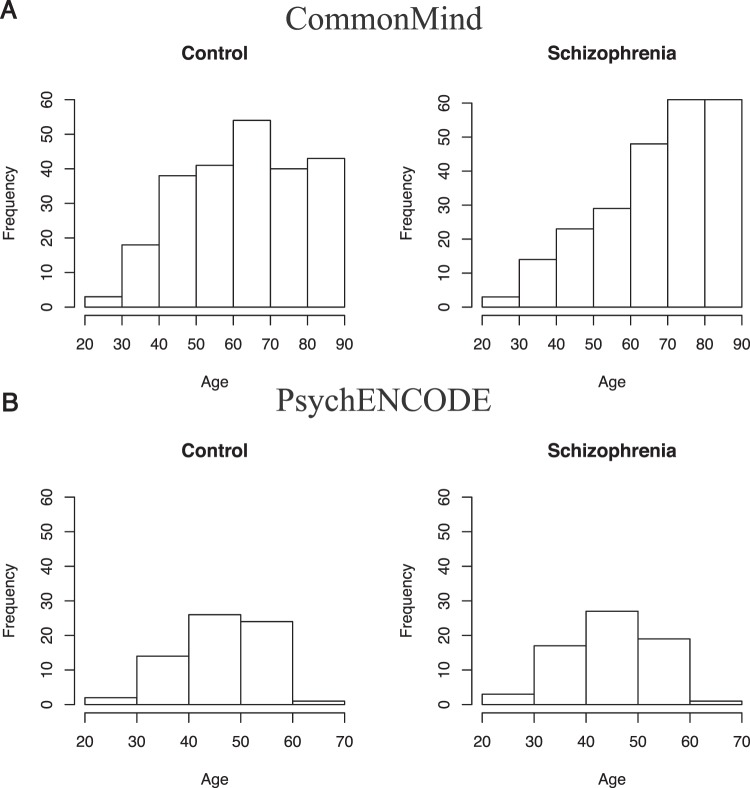
Demographic Data. Histogram of diagnoses by age for (**A**) the CMC and (**B**) the psychENCODE Cohorts. The psychENCODE cohort has fewer samples and a narrower age range.

In order to study aging in schizophrenia, RNA sequencing data generated from the BA9 of the CMC and BA46 of the psychENCODE postmortem brain collections were reanalyzed. As CMC has the largest number of brain samples with RNA sequencing data and gene expression differences in schizophrenia have already been identified in this cohort^1,13^, we reasoned that this cohort likely has the necessary statistical power to detect differences in our analysis. Following the exclusion criteria (see Methods) 237 unaffected controls and 239 schizophrenia subjects between the ages of 25 to 90 remained (Fig. 1A). In the psychENCODE cohort only BA46 samples from schizophrenia and control cases that were between the ages of 25 and 65 were included (See Methods). This left a total of 67 cases and 67 control samples (Fig. 1B).

Gene count tables were generated for the CMC cohort using the Ensembl annotation for the hg19 human genome build and the DESeq2^14^ package from the bioconductor project was used to identify differentially expressed loci. The following statistical design that assumes an interaction between diagnoses and age was used (see Methods for details):

$$ \sim {\rm{Diagnosis}}+{\rm{Age}}+{\rm{Age}}:{\rm{Diagnosis}}+{\rm{Gender}}+{\rm{Ethnicity}}+{\rm{PMI}}+{\rm{RIN}}+{\rm{RuvFactors}}$$

**Figure 2:**
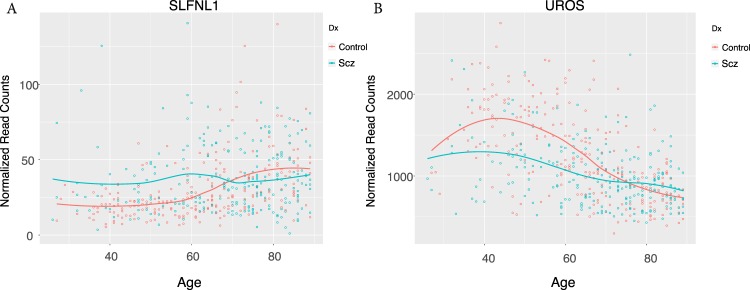
Gene Expression Differences Between Schizophrenia and Control Brains During Aging Representative plots of the most common gene expression trajectories found in the CMC analysis. Observed normalized read counts were plotted vs age and the loess function was used to fit the best line through the data points.

**Figure 3:**
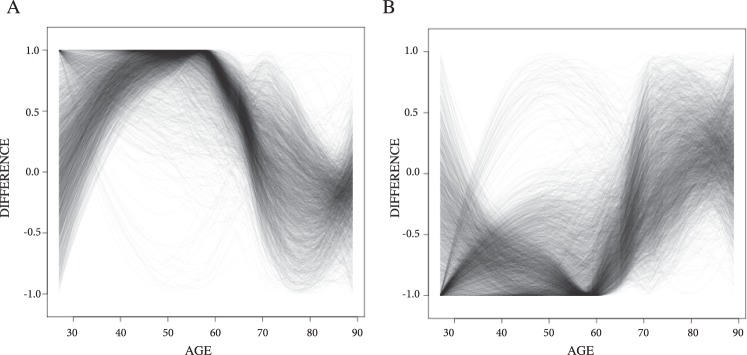
Gene Expression Differences Between Controls and Schizophrenia At Each Age The normalized difference in gene expression between control and schizophrenia samples is plotted for ages 25 to 90 for genes identified to be differentially expressed in the CMC by Wald tests using DESeq2. As the values at leach locus are normalized to the observed maximum difference, only values between 1 and −1 are possible. In order to avoid over plotting, the loci were grouped into those with (**A**) positive maximum difference (expression higher in controls) occurring at age 60 or less (3114 loci), (**B**) negative maximum difference (expression higher in schizophrenia) occurring at age 60 or less (3369 loci).

PMI refers to Post Mortem Interval whereas RIN is the RNA Integrity Number, which is an estimate of RNA quality^15,16^. RuvFactors were calculated using the RuvSeq package which is a statistical method to remove unwanted variation from RNA sequencing data^17^. Based on these criteria, Wald tests identified 7434 differentially expressed genes in which the adjusted *p*-value (corrected for multiple testing) was <0.1 and each gene on average had at least 20 reads per sample. Similar to previous studies, we found moderate differences in fold change in the statistically significant differences we identified (Fig. 2 and Supplementary Fig. 1 plots the top 100 loci with the lowest *p*-values). These graphs revealed that the difference in gene expression levels between schizophrenia cases and unaffected controls vary with age (Fig. 2). The maximum gene expression difference in the CMC was consistently between 40 and 60 years of age (Supplementary Figure 1) with the caveat that there are relatively few samples under the age of 40 in the CMC cohort. For many loci the expression differences at ages older than 60 gets smaller and the direction of the change is reversed. In order to summarize the results of the differential expression analysis, the normalized difference between schizophrenia cases and unaffected controls was plotted for each locus (Fig. 3 and Supplementary Fig. 2. See Normalized Difference Plots in Methods). Briefly, schizophrenia expression levels (the blue lines in Fig. 2) were subtracted from control expression levels (the red lines in Fig. 2) for each age. The resulting differences in expression at each age were plotted for every locus (Fig. 3 - See methods for details). In order to avoid over plotting, the differentially expressed loci were subdivided into those with a maximum difference at ages 60 or less and were 1) more expressed (3114 loci) or 2) less expressed (3369 loci) in controls (Fig. 3) and those with a maximum difference at ages over 60 (Supplementary Fig. 2) that were 3) more expressed (405 loci) or 4) less expressed (546 loci) in controls. These plots verified that the difference in gene expression between cases and controls is not constant across age. Although, the CMC cohort contains 307 samples over the age of 60 there are only 33 samples under the age of 40. Therefore, the smaller gene expression differences observed between cases and controls under the age of 40 needs to be interpreted with caution.

### Age stratification improves consistency between studies

As the largest expression differences between schizophrenia and control samples occurred consistently between 40 and 60 years of age in the CMC analysis, differential expression analysis for this age group in the CMC and psychENCODE cohorts was performed. Eighty-two cases and 54 controls were present in the CMC and 51 cases and 48 controls were present in the psychENCODE cohorts. Gene count tables were generated based on the Ensembl hg19 annotation for both cohorts. For this analysis the interaction term was removed and the following regression formula was used:

$$ \sim {\rm{Diagnosis}}+{\rm{Age}}+{\rm{RIN}}+{\rm{PMI}}+{\rm{Race}}+{\rm{Sex}}+{\rm{RuvFactors}}$$

**Figure 4:**
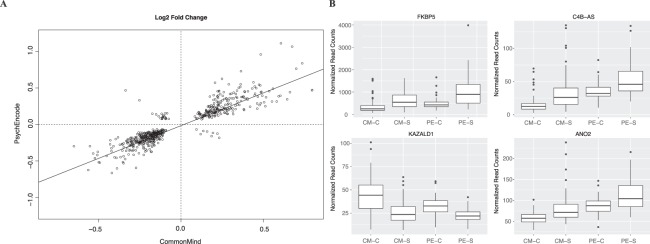
Gene Expression Differences For the 40 to 60 Age Group. (**A**) The fold change differences detected in the psychENCODE plotted against the fold change differences detected in the CMC for the 556 loci identified to be significantly differentially expressed in both cohorts at the 40 to 60 year old age group. (**B**) Boxplots of differentially expressed loci selected based on significance level and/or relevance to disease. The x-axis label CM-C is CommonMind Control, CM-S is CommonMind Schizophrenia, PE-C is psychENCODE Control, PE-S is psychENCODE Schizophrenia.

The resulting analysis identified 2167 differentially expressed genes in the CMC and 4086 in psychENCODE that had an adjusted *p*-value of less than 0.1 and at least 20 reads per gene. After excluding results driven by outliers (using cooks distance) 556 loci replicated between the cohorts (Fig. 4, Supplementary Table 1). The *p*-values ranged from 1.25e-05 to 0.1 (mean 0.045) for the CMC cohort and 2.71e-09 to 0.1 (mean 0.027) for the psychENCODE cohort whereas the absolute fold change ranged from 1.05–1.86 (mean 1.18) for the CMC and 1.04–2.16 (mean 1.17) for psychENCODE. In order to determine the consistency of the changes between the two cohorts, the fold change difference in the two studies was plotted (Fig. 4A). The fold change difference between the two studies were highly correlated (r^2^ = 0.82) Boxplots were used to gage the variability between diagnosis groups (Fig. 4B). Gene set enrichment analysis performed using the DAVID annotation tool^18^ revealed that these 556 genes are mostly associated with mitochondrial functions (fold enrichment 1.88–6.48, *p-value* 0.0017–0.1), a biological pathway implicated to be involved in the pathology of schizophrenia (Table 1).

**Table 1:** Gene Set Enrichment Analysis For Differentially Expressed Loci In The 40 To 60 Year Age Group.

Category	Term	Gene Count	Fold Enrichment	Bonferroni
SP_PIR_KEYWORDS	mitochondrion	43	2.16	1.74E-03
SP_PIR_KEYWORDS	transit peptide	30	2.64	1.78E-03
SP_PIR_KEYWORDS	respiratory chain	11	6.48	2.84E-03
UP_SEQ_FEATURE	transit peptide:Mitochondrion	30	2.67	4.61E-03
GOTERM_CC_FAT	GO:0005739~mitochondrion	51	1.88	5.30E-03
GOTERM_CC_FAT	GO:0070469~respiratory chain	11	5.88	5.54E-03
GOTERM_CC_FAT	GO:0044429~mitochondrial part	33	2.22	1.20E-02
GOTERM_BP_FAT	GO:0030198~extracellular matrix organization	13	5	1.95E-02
SP_PIR_KEYWORDS	mitochondrion inner membrane	16	3.47	2.64E-02
KEGG_PATHWAY	hsa05010:Alzheimer’s disease	14	3.31	3.17E-02
KEGG_PATHWAY	hsa05012:Parkinson’s disease	12	3.61	5.05E-02
KEGG_PATHWAY	hsa00190:Oxidative phosphorylation	12	3.56	5.75E-02
GOTERM_CC_FAT	GO:0005746~mitochondrial respiratory chain	9	5.63	6.11E-02
GOTERM_CC_FAT	GO:0019866~organelle inner membrane	21	2.56	7.50E-02
GOTERM_CC_FAT	GO:0044455~mitochondrial membrane part	12	3.85	9.58E-02

### Gene set enrichment analysis implicates similar gene pathways in the CMC and The psychENCODE cohorts

Although the psychENCODE cohort was much smaller and only spanned an age range of 25 to 65, the existence of an interaction between age and diagnosis was investigated in this cohort. Thus the analysis performed on the CMC cohort using the formula ~Diagnosis + Age + Age: Diagnosis + Gender + Ethnicity + PMI + RIN + RuvFactors was repeated. The threshold for statistical significance was again set at an adjusted *p*-value of <0.1 and a minimum average read count of 20/sample. Based on these criteria we identified 29 loci to be differentially expressed (Supplementary Table 2). Fifteen out of these loci were also differentially expressed in the CMC cohort (11 in the same direction). Gene set enrichment analysis on the 29 identified loci did not yield statistically significant results. However, a plot of the *p*-values for all genes tested in the psychENCODE cohort revealed that there are many nominally significant genes (unadjusted *p*-value <0.05) that failed to reach the threshold of statistical significance following multiple testing (Supplementary Fig. 3) suggesting that this small cohort might be underpowered. Gene set enrichment analysis was performed using the DAVID annotation tool^18^ on the 2976 nominally expressed genes in psychENCODE (unadjusted *p* < 0.05, minimum mean read count 20 and outlier removal by cooks distance) and the significantly expressed loci in the CMC to determine whether there was any overlap between the two cohorts. Gene pathways associated with acetylation (*p* = 4.75E-57) and phosphoproteins (*p* = 3.55E-40) had the most significant enrichment in the CMC analysis. In addition multiple gene pathways associated with mitochondrial functions, splicing and ribosome activity were significantly enriched (Table 2, Supplementary Table 3). The 4 gene pathways that were significantly enriched in the psychENCODE cohort were alternative splicing (*p* = 1.25E-10), splice variant (*p* = 5.28-09) phosphoprotein (*p* = 1.66E-07) and coiled coil pathways (*p* = 0.09) (Table 2).

**Table 2:** Gene Set Enrichment Analysis Results*.

Category	Term	Gene Count	Fold Enrichment	Bonferroni
**CommonMind**				
SP_PIR_KEYWORDS	acetylation	1070	1.50	4.75E-57
SP_PIR_KEYWORDS	phosphoprotein	2375	1.21	3.55E-40
GOTERM_CC_FAT	GO:0005739~mitochondrion	458	1.58	6.12E-28
GOTERM_CC_FAT	GO:0030529~ribonucleoprotein complex	250	1.82	3.61E-24
SP_PIR_KEYWORDS	mitochondrion	359	1.60	9.83E-22
SP_PIR_KEYWORDS	alternative splicing	2325	1.15	4.17E-21
GOTERM_CC_FAT	GO:0070013~intracellular organelle lumen	647	1.36	1.38E-19
GOTERM_CC_FAT	GO:0044429~mitochondrial part	266	1.67	3.02E-19
GOTERM_CC_FAT	GO:0005840~ribosome	125	2.18	3.11E-19
SP_PIR_KEYWORDS	ribonucleoprotein	152	2.01	3.53E-19
**PsychENCODE**				
SP_PIR_KEYWORDS	alternative splicing	1006	1.19	1.25E-10
UP_SEQ_FEATURE	splice variant	1002	1.18	5.28E-09
SP_PIR_KEYWORDS	phosphoprotein	957	1.16	1.66E-07
SP_PIR_KEYWORDS	coiled coil	280	1.22	9.15E-02

*Only the 10 most significant results listed for CommonMind.

### Differentially Expressed Genes Identified In The CMC And The psychENCODE Cohorts Are Enriched In The 108 Regions Associated With Schizophrenia

In order to determine whether the differentially expressed loci identified in the CMC and the psychENCODE cohorts are enriched near the genomic regions associated with schizophrenia^19^, Inrich analysis^20^ was performed. The 556 loci identified in the 40 to 60 year age group were not enriched in genomic regions associated with schizophrenia. However, out of the 95 schizophrenia regions that contain genes (based on the Ensembl gene annotations), the differentially expressed genes identified in the CMC at a threshold of adjusted *p*-value <0.1 overlapped 54 genomic regions associated with schizophrenia (Inrich analysis adjusted *p* = 0.01, Supplementary Table 4). Repeating the Inrich analysis for differentially expressed genes that had an adjusted p-value of <0.05 (overlapped 50 GWAS regions, Inrich adjusted *p* = 0.0024) and < 0.01 (overlapped 35 GWAS regions, Inrich adjusted *p* = 0.0016) improved the significance of the enrichment. The nominally significant psychENCODE loci overlapped 33 genomic regions associated with schizophrenia (Inrich *p* = 0.02) (Supplementary Table 5). Twenty-six out of the thirty-three regions (76% overlap) identified in the psychENCODE cohort were also statistically significant in the Inrich CMC analysis (Supplementary Tables 4 and 5).

## Discussion

The process of brain aging differs significantly between schizophrenia cases and unaffected controls. The level of differential expression between diagnoses groups in the brain varies with age highlighting the complex affect aging has on disease gene expression profiles. As the largest gene expression level differences in disease for the CMC were in the 40 to 60 year age range, we compared this age group between the CMC and psychENCODE. Five hundred fifty six loci enriched in mitochondrial functions were identified that surpassed the threshold for multiple testing in both the CMC and psychENCODE cohorts. The consistency of fold change differences (r^2^ = 0.82) between the two cohorts provides further confidence in this finding. In comparison, the analysis of the entire CMC cohort yields much smaller gene expression difference compared to other cohorts - a mean of 1.09 and a range of 1.03–1.33 fold^1^. The similar differences observed between the CMC and psychENCODE when the analysis is restricted to the 40 to 60 year age group strongly suggests that age stratification in schizophrenia gene expression studies is beneficial. Although the largest gene expression differences observed in the CMC data set was between the ages of 40 and 60, the scarcity of samples under 40 years of age prevented us from characterizing gene expression changes in younger subjects. Clearly, ages from late adolescence to early adulthood are extremely important to schizophrenia pathophysiology as disease onset occurs at this stage. However, since most psychiatric brain banks lack samples at these young ages, we are unable to perform a thorough analysis of a key stage in schizophrenia and are likely missing important gene expression changes associated with disease pathology. Evidence for a statistical interaction between age and schizophrenia was discovered in both the CMC and the psychENCODE cohorts. Gene set enrichment analysis revealed disruptions in splicing and phosphoprotein pathways to be common to both cohorts. In addition, the differentially expressed genes identified were enriched in the genomic regions associated with schizophrenia. This finding was especially strong in the CMC cohort and encompassed more than half of all genic regions associated with disease (54 out of 95).

Modeling an interaction between age and diagnosis did not eliminate discrepant findings between the CMC and psychENCODE cohorts. One reason for this discrepancy may be related to the differences between the cohorts. The CMC project generated sequencing data on BA9 samples whereas the PsychENCODE consortium sequenced BA46 samples. The psychENCODE cohort was also less than 3.5 times that of the CMC and may have been underpowered. The overlap in gene set enrichment and Inrich analysis between the CMC and the nominally significant genes in the psychENCODE cohorts support this notion. The fact that the psychENCODE cohort spanned ages between 25 and 65 as opposed to the CMC that spanned ages between 25 and 90 may have also contributed to the differences in these findings. Another reason for the discrepant findings is likely related to the heterogeneous nature of schizophrenia. As schizophrenia genetic studies required over a hundred thousand samples in order to overcome the heterogeneity problem^19^, expecting every loci to be differentially expressed in every single schizophrenia postmortem brain sample is unrealistic. It is likely that the disruption of different genes that belong to the same biological pathway results in similar pathology in different people. This is congruent with the presented gene set enrichment analysis that found disruptions in RNA splicing and phosphoprotein pathways in both the CMC and the psychENCODE cohorts.

This work implicates gene pathways associated with mitochondrial function, splicing and phosphoproteins in schizophrenia pathology. Previous schizophrenia postmortem brain studies have reported differential gene expression for mitochondrial genes in disease^21,22^. Widespread splicing deficits have also been reported in schizophrenia^2,23,24^ and numerous phosphoproteins such as synapsin II^25^ and DARPP-32^26^ have been implicated in disease pathology. It is important to note that mitochondrial changes^27,28^, widespread alterations in splicing patterns^29^ and altered phosphorylation^30,31^ also occur in the aging brain. Potentially, the deficits observed in schizophrenia might be similar to the changes that occur in the aging brain.

This notion is in line with previous studies that have found a link between aging and schizophrenia. Many studies have found schizophrenia patients to have lower life expectancy (for a review see^8^) and the suggestion has been made that schizophrenia might be a systematic disease of accelerated aging^32^. Although this work discovered age related changes in schizophrenia, our results do not necessarily support accelerated aging in the disease. It is important to note that our current understanding of brain aging at the molecular level is limited and therefore, we may be failing to recognize patterns associated with accelerated aging in our data. These results are also consistent with previous pathway analysis performed on microarray data that found transcriptional differences in the aging schizophrenia brain^33,34^. Given that inflammation is associated with old age, the findings of increased inflammation in schizophrenia^35–37^ might also be related to the aging disruptions we are finding. In summary, our results along with previous findings strongly support the existence of a link between aging and schizophrenia pathology. Additional postmortem brain collections that examine schizophrenia throughout the entire lifespan and are cognizant of the importance of age are needed to fully explore the effects of age on gene expression in the disease.

Individuals suffering from schizophrenia have a tendency to smoke^38^, use recreational drugs, and consume excess levels of alcohol compared to the general population^39^. In addition, schizophrenia patients use various antipsychotic and other psychiatric medications that cause weight gain and other health problems^40,41^. These factors have the potential to disrupt the aging process in the brain and may account for our findings. Conceivably, the expression differences observed in the 40 to 60 age group are caused by such factors. However, the statistically significant enrichment between age dependent RNA expression changes in the brain and the 108 genomic regions associated with schizophrenia is intriguing. Further research is needed to determine whether some of the age related gene expression differences observed in the schizophrenia brain might be inherited.

Duration of illness, along with lifetime antipsychotic use are likely to affect gene expression since they are associated with brain volume changes in schizophrenia^42,43^. Duration of illness in schizophrenia is of particular interest as it appears to influence treatment response^44,45^. The effects of these factors on gene expression could not be assessed in the current study because this information was not available for the CommonMind collection. In the smaller psychENCODE cohort, which spans a narrower age range and appears to be statistically underpowered, both duration of illness and lifetime antipsychotic use were highly correlated with age. Generally, older subjects with schizophrenia had lived with the disease longer and had used more antipsychotics. Given this association with age, an adequate assessment of the effects of disease duration or the effects of lifetime antipsychotic use will require comparison of early and late disease onset cases that live into old age and comparison of subjects at similar ages that differ in their lifetime antipsychotic use. Each of these comparisons will likely require large cohorts, potentially similar in size to CommonMind, because of the subtle gene expression differences that occur in schizophrenia. It is probable that at least a subset of the differentially expressed genes identified in the current study are associated with the pathology related to duration of illness and/or lifetime antipsychotic use but definitive conclusions can not be made. Larger postmortem brain collections will also enable characterization of the associations between gene expression and disease subtypes or assessment scores, neither of which were available for this study. Potentially, an analysis cognizant of disease subtypes or assessment scores will be extremely beneficial as it may refine the heterogenous schizophrenia classification and yield larger and more consistent gene expression differences.

We conclude that age is an important aspect of schizophrenia pathophysiology and special consideration needs to be given to age in gene expression studies of disease. Appropriate treatment of age as a variable is likely to aid in efforts to unravel the molecular etiology of schizophrenia and the identification of biomarkers and therapeutic targets for the disease.

## Materials and Methods

### Samples

Differential expression analysis was performed on the DLPFC RNA sequencing data from CMC^1^ (N = 476) and the PsychENCODE^46^ (N = 134) projects. Details regarding the ethical approval process and relevant adherence to relevant guidelines and regulations are detailed in the original publications^1,46^. Access to the RNA sequencing data was approved by the NIMH Respository and Genomic Resource Data Access Committee and deidentified data was downloaded. In the CMC cohort only BA9 samples from schizophrenia cases and unaffected controls were included. Samples from individuals with Klinefelter syndrome were excluded and only a single individual chosen at random from a sibling pair was included in the analysis. Samples below the age of 25 were excluded since there were only 3 schizophrenia samples in this age group. The age group labeled 90+ was also excluded since an exact age could not be assigned for the regression analysis. Samples less then 25 and more then 65 years of age in the psychENCODE cohort were excluded in order to have a balanced number of cases and controls across all age groups (the 3 samples above 65 were controls and 6 out of the 7 samples below 25 were schizophrenia cases). psychENCODE samples with a PMI of greater than 50 hours were also excluded in order to be consistent with the CMC cohort.

### Differential expression analysis of schizophrenia and aging

All analysis was performed using R version 3.4.3. Bam files for each corresponding study was downloaded and count tables were generated for genes (considering only exons) using the Ensembl hg19 annotation. Specifically, the readGAlignmentPairs command from the GenomicAlignments package^47^ was used to read the alignments from the bam files and the resulting data structure along with the Ensembl hg19 annotation was passed as parameters to the summarizeOverlaps command. The summarizeOvelaps command calculates the number of reads that span the exon intervals in the annotation and provide the total number of reads that are present for each gene. RUVSeq package was used to account for hidden batch effects and remove unwanted variation from the samples^17^. In order to identify loci that are not differentially expressed between the diagnosis groups, which is required by RUVSeq, we performed an initial analysis using DESeq2^14^ in which we only considered diagnosis and age. Genes that had an unadjusted p-value of >0.7 were considered to be not differentially expressed and passed to the Ruvg command to calculate normalization factors that account for unwanted variation^17^. The factors calculated from RUVseq were included in the design formula of the DESeq analysis. Wald tests were performed in DESeq2 using the DESeq command, which also performs normalization on the samples, in order to identify differentially expressed genes. The statistical model used for the Wald test included an interaction term between diagnosis and age. This and all subsequent formulas were used as the ‘design’ parameter in DESeq2.

$$(\,\, \sim \,\,{\rm{Diagnosis}}+{\rm{Age}}+{\rm{Diagnosis}}:{\rm{Age}}+{\rm{RIN}}+{\rm{PMI}}+{\rm{Race}}+{\rm{Sex}}+{\rm{RuvFactors}})$$

The resulting *p*-values were corrected (or adjusted) for multiple testing using the Benjamini & Hochberg method implemented in the DESeq2 package. Only loci with a corrected *p*-value <0.1 on average 20 read counts per gene were considered significantly differentially expressed. An adjusted *p*-value of 0.1 is recommend over 0.05 since the multiple correction methods used to adjust the *p*-values are overly stringent^14^. We repeated the same DESeq2 analysis without RuvFactors and found that the age related trajectory differences are observed even in the absence of batch effect correction (data not shown). Cooks distance was calculated for each differentially expressed locus that surpassed the threshold of statistical significance in order to remove genes that appear to be different because of outliers. The Cooks distance cutoff threshold was identified empirically for each cohort. A similar analysis was performed for the 40 to 60 year old age group using the same formula as above but lacking the interaction term.

$$(\,\, \sim \,\,{\rm{Diagnosis}}+{\rm{Age}}+{\rm{RIN}}+{\rm{PMI}}+{\rm{Race}}+{\rm{Sex}}+{\rm{RuvFactors}})$$

New normalization factors were calculated using RuvSeq for the 40 to 60 year age group (an initial DESeq2 analysis was performed with diagnosis only and non-differentially expressed genes were identified as before).

### Differential expression plots

The raw normalized counts from the DESeq data object were extracted using the ‘counts’ function with the normalized parameter set to true. The obtained normalized read counts were plotted over age using the bioconductor ggplot2 package. We opted to not use log-transformed values in order to provide the most accurate representation of the data.

### Normalized difference plots

For each differentially expressed locus identified in the CMC analysis, the loess function in R was used to fit gene expression in control or schizophrenia samples and age. The R ‘predict’ function was run to calculate gene expression values for the age range 25–90 based on the results of the loess function. For each locus, the resulting values for the schizophrenia samples were subtracted from control samples and this value was normalized to the absolute maximum difference in all ages using the following formula:

$$({\rm{control}}\,-\,{\rm{schizophrenia}})/{\rm{\max }}({\rm{abs}}({\rm{control}}-{\rm{schizophrenia}}))$$

Normalization meant that only values between −1 and 1 were possible. Each locus had a value of 1 or −1 at some age indicating the maximal gene expression difference in disease for the locus. The normalized values for each locus was plotted using a very thin line in order to avoid over plotting.

### GWAS enrichment and gene set analysis

Differentially expressed genes in the CMC with an adjusted *p*-value of 0.1 or less and a minimum mean read count of 20 were used for gene set enrichment and Inrich^20^ analysis. The DAVID 6.7 Bioinformatics Resource (https://david-d.ncifcrf.gov/) was selected for the gene set enrichment analysis as it is a robust and widely used tool with over 33000 citations^48^. DAVID performs a competitive analysis using a modified Fisher’s exact test. The standard DAVID analysis^49^ was performed using the default conditions of minimum 2 genes per term and an EASE score, which is a modified and more stringent Fisher Exact P-value, of 0.1. The genes in the human genome were used as the background gene list. The databases included in the DAVID analysis were (Selected from Annotation Summary Results page that is generated by DAVID after gene sets are uploaded and mapped):

OMIM (https://www.ncbi.nlm.nih.gov/omim) from the Disease category; COG_ONTOLOGY (https://www.ncbi.nlm.nih.gov/COG/), SP_PIR_KEYWORD (https://proteininformationresource.org/pirwww/dbinfo/iproclass.shtml) and UP_SEQ_FEATURES (https://www.uniprot.org/) from the Functional Categories category; GOTERM_BP_FAT, GOTERM_CC_FAT and GOTERM_MF_FAT (https://www.ebi.ac.uk/GOA) from the Gene_Ontology category; BBID^50^, BIOCARTA (https://cgap.nci.nih.gov/Pathways/BioCarta_Pathways) and KEGG_PATHWAY (https://www.genome.jp/kegg/) from the Pathways category; INTERPRO (https://www.ebi.ac.uk/interpro/), PIR_SUPERFAMILY (https://proteininformationresource.org/pirwww/dbinfo/iproclass.shtml) and SMART (http://smart.embl-heidelberg.de/) from the Protein_Domains category.

Inrich, analysis was performed as described^51^ to determine whether intervals near schizophrenia associated loci were more likely to be associated with the identified differentially expressed genes. Only genes that fell within the GWAS regions were considered. Adjusted *p*-value <0.05 from the Inrich program was set as the threshold for significance. For the PsychENCODE cohort, genes with a nominal *p*-value of 0.05 or less and a minimum read count of 20 were used for the same analyses. The same genes were used for gene set enrichment analysis performed using the Database for Annotation, Visualization and Integrated Discovery (DAVID) 6.7 web tool^18^. In the 40 to 60 age group the 556 genes that replicated between the two cohorts were subjected to DAVID and Inrich analyses. Gene pathways reaching a Bonferroni corrected *p*-values < 0.1 from the DAVID analysis are reported.

## Supplementary information

Supplementary Materials

## Data Availability

The CMC and the psychEncode datasets are available from the NIMH Repository and Genomics Resources (NRGR).

## Code Availability

Analysis code used for this manuscript is available upon request and without restriction.
