# Supplementary material for: Gene Expression Profiles Associated with Brain Aging are Altered in Schizophrenia
Source: Sci Rep. 2019 Apr 11;9:5896. doi: 10.1038/s41598-019-42308-5 (PMC6459977; doi:10.1038/s41598-019-42308-5)
Supplement: Supplementary file 1 — Supplementary Materials [file 41598_2019_42308_MOESM1_ESM.pdf]

Supplementary Materials

Gene Expression Profiles Associated with Brain Aging are Altered in Schizophrenia

Sarven Sabunciyan*, Ph.D.

Department of Pediatrics, Johns Hopkins University, Baltimore, MD 21287

Supplementary Figure 1
(100 plots)

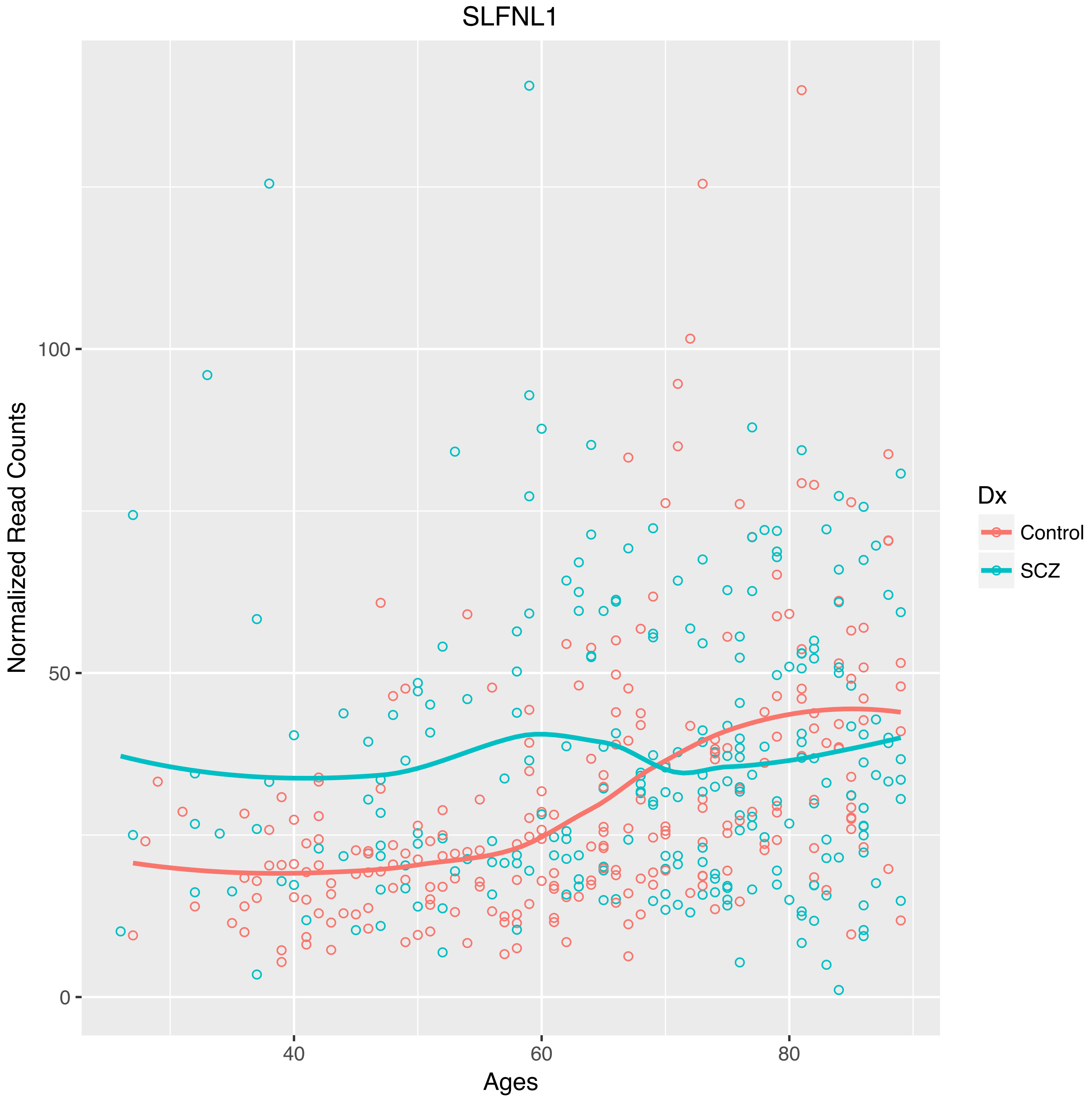

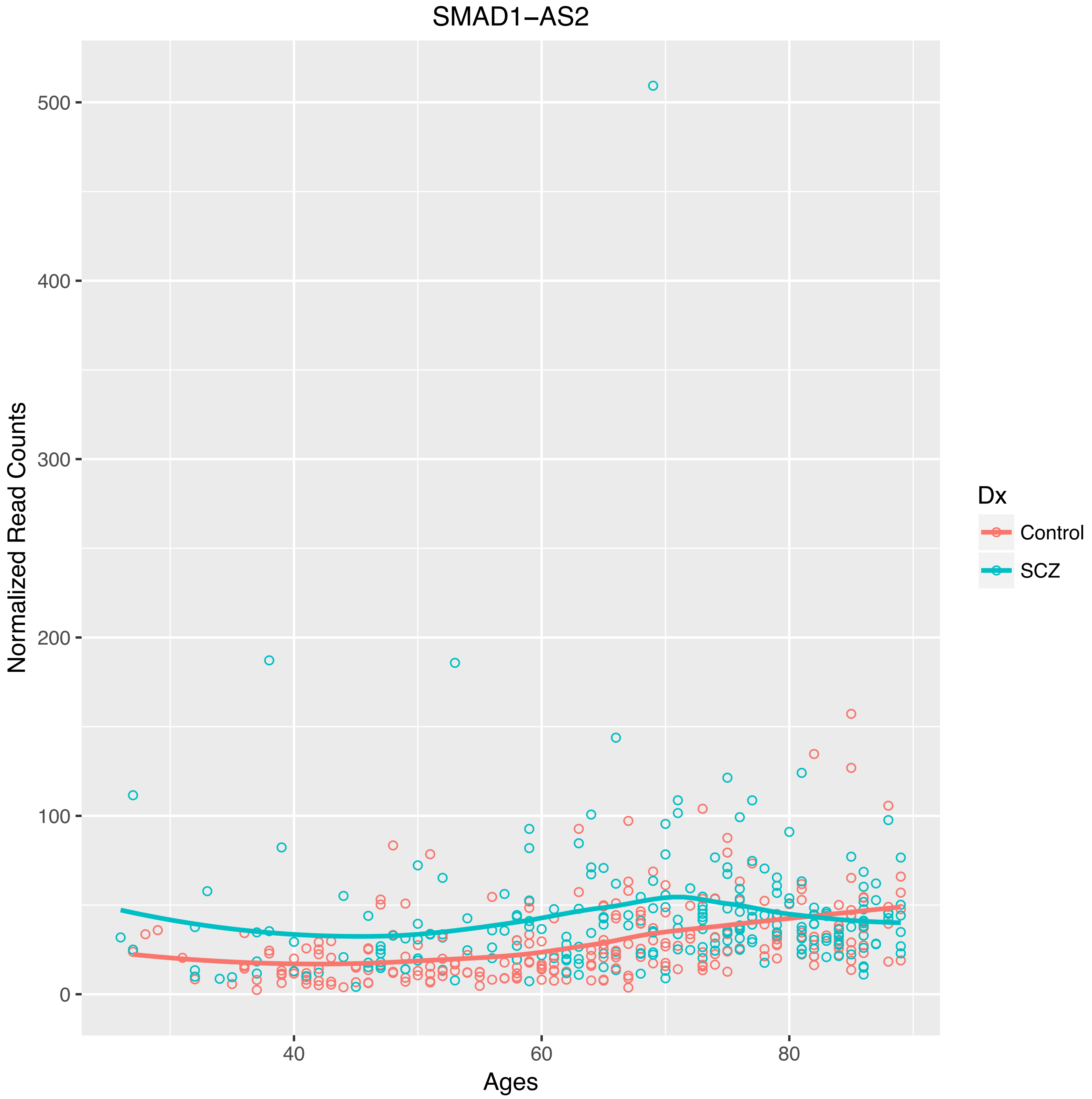

SMAD1−AS2
Normalized Read Counts
0
100
200
300
400
500
40
60
80
Ages
Dx
Control
SCZ

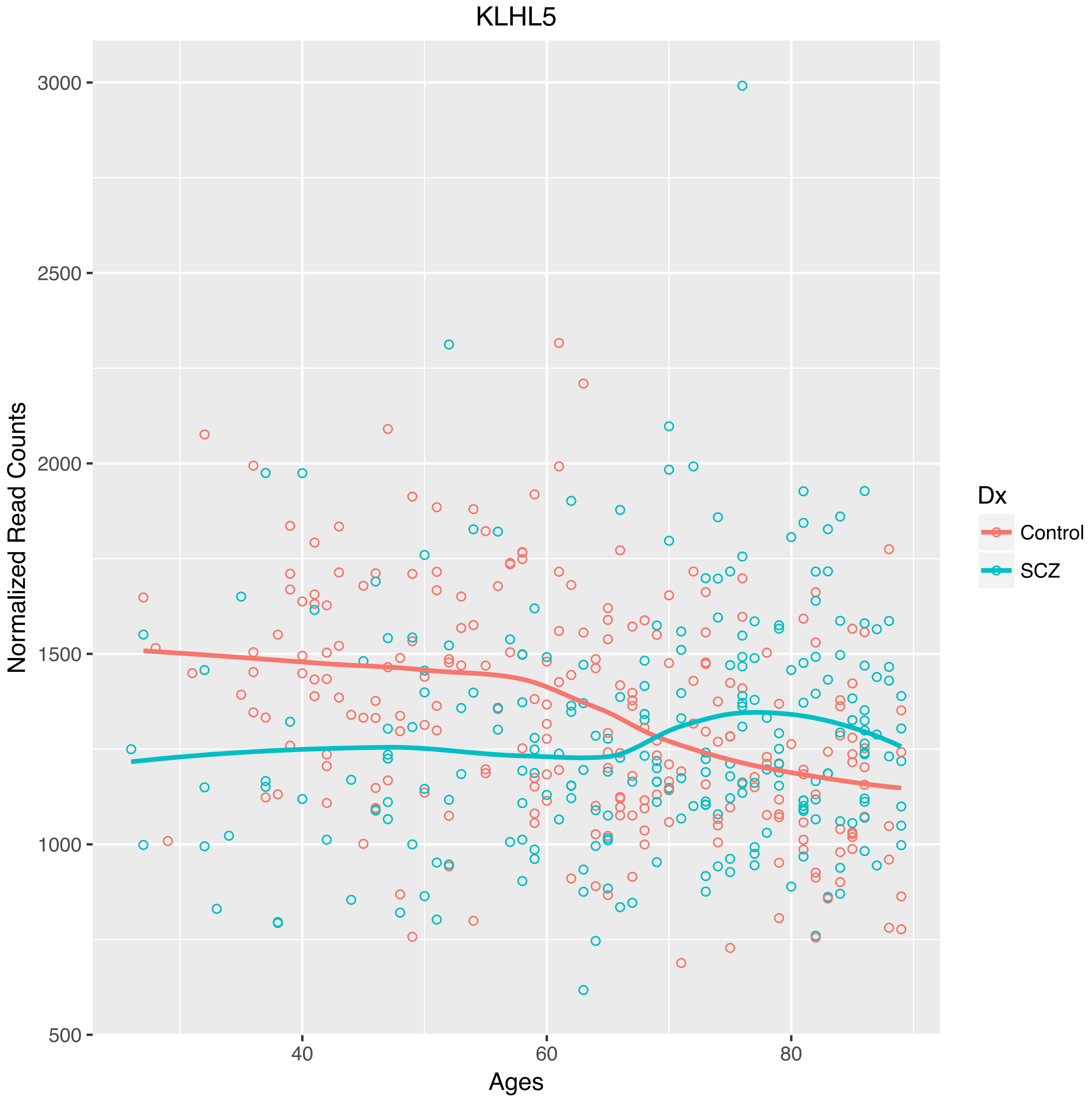
KLHL5
Normalized Read Counts
3000
2500
2000
1500
1000
500
40
60
80
Ages
Dx
Control
SCZ

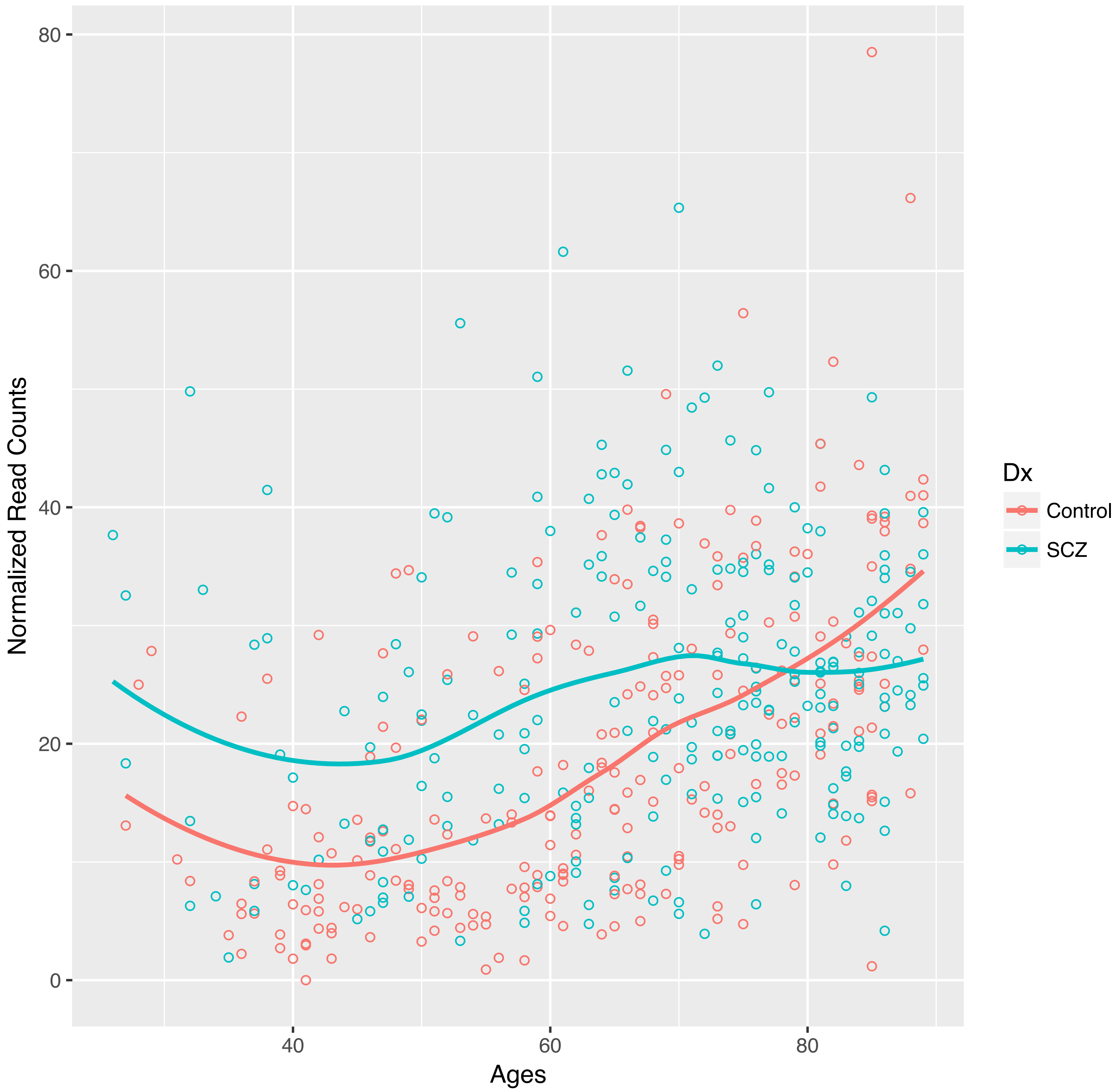
Normalized Read Counts
Ages
Dx
Control
SCZ
0
20
40
60
80
40
60
80

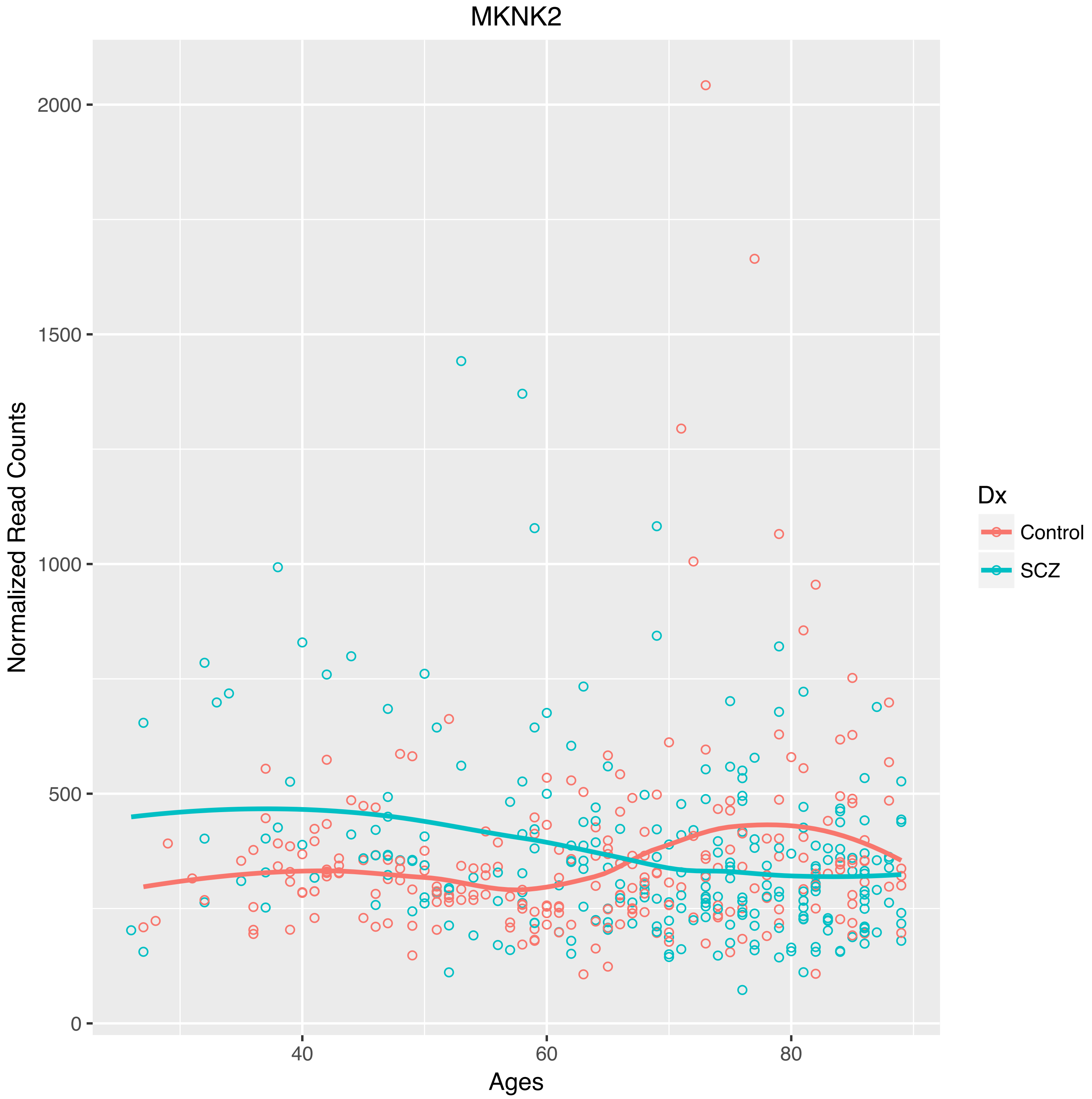
MKNK2
Normalized Read Counts
0
500
1000
1500
2000
40
60
80
Ages
Dx
Control
SCZ

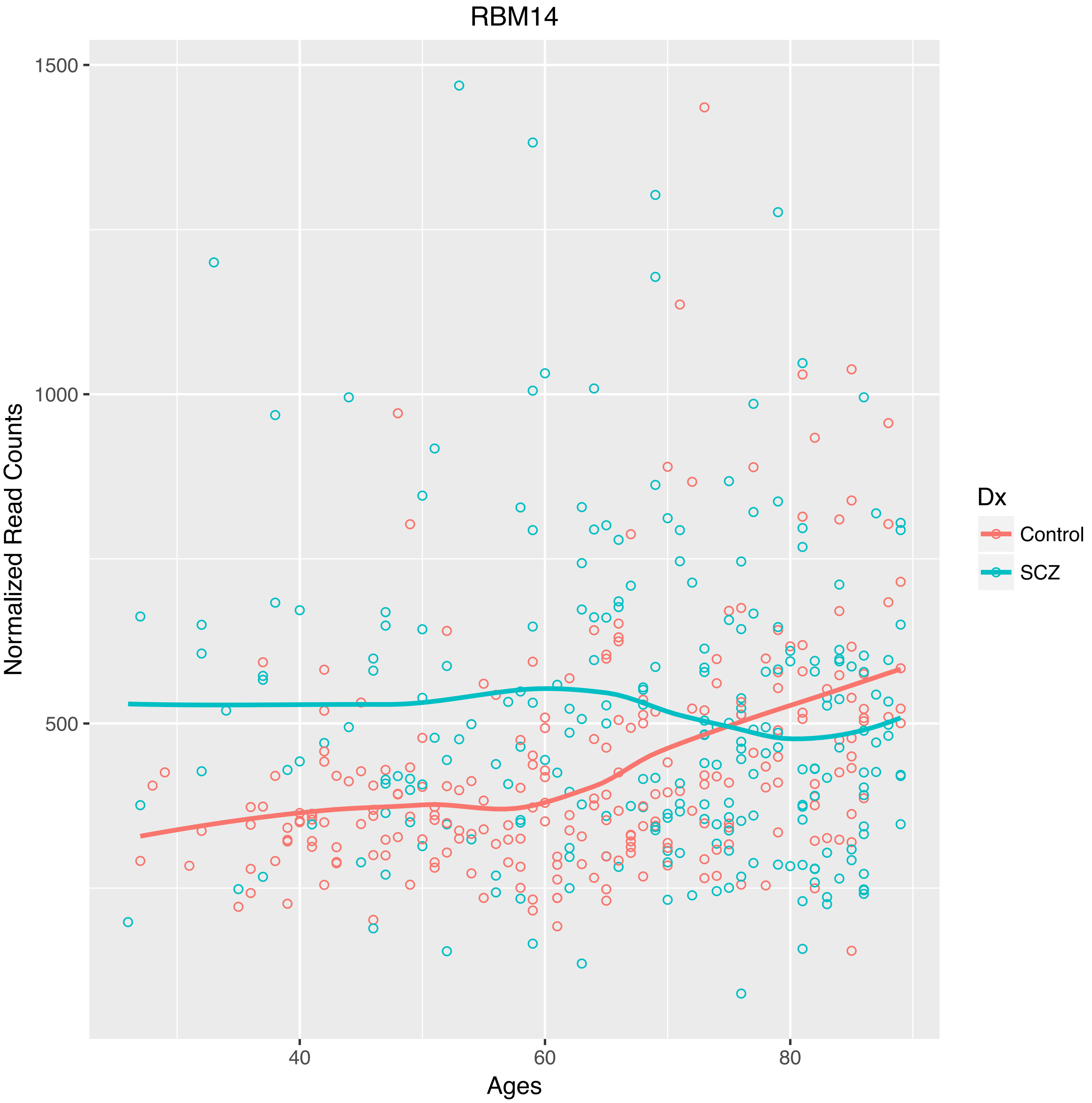
RBM14
1500
1000
500
Normalized Read Counts
40
60
80
Ages
Dx
Control
SCZ

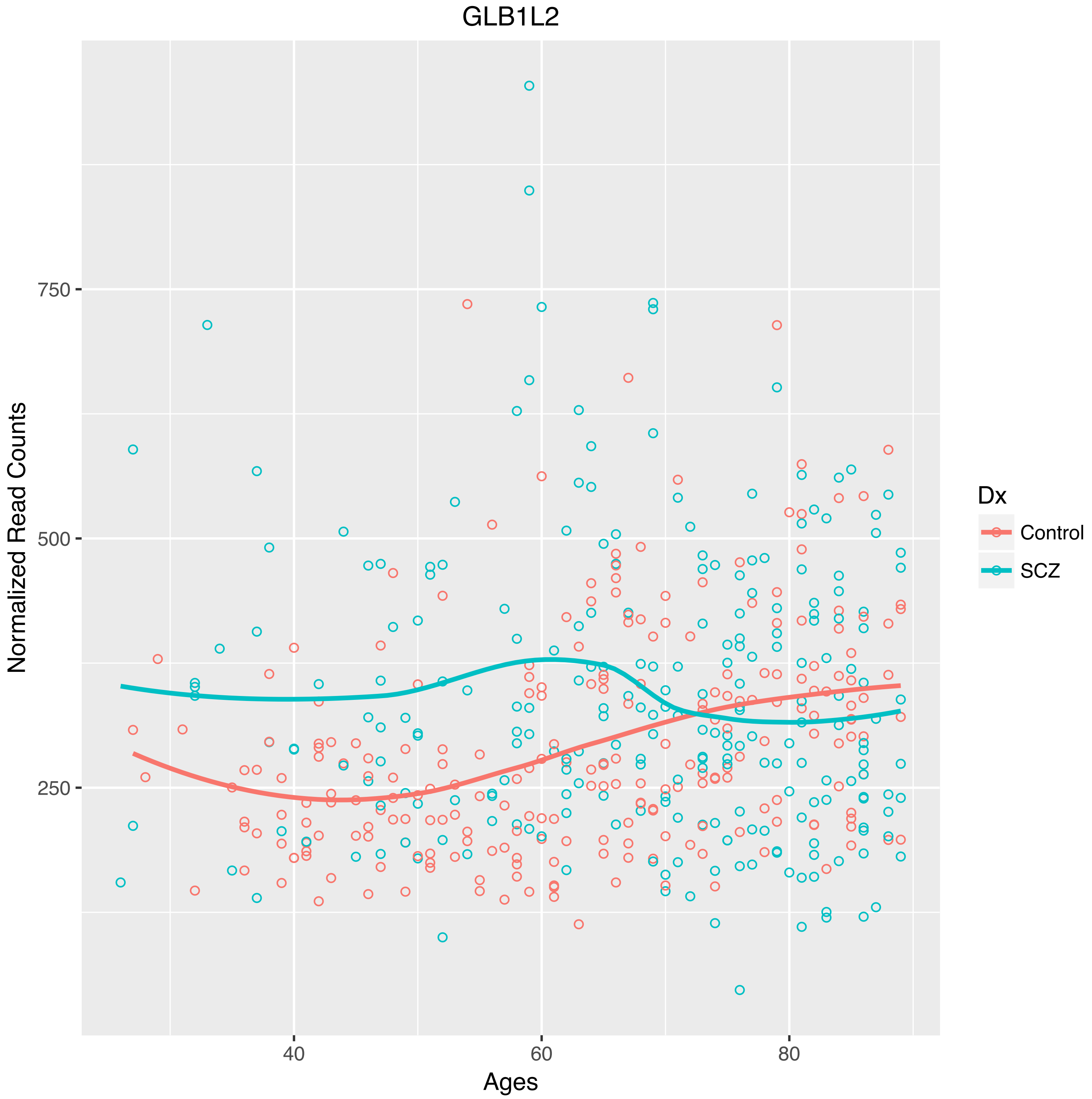
GLB1L2
Normalized Read Counts
750
500
250
40
60
80
Ages
Dx
Control
SCZ

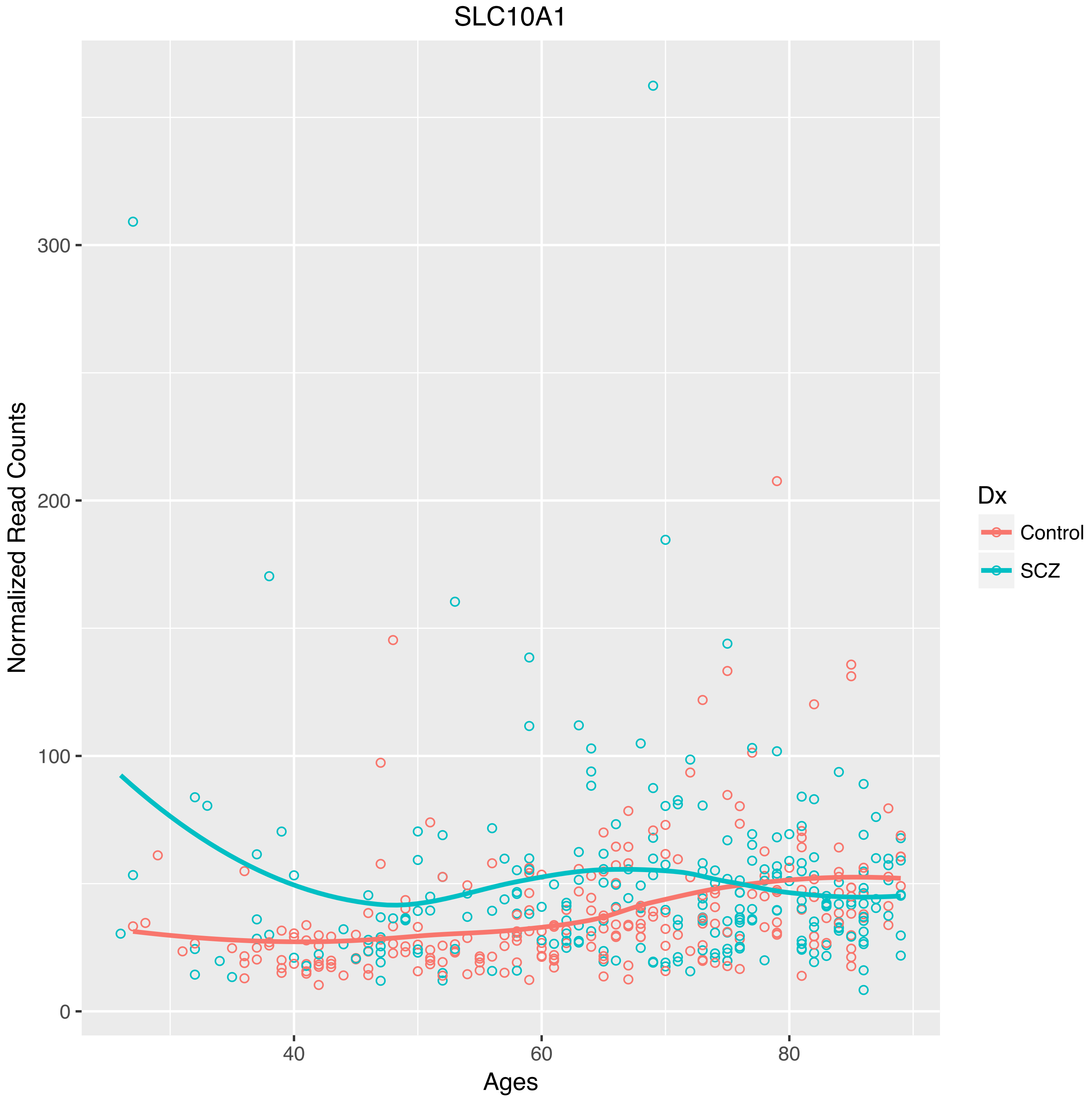

SLC10A1
Normalized Read Counts
300
200
100
0
40
60
80
Ages
Dx
Control
SCZ

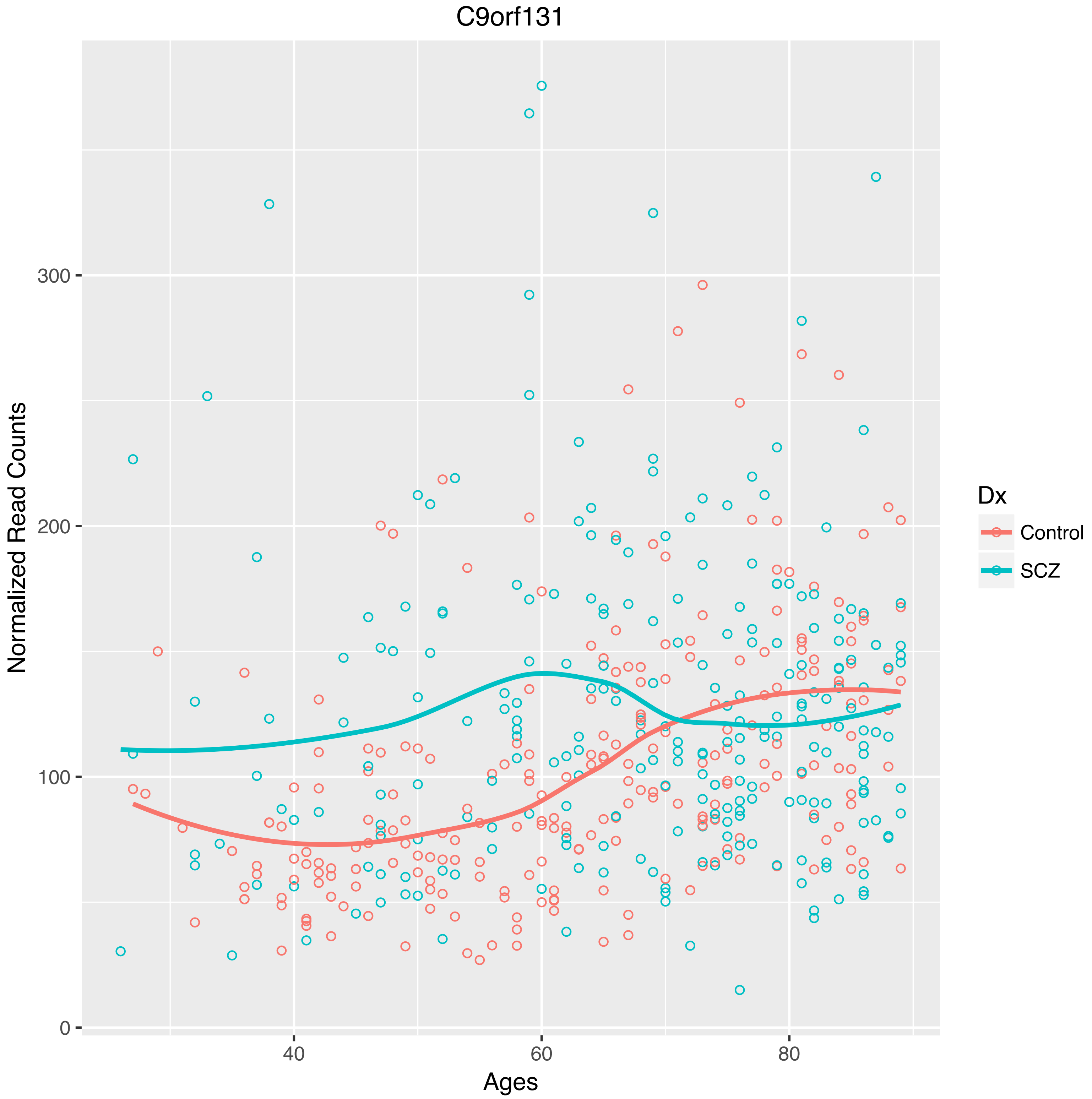
C9orf131
Normalized Read Counts
300
200
100
0
40
60
80
Ages
Dx
Control
SCZ

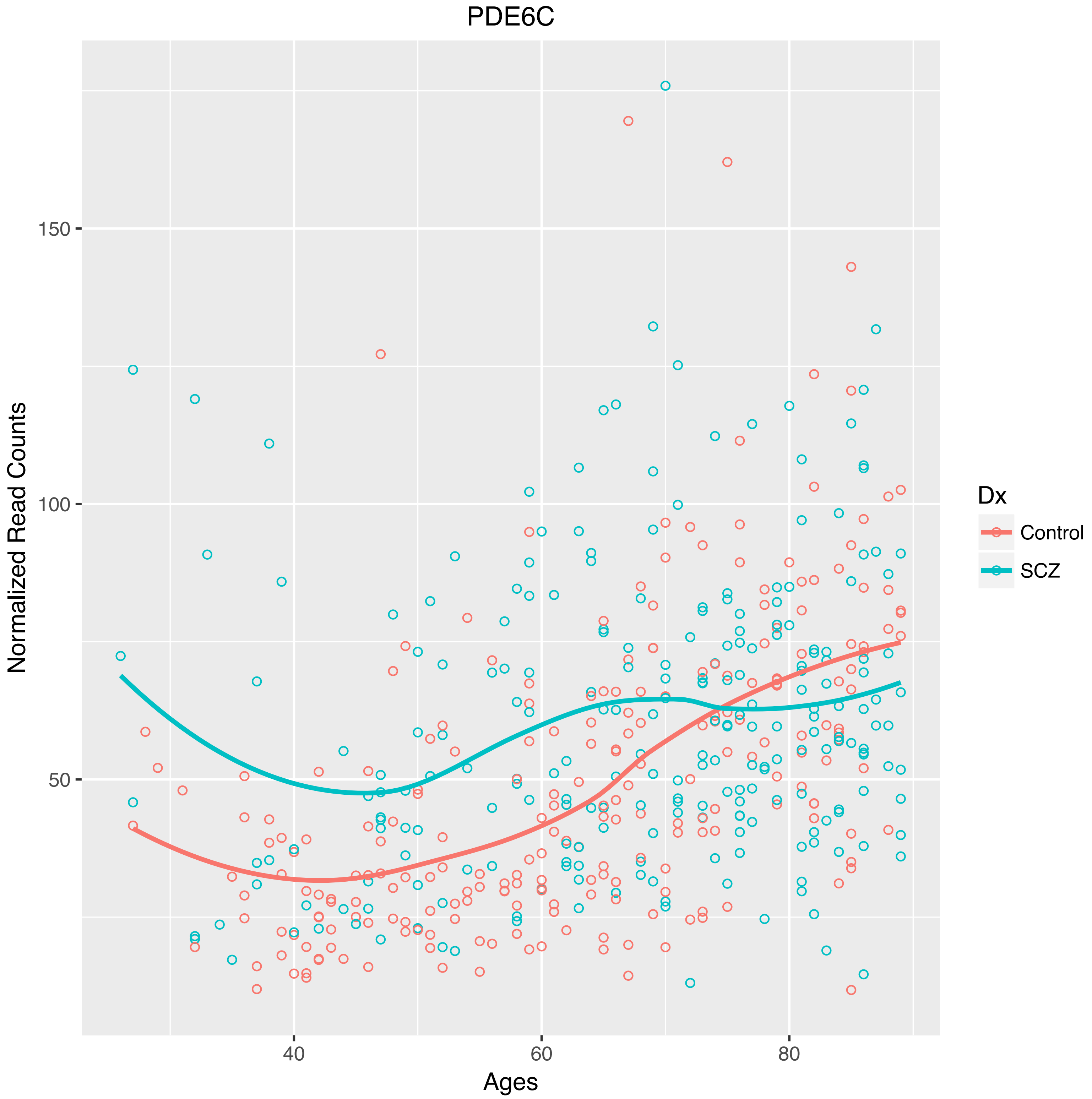

PDE6C
Normalized Read Counts
150
100
50
40
60
80
Ages
Dx
Control
SCZ

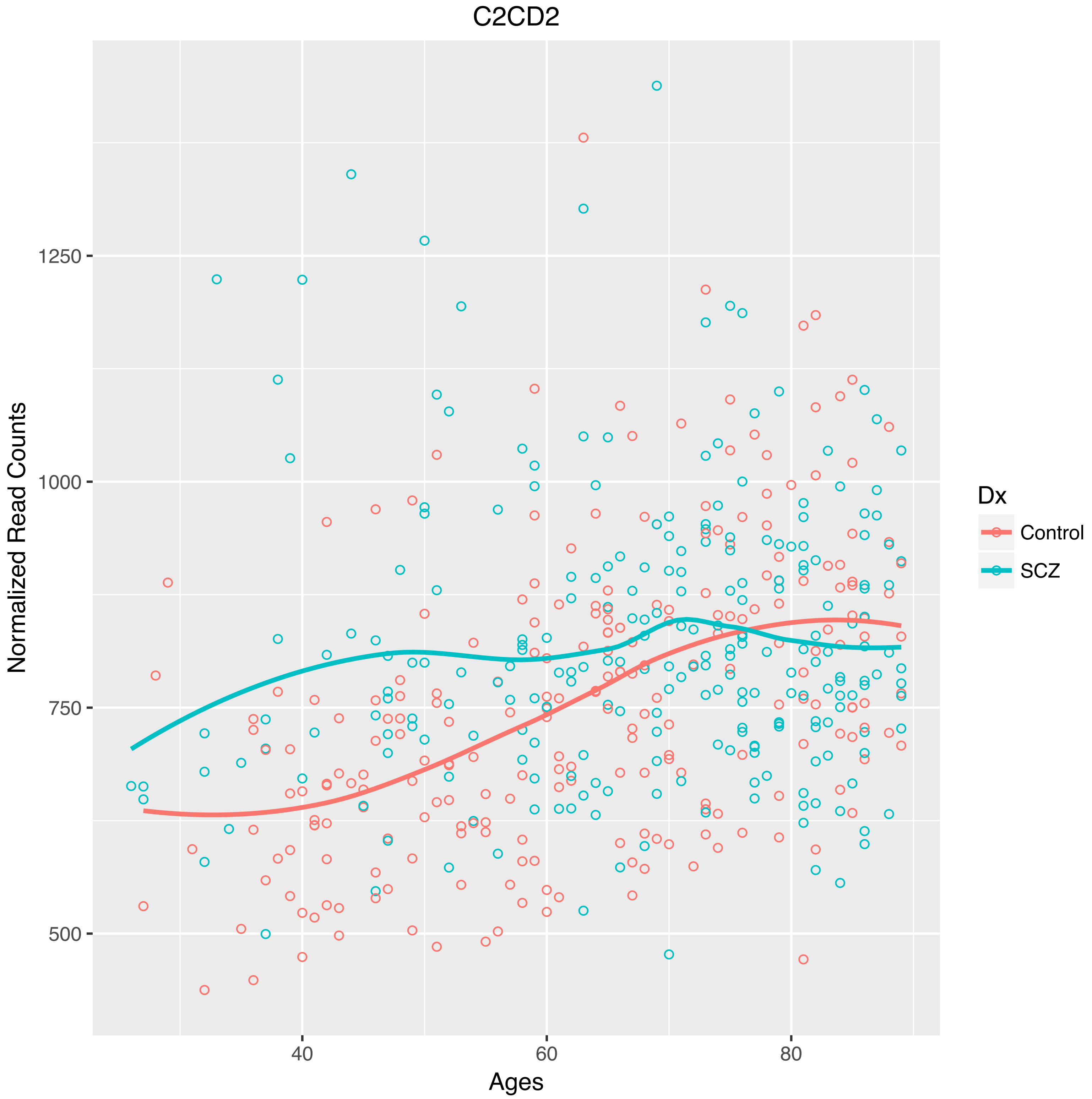

C2CD2
Normalized Read Counts
1250
1000
750
500
40
60
80
Ages
Dx
Control
SCZ

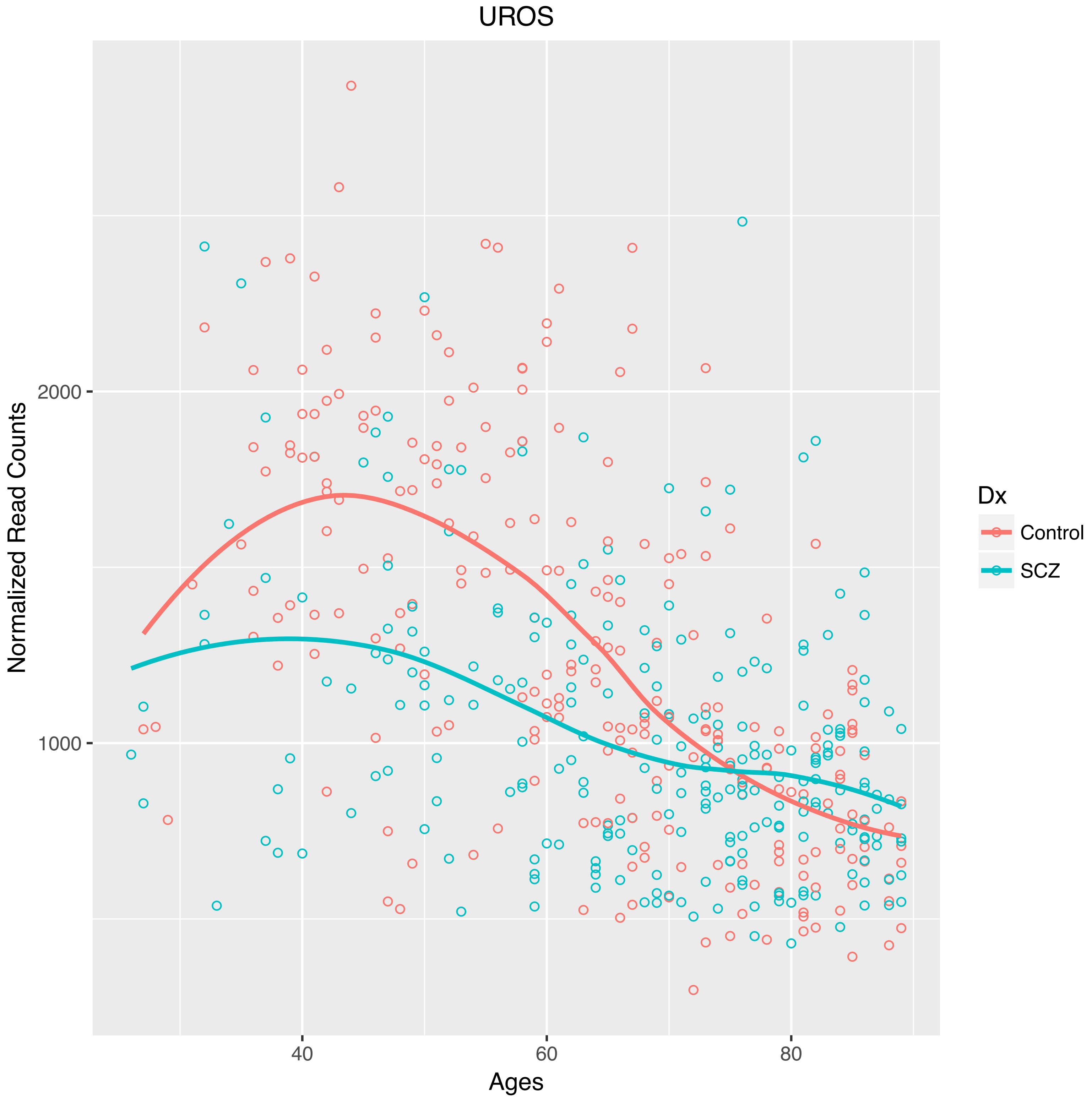

UROS
Normalized Read Counts
2000
1000
40
60
80
Ages
Dx
Control
SCZ

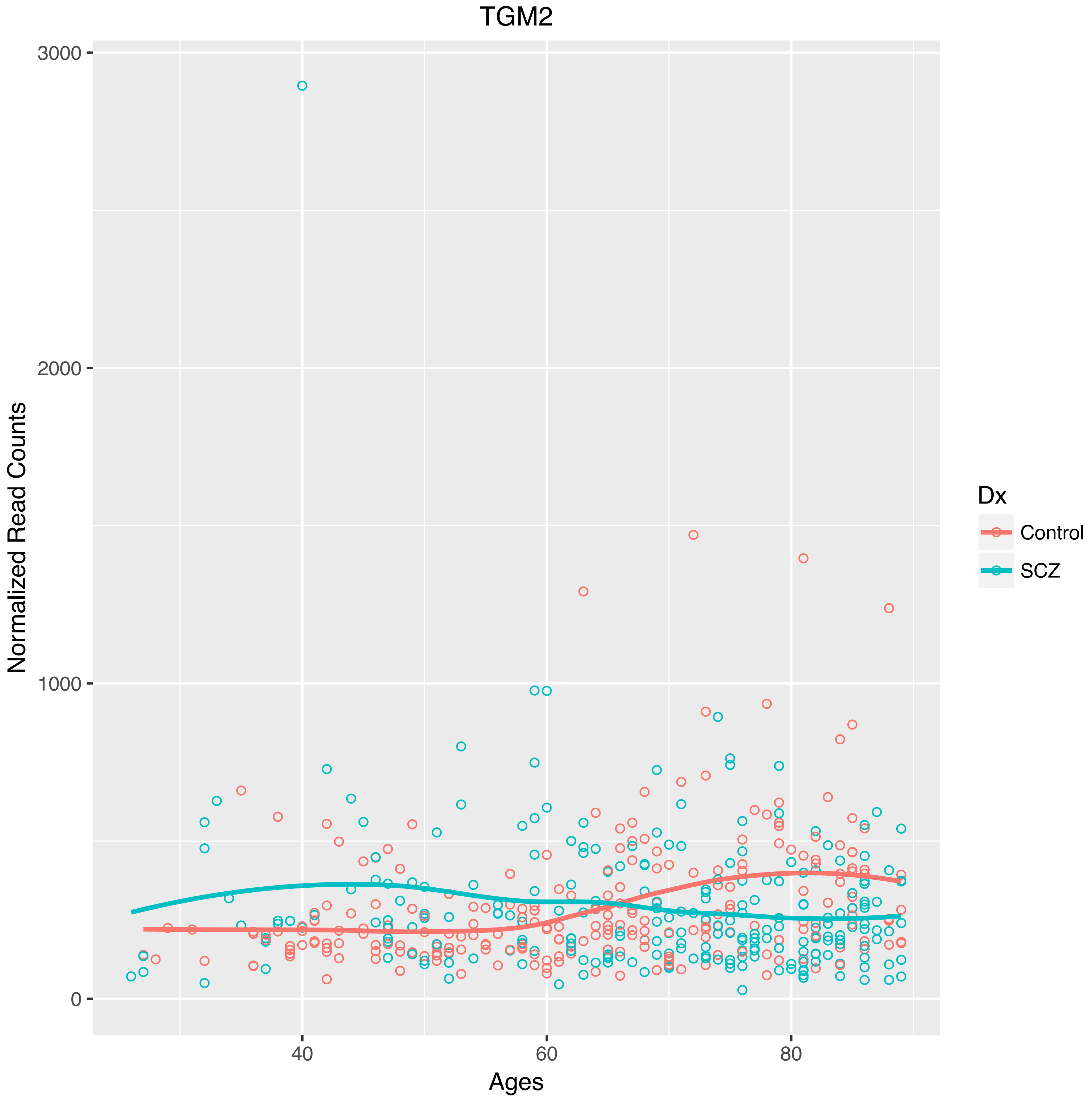
TGM2
3000
2000
1000
0
Normalized Read Counts
40
60
80
Ages
Dx
Control
SCZ

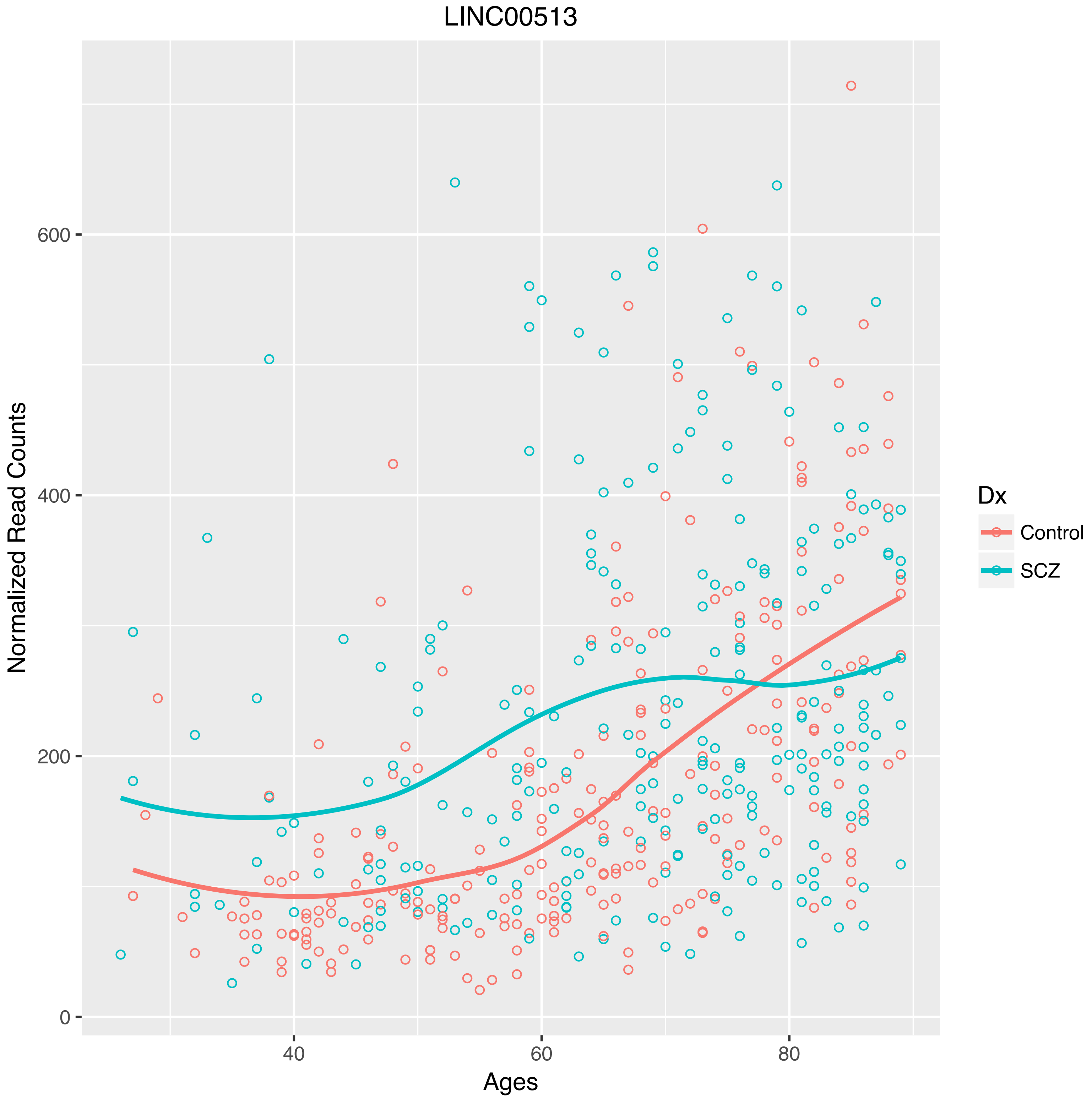
LINC00513
Normalized Read Counts
Ages
0
200
400
600
40
60
80
Dx
Control
SCZ

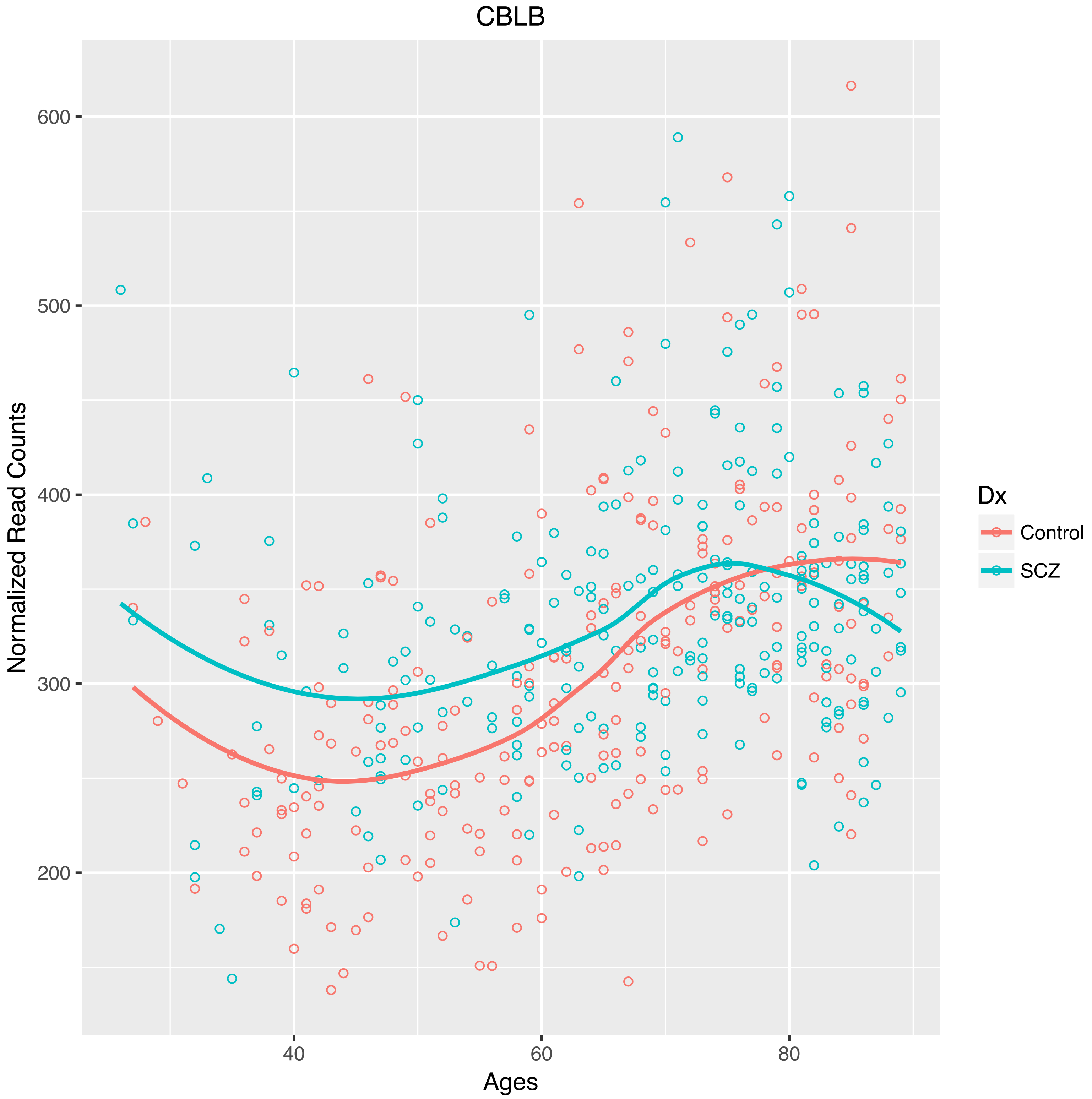
CBLB
Normalized Read Counts
200
300
400
500
600
Ages
40
60
80
Dx
Control
SCZ

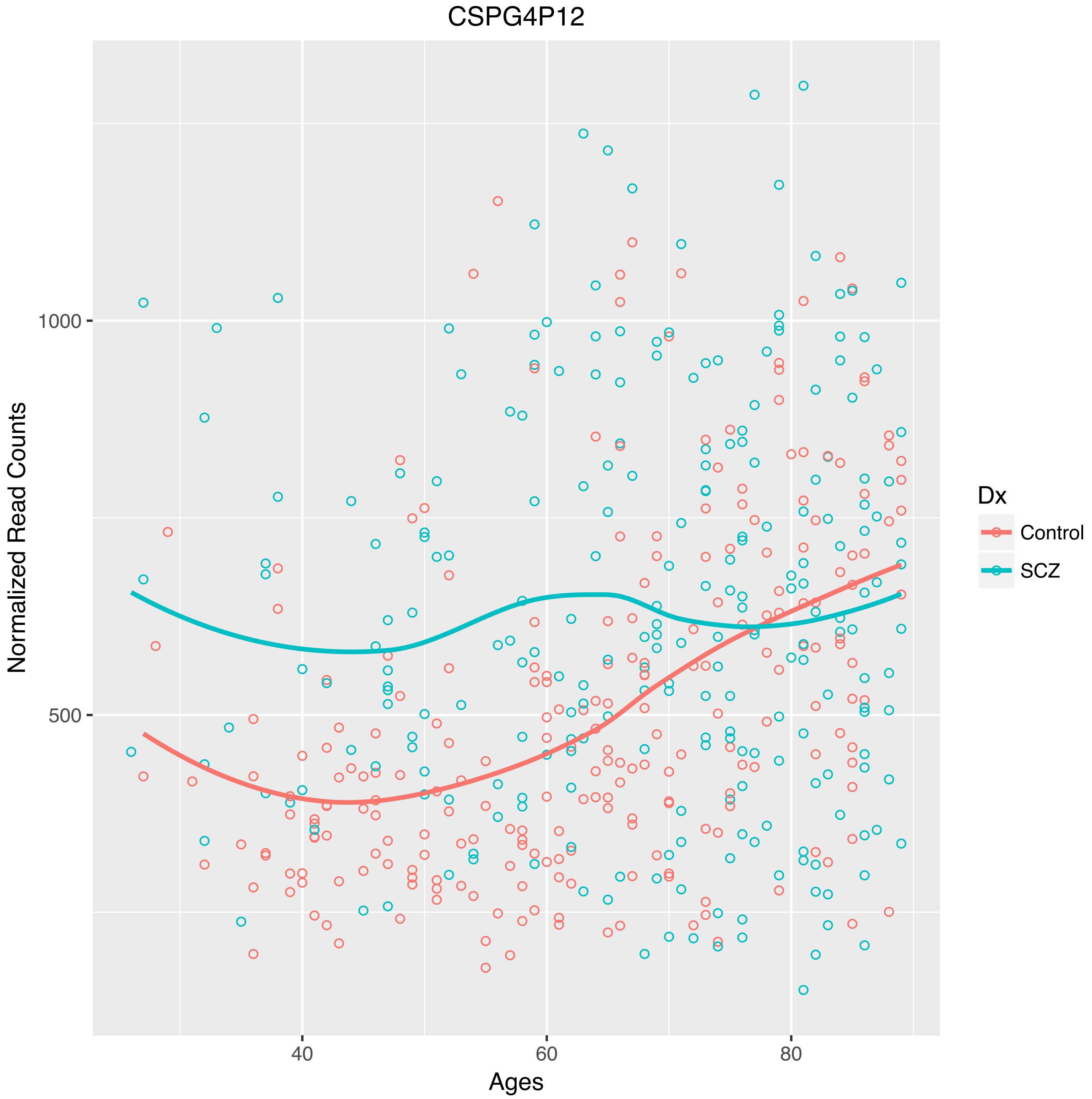

CSPG4P12
Normalized Read Counts
1000
500
40
60
80
Ages
Dx
Control
SCZ

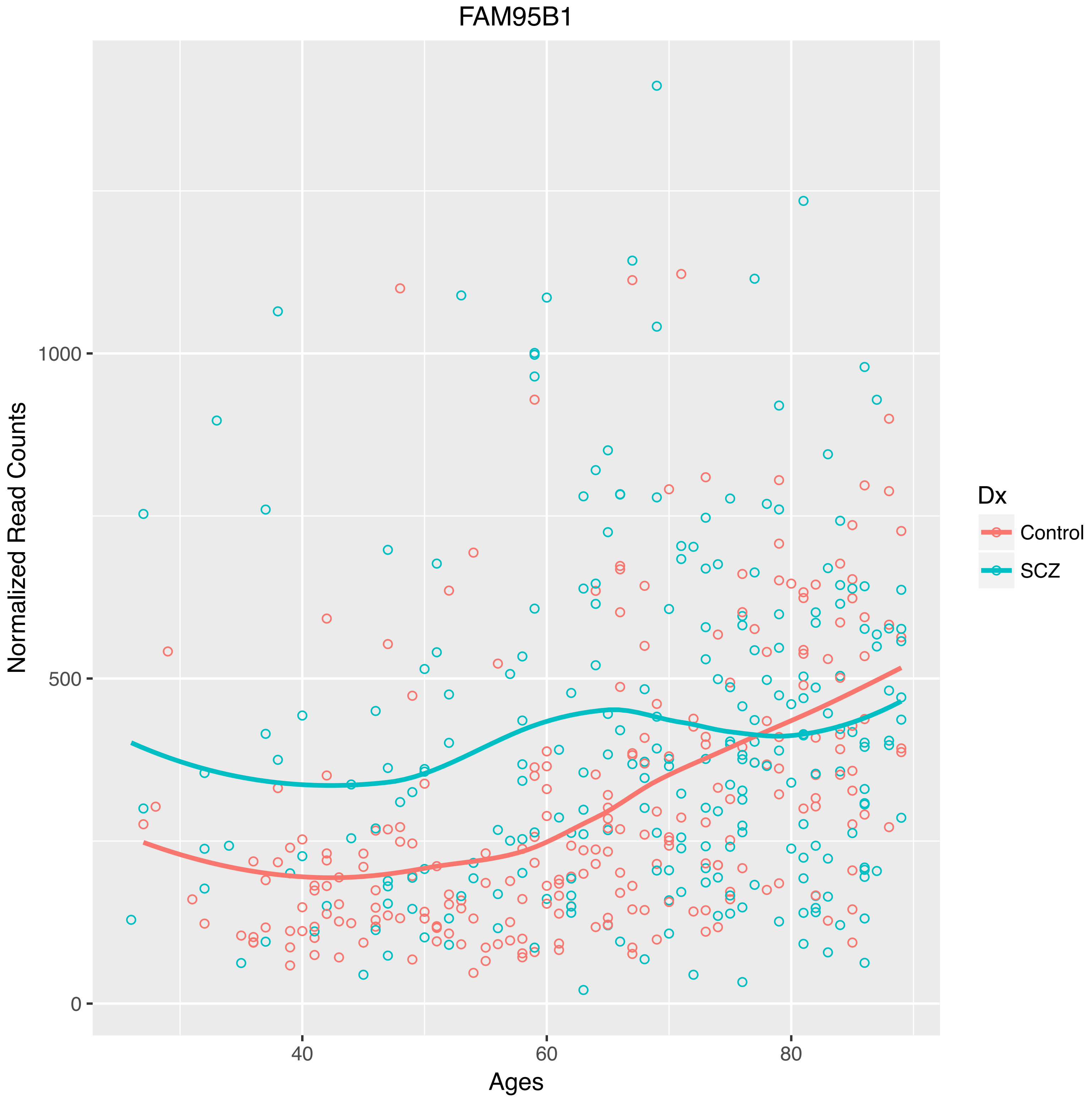
FAM95B1
Normalized Read Counts
1000
500
0
40
60
80
Ages
Dx
Control
SCZ

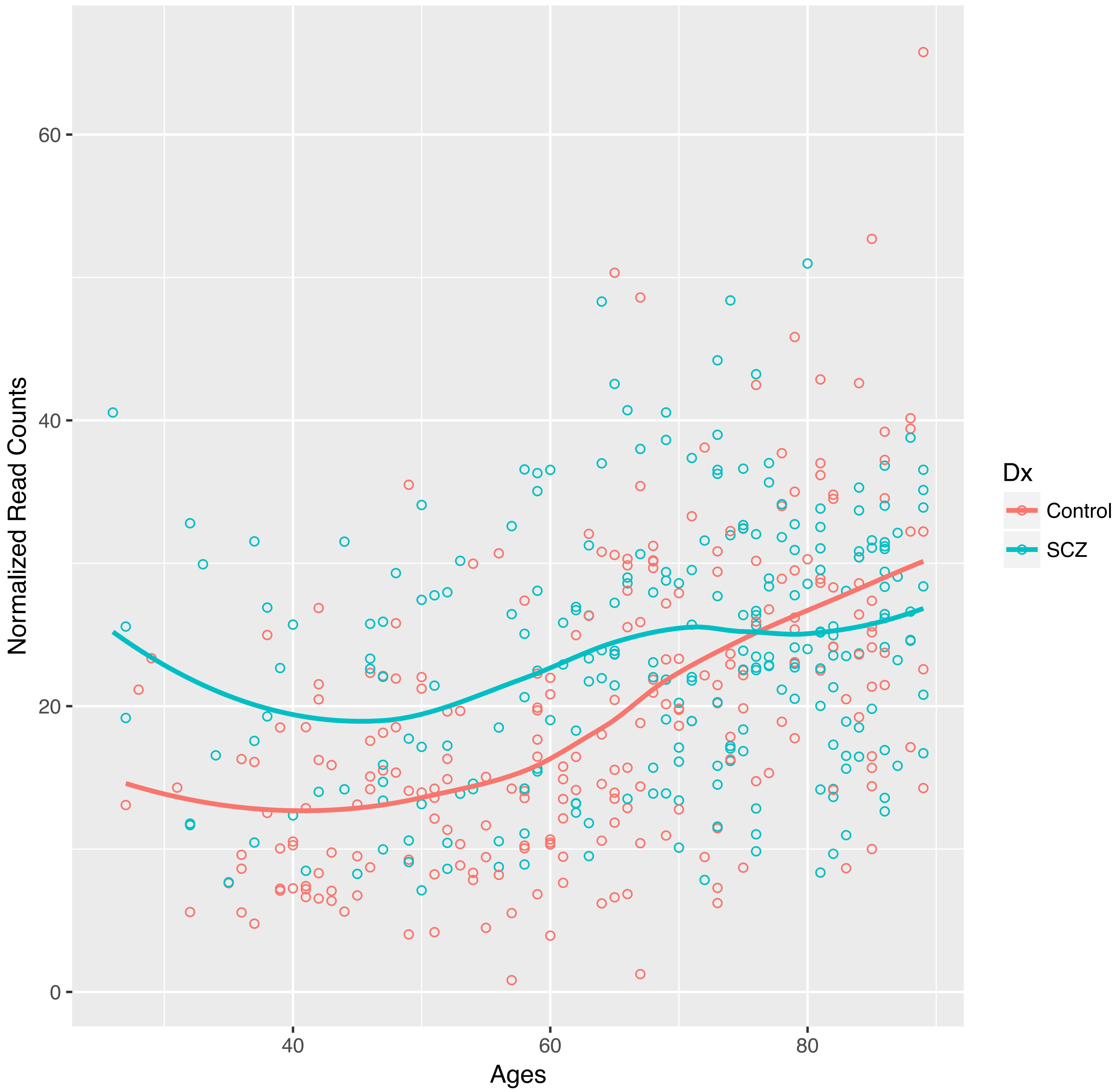

Normalized Read Counts
0
20
40
60
Ages
40
60
80
Dx
Control
SCZ

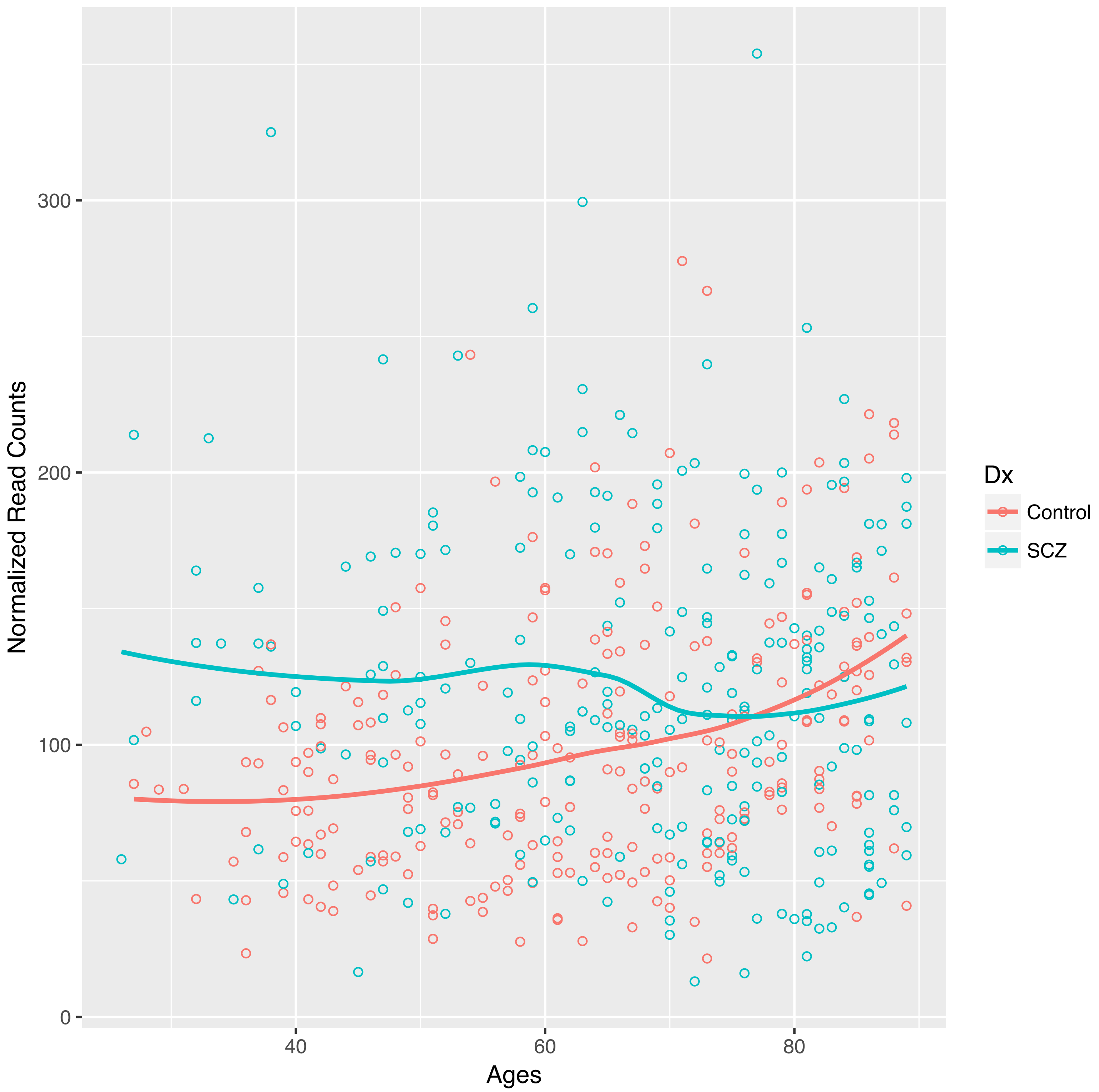

Normalized Read Counts
300
200
100
0
40
60
80
Ages
Dx
Control
SCZ

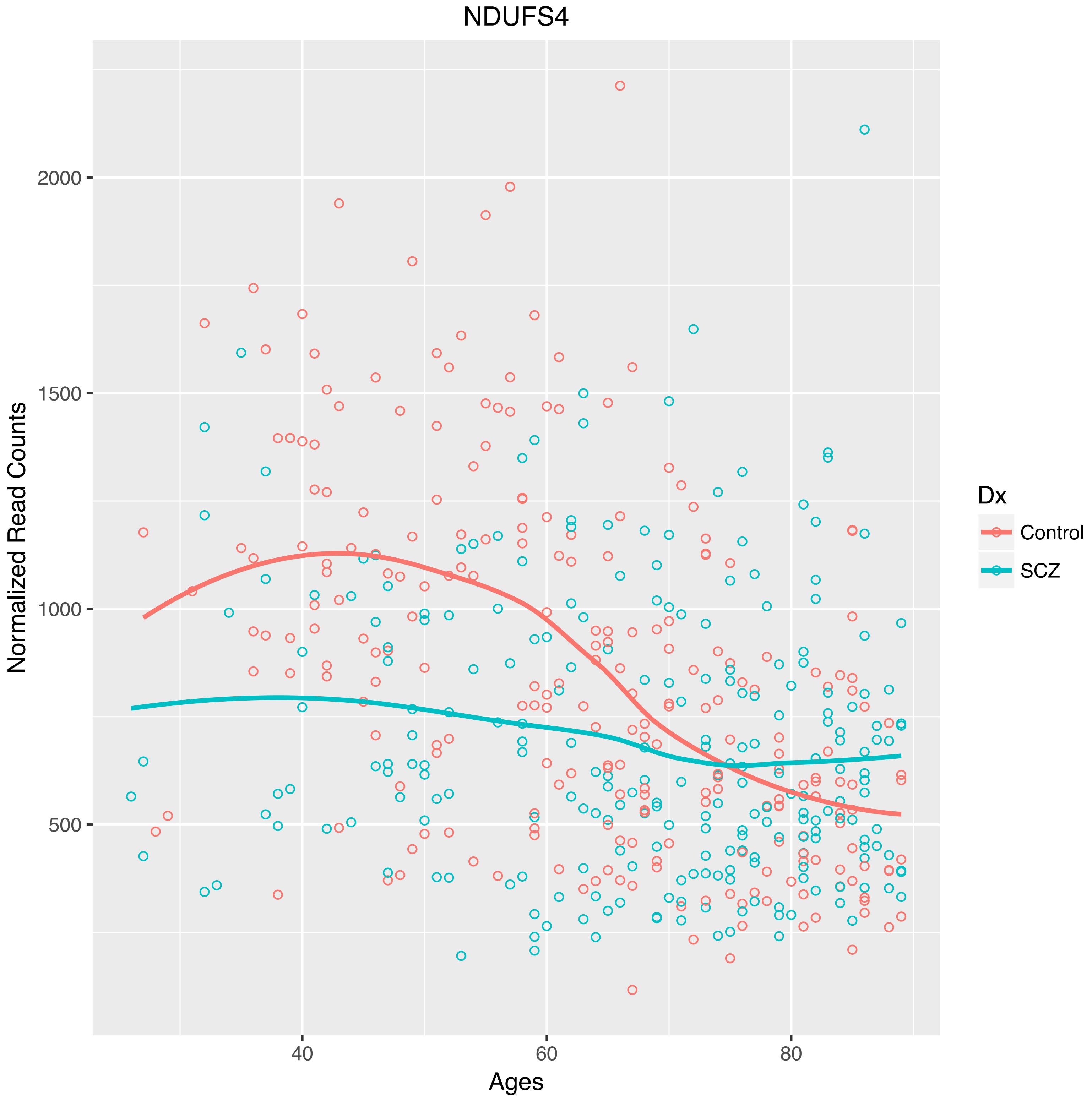

NDUFS4
Normalized Read Counts
2000
1500
1000
500
40
60
80
Ages
Dx
Control
SCZ

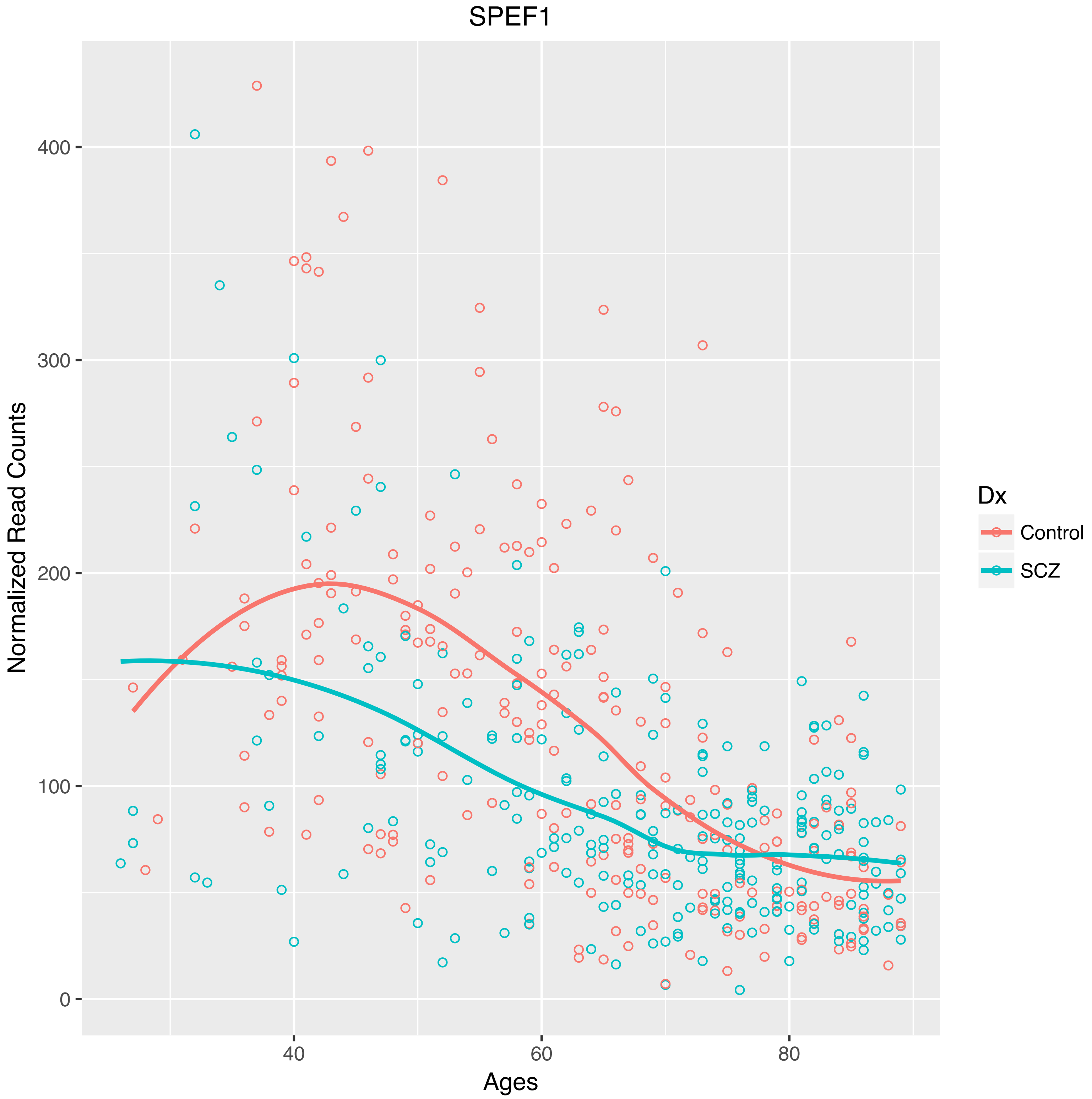
SPEF1
Normalized Read Counts
0
100
200
300
400
40
60
80
Ages
Dx
Control
SCZ

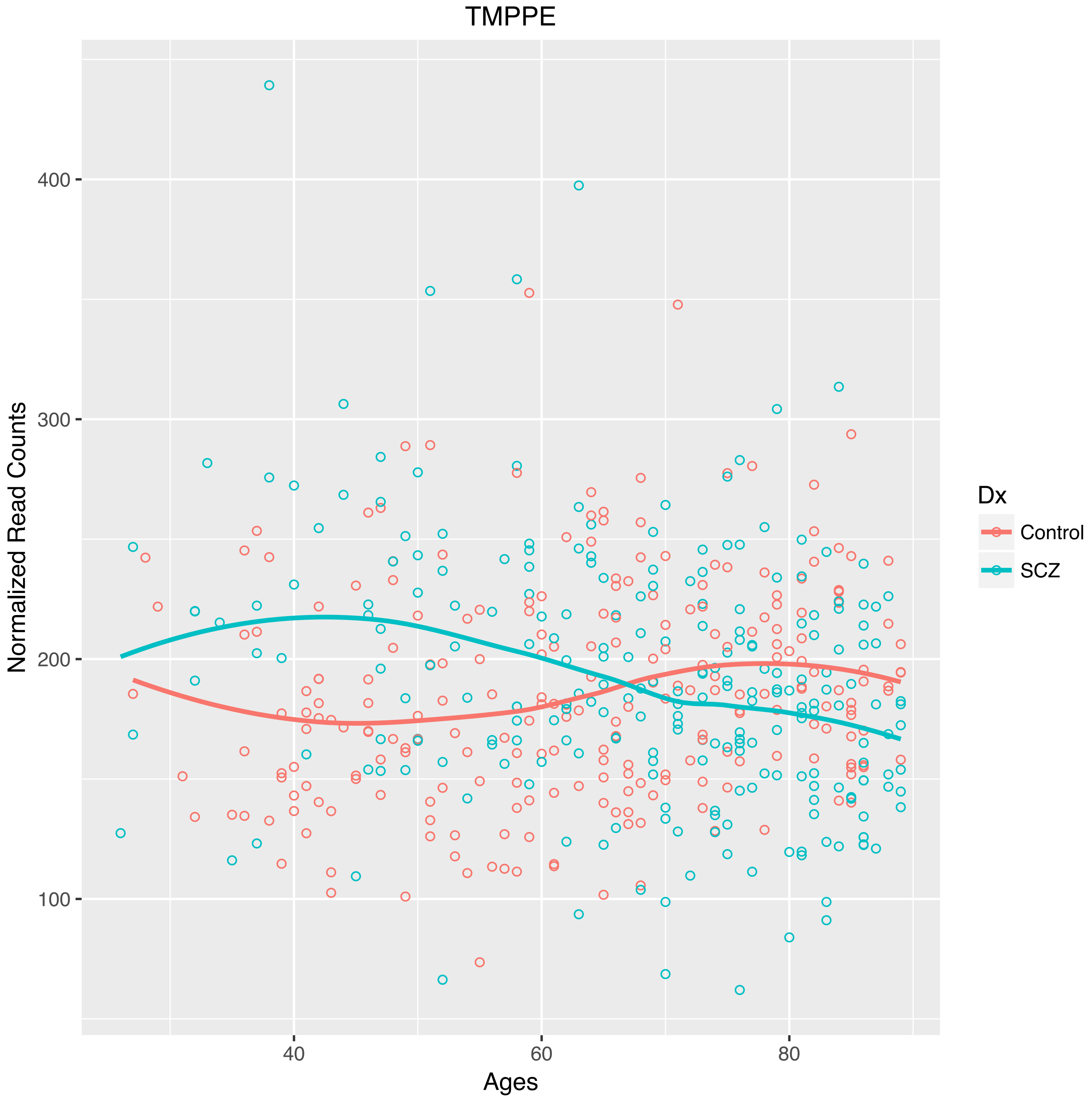

TMPPE
Normalized Read Counts
400
300
200
100
40
60
80
Ages
Dx
Control
SCZ

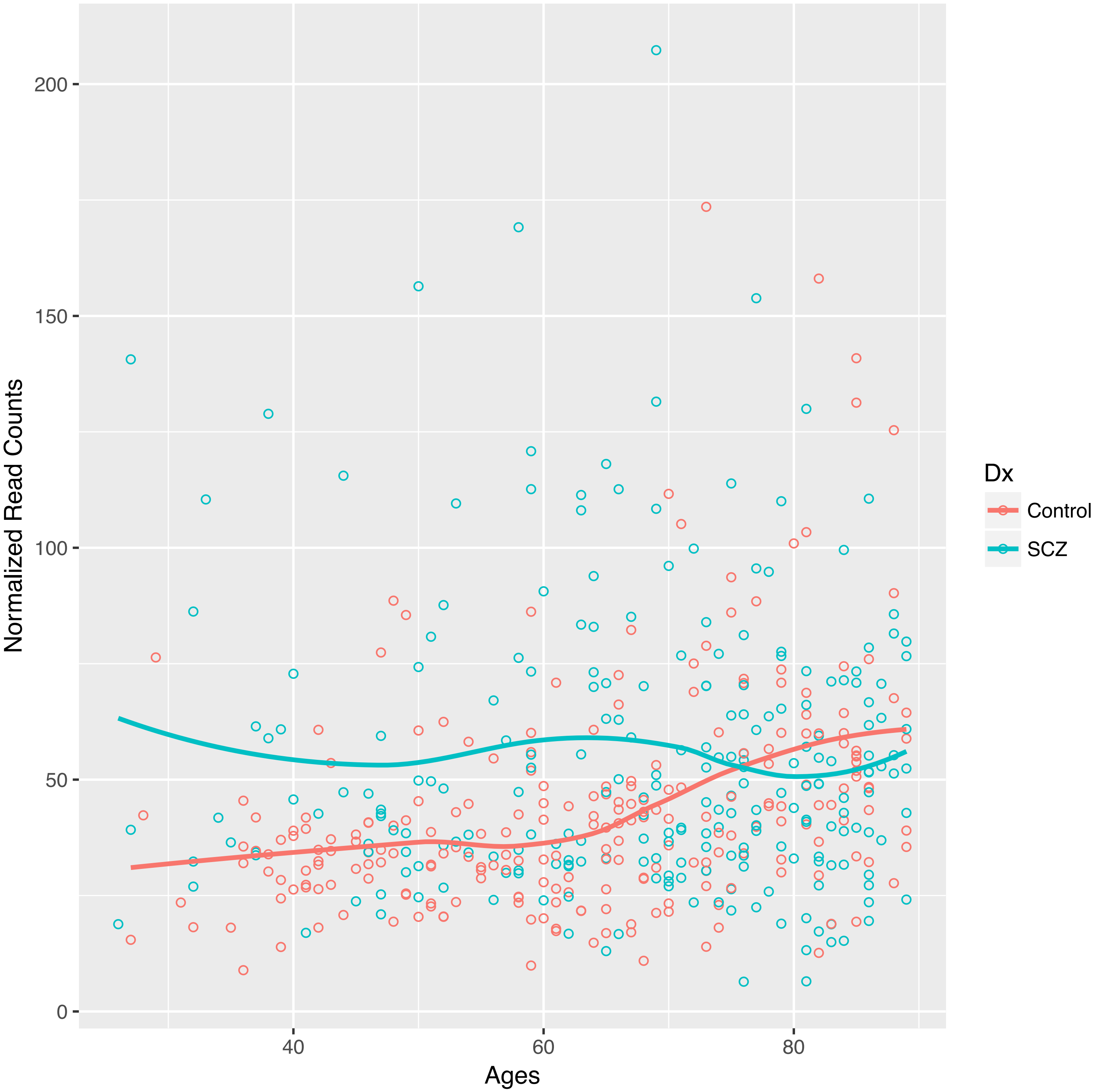

Normalized Read Counts
0
50
100
150
200
40
60
80
Ages
Dx
Control
SCZ

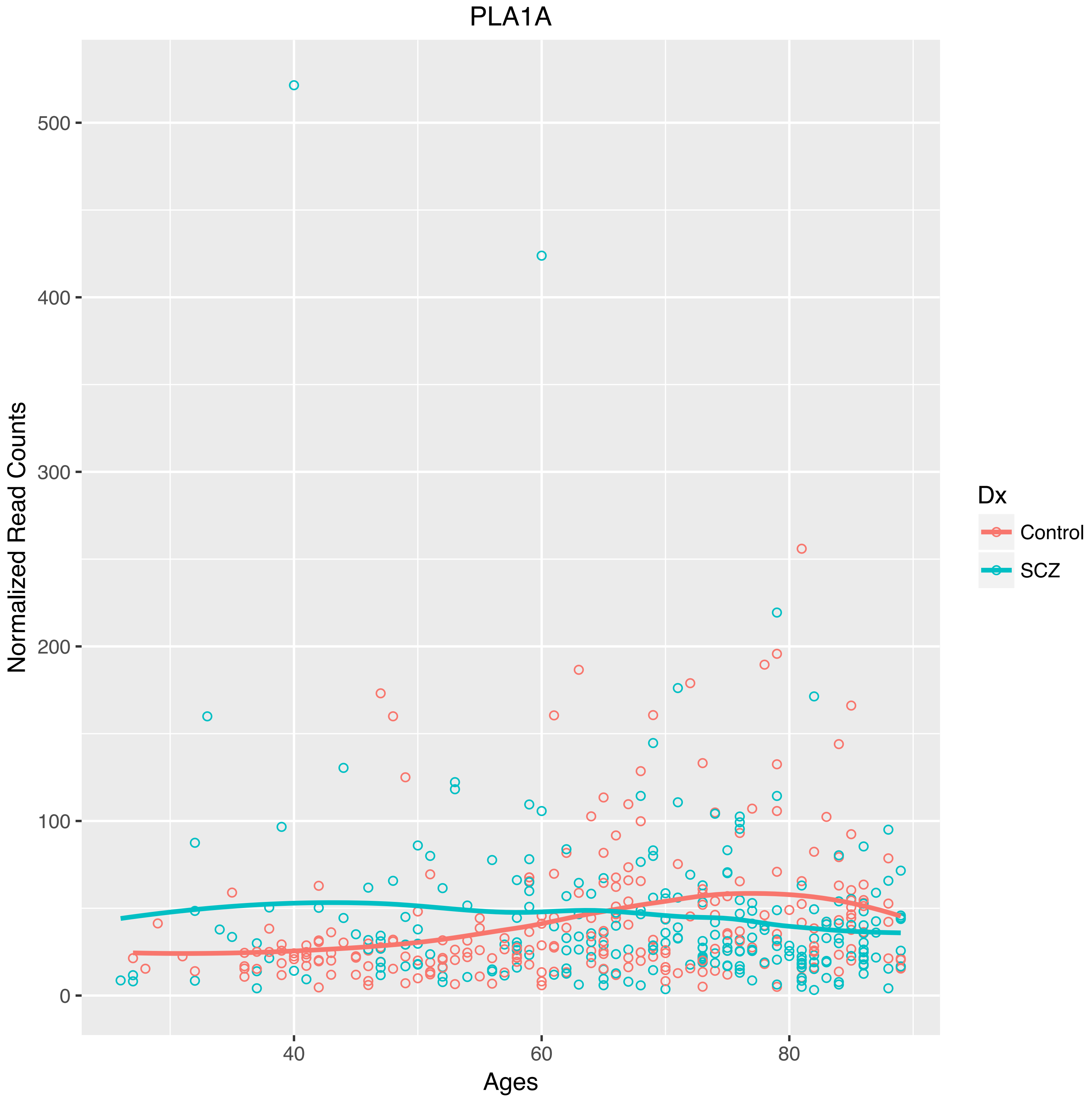

PLA1A
Normalized Read Counts
0
100
200
300
400
500
40
60
80
Ages
Dx
Control
SCZ

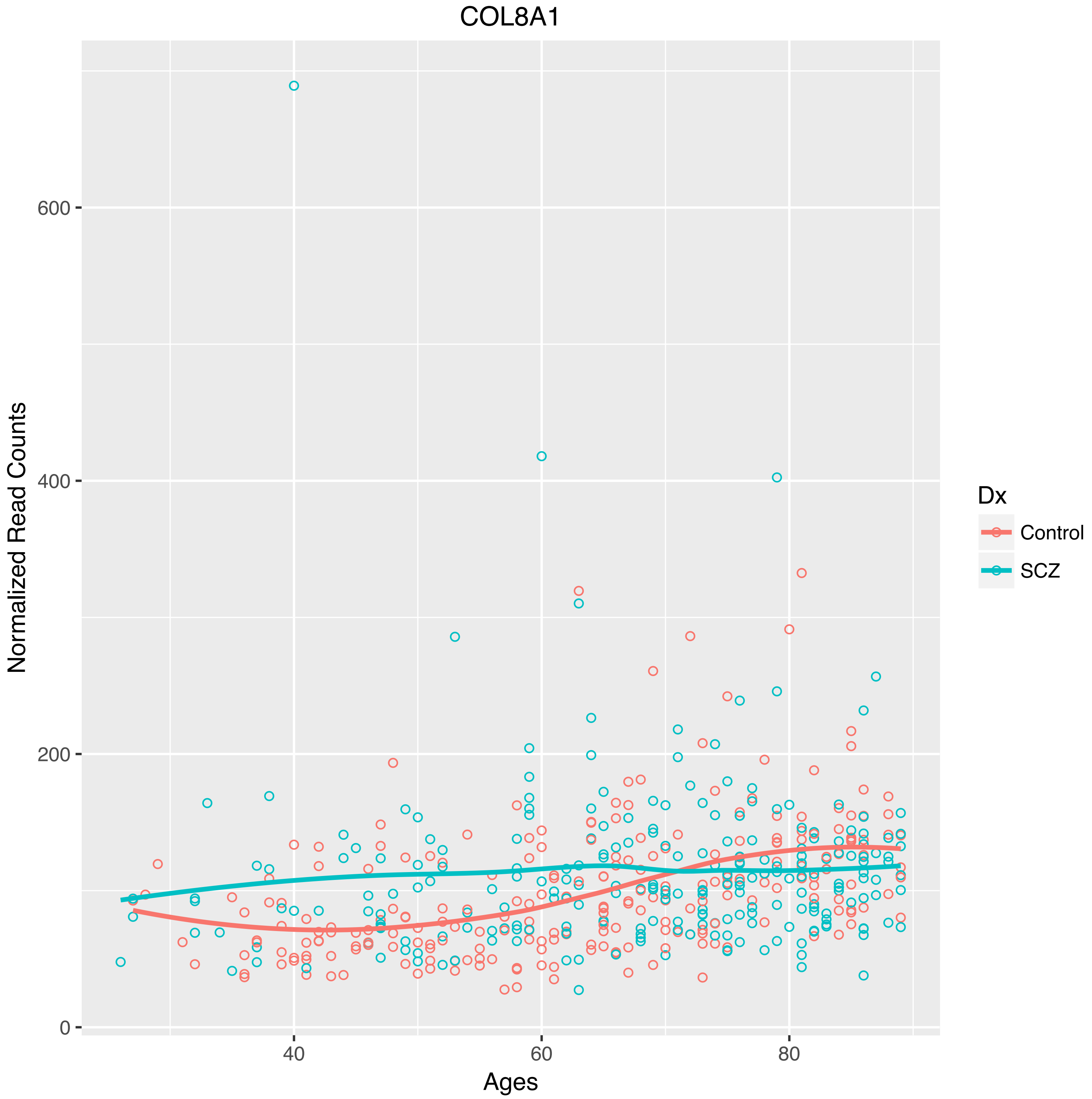
COL8A1
Normalized Read Counts
0
200
400
600
Ages
40
60
80
Dx
Control
SCZ

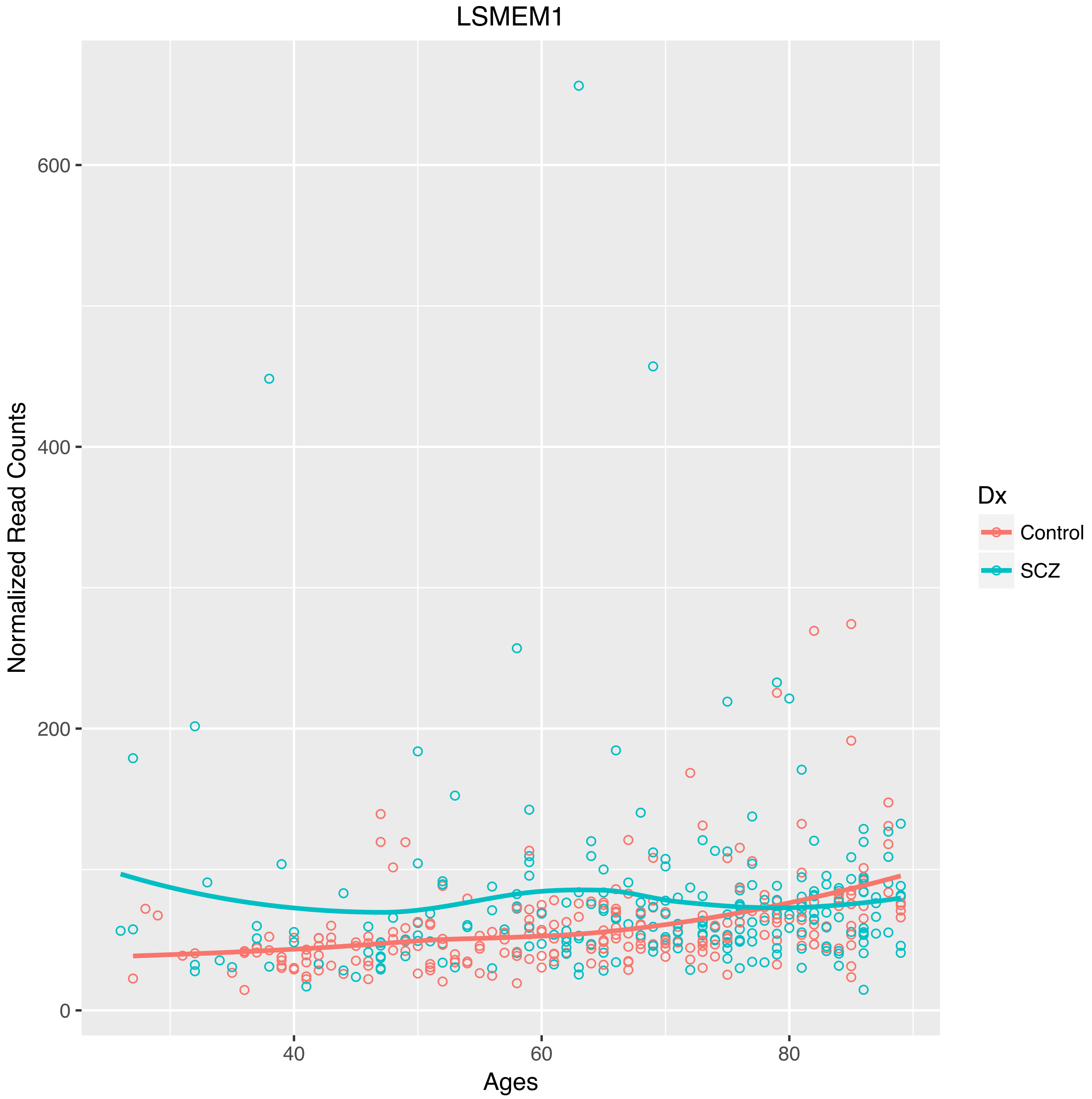
LSMEM1
Normalized Read Counts
Ages
0
200
400
600
40
60
80
Dx
Control
SCZ

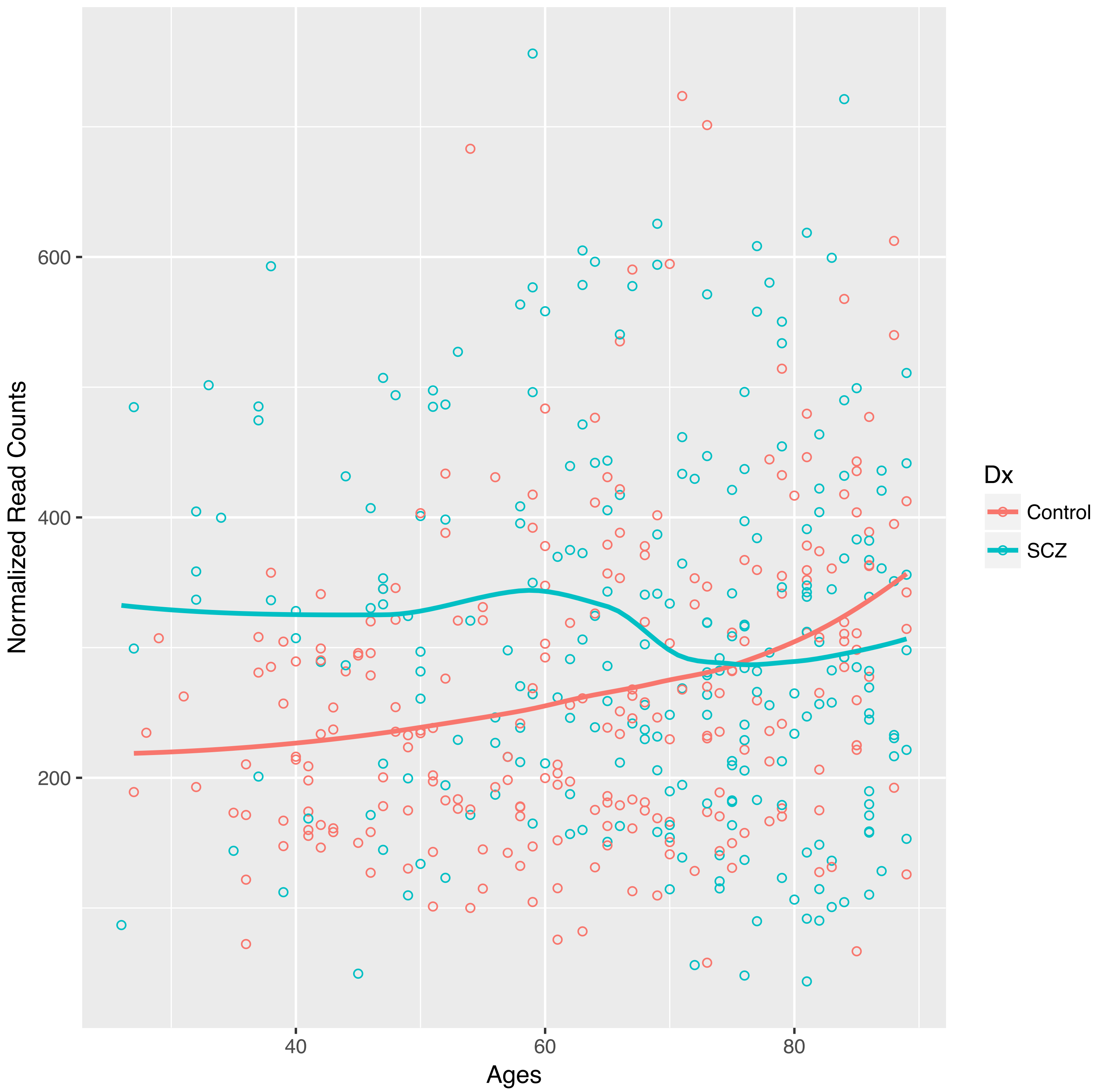

Normalized Read Counts
600
400
200
40
60
80
Ages
Dx
Control
SCZ

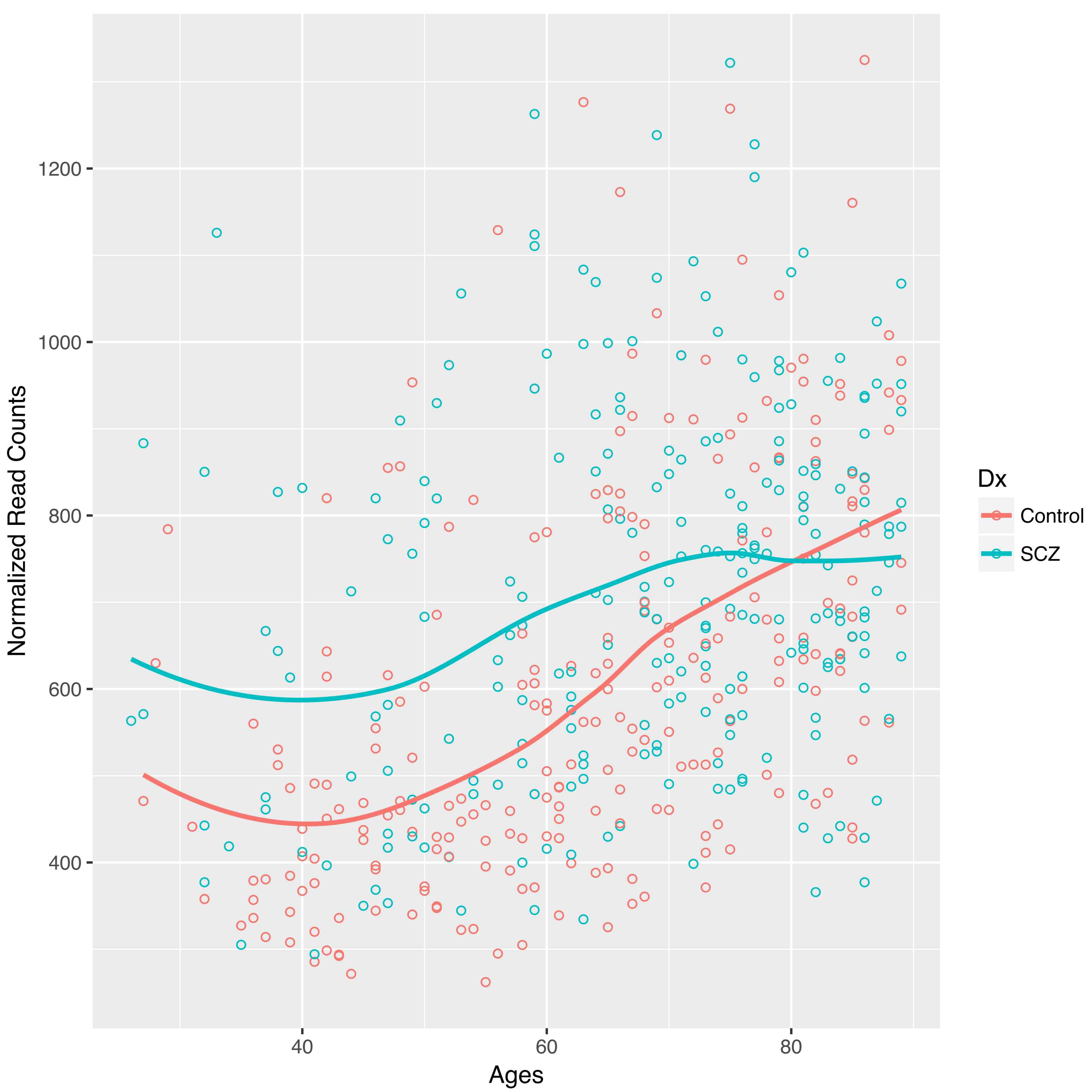

Normalized Read Counts
1200
1000
800
600
400
40
60
80
Ages
Dx
Control
SCZ

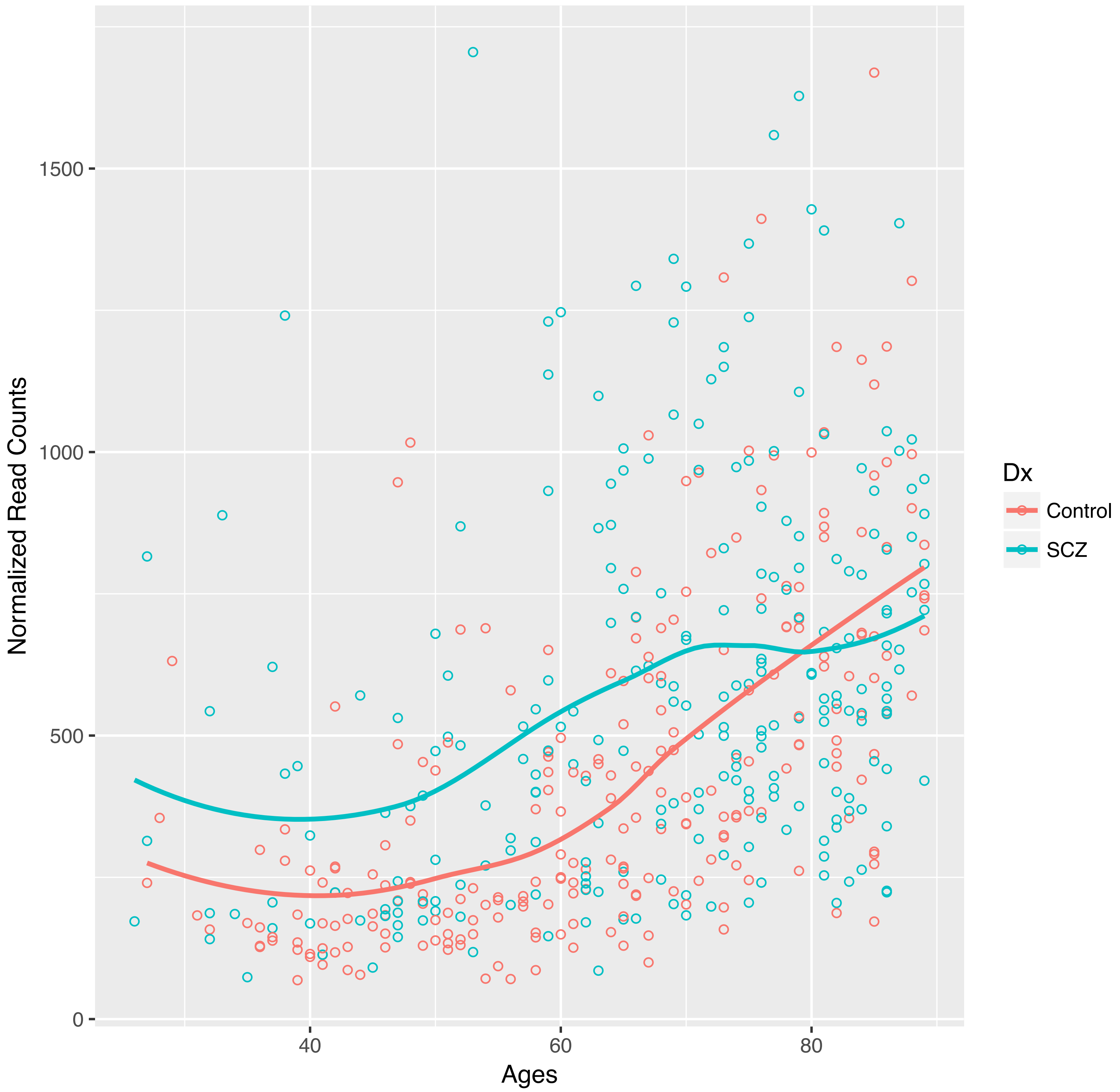

Normalized Read Counts
1500
1000
500
0
40
60
80
Ages
Dx
Control
SCZ

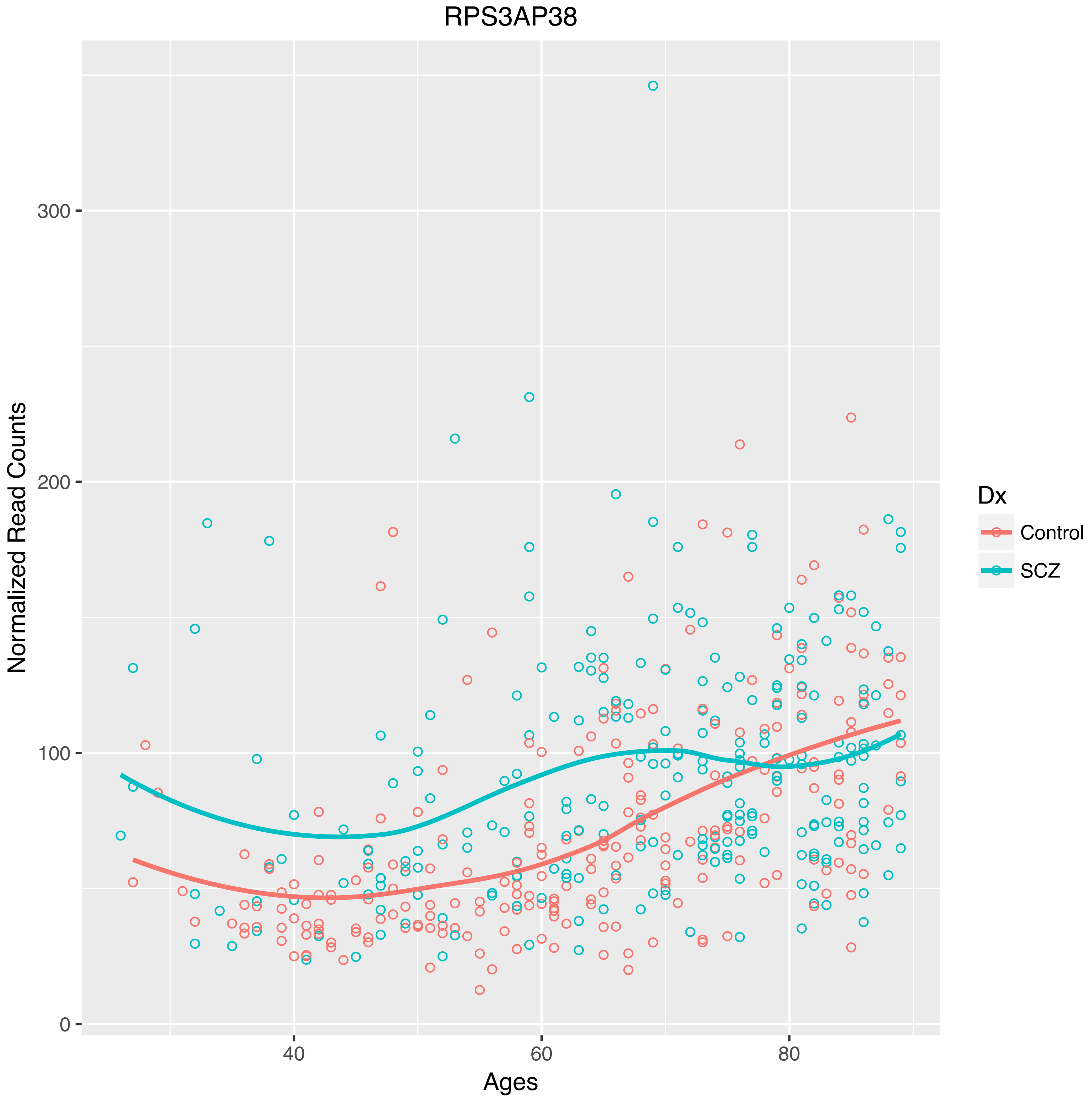

RPS3AP38
Normalized Read Counts
Ages
0
100
200
300
40
60
80
Dx
Control
SCZ

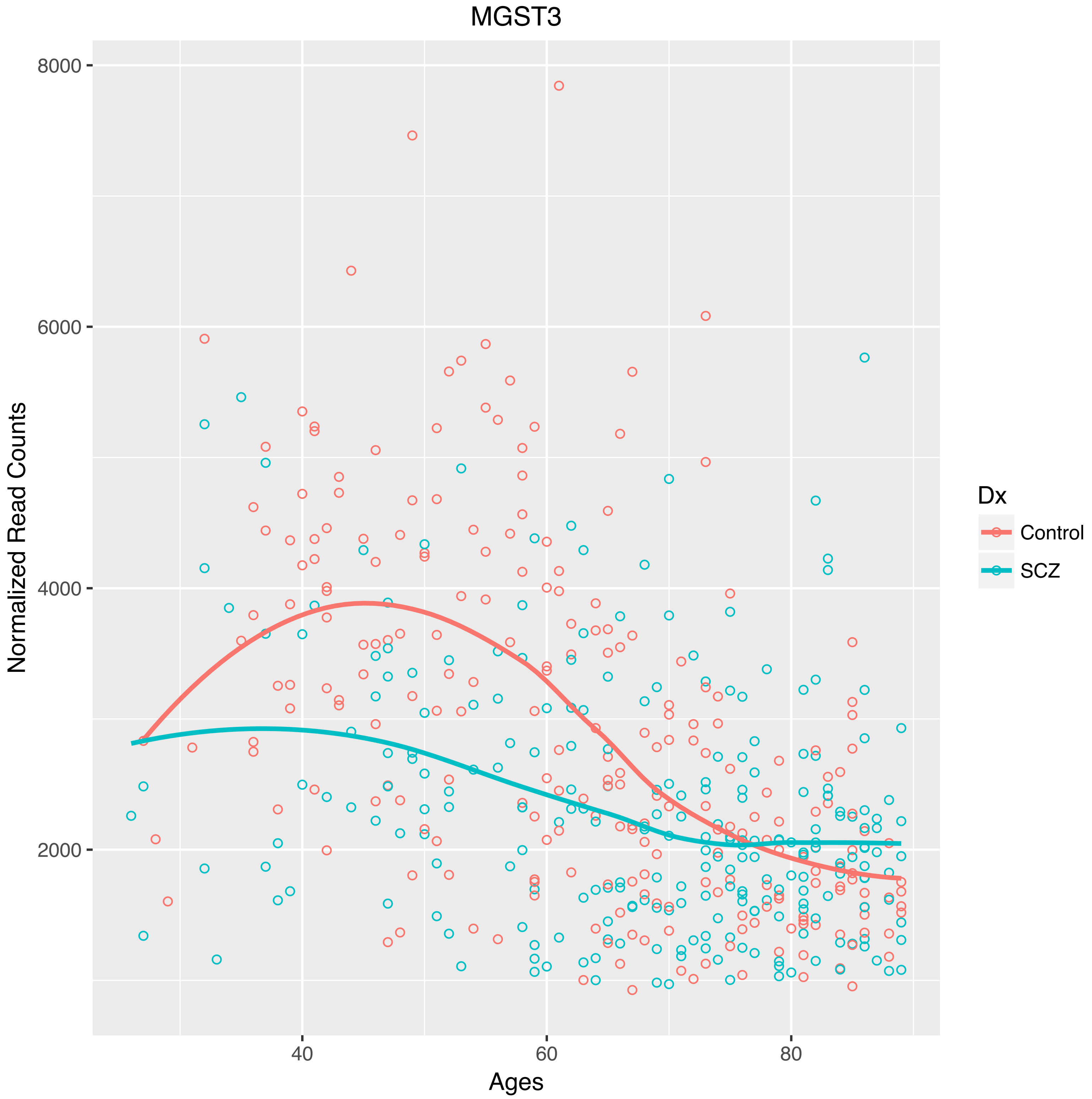

MGST3
8000
6000
4000
2000
Normalized Read Counts
40
60
80
Ages
Dx
Control
SCZ

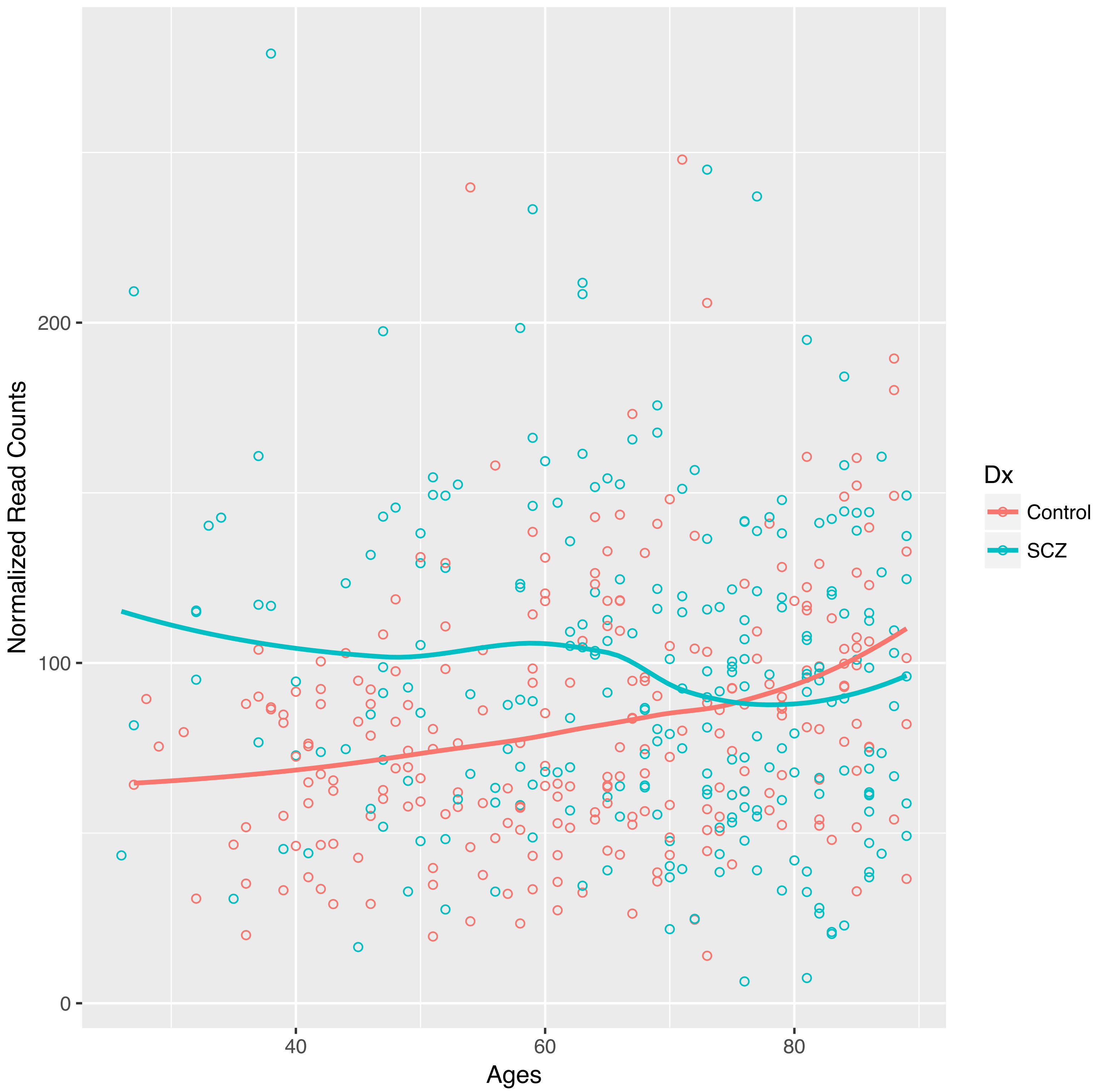

Normalized Read Counts
200
100
0
40
60
80
Ages
Dx
Control
SCZ

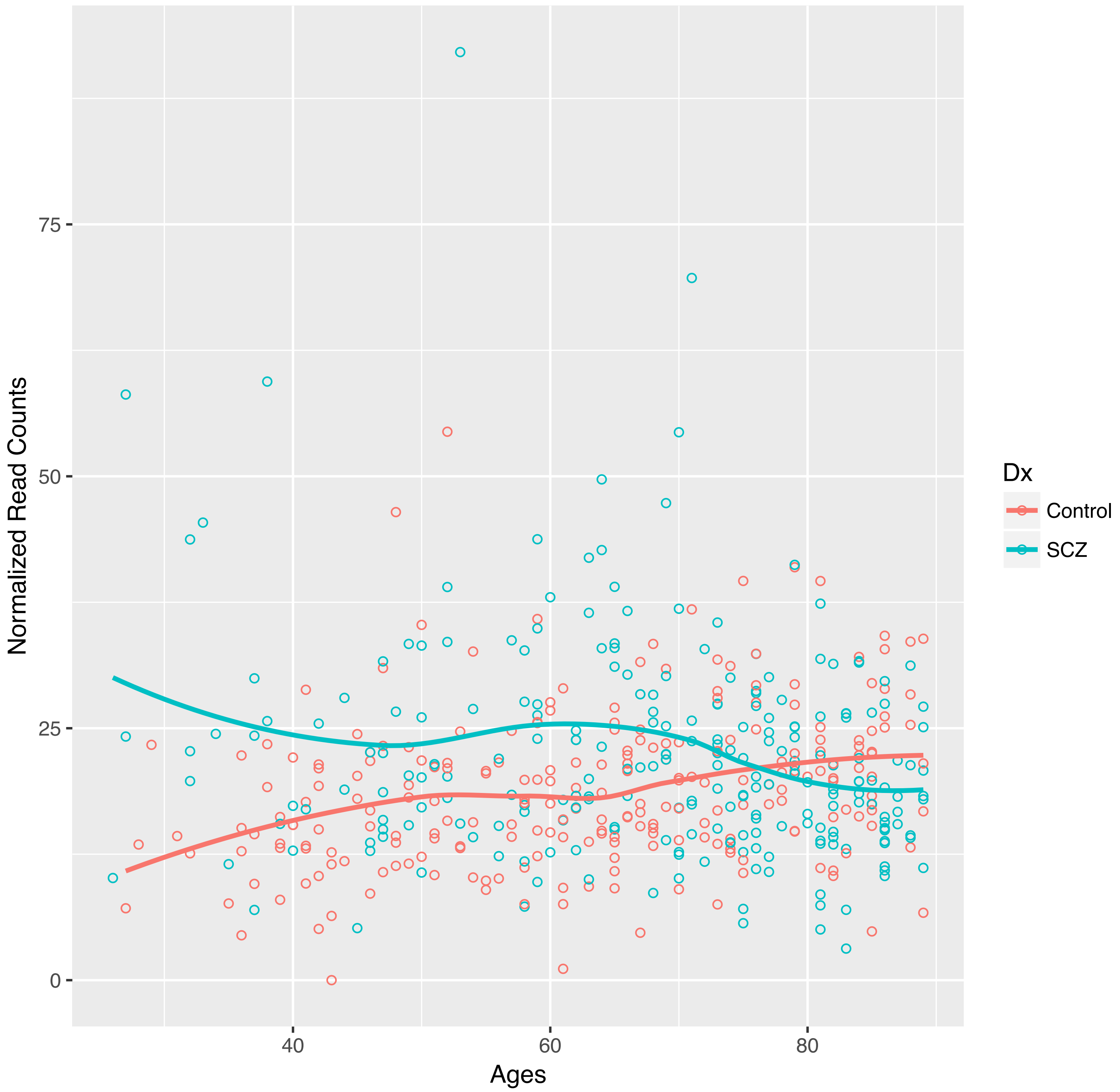

Normalized Read Counts
0
25
50
75
40
60
80
Ages
Dx
Control
SCZ

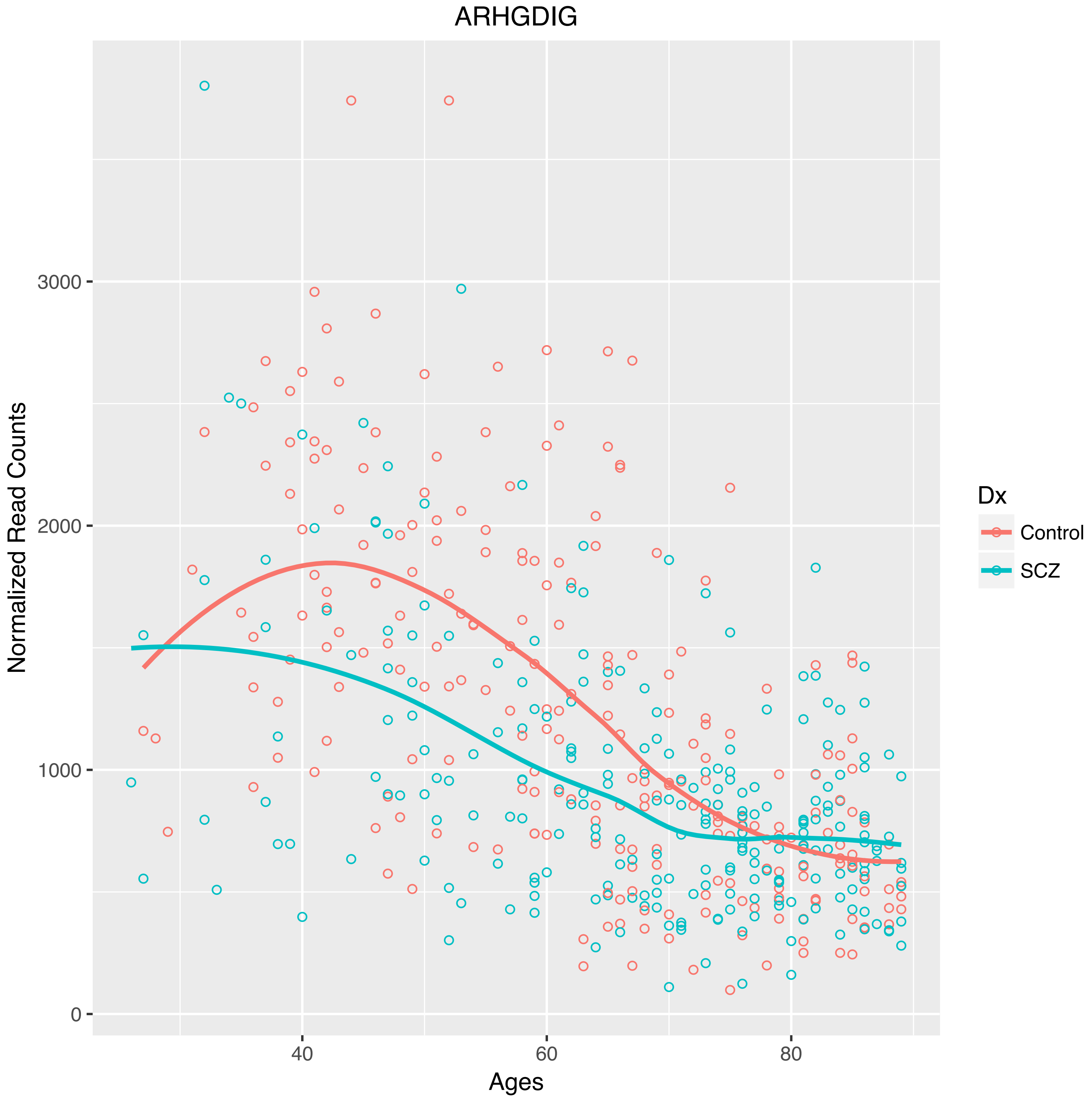
ARHGDIG
Normalized Read Counts
0
1000
2000
3000
40
60
80
Ages
Dx
Control
SCZ

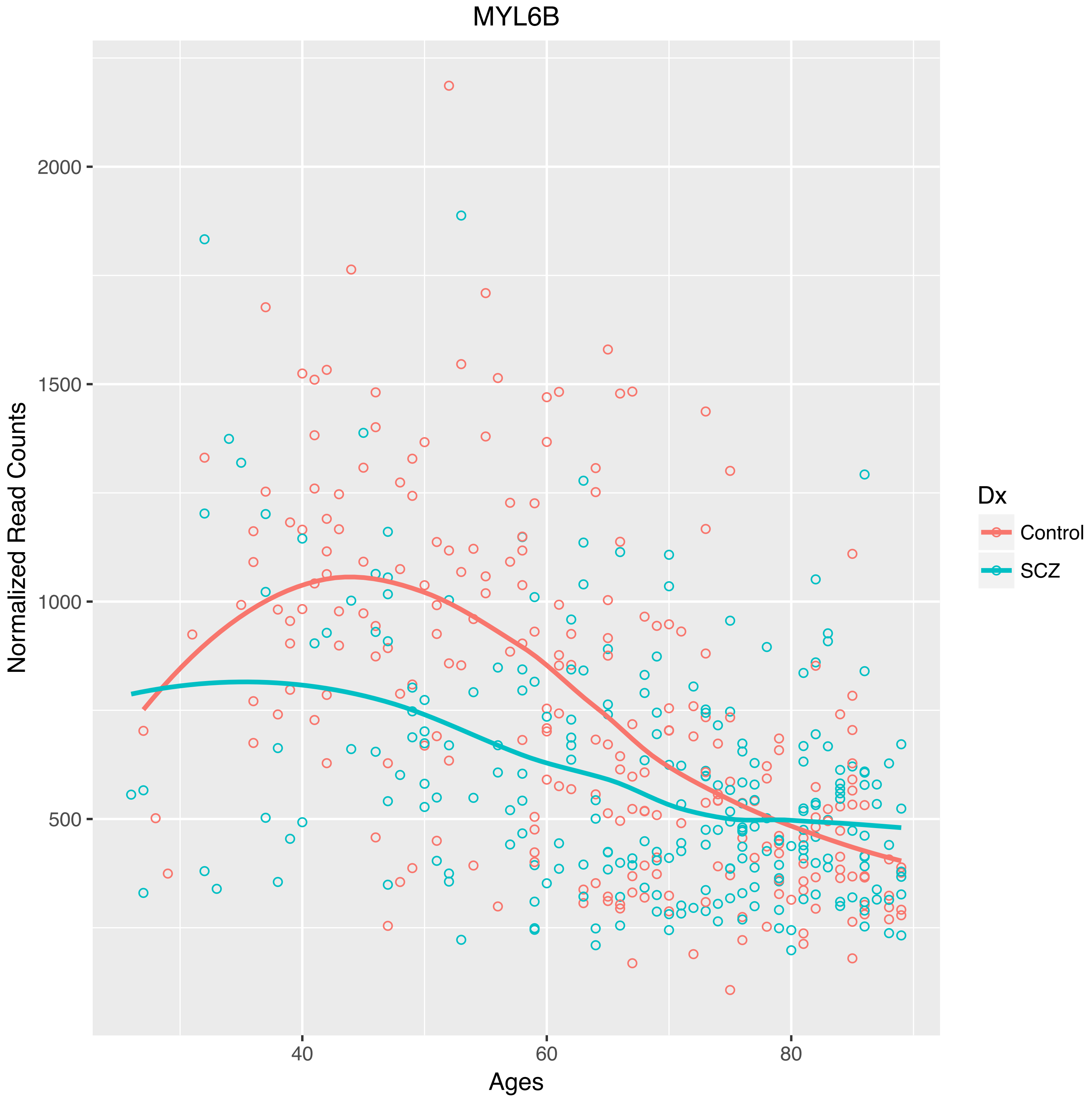

MYL6B
Normalized Read Counts
2000
1500
1000
500
40
60
80
Ages
Dx
Control
SCZ

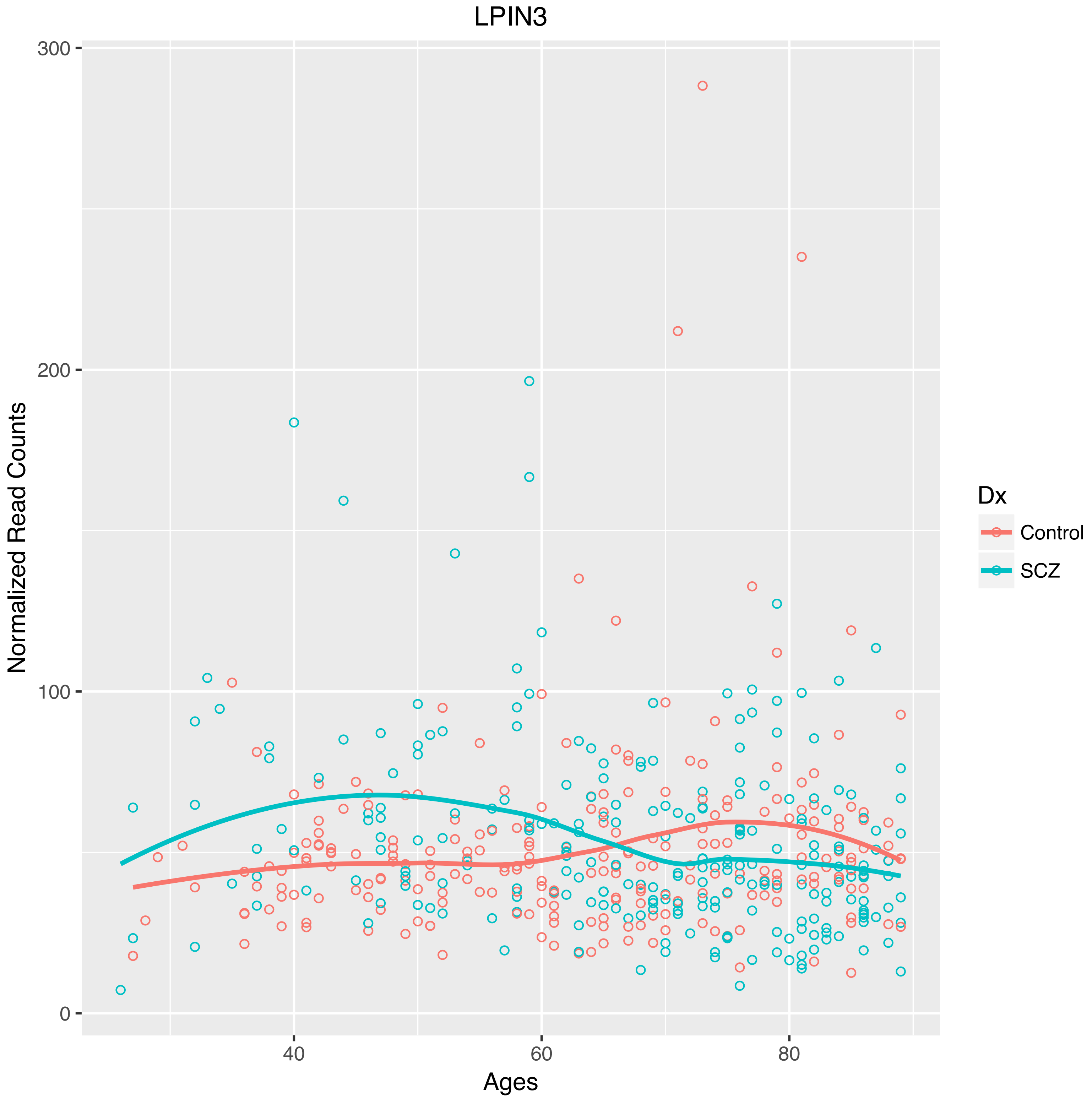

LPIN3
Normalized Read Counts
Ages
0
100
200
300
40
60
80
Dx
Control
SCZ

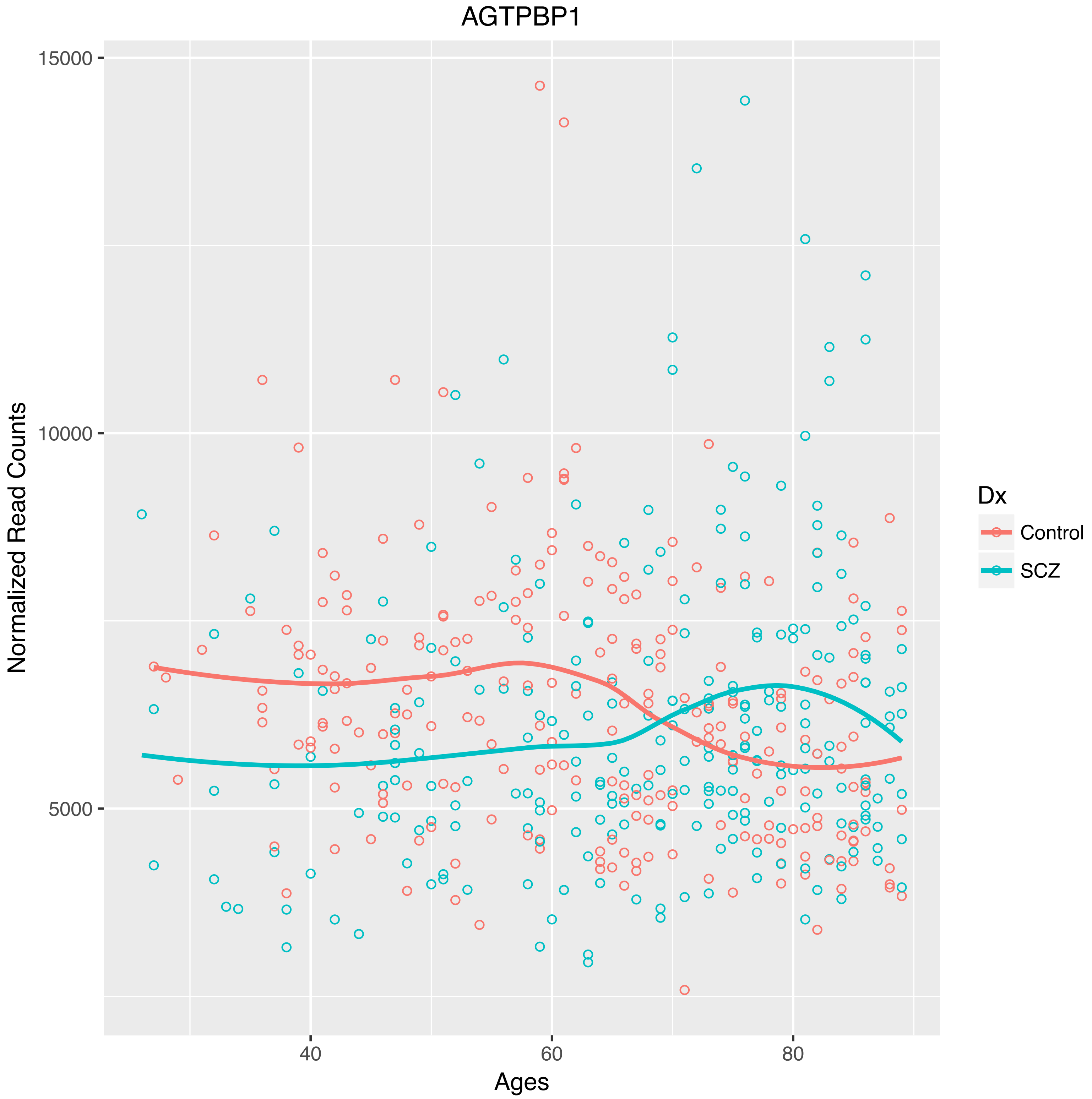

AGTPBP1
15000
10000
5000
Normalized Read Counts
40
60
80
Ages
Dx
Control
SCZ

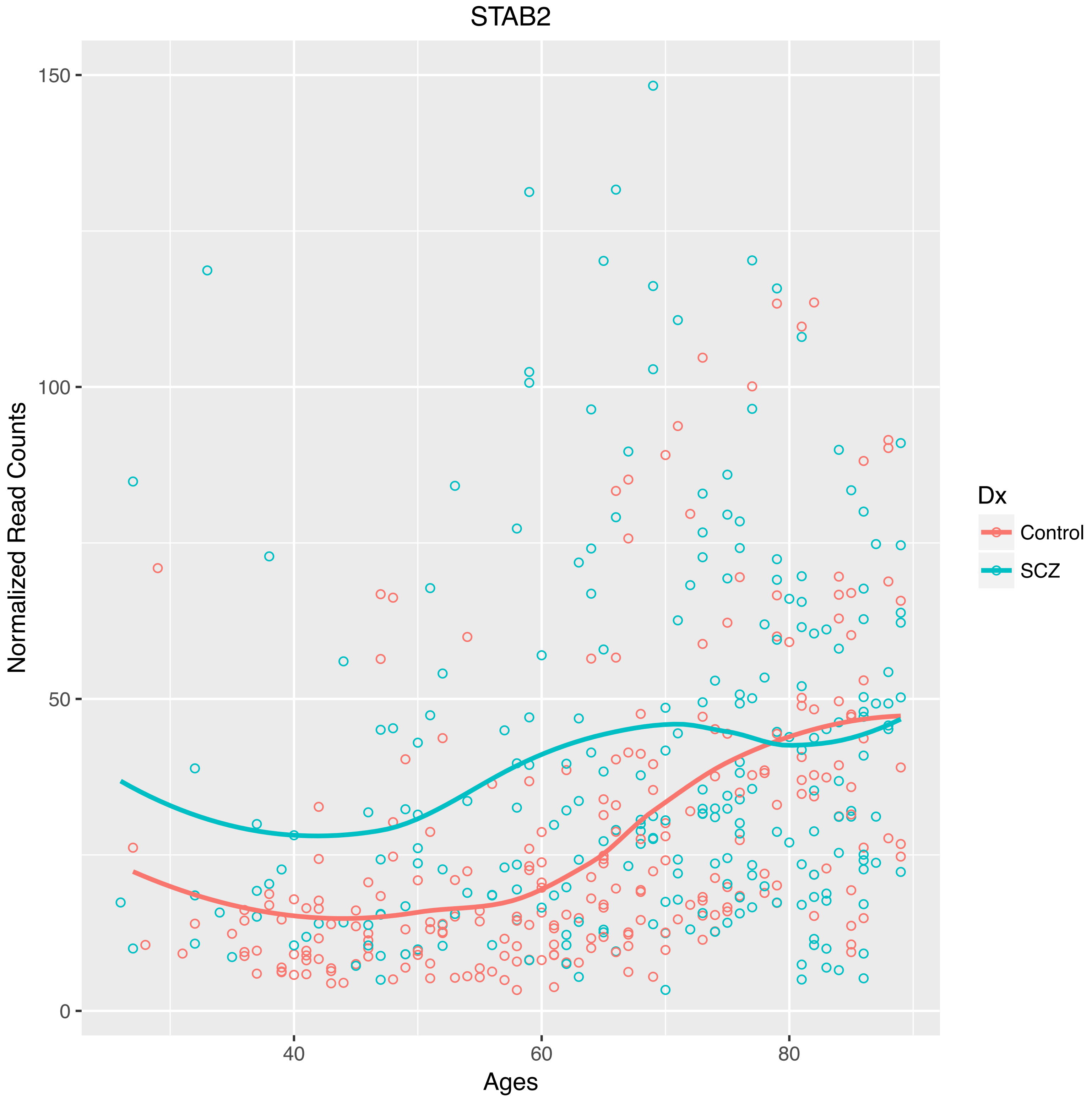

STAB2
Normalized Read Counts
150
100
50
0
40
60
80
Ages
Dx
Control
SCZ

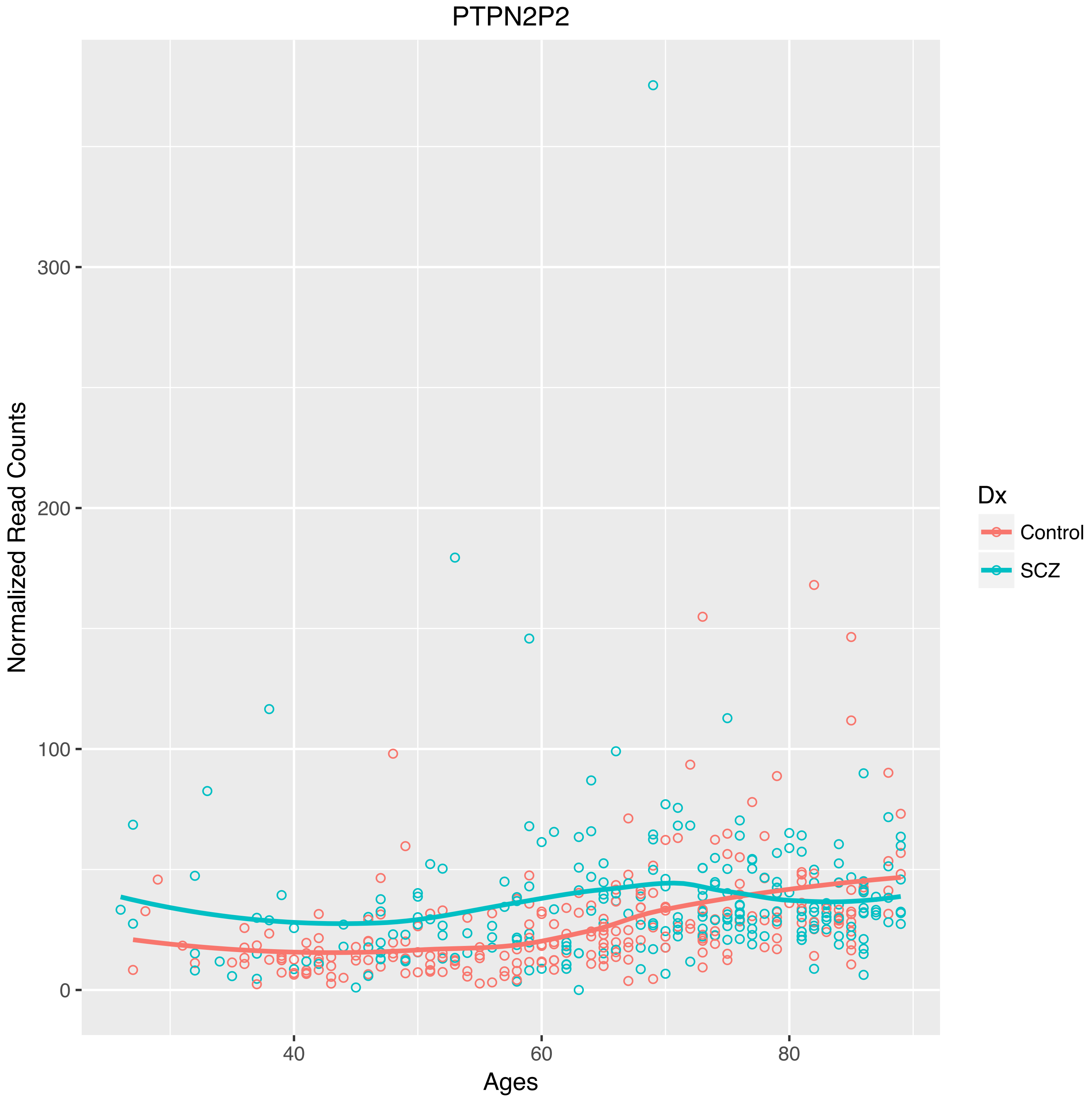

PTPN2P2
Normalized Read Counts
0
100
200
300
40
60
80
Ages
Dx
Control
SCZ

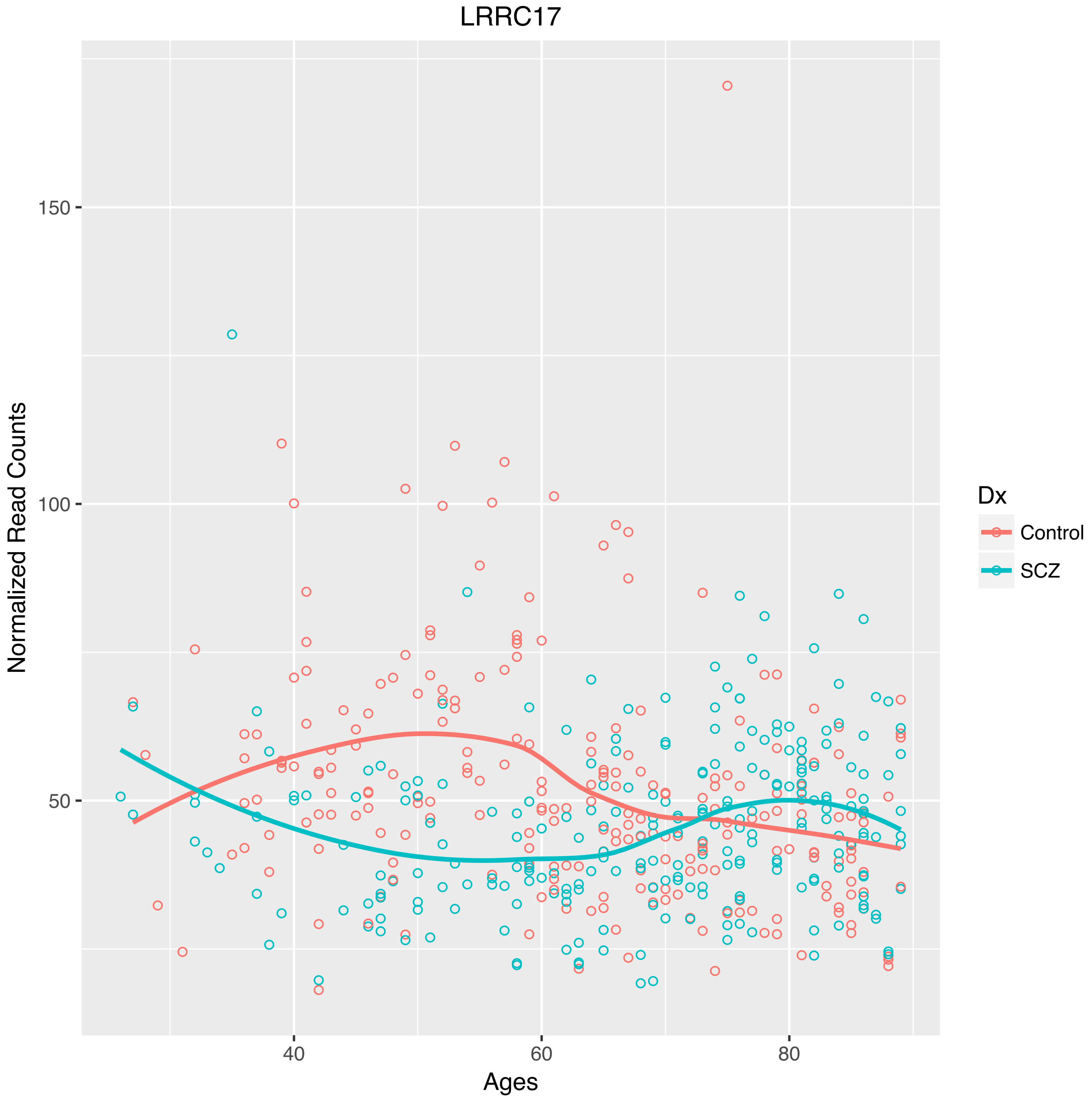
LRRC17
Normalized Read Counts
150
100
50
40
60
80
Ages
Dx
Control
SCZ

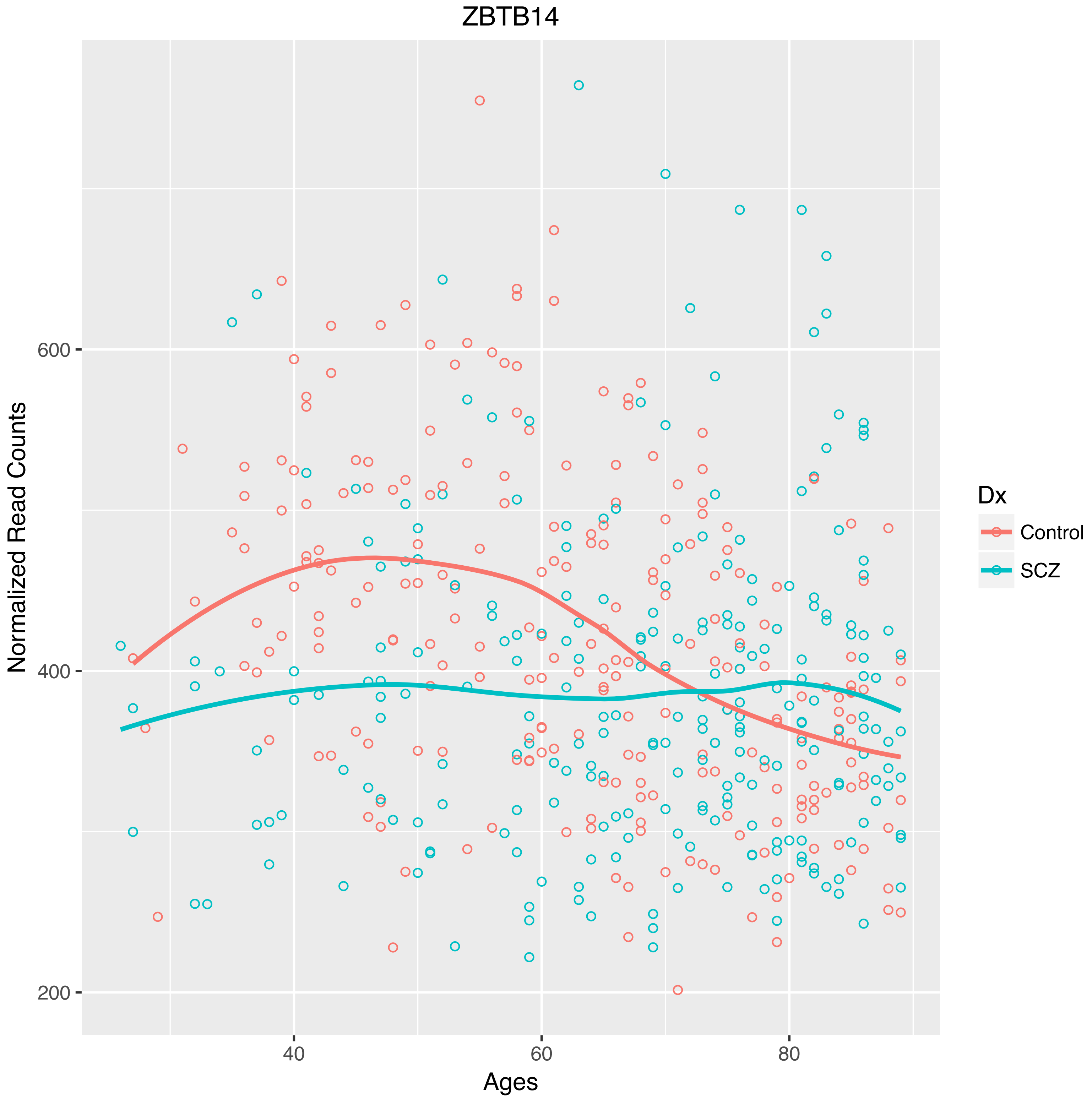
ZBTB14
Normalized Read Counts
600
400
200
Ages
40
60
80
Dx
Control
SCZ

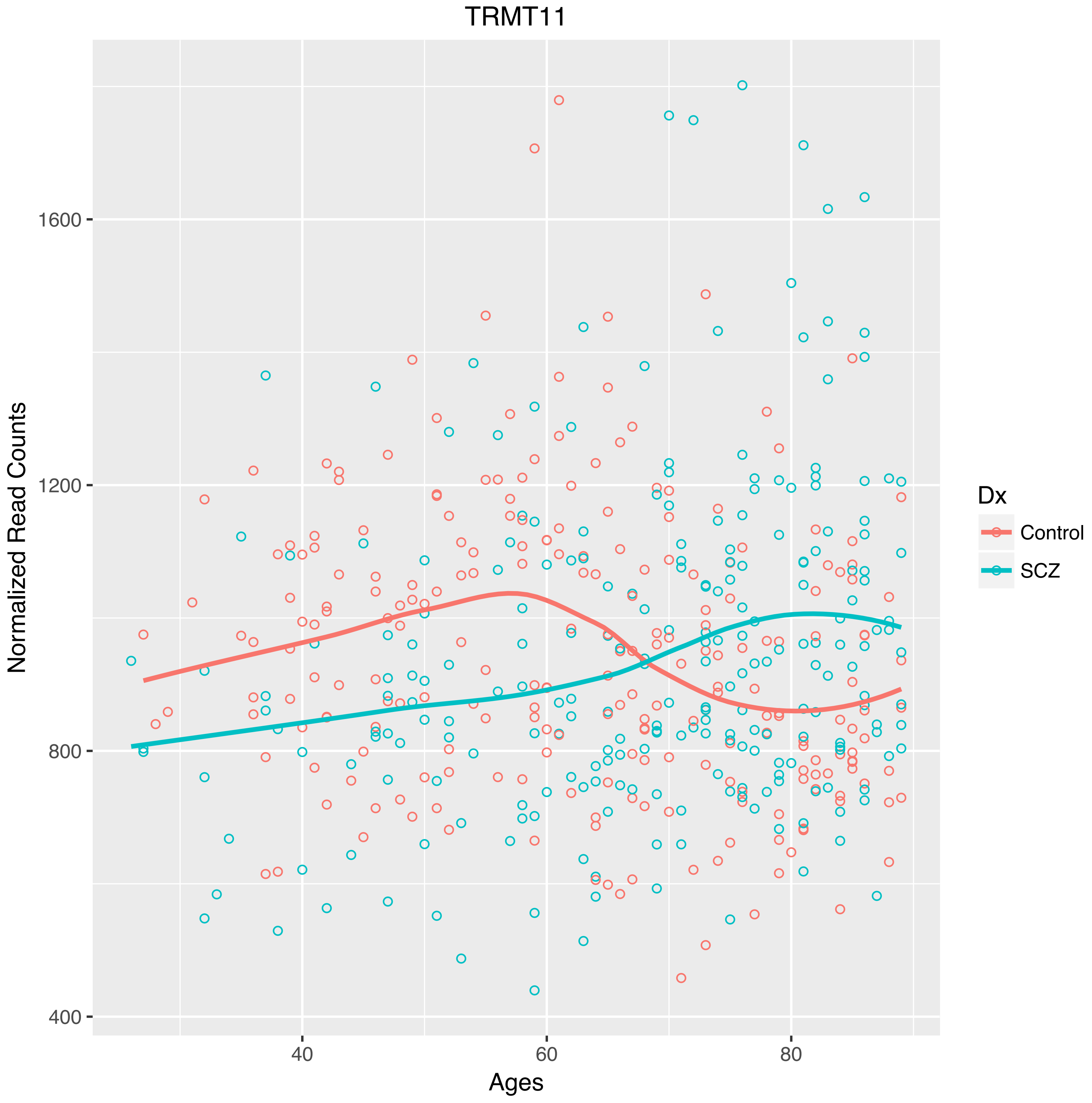

TRMT11
Normalized Read Counts
1600
1200
800
400
40
60
80
Ages
Dx
Control
SCZ

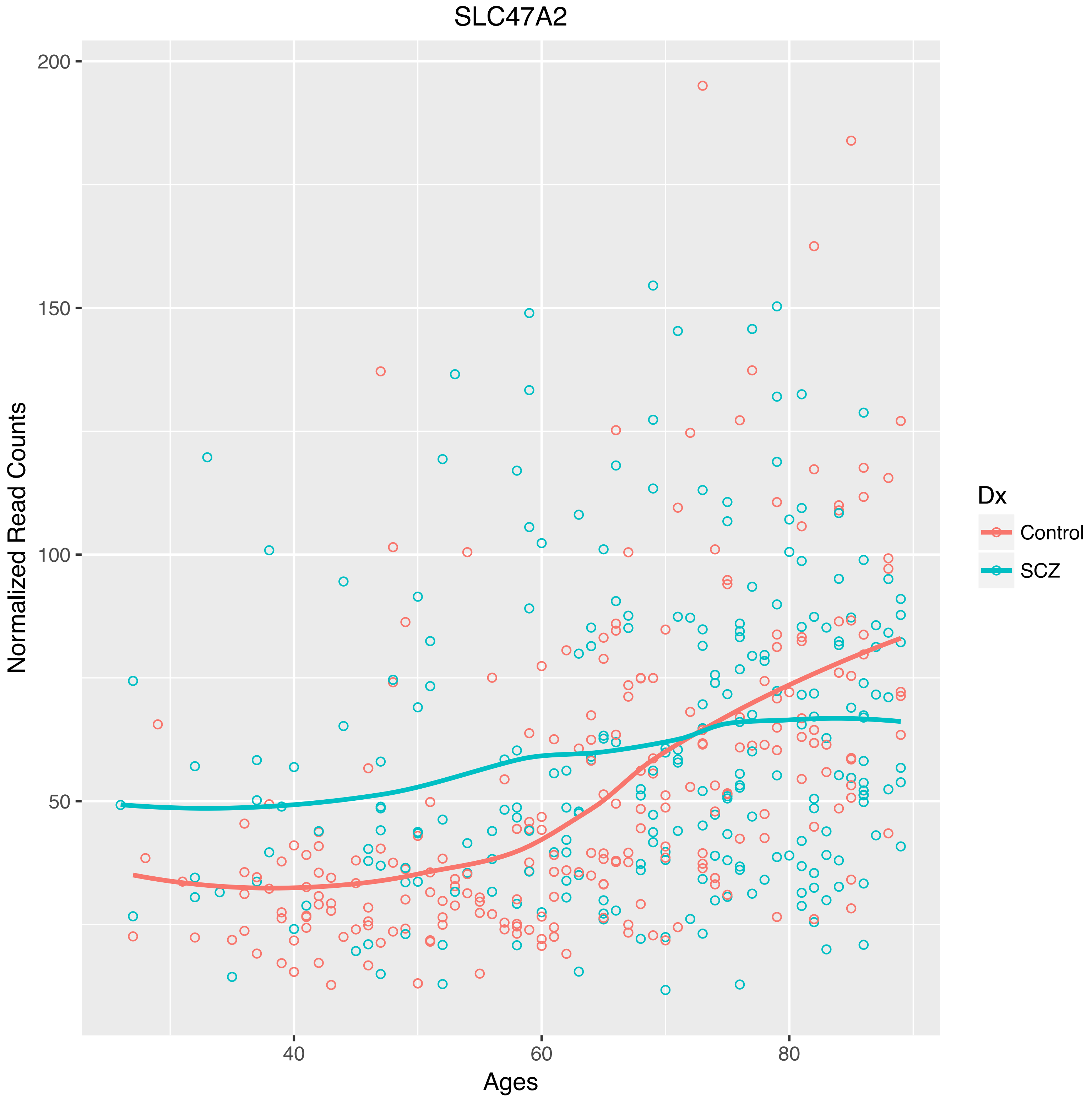

SLC47A2
Normalized Read Counts
200
150
100
50
40
60
80
Ages
Dx
Control
SCZ

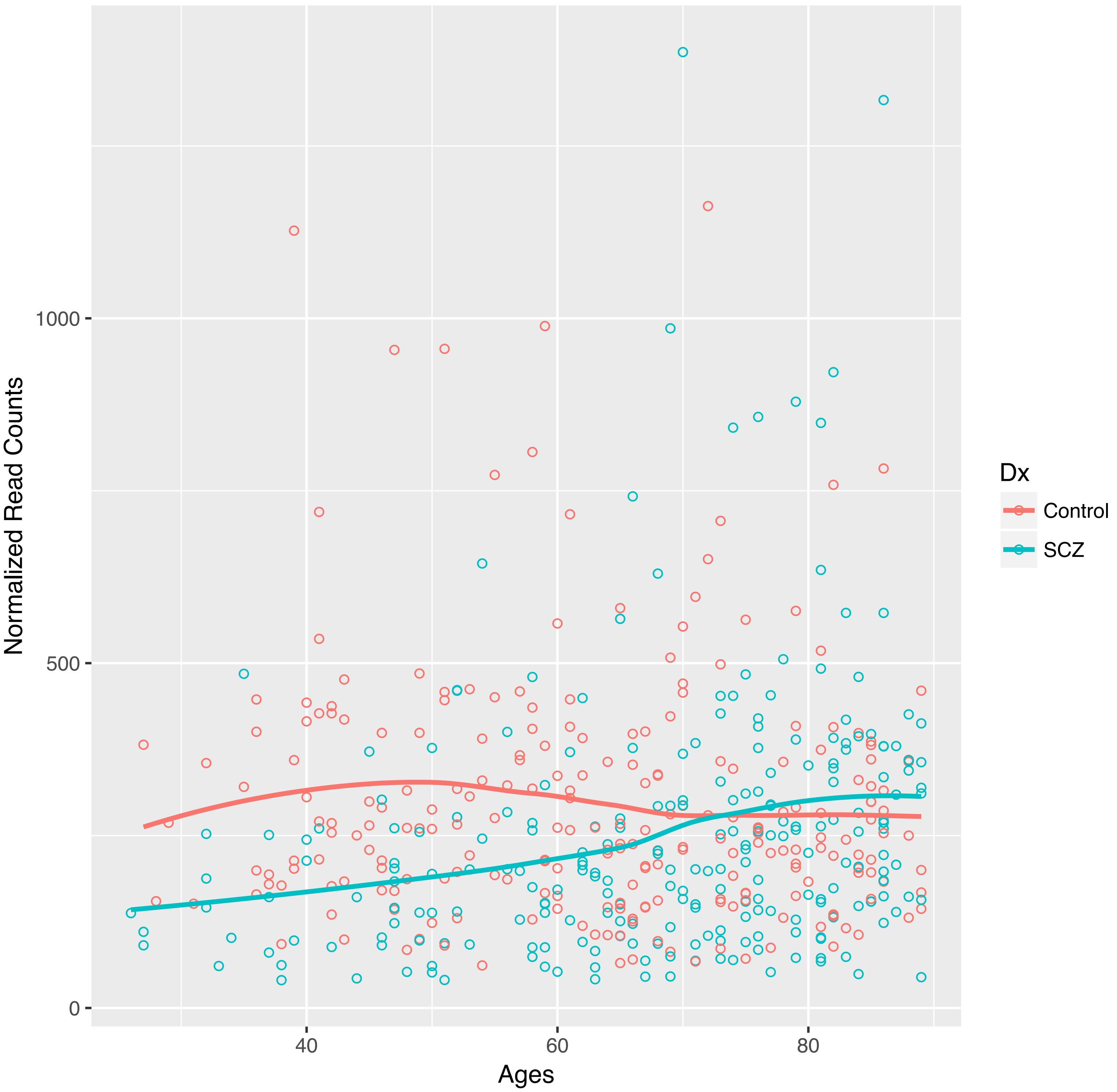
Normalized Read Counts
0
500
1000
40
60
80
Ages
Dx
Control
SCZ

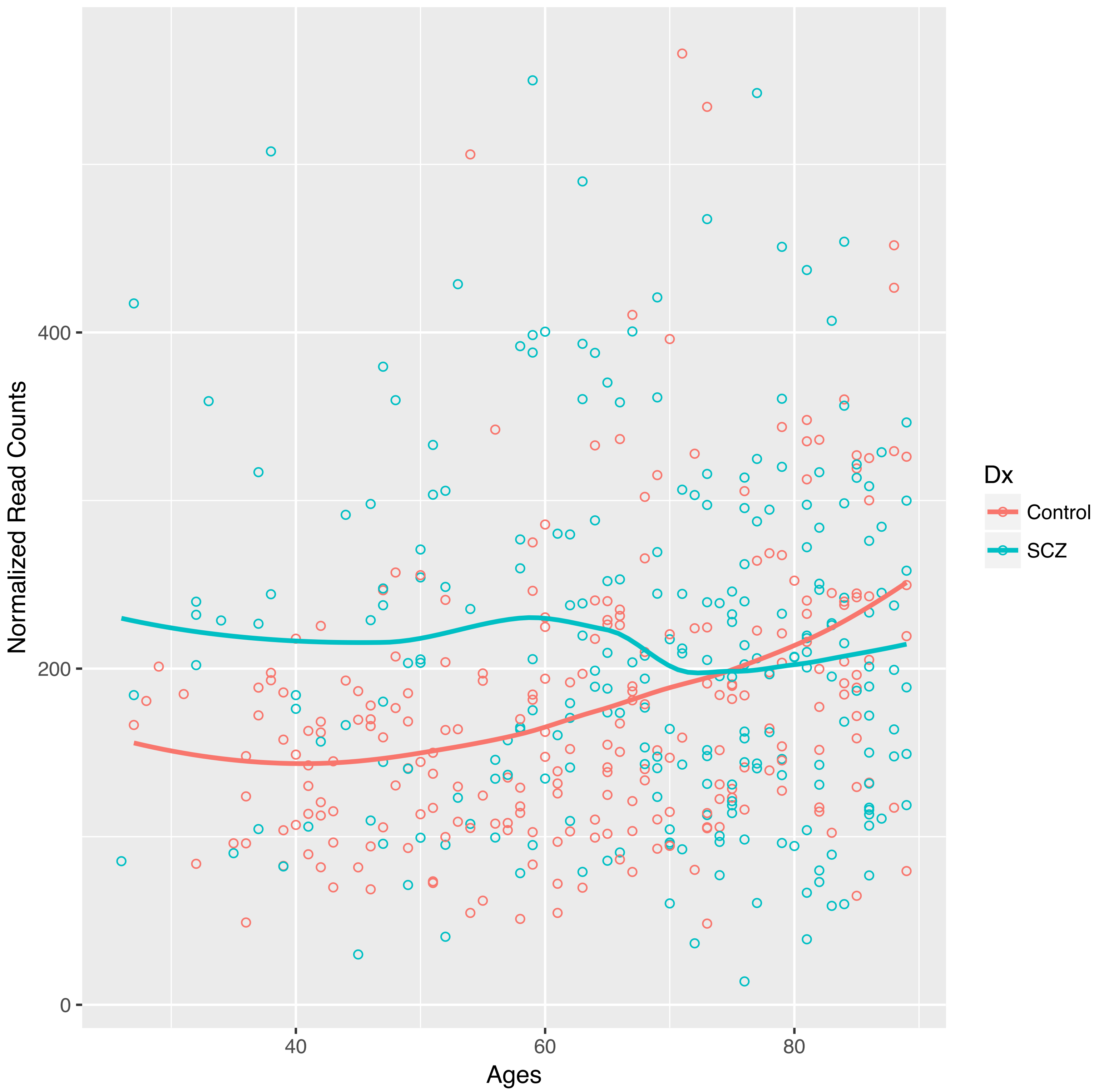

Normalized Read Counts
0
200
400
40
60
80
Ages
Dx
Control
SCZ

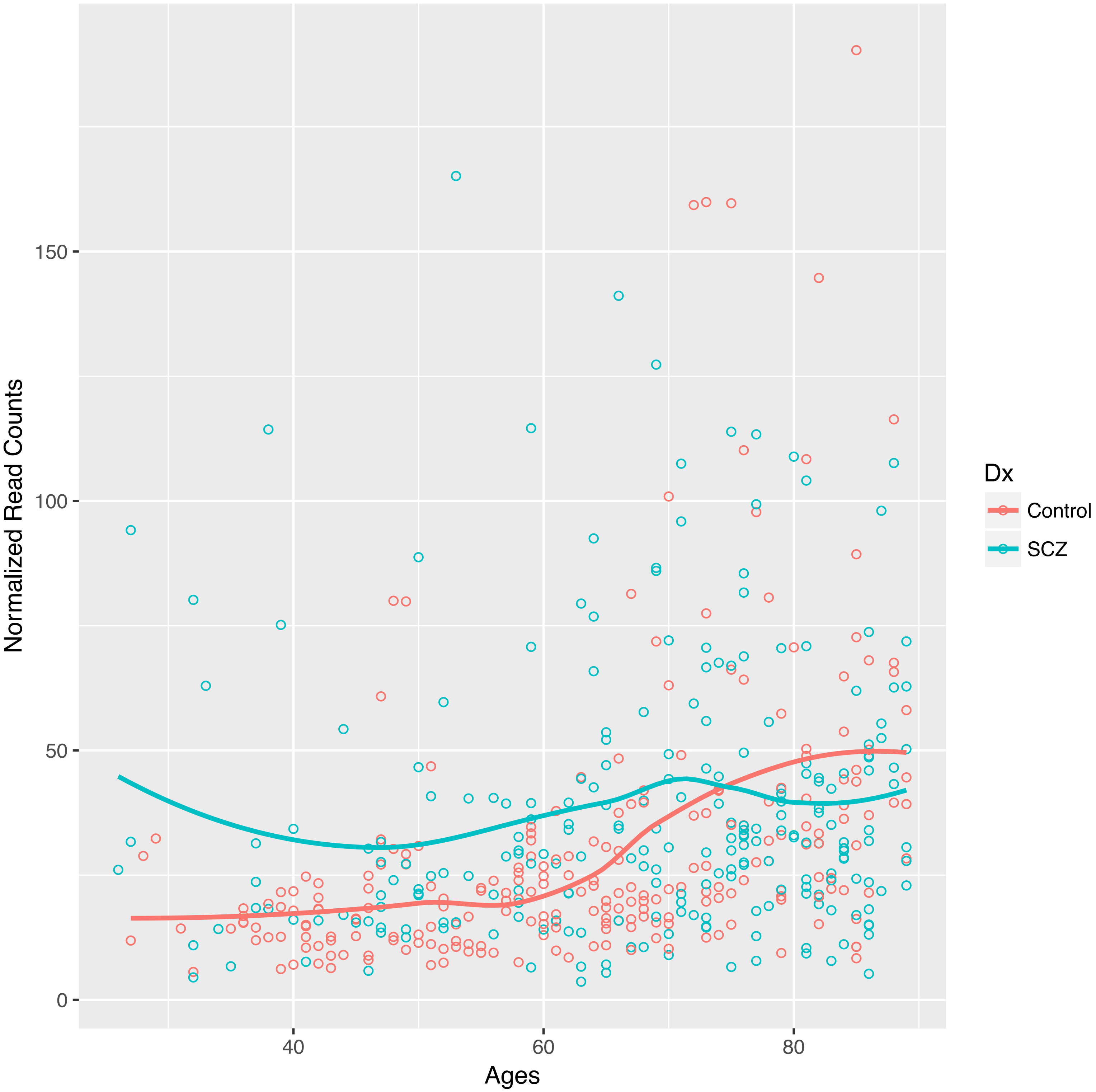

Normalized Read Counts
0
50
100
150
40
60
80
Ages
Dx
Control
SCZ

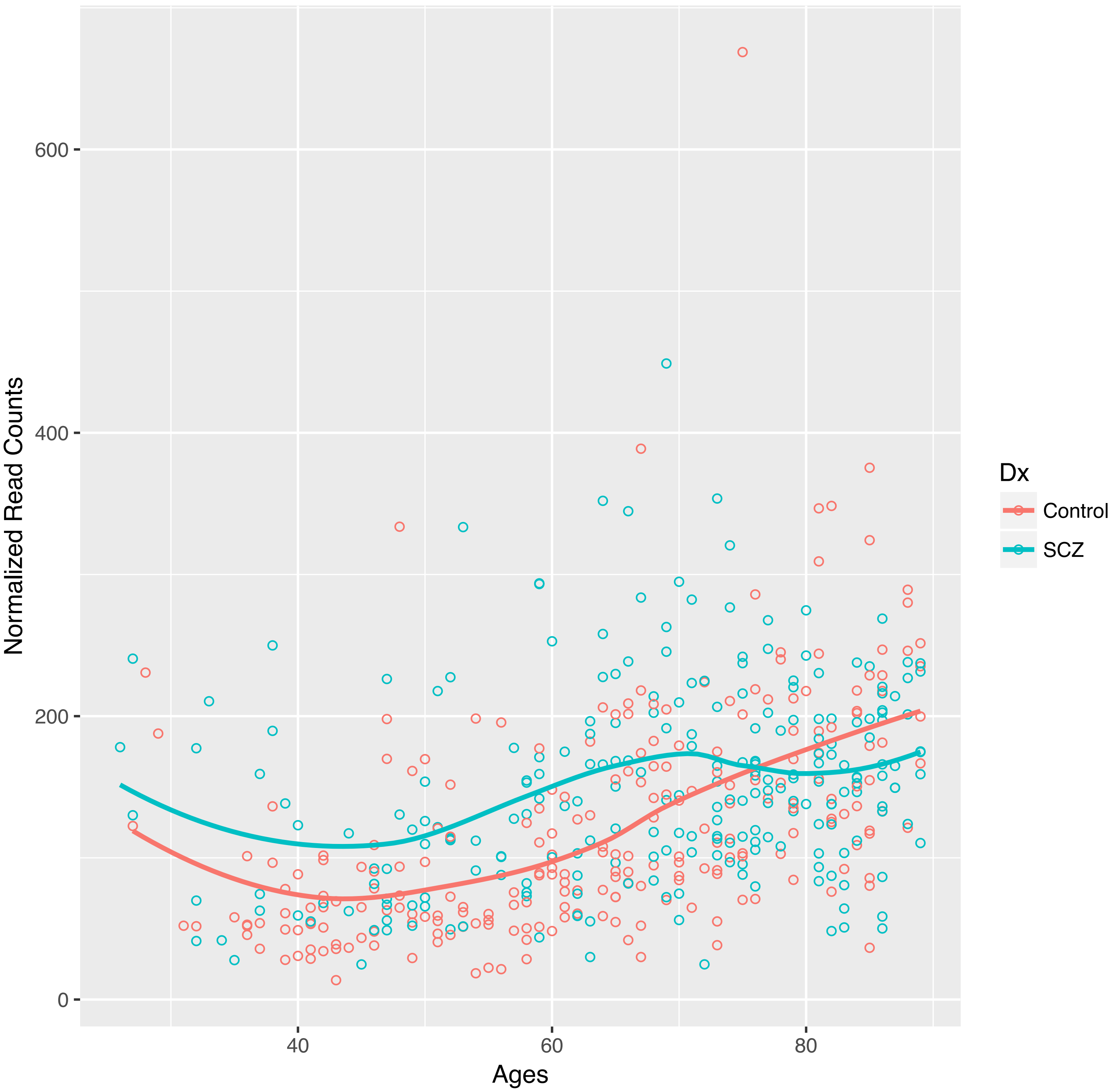

600
400
200
0
40
60
80
Ages
Normalized Read Counts
Dx
Control
SCZ

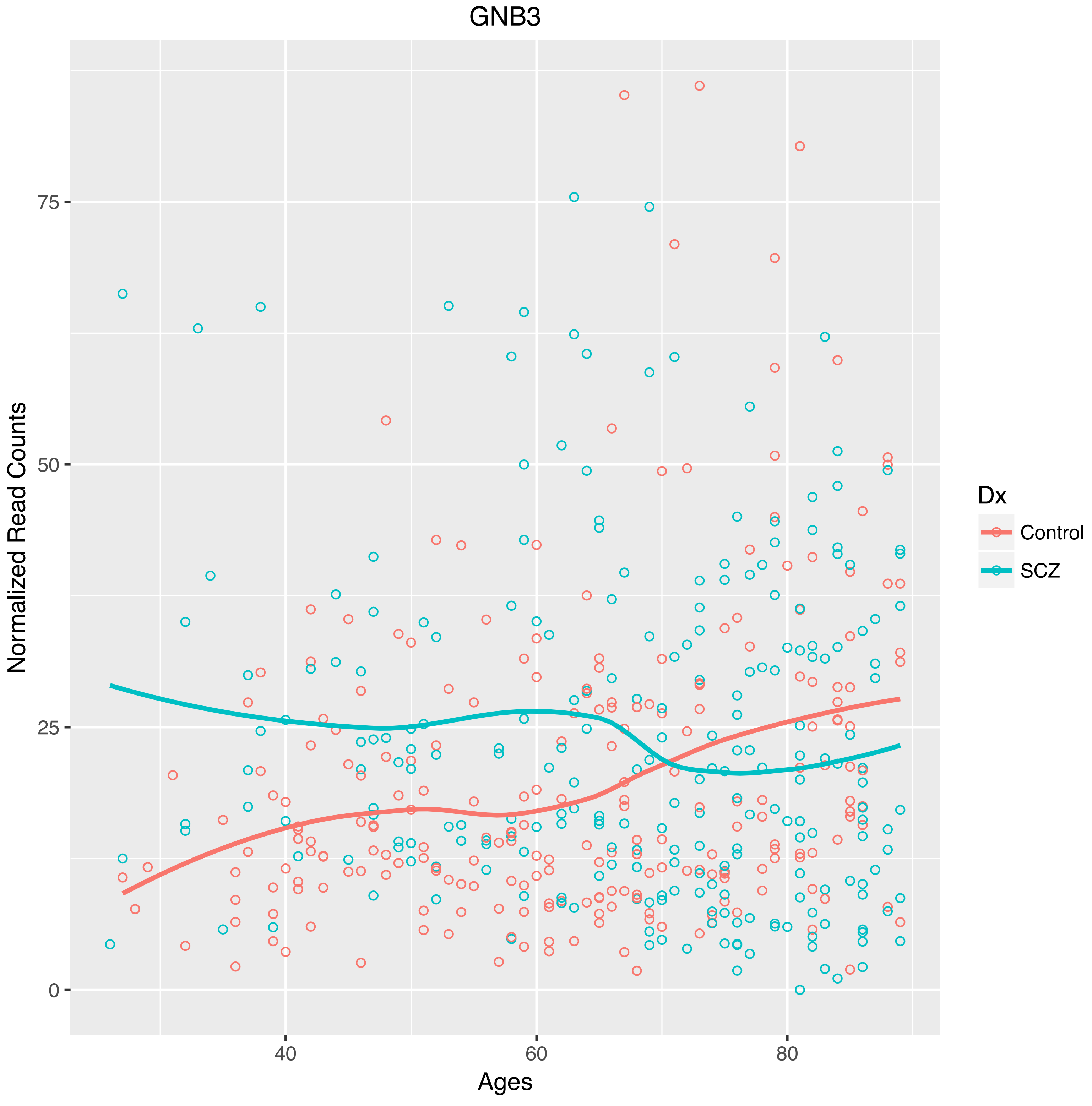
GNB3
Normalized Read Counts
75
50
25
0
40
60
80
Ages
Dx
Control
SCZ

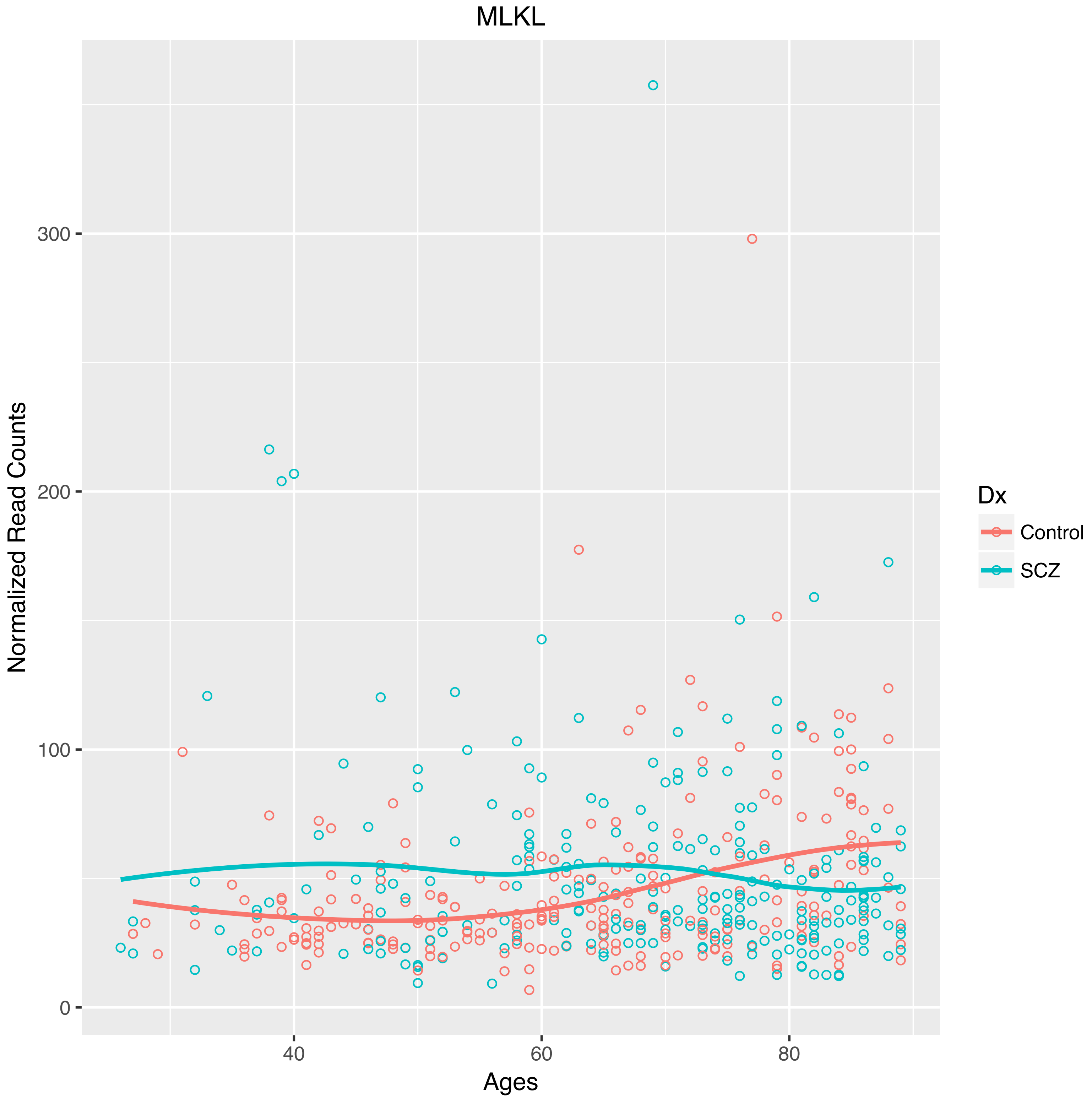

MLKL
Normalized Read Counts
300
200
100
0
40
60
80
Ages
Dx
Control
SCZ

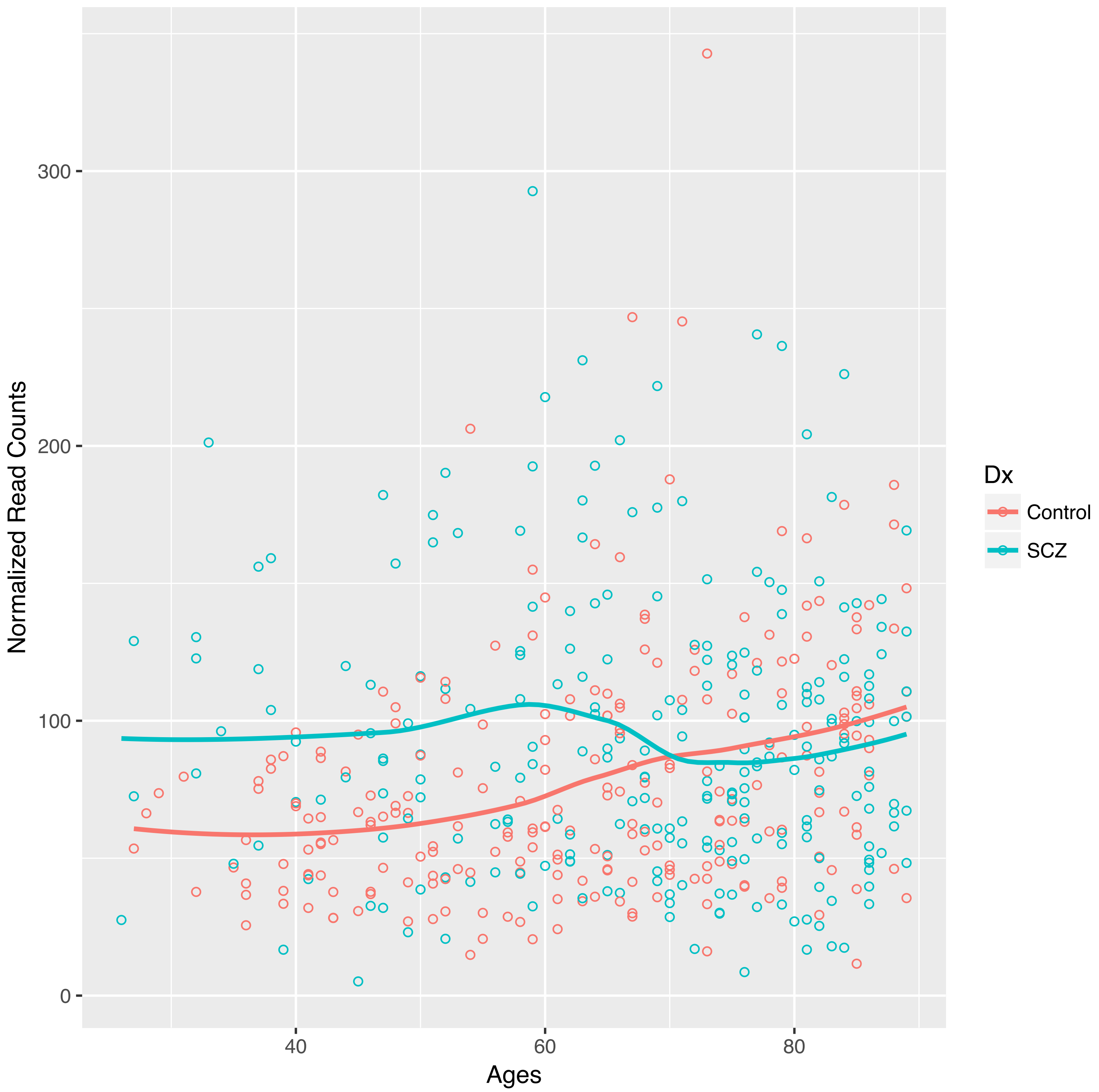

Normalized Read Counts
300
200
100
0
40
60
80
Ages
Dx
Control
SCZ

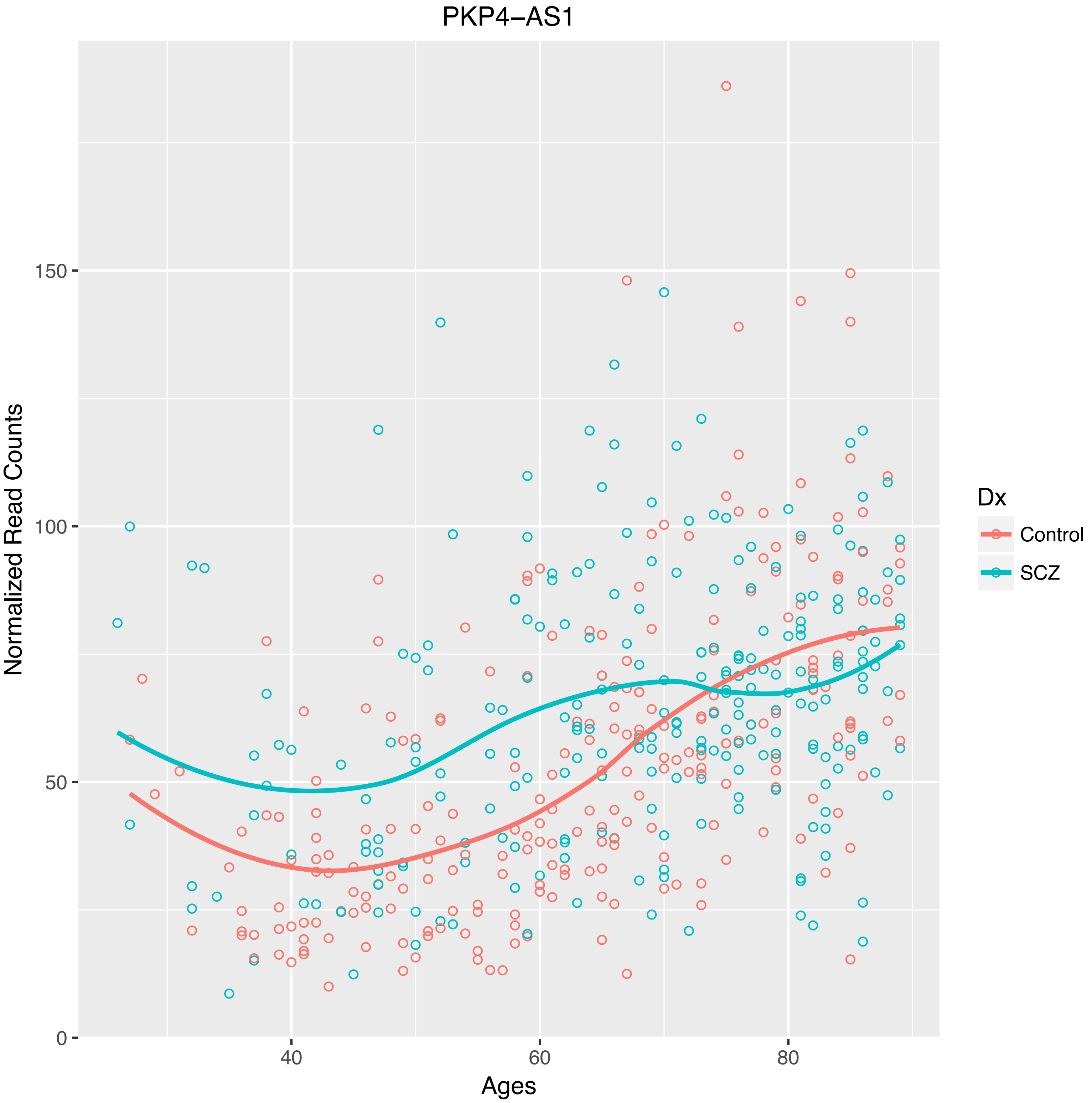

PKP4−AS1
Normalized Read Counts
150
100
50
0
40
60
80
Ages
Dx
Control
SCZ

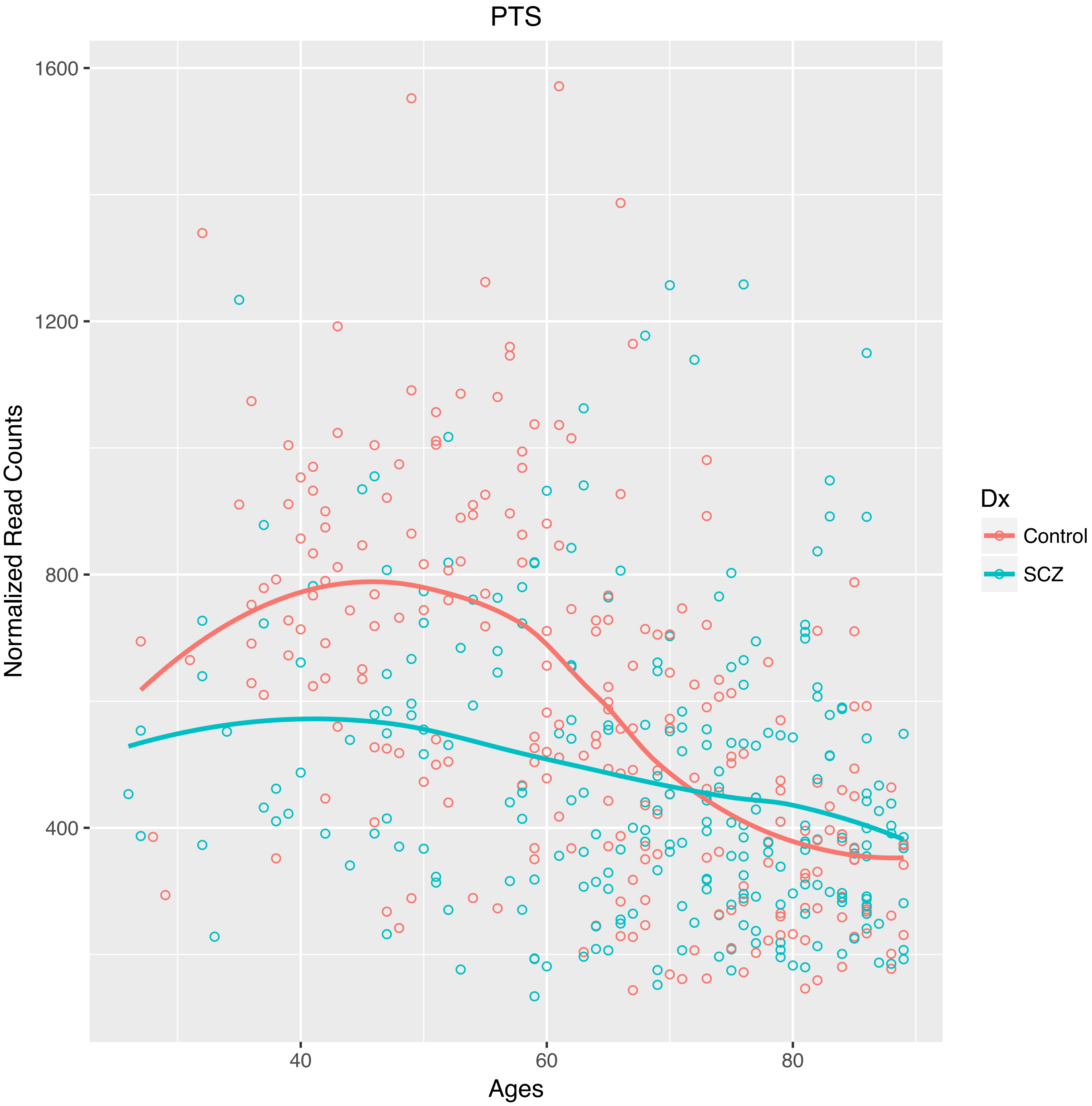
PTS
Normalized Read Counts
1600
1200
800
400
40
60
80
Ages
Dx
Control
SCZ

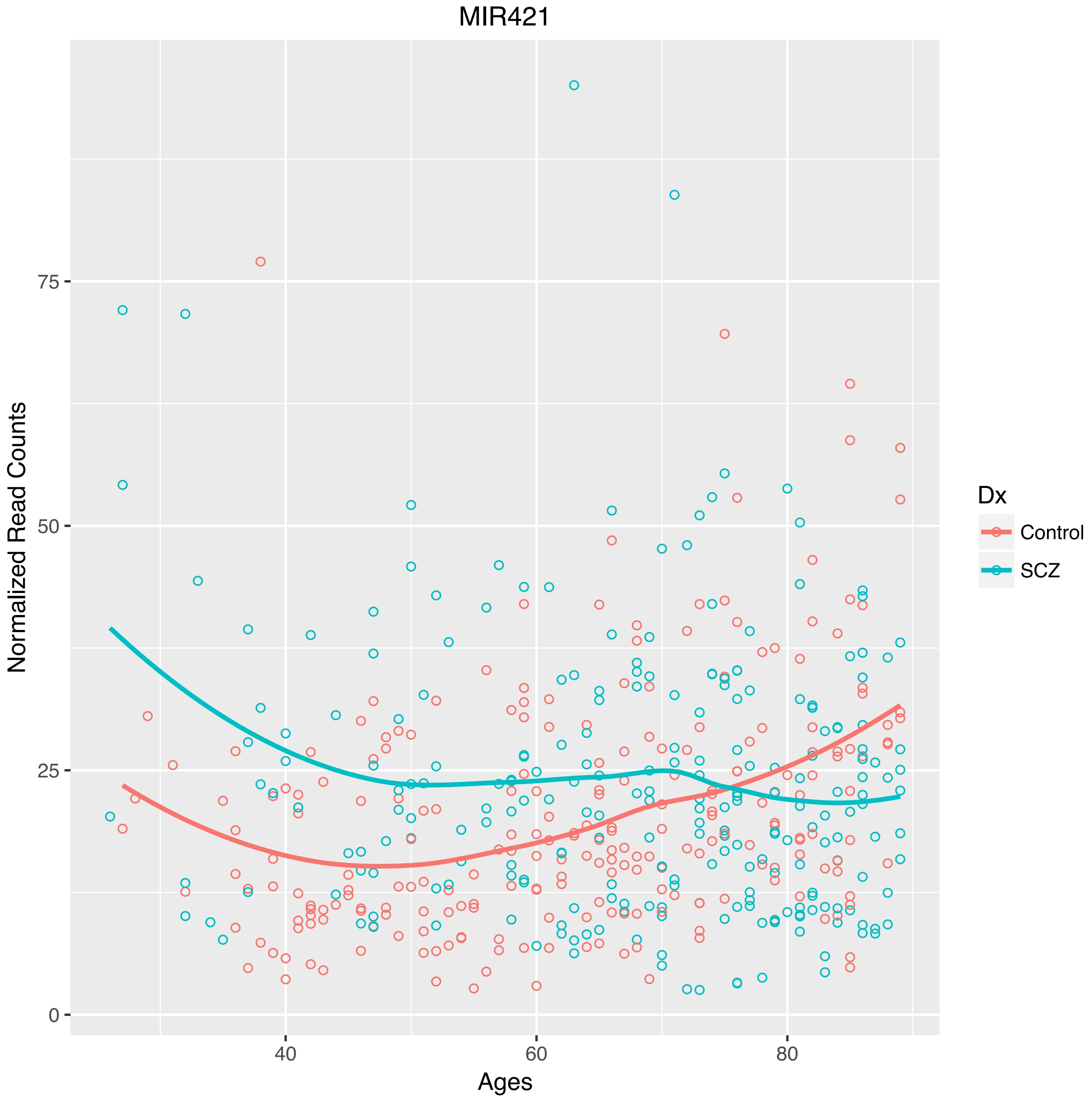

MIR421
Normalized Read Counts
0
25
50
75
40
60
80
Ages
Dx
Control
SCZ

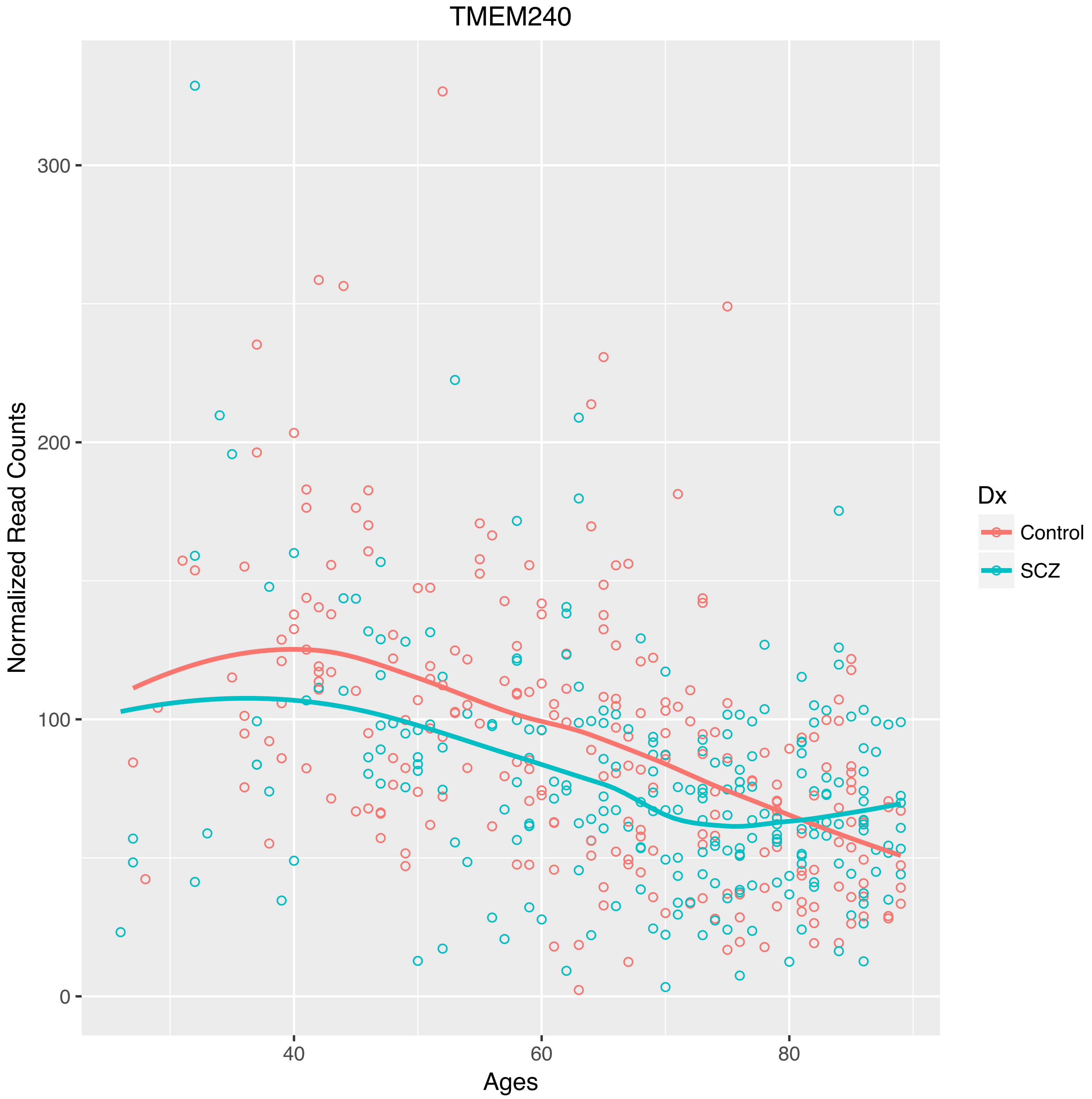
TMEM240
Normalized Read Counts
0
100
200
300
40
60
80
Ages
Dx
Control
SCZ

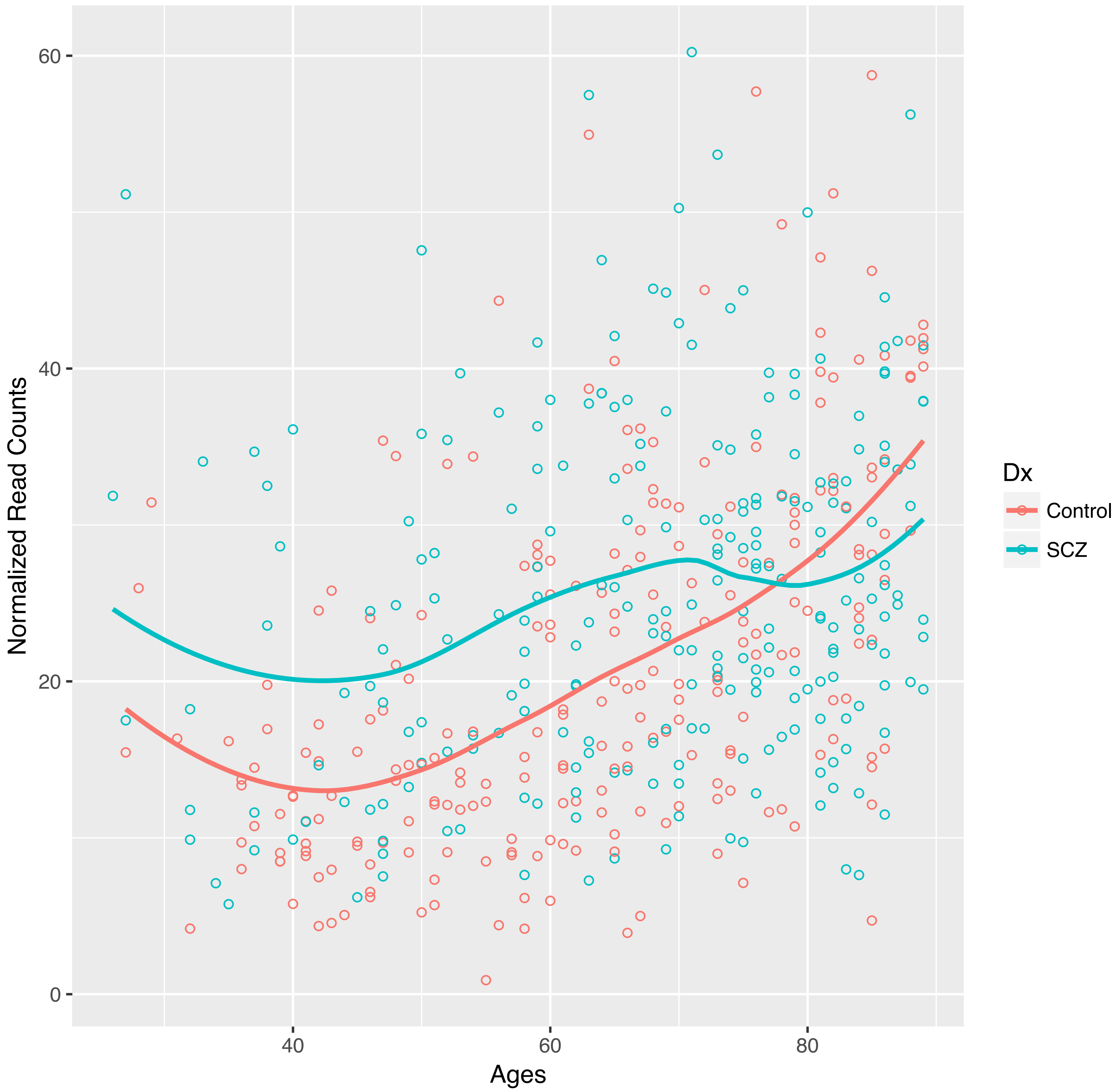

Normalized Read Counts
0
20
40
60
40
60
80
Ages
Dx
Control
SCZ

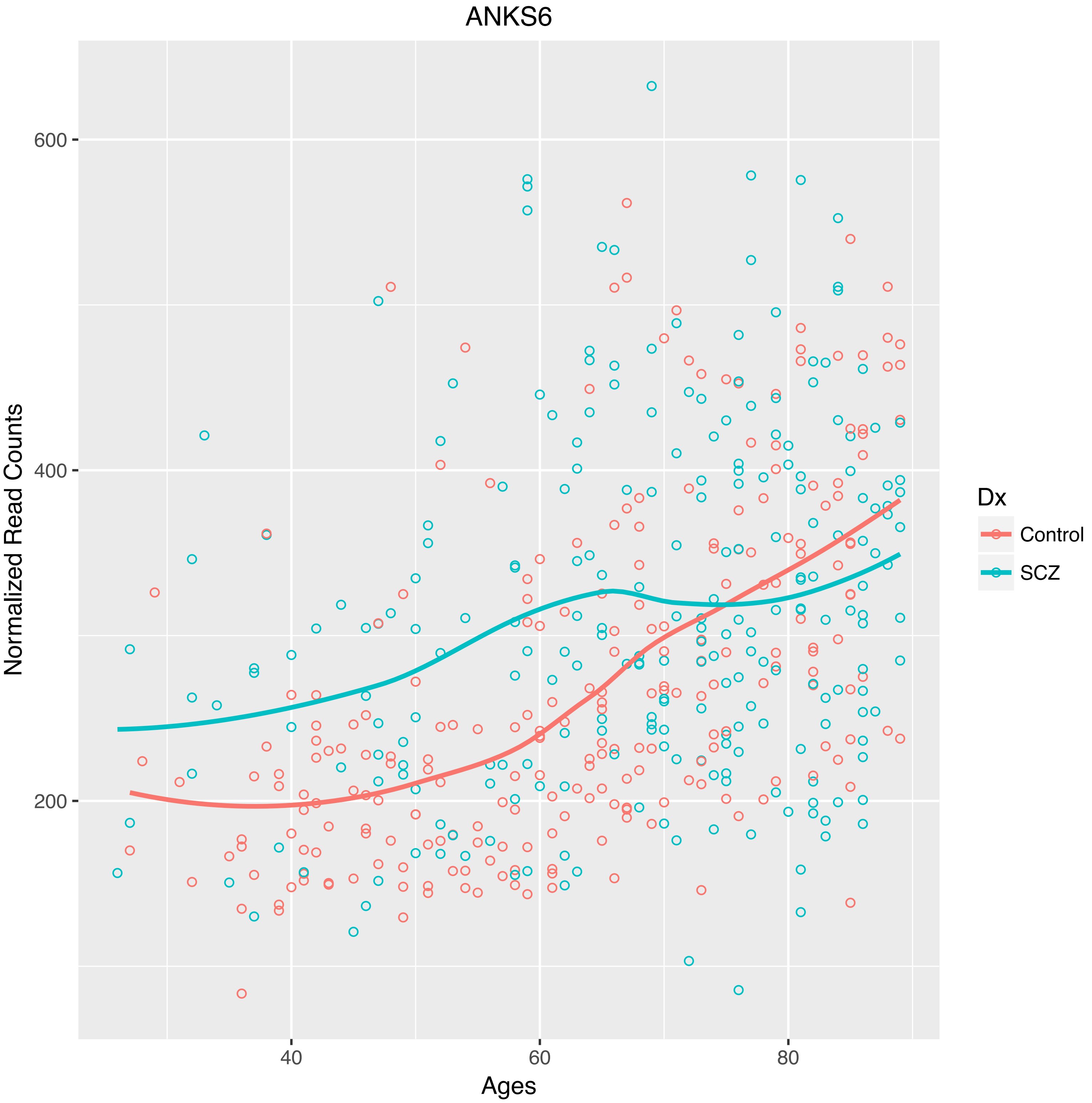
ANKS6
Normalized Read Counts
600
400
200
40
60
80
Ages
Dx
Control
SCZ

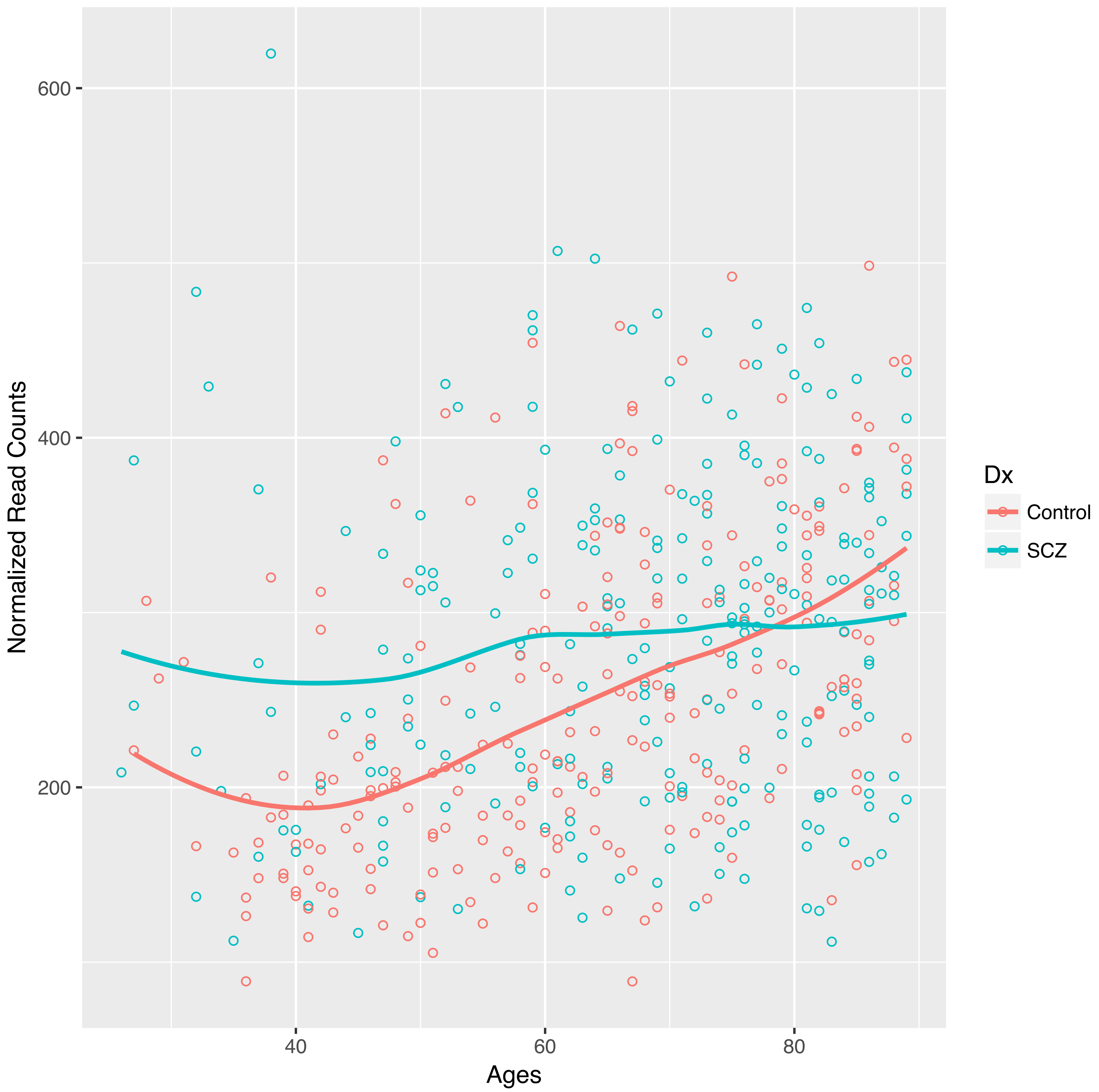
600
400
200
40
60
80
Ages
Normalized Read Counts
Dx
Control
SCZ

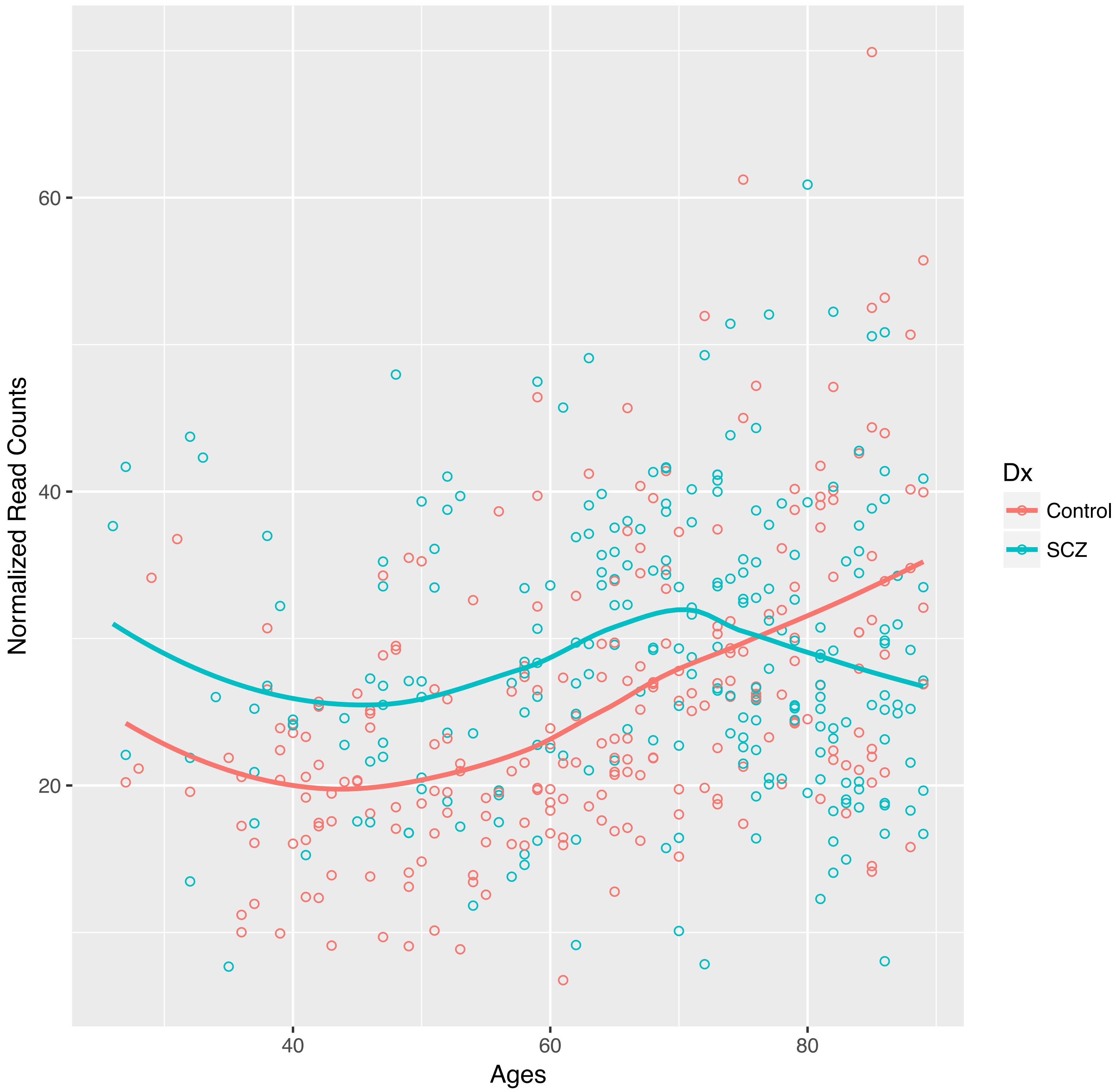

Normalized Read Counts
60
40
20
40
60
80
Ages
Dx
Control
SCZ

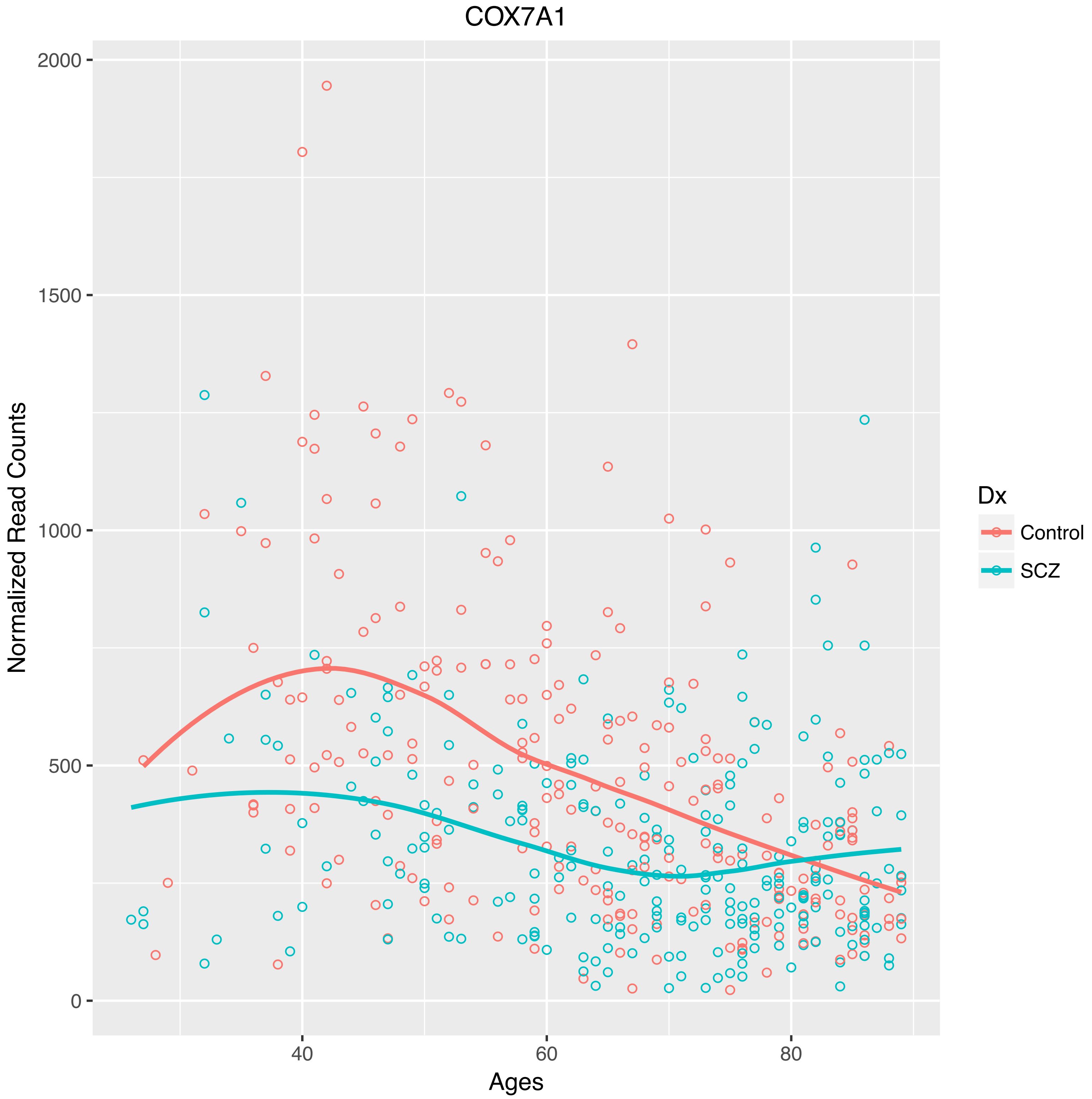

COX7A1
Normalized Read Counts
Ages
2000
1500
1000
500
0
40
60
80
Dx
Control
SCZ

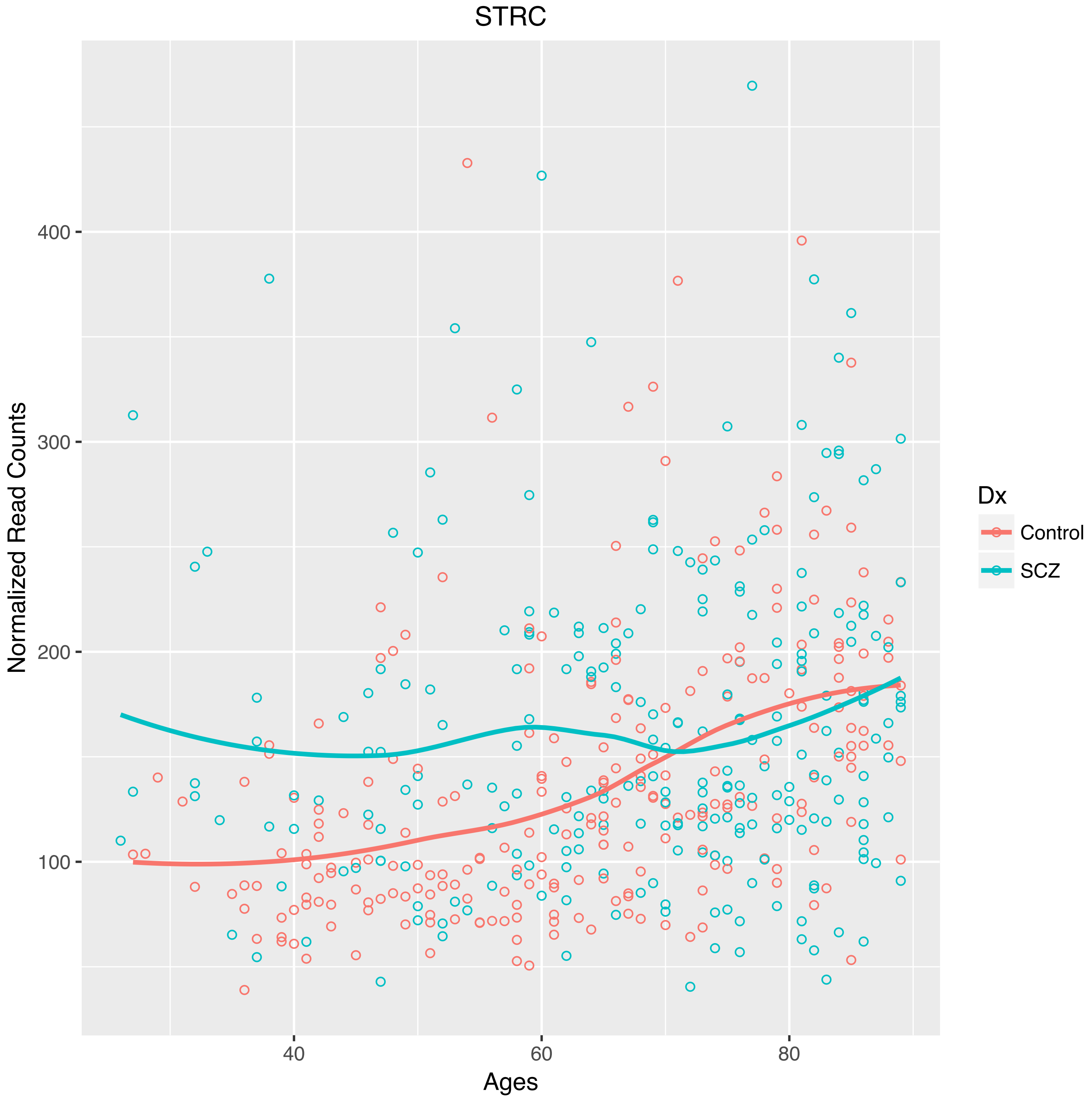

STRC
Normalized Read Counts
400
300
200
100
40
60
80
Ages
Dx
Control
SCZ

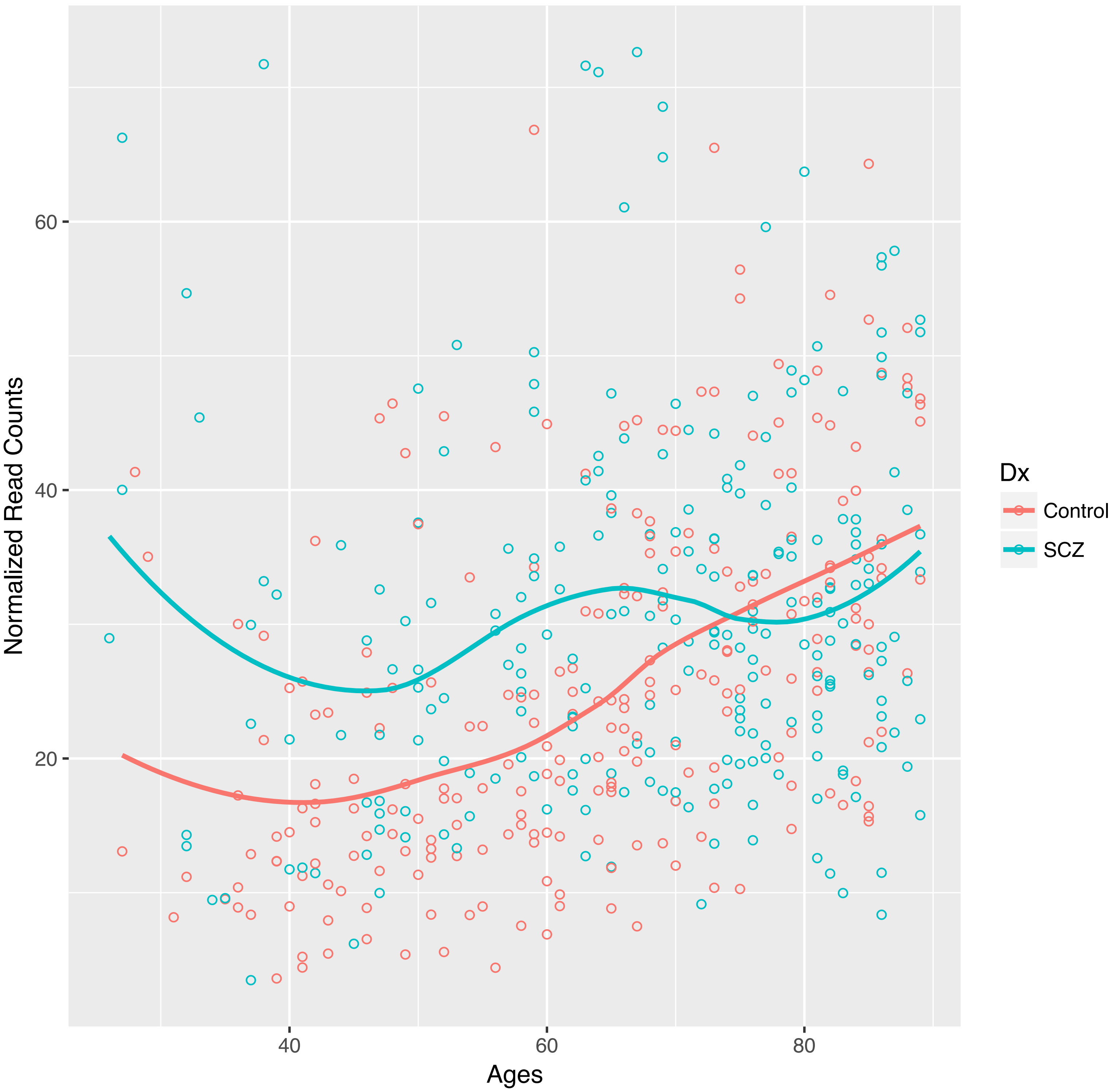
Normalized Read Counts
60
40
20
40
60
80
Ages
Dx
Control
SCZ

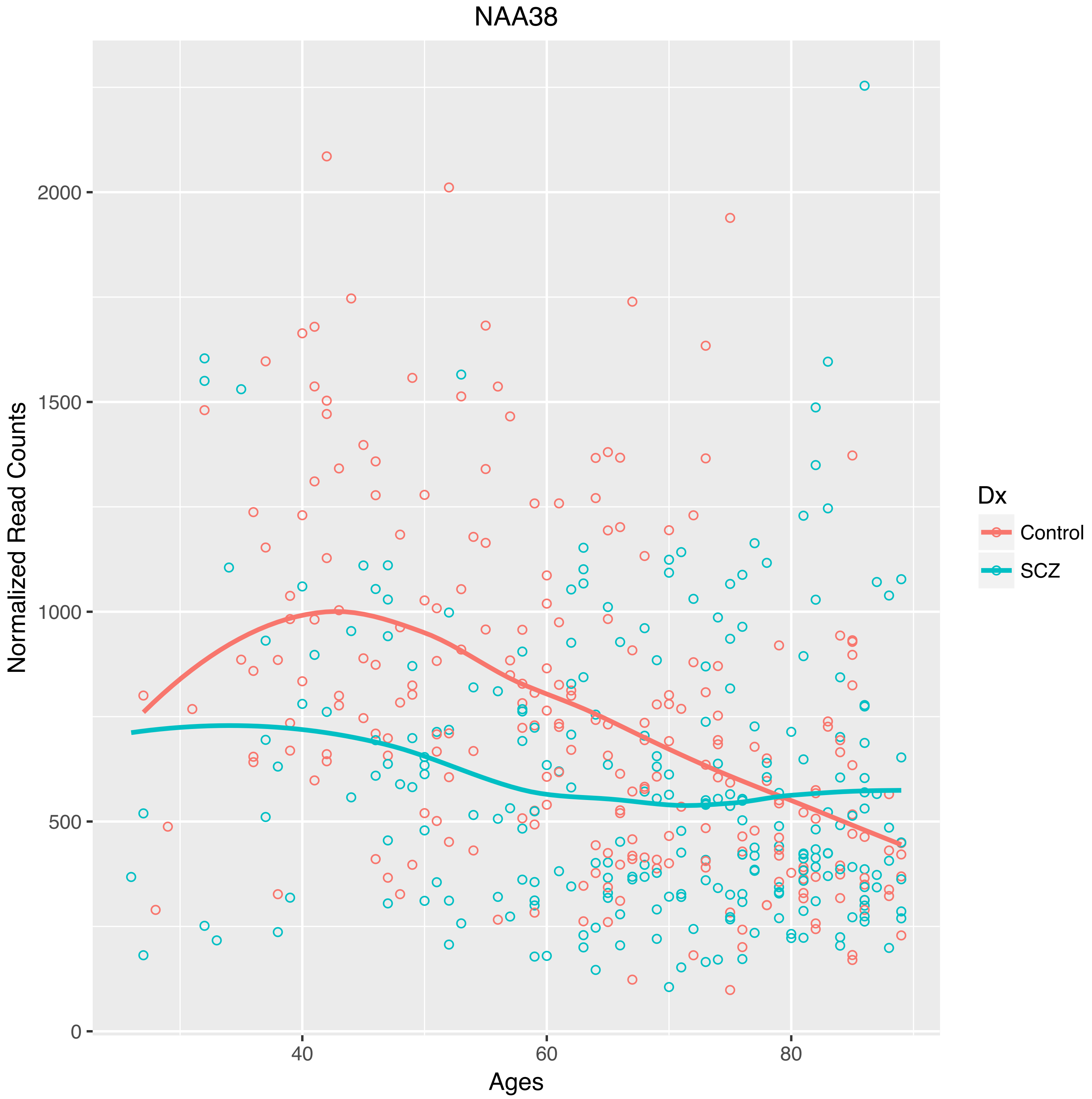

NAA38
Normalized Read Counts
0
500
1000
1500
2000
40
60
80
Ages
Dx
Control
SCZ

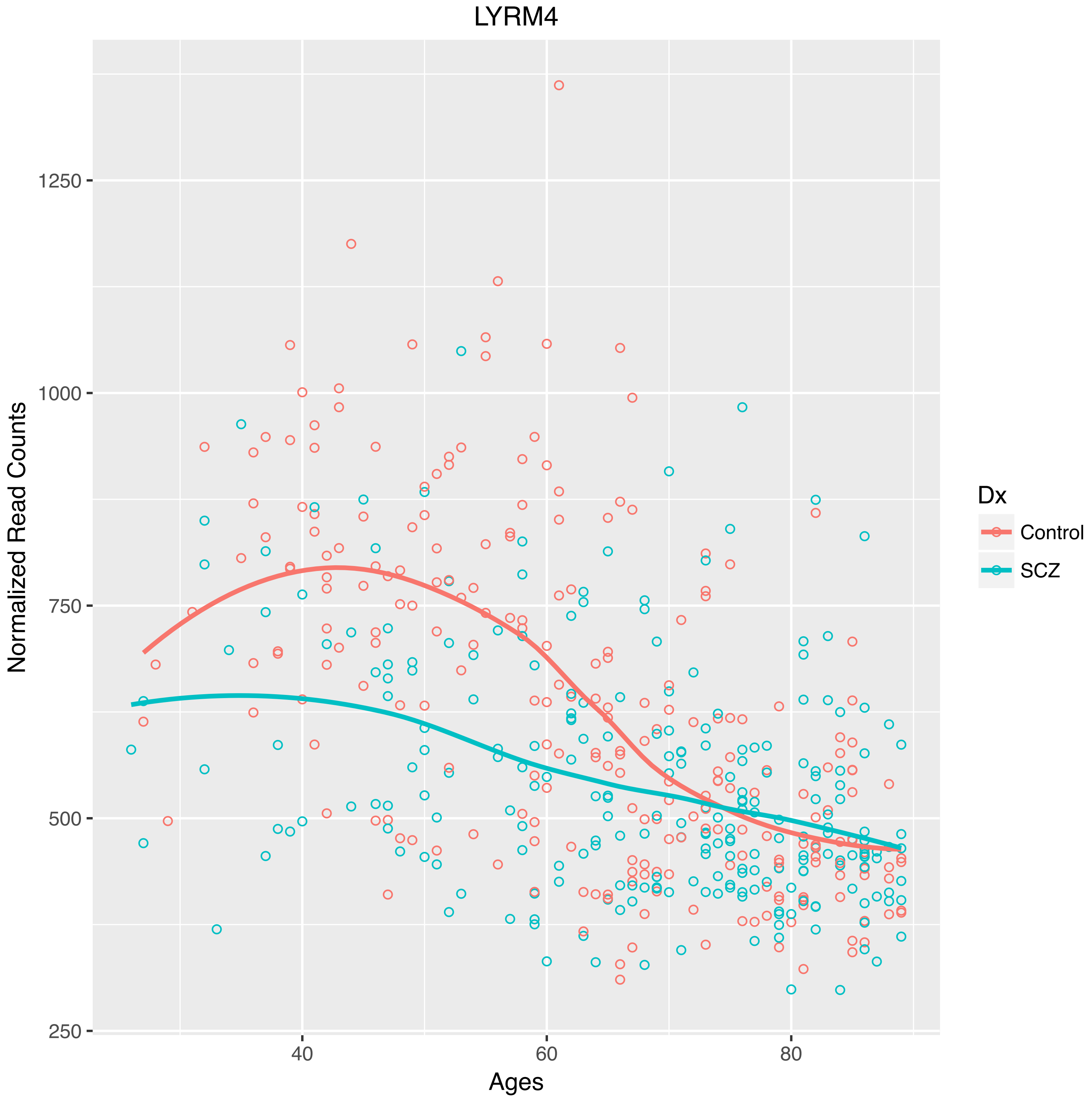

LYRM4
Normalized Read Counts
1250
1000
750
500
250
40
60
80
Ages
Dx
Control
SCZ

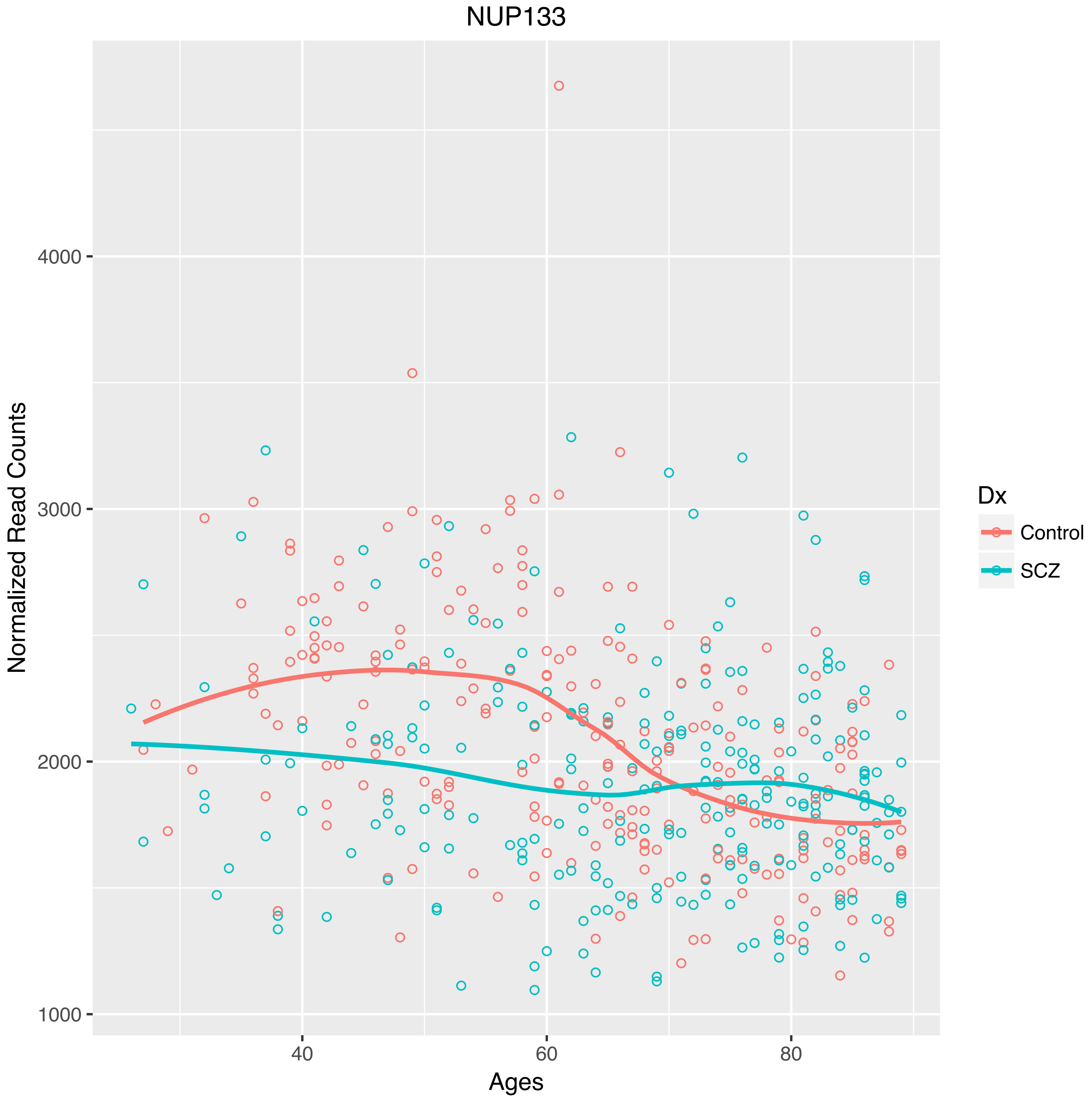

NUP133
Normalized Read Counts
1000
2000
3000
4000
40
60
80
Ages
Dx
Control
SCZ

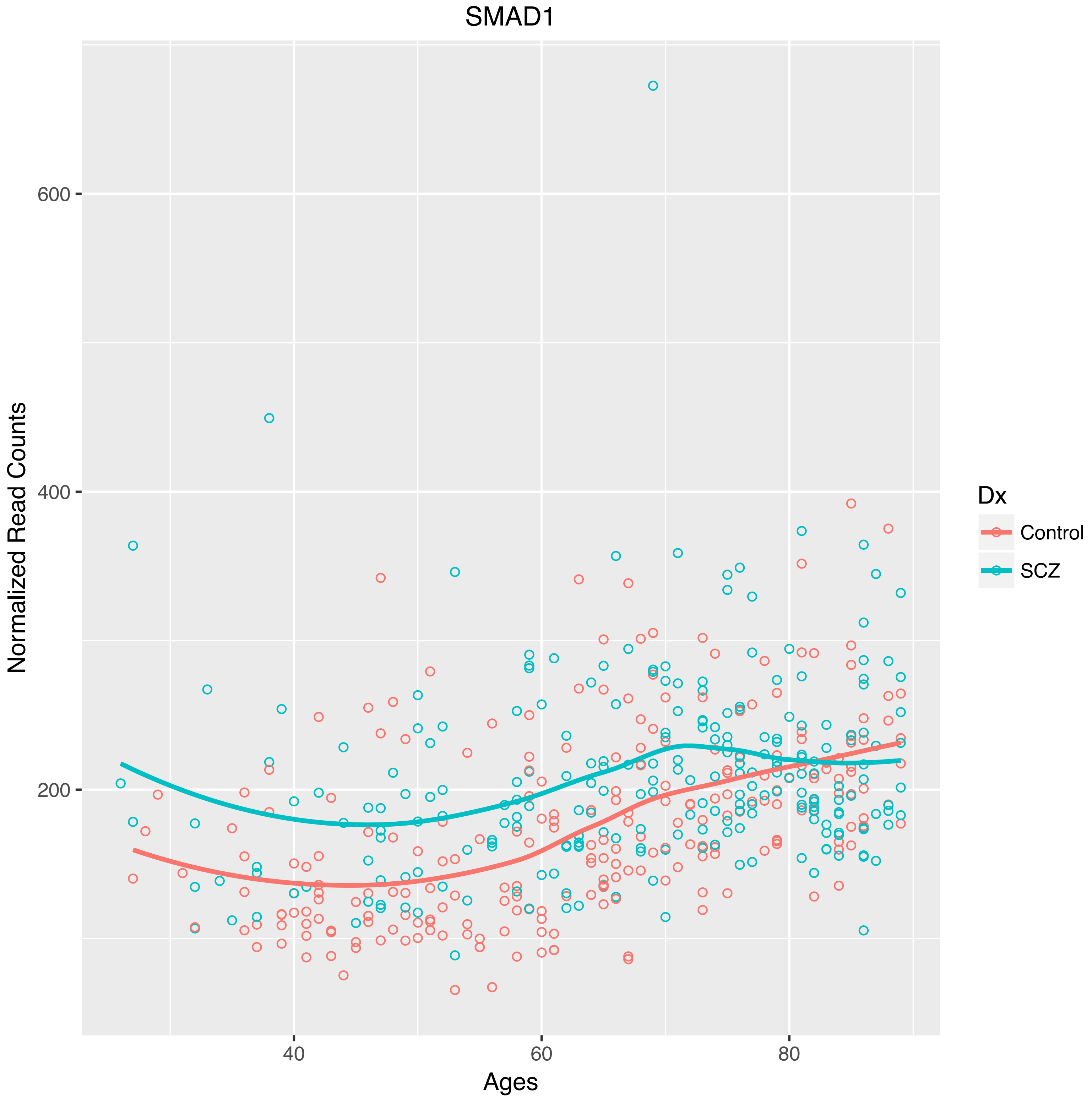

SMAD1
Normalized Read Counts
600
400
200
40
60
80
Ages
Dx
Control
SCZ

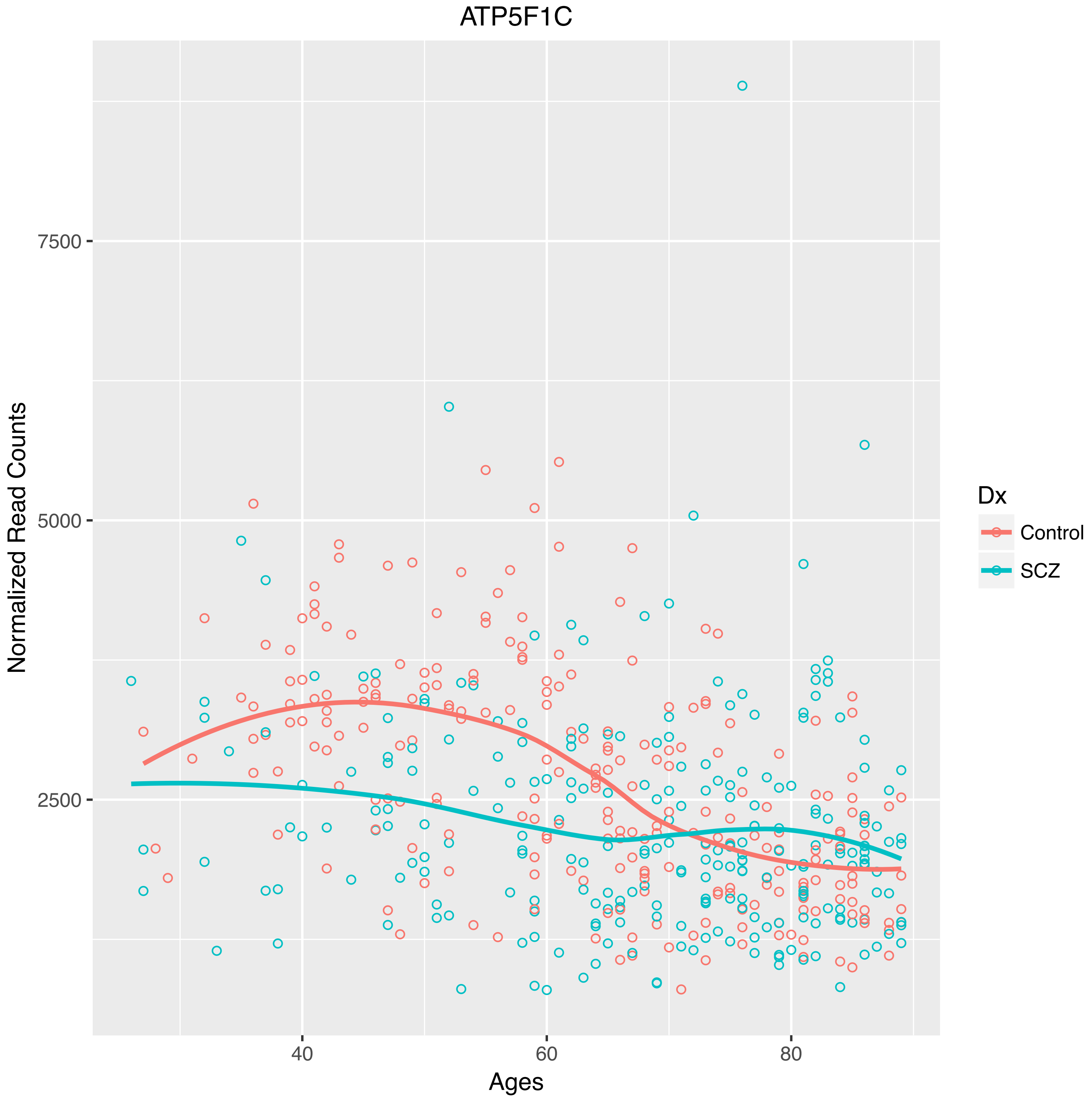

ATP5F1C
Normalized Read Counts
7500
5000
2500
40
60
80
Ages
Dx
Control
SCZ

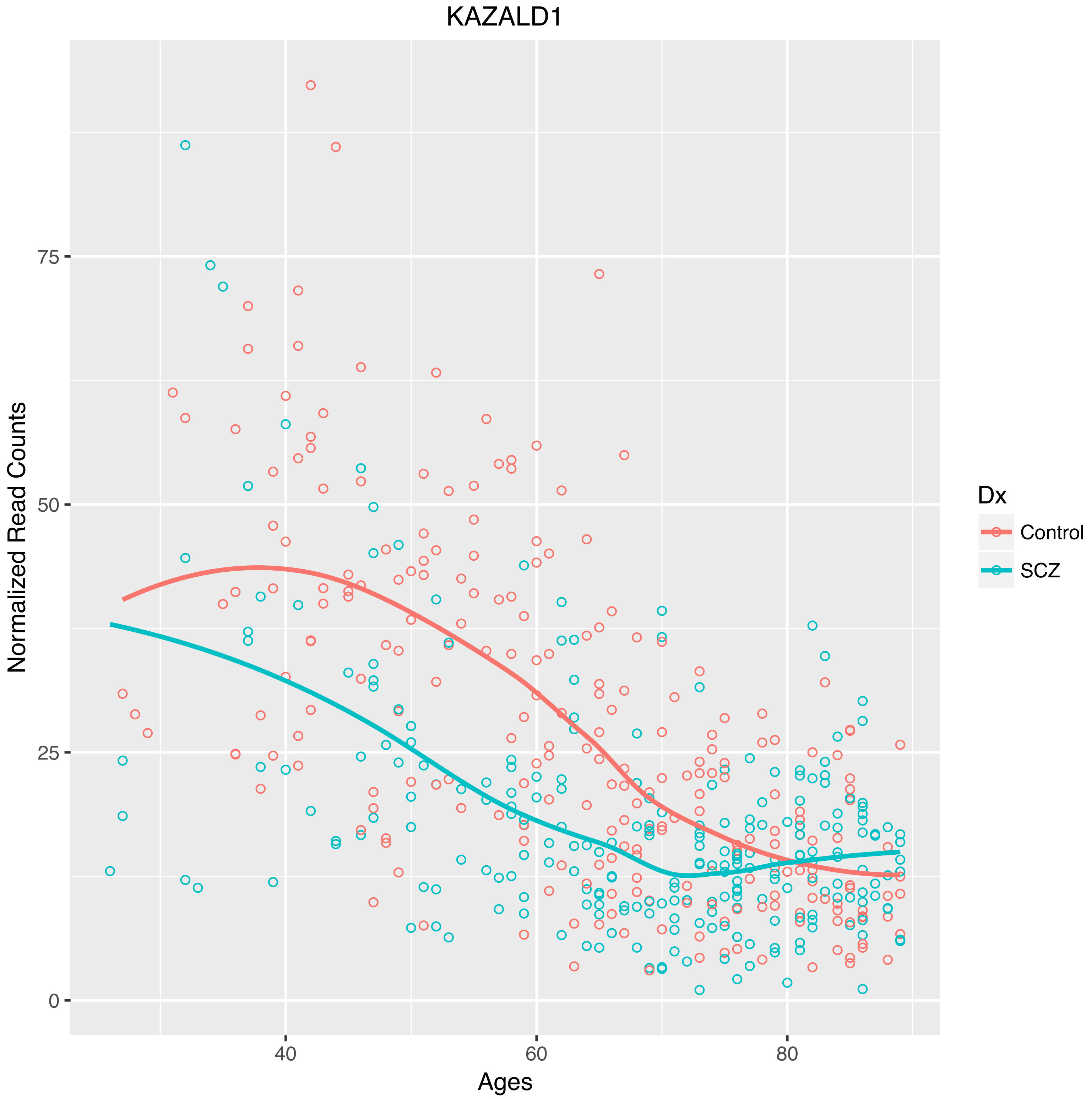

KAZALD1
Normalized Read Counts
75
50
25
0
40
60
80
Ages
Dx
Control
SCZ

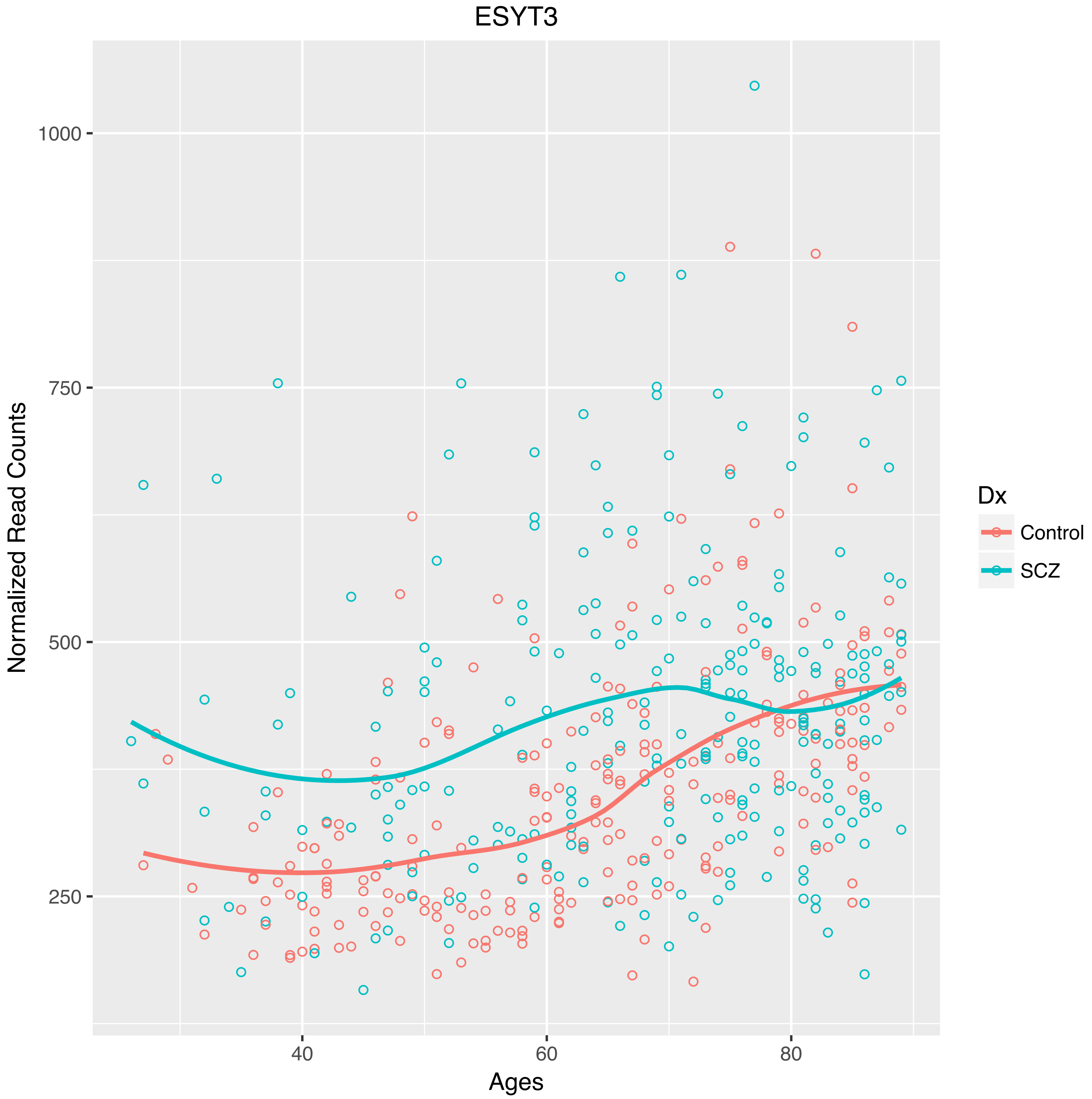

ESYT3
Normalized Read Counts
1000
750
500
250
40
60
80
Ages
Dx
Control
SCZ

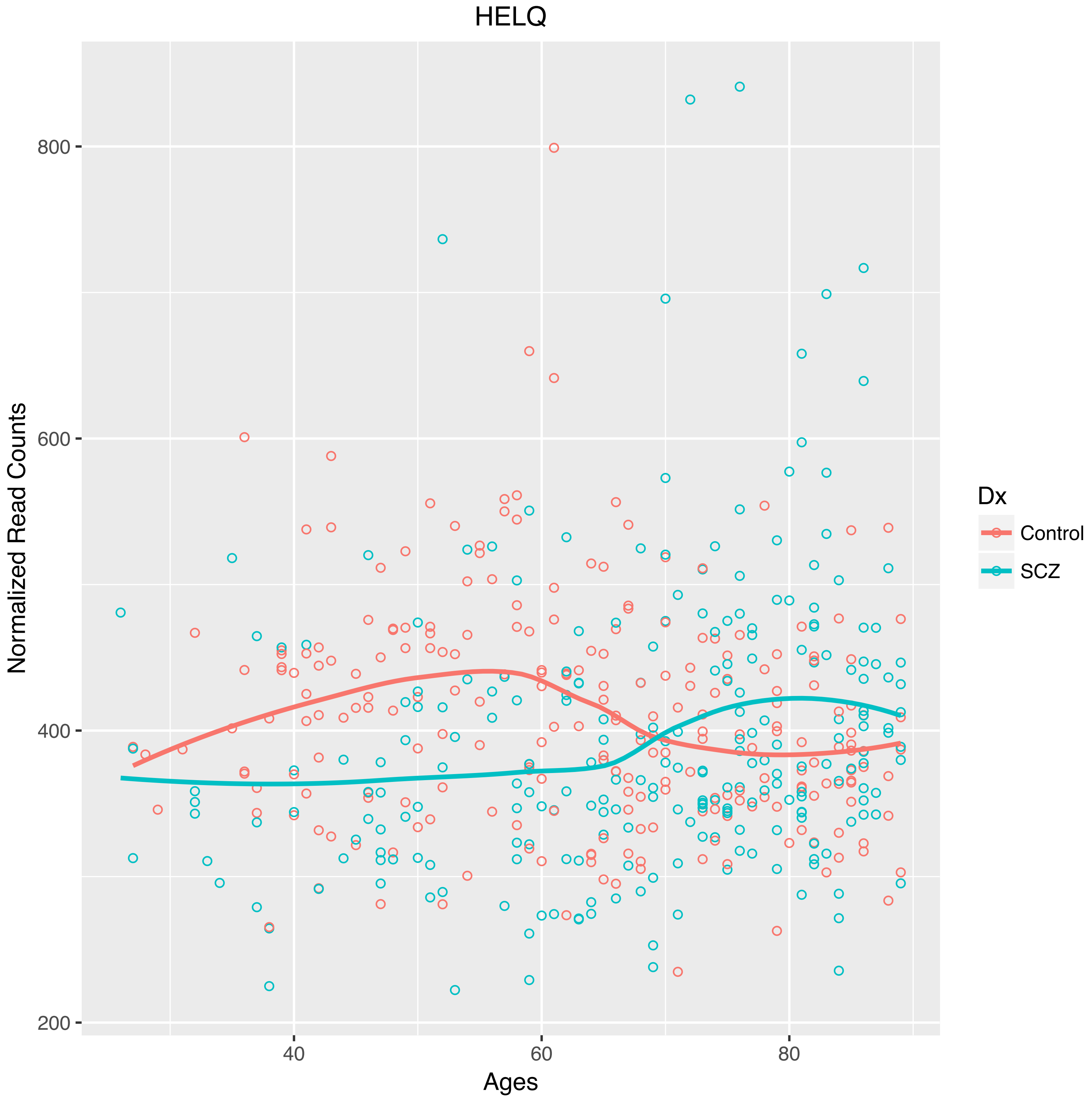

HELQ
Normalized Read Counts
800
600
400
200
40
60
80
Ages
Dx
Control
SCZ

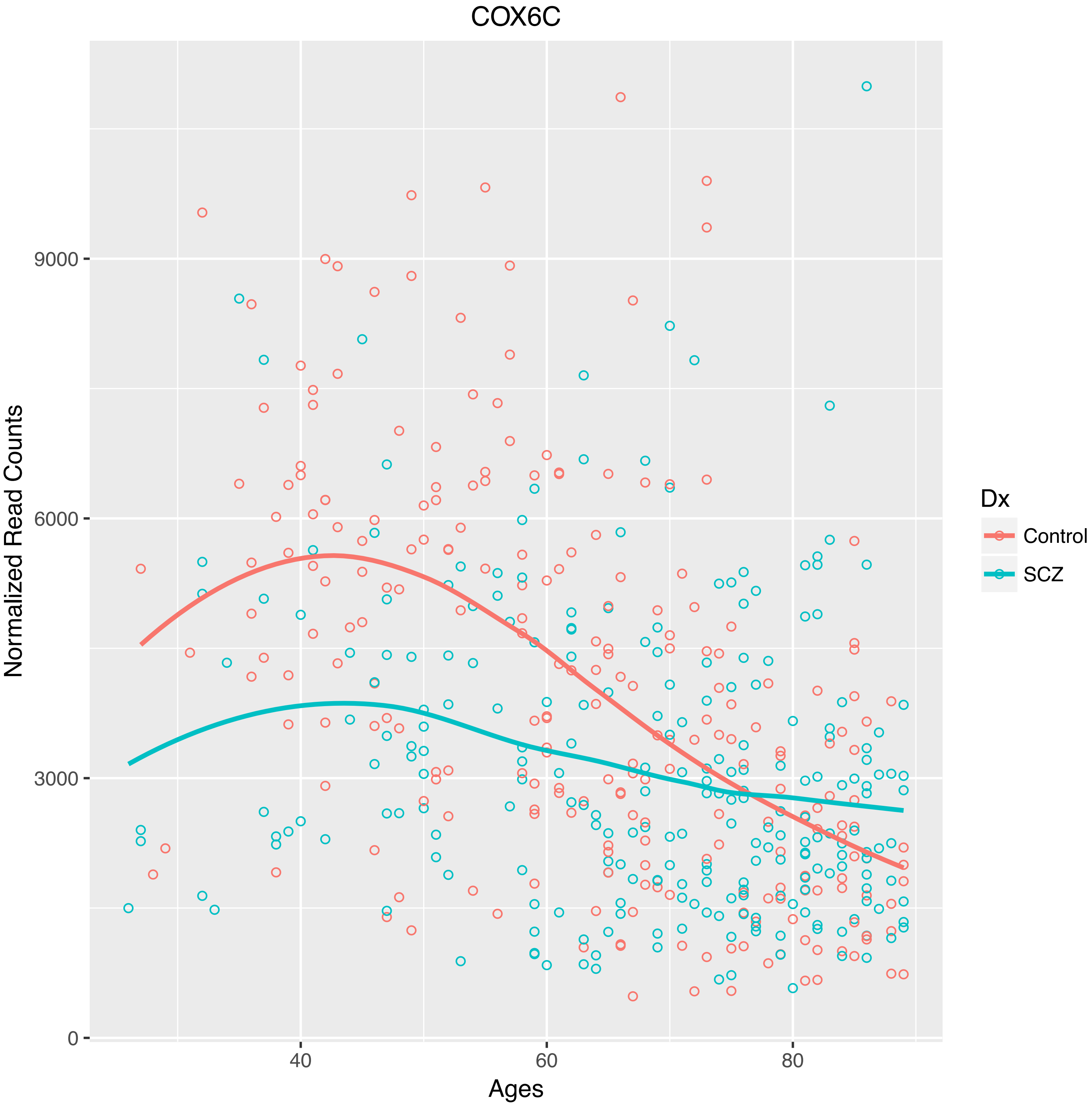
COX6C
Normalized Read Counts
9000
6000
3000
0
40
60
80
Ages
Dx
Control
SCZ

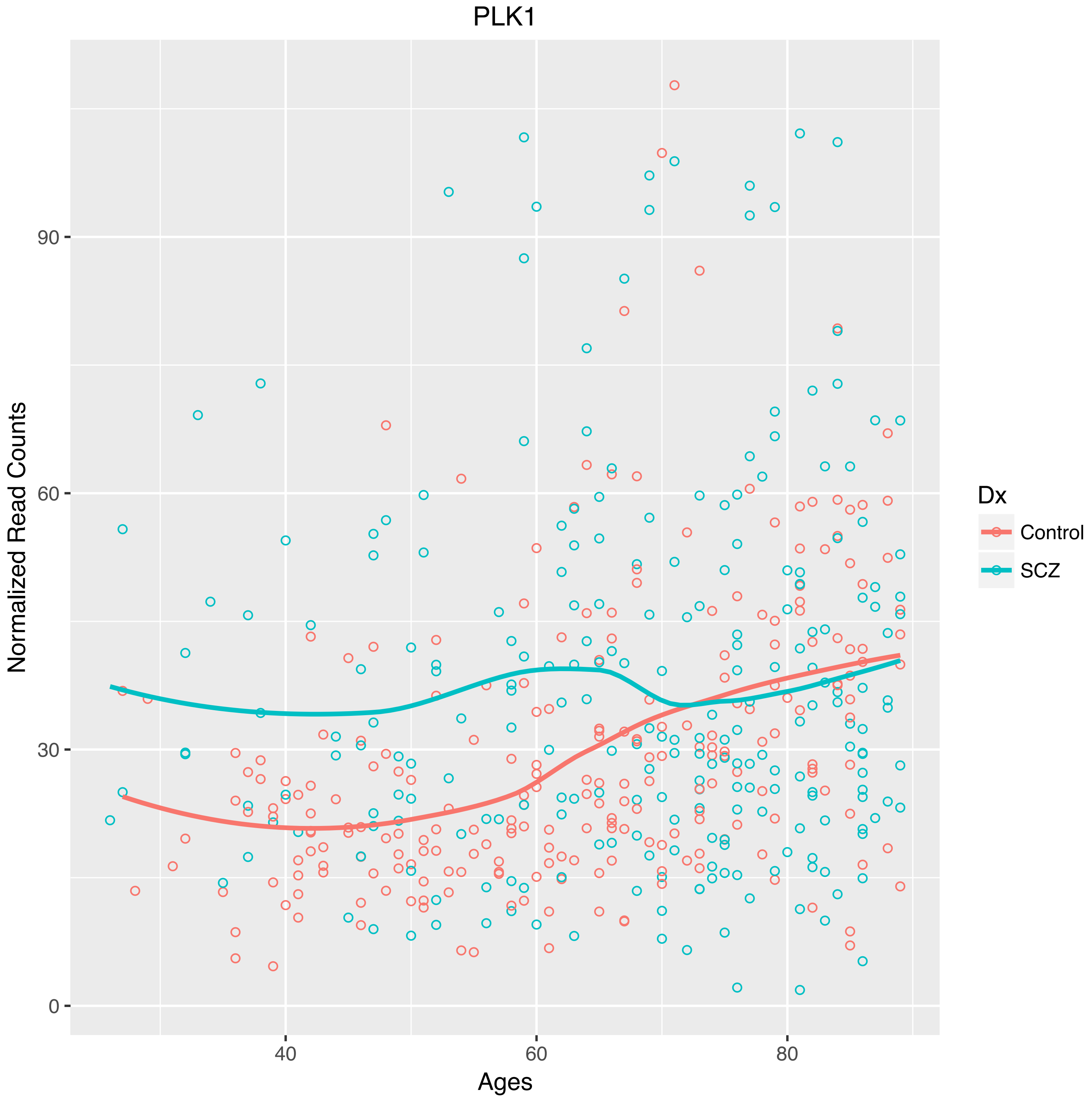

PLK1
Normalized Read Counts
90
60
30
0
40
60
80
Ages
Dx
Control
SCZ

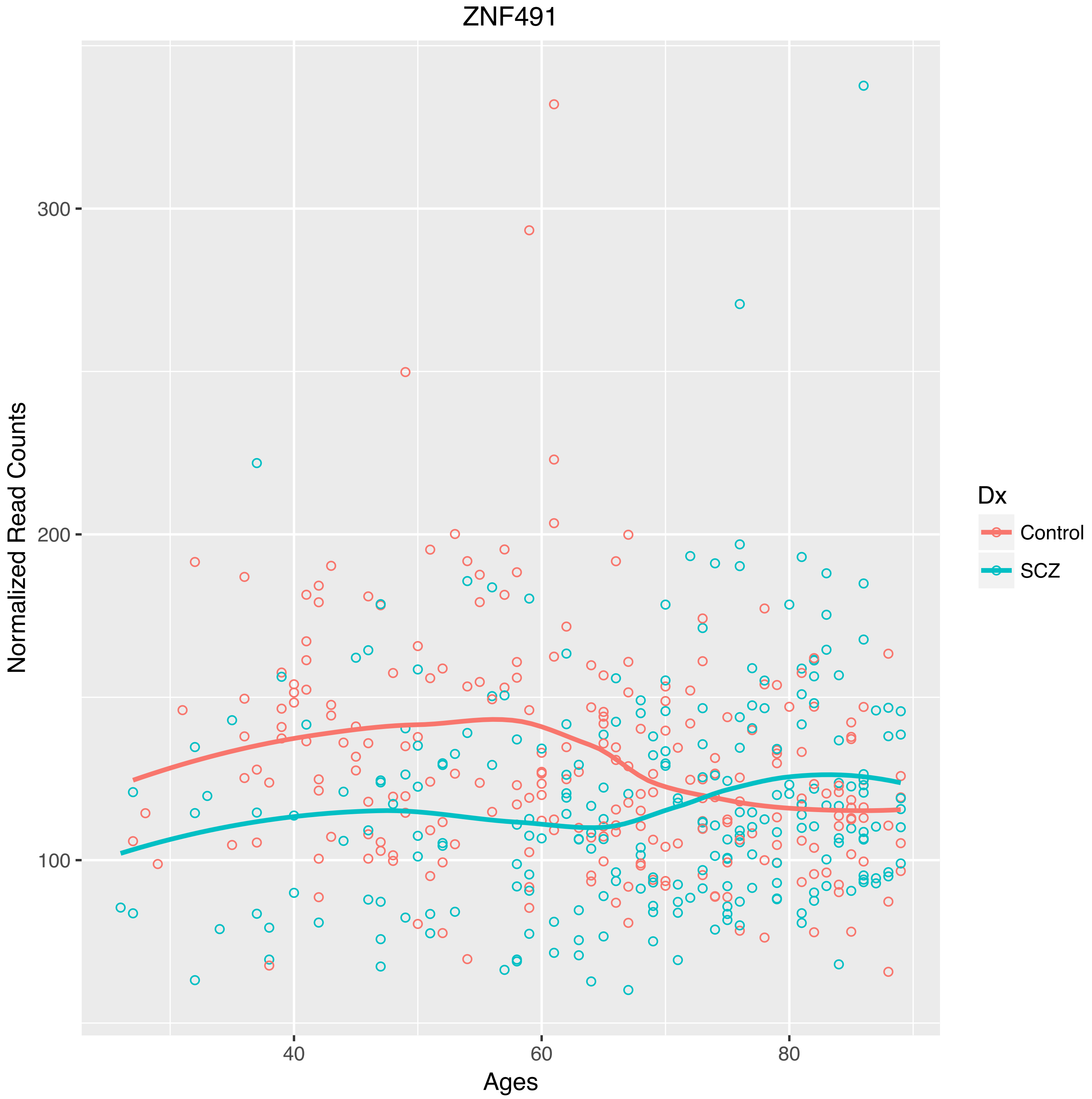
ZNF491
Normalized Read Counts
300
200
100
40
60
80
Ages
Dx
Control
SCZ

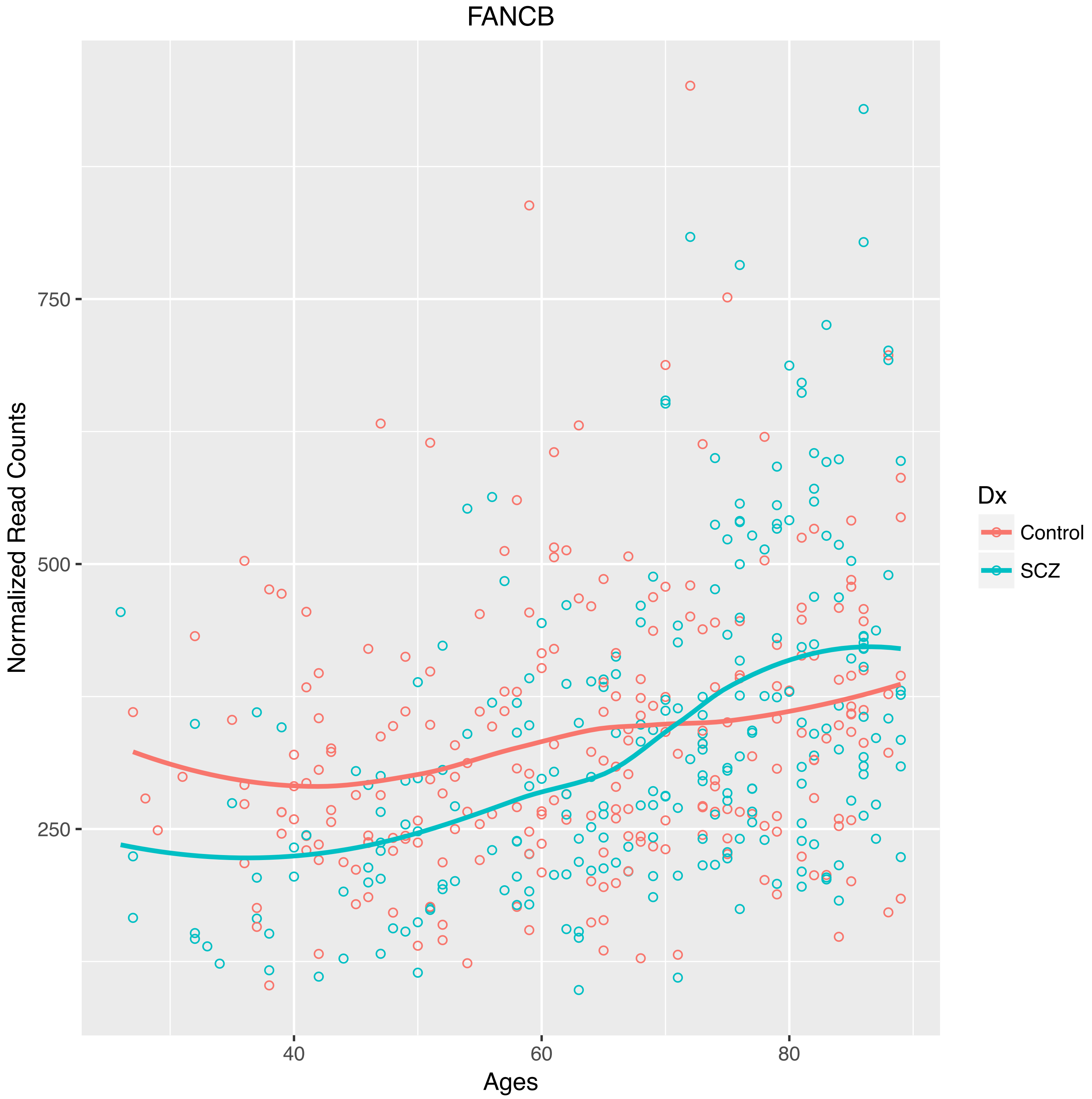
FANCB
Normalized Read Counts
750
500
250
40
60
80
Ages
Dx
Control
SCZ

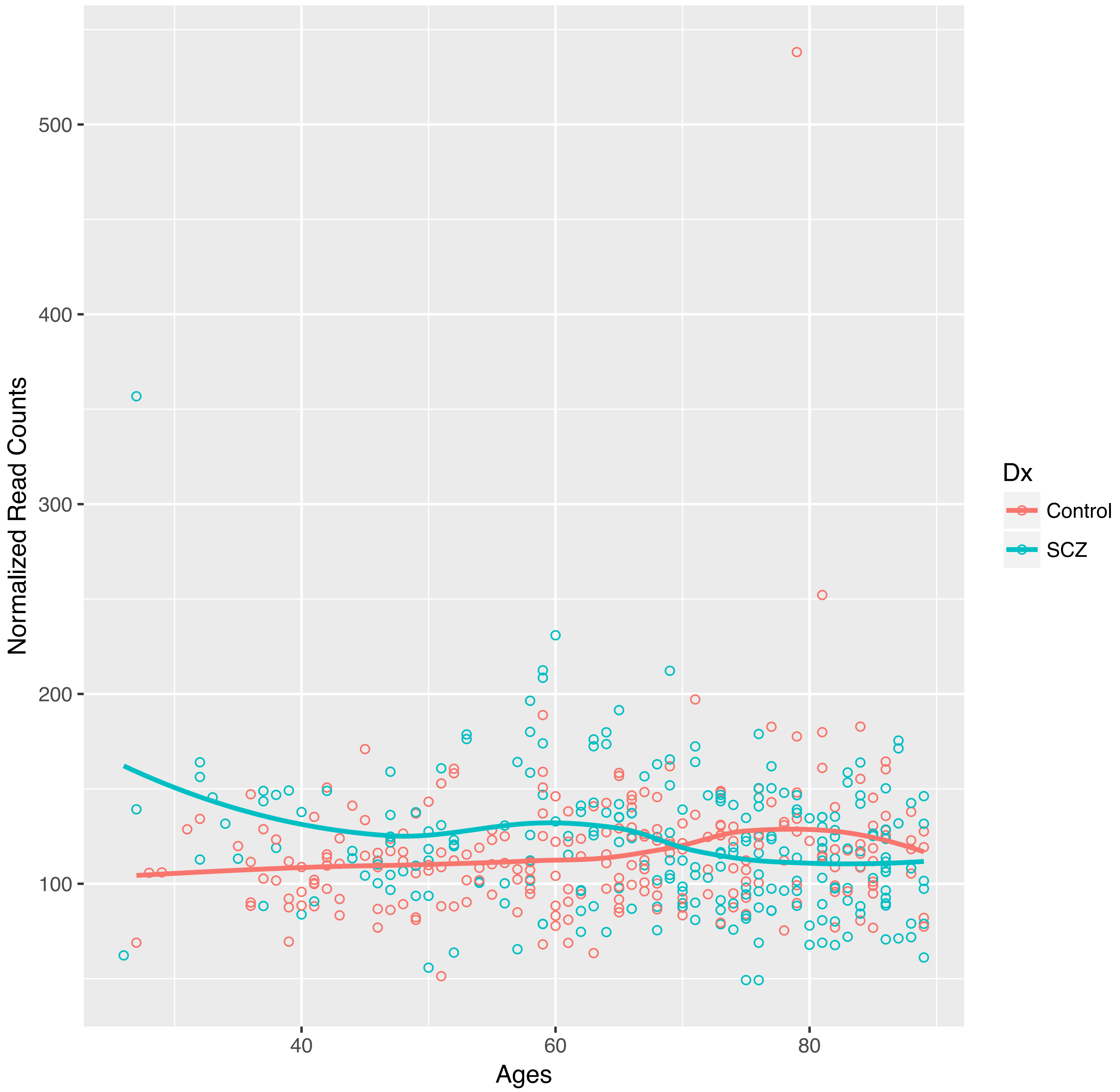

Normalized Read Counts
500
400
300
200
100
40
60
80
Ages
Dx
Control
SCZ

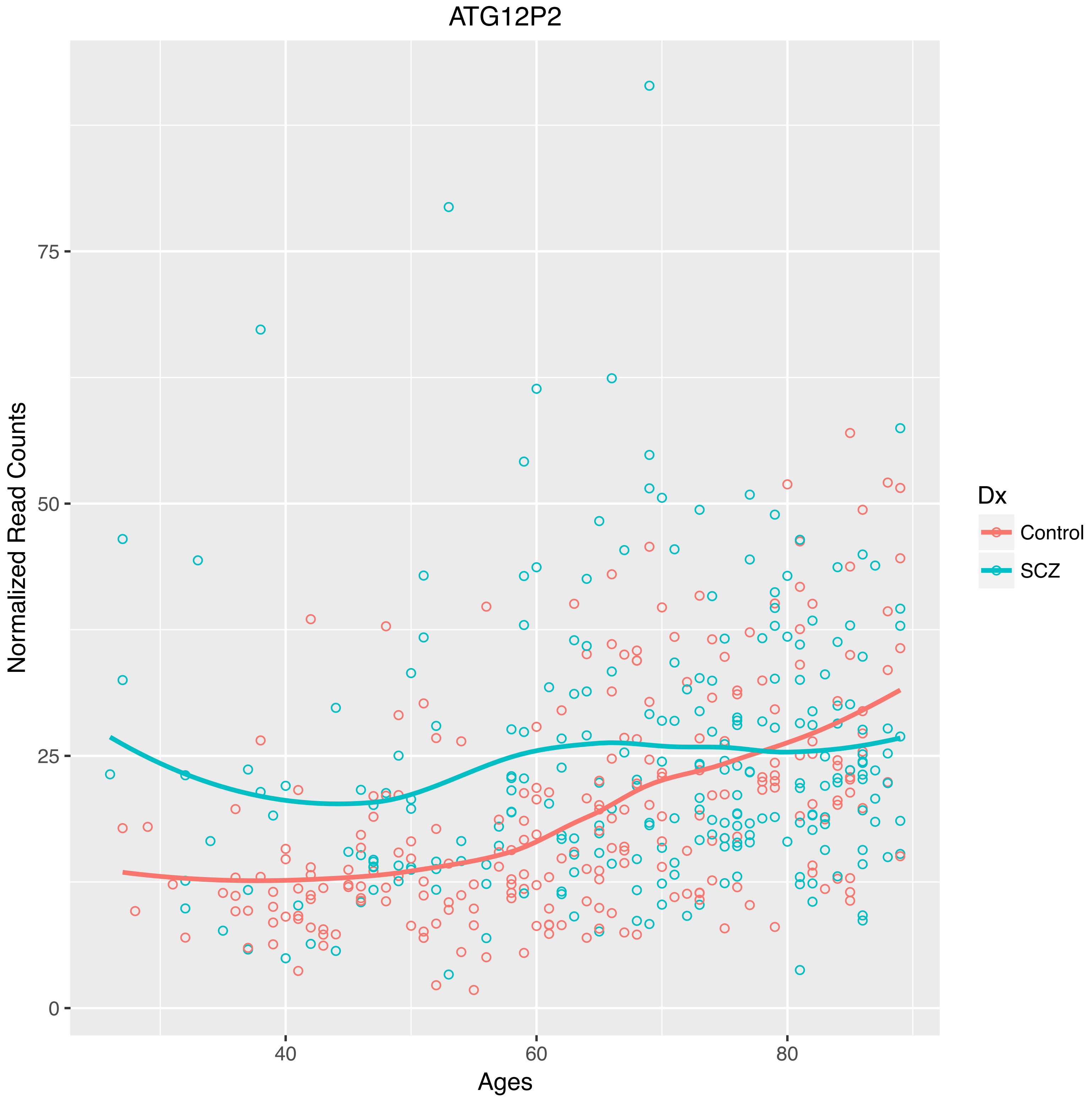

ATG12P2
Normalized Read Counts
0
25
50
75
40
60
80
Ages
Dx
Control
SCZ

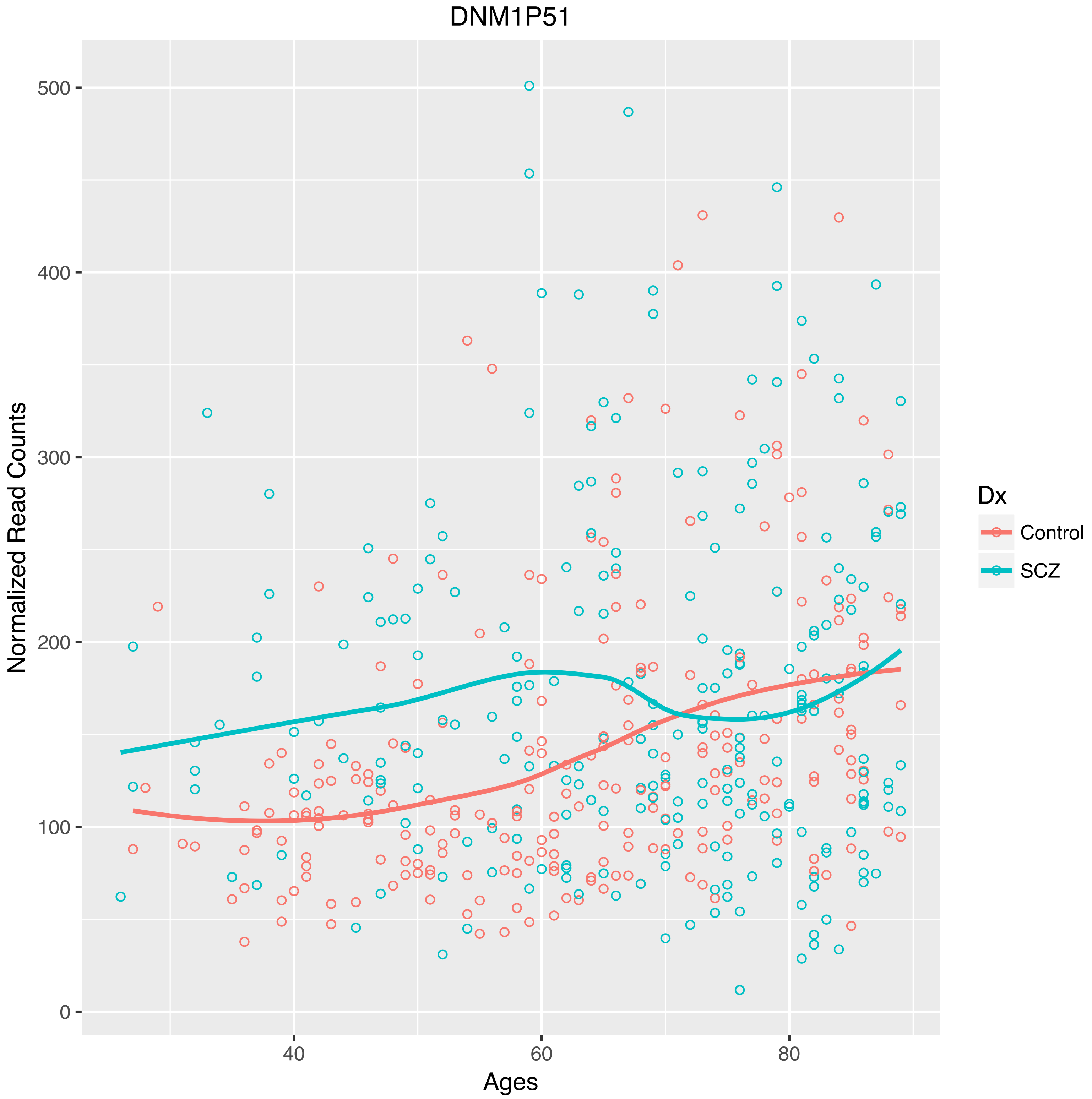

DNM1P51
Normalized Read Counts
0
100
200
300
400
500
40
60
80
Ages
Dx
Control
SCZ

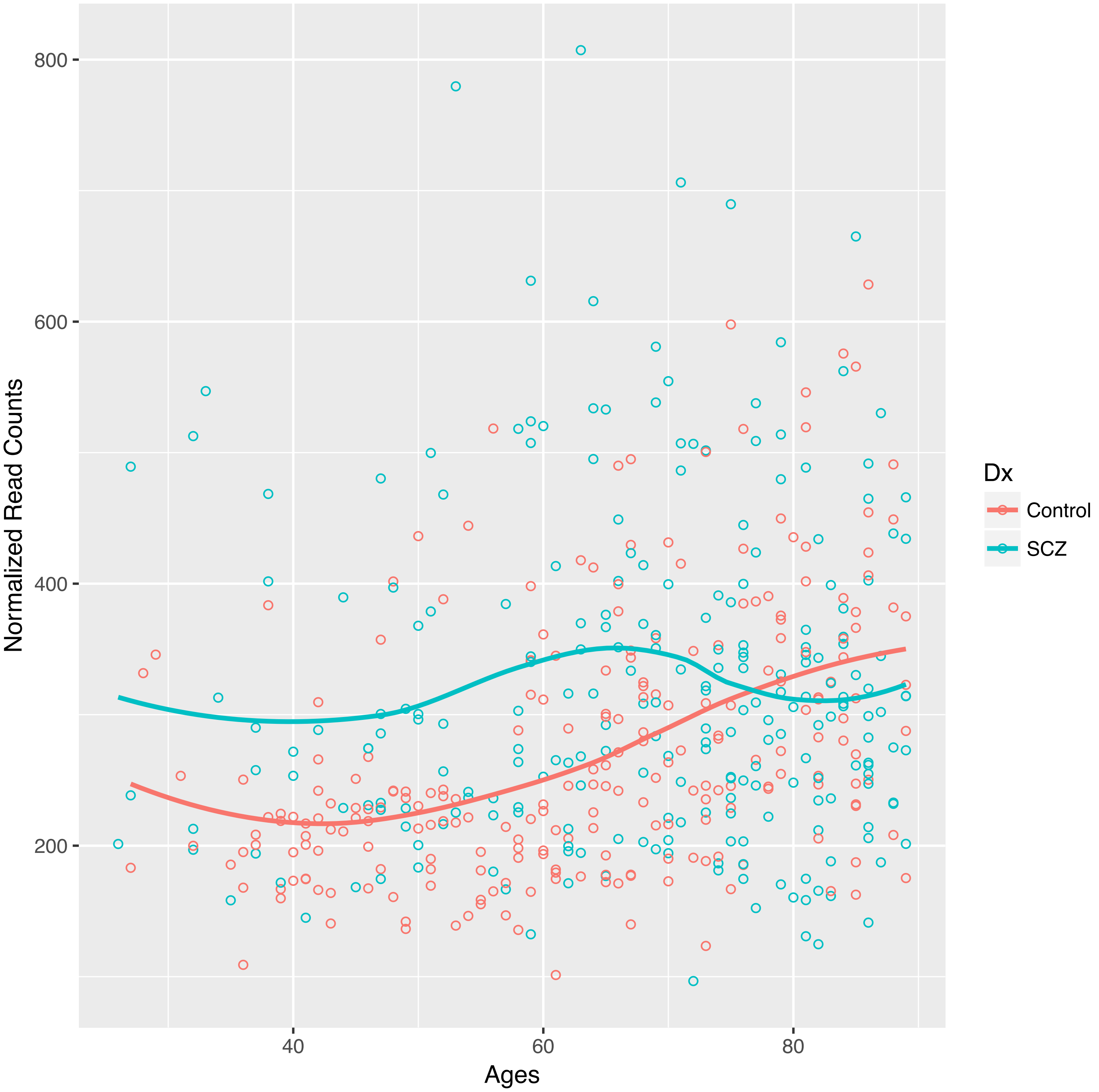

Normalized Read Counts
800
600
400
200
40
60
80
Ages
Dx
Control
SCZ

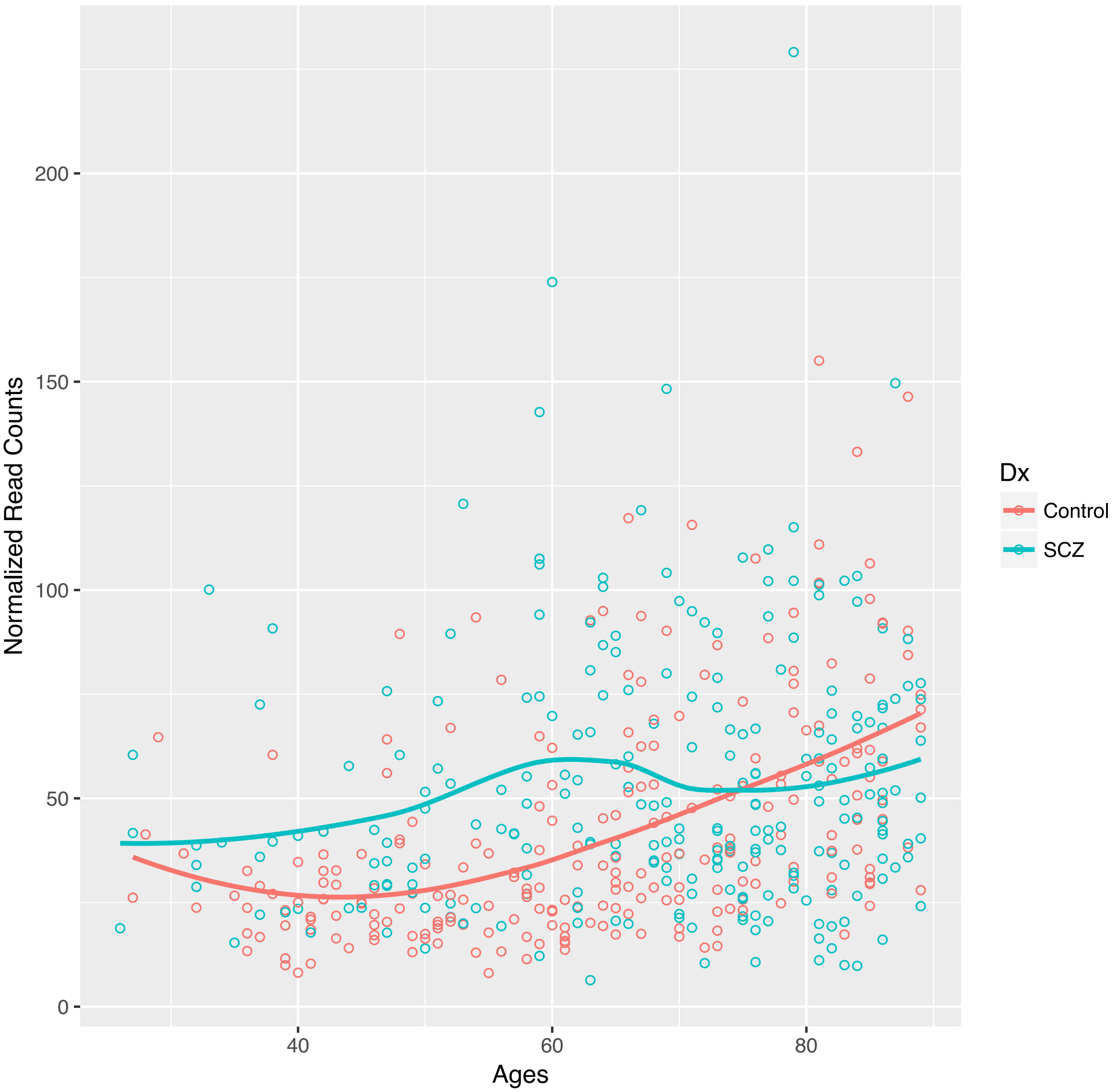

Normalized Read Counts
0
50
100
150
200
40
60
80
Ages
Dx
Control
SCZ

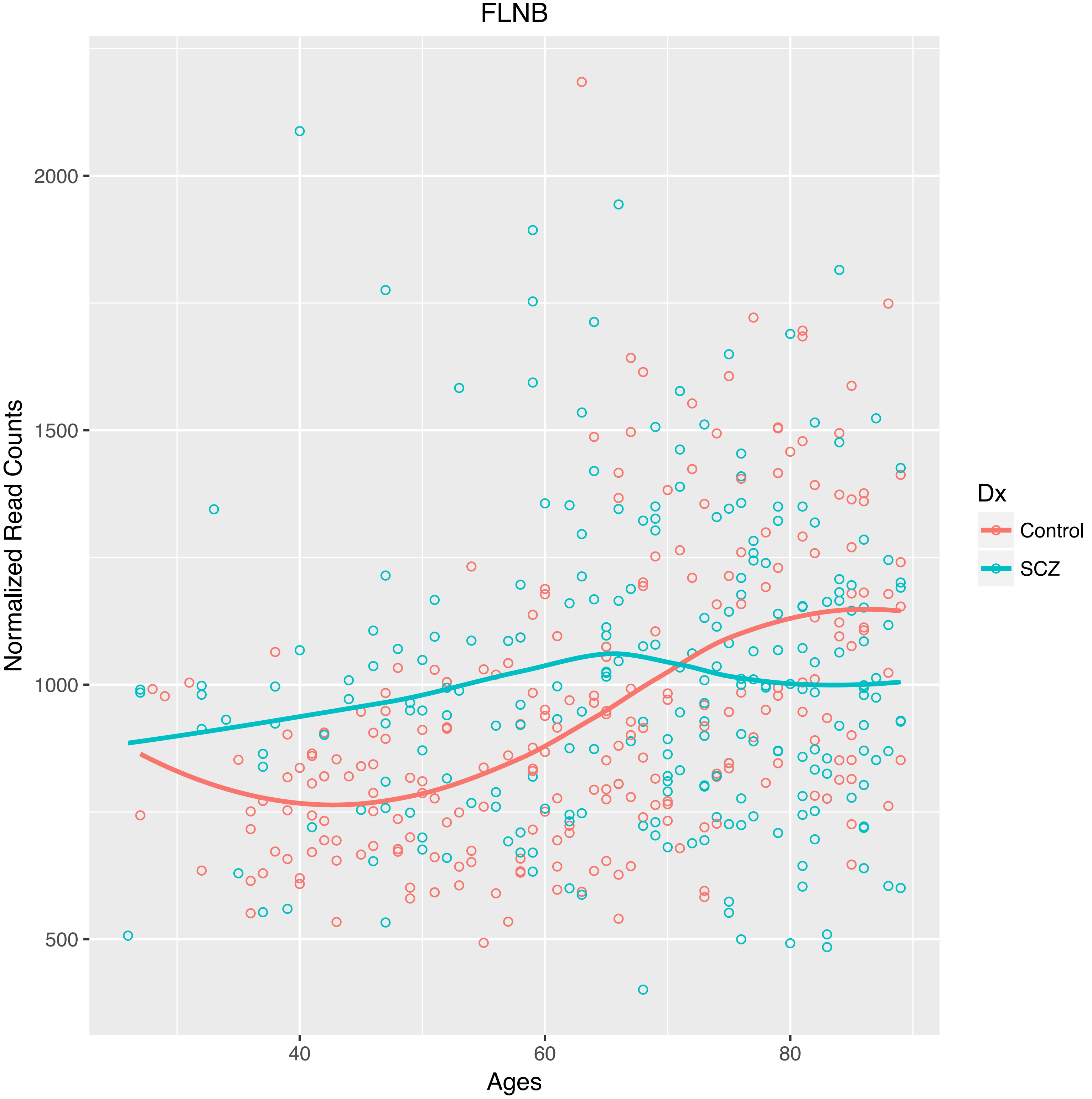
FLNB
Normalized Read Counts
2000
1500
1000
500
40
60
80
Ages
Dx
Control
SCZ

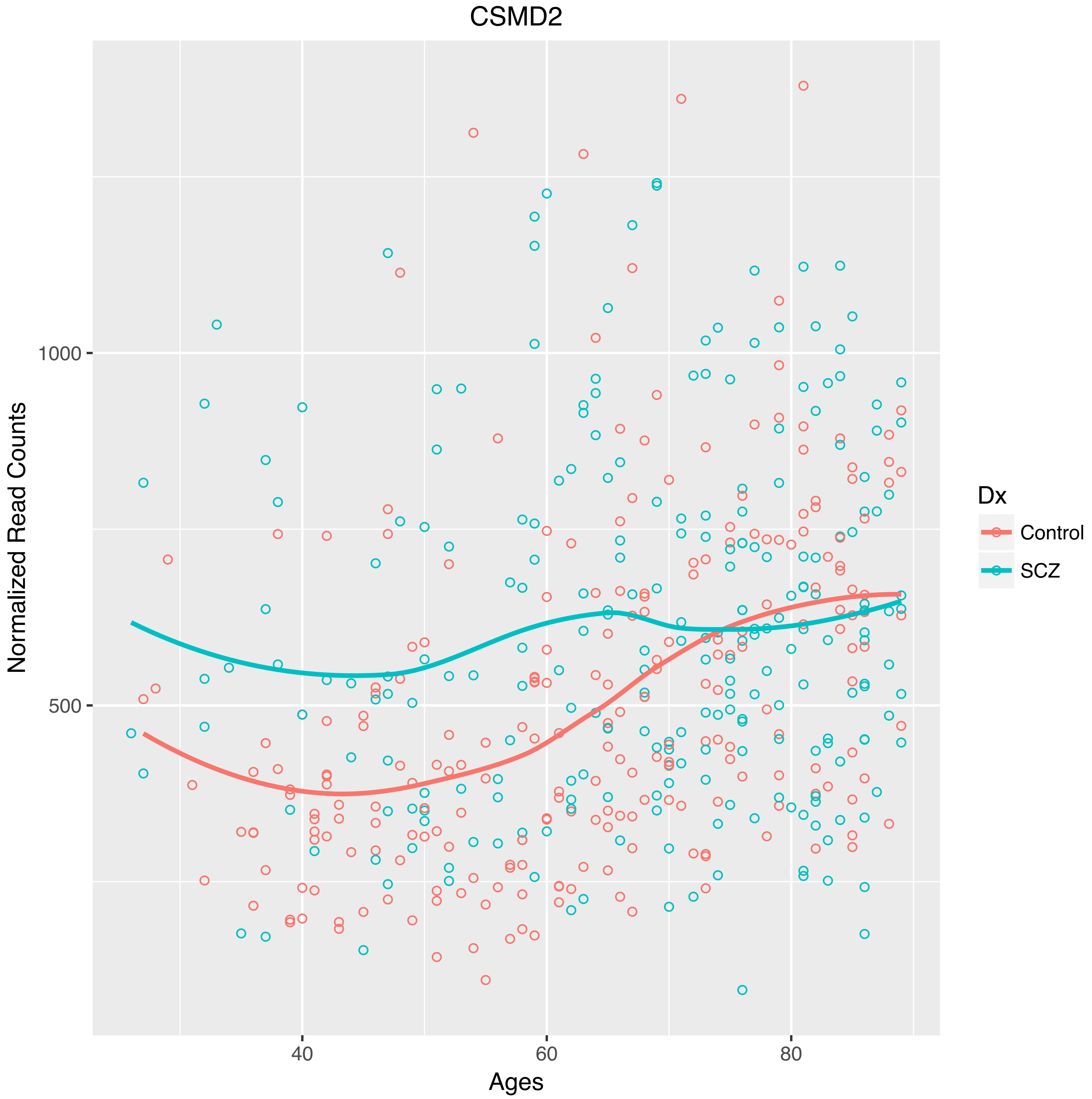
CSMD2
Normalized Read Counts
1000
500
40
60
80
Ages
Dx
Control
SCZ

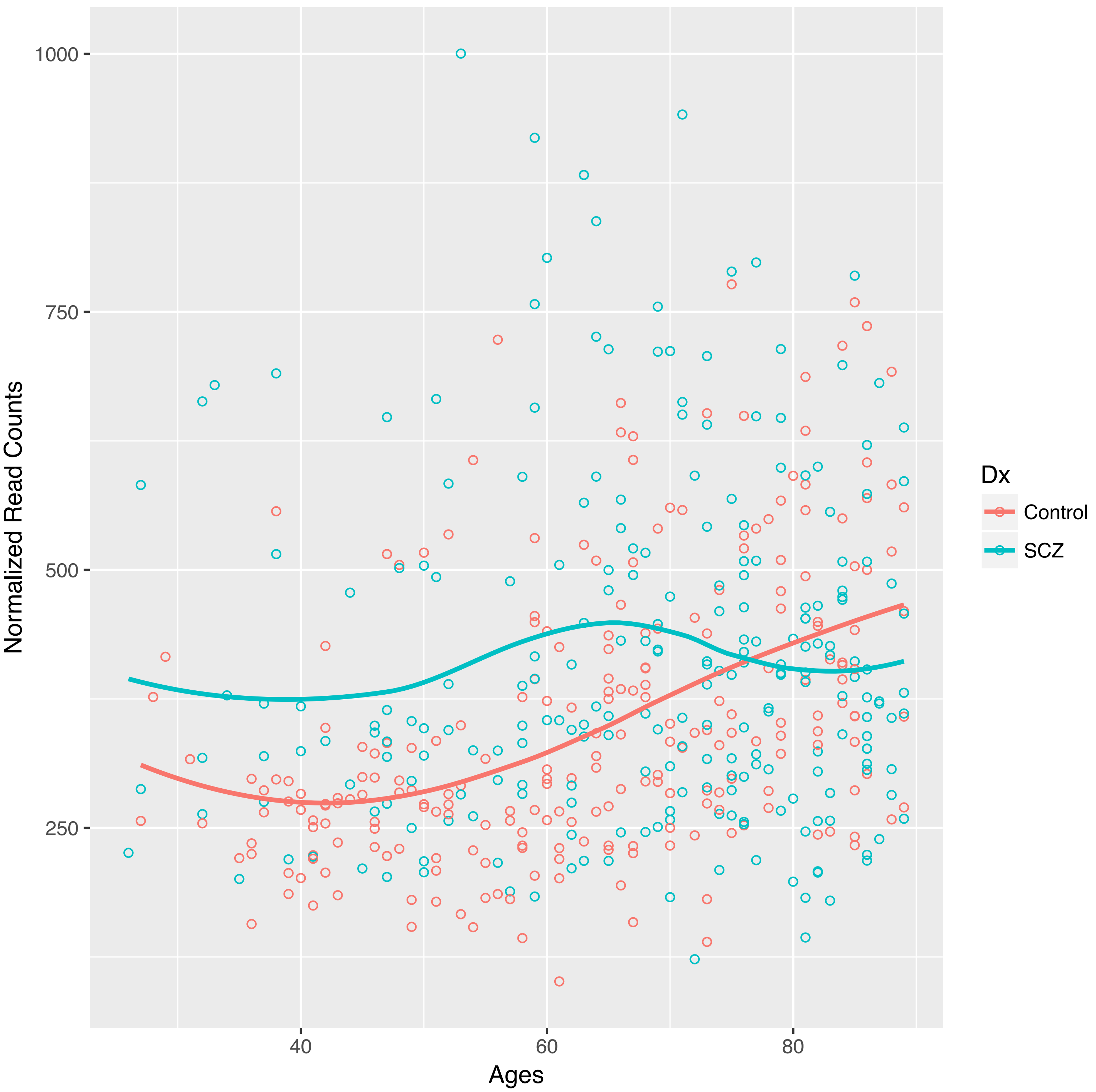

Normalized Read Counts
1000
750
500
250
40
60
80
Ages
Dx
Control
SCZ

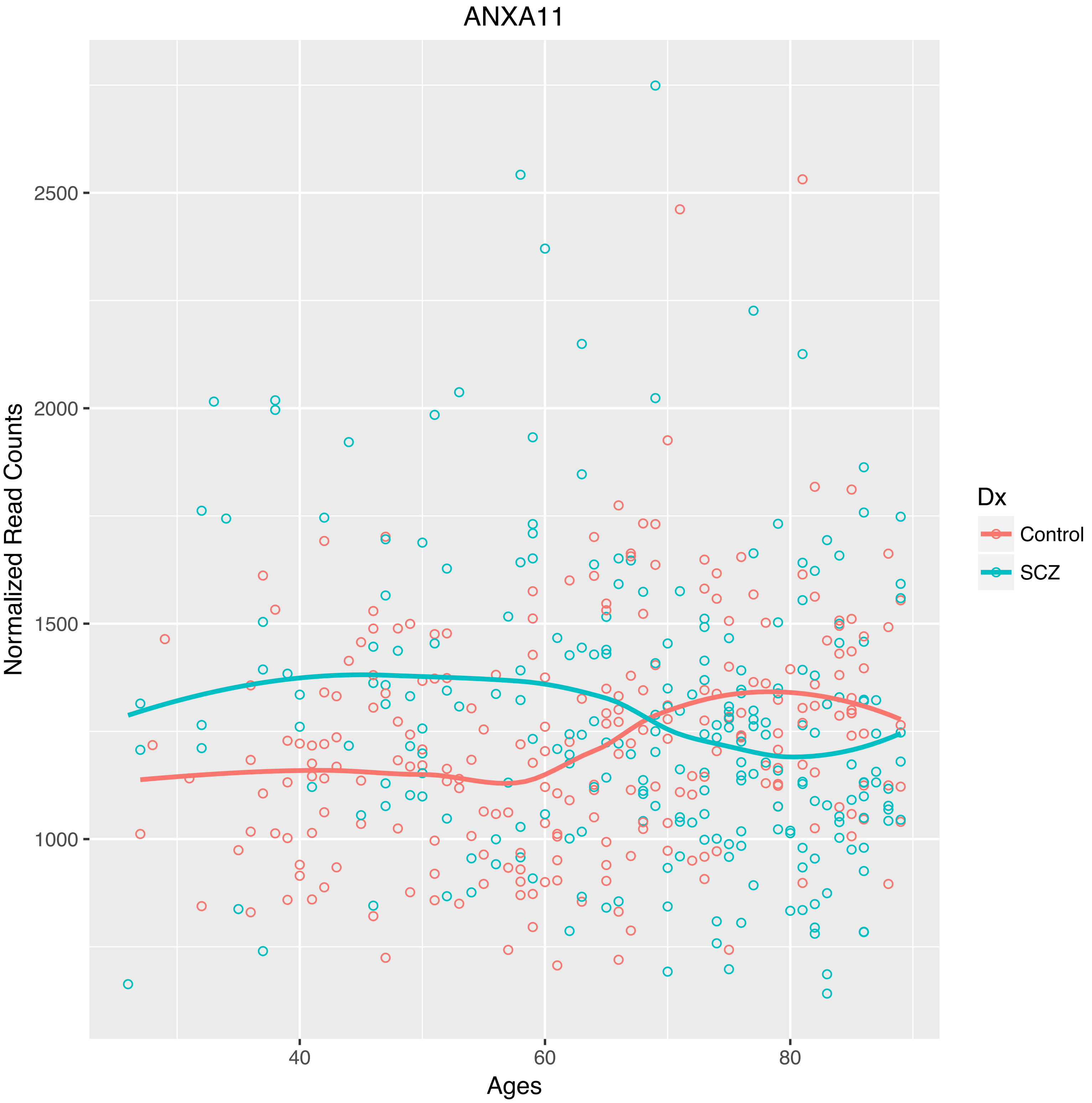

ANXA11
Normalized Read Counts
2500
2000
1500
1000
40
60
80
Ages
Dx
Control
SCZ

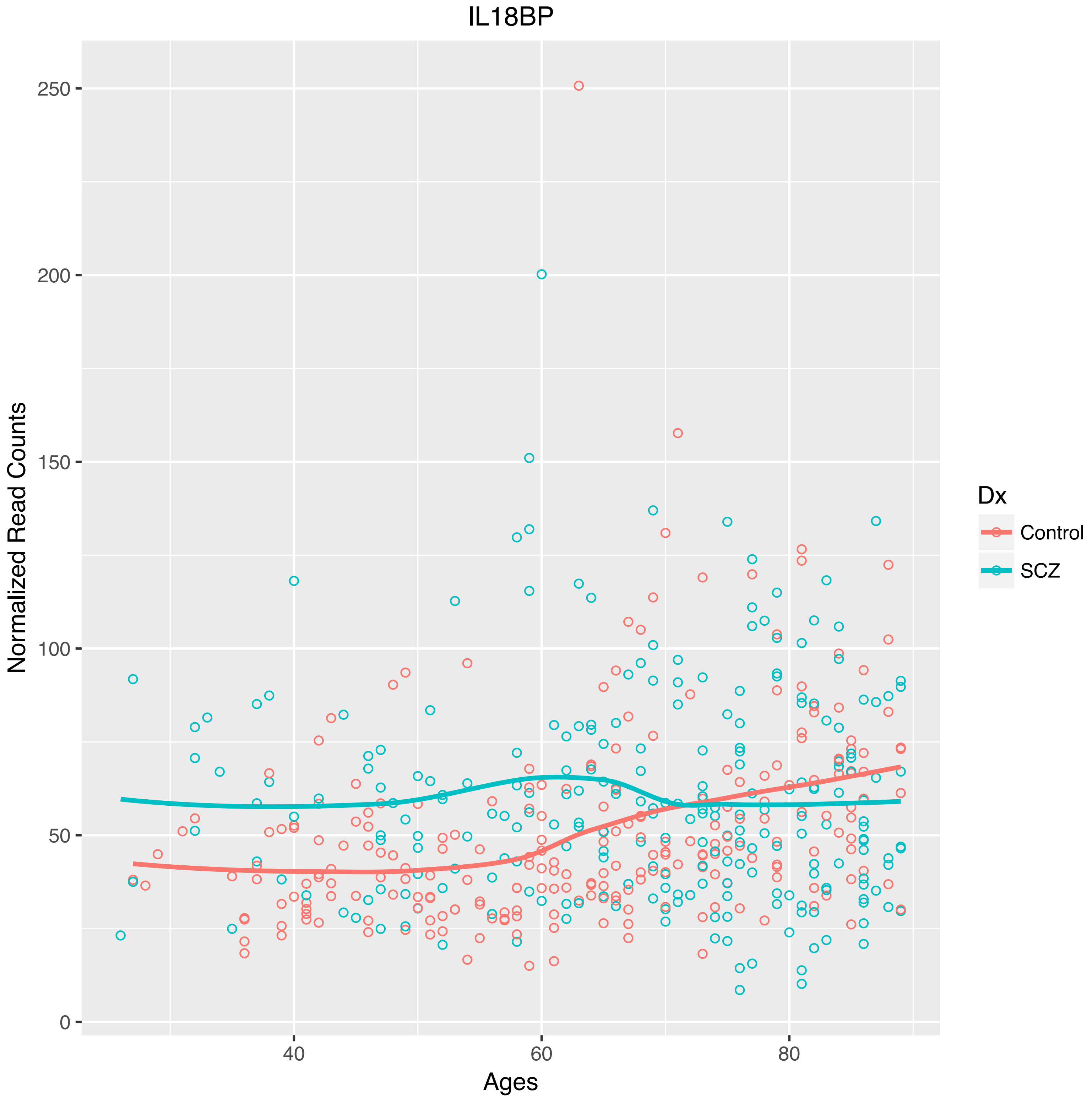

IL18BP
Normalized Read Counts
Ages
0
50
100
150
200
250
40
60
80
Dx
Control
SCZ

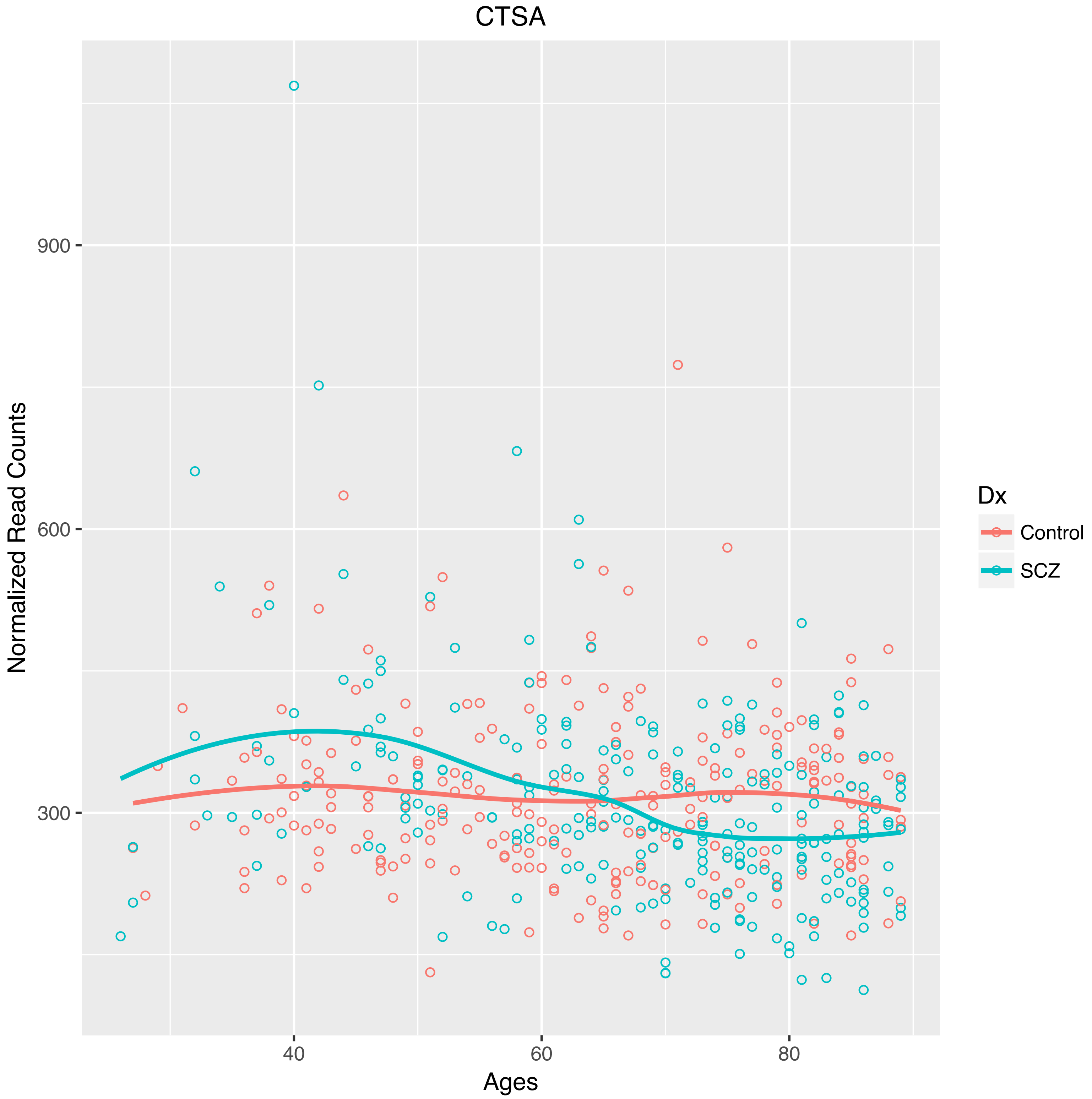

CTSA
Normalized Read Counts
900
600
300
40
60
80
Ages
Dx
Control
SCZ

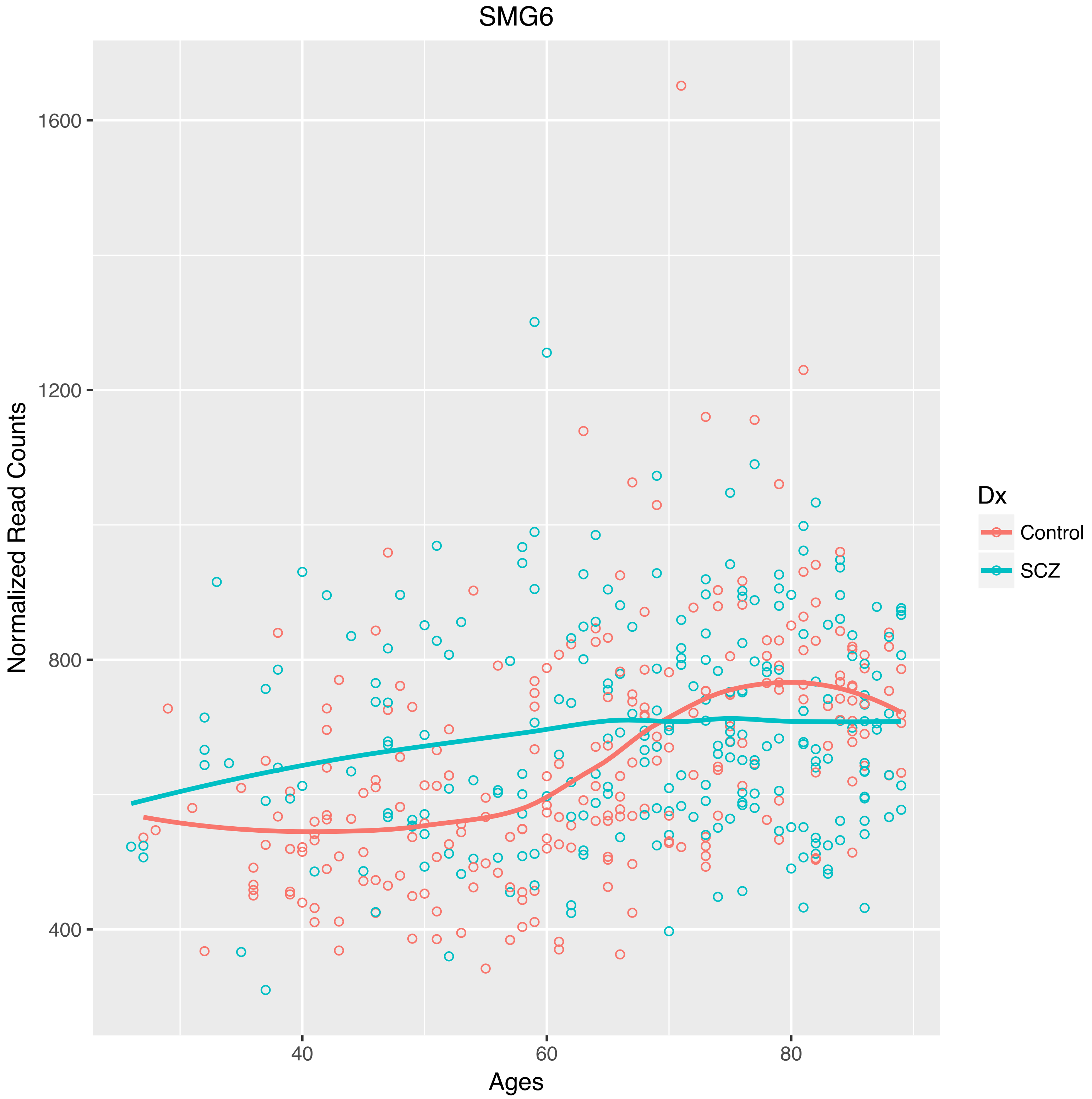

SMG6
Normalized Read Counts
1600
1200
800
400
40
60
80
Ages
Dx
Control
SCZ

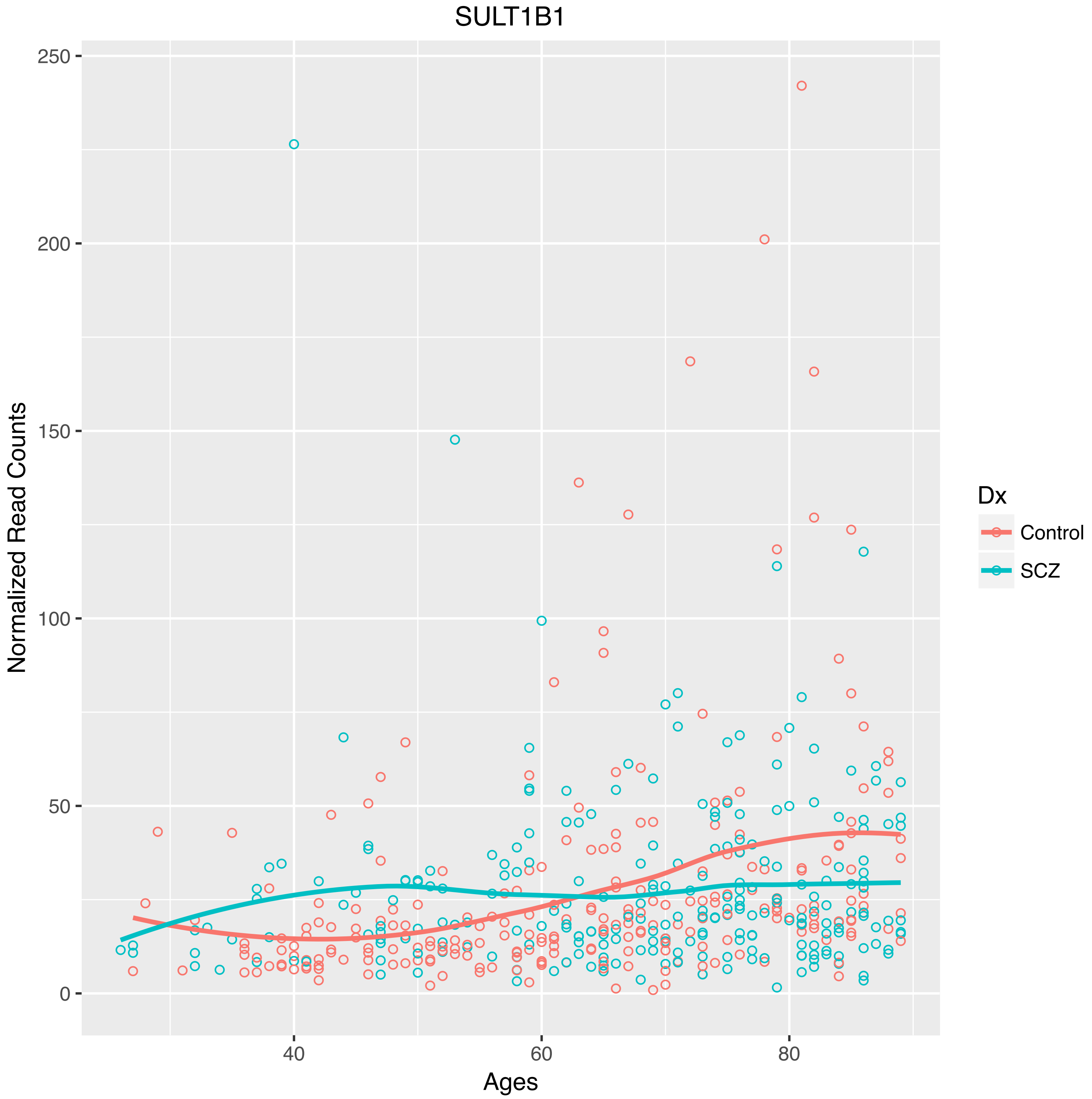

SULT1B1
Normalized Read Counts
250
200
150
100
50
0
40
60
80
Ages
Dx
Control
SCZ

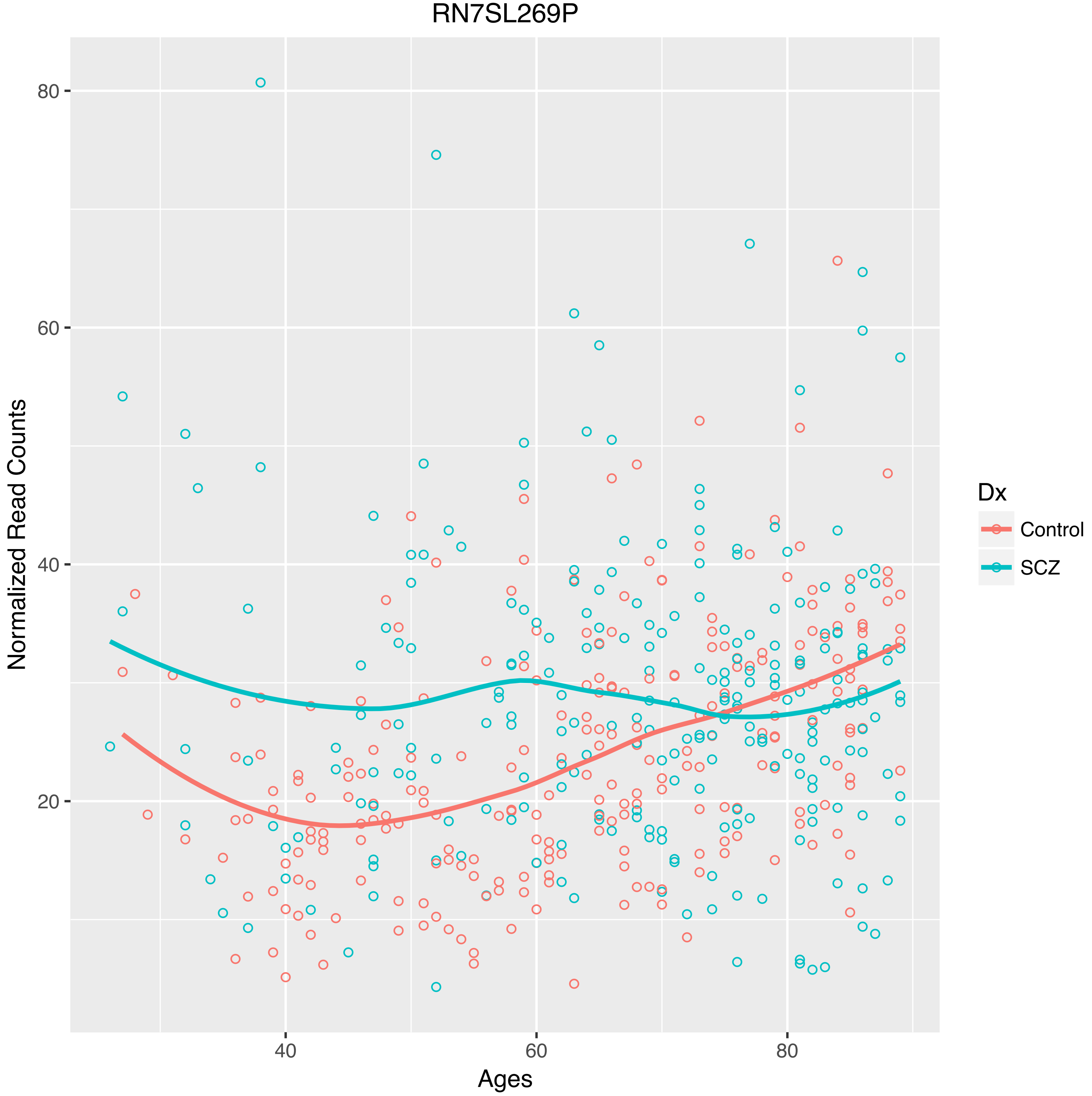
RN7SL269P
Normalized Read Counts
Ages
80
60
40
20
40
60
80
Dx
Control
SCZ

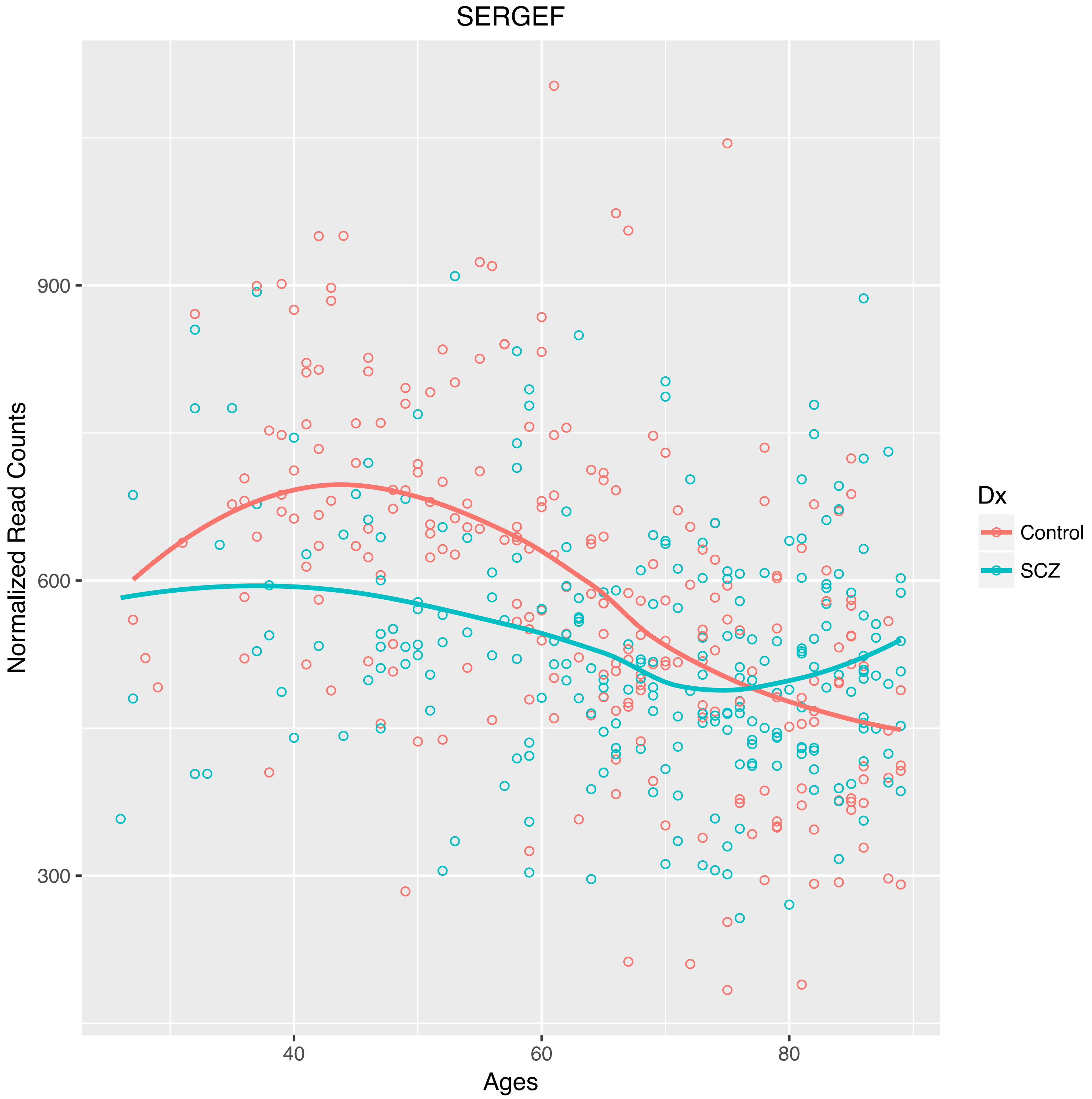
SERGEF
Normalized Read Counts
900
600
300
40
60
80
Ages
Dx
Control
SCZ

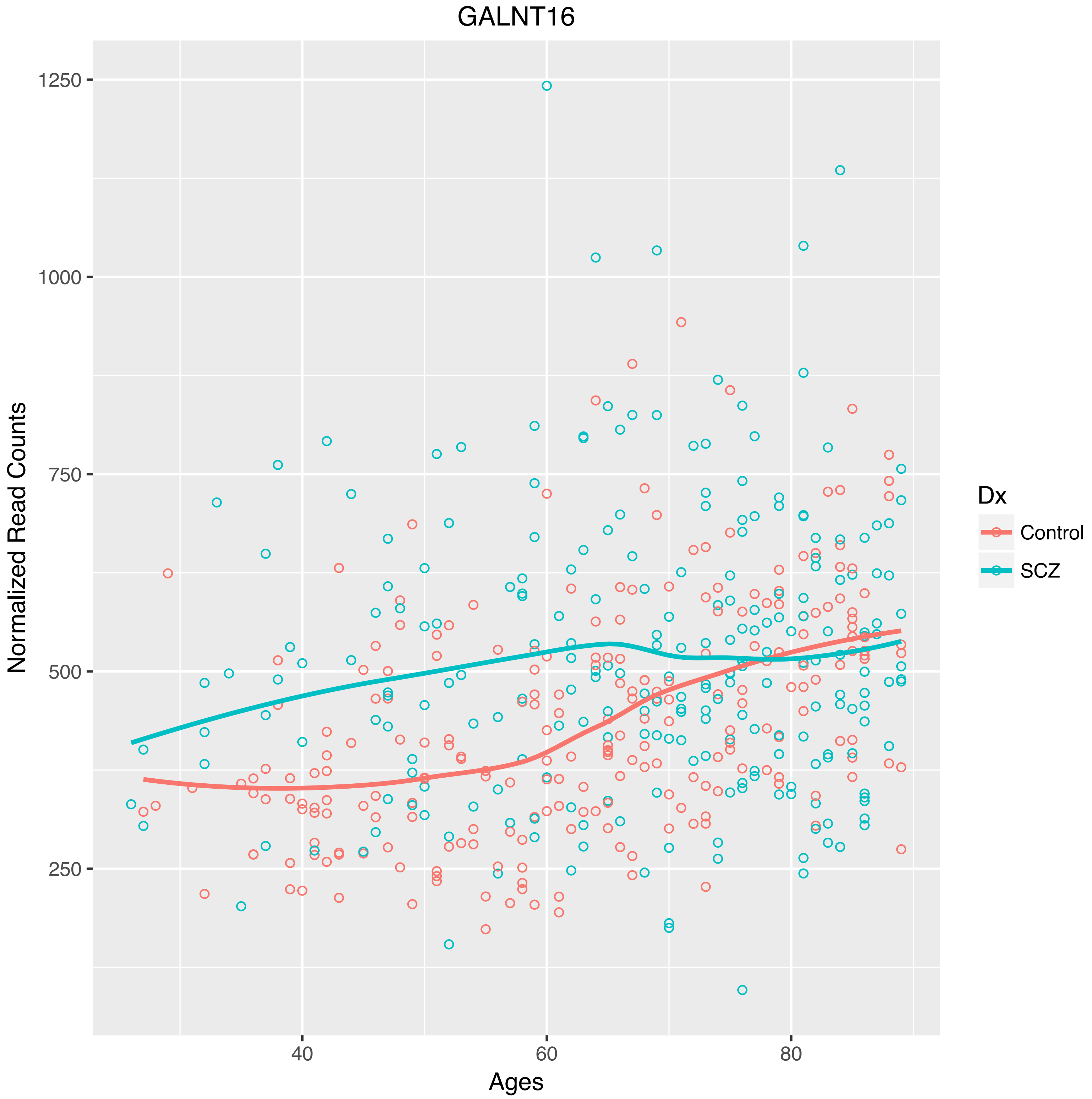

GALNT16
Normalized Read Counts
Ages
1250
1000
750
500
250
40
60
80
Dx
Control
SCZ

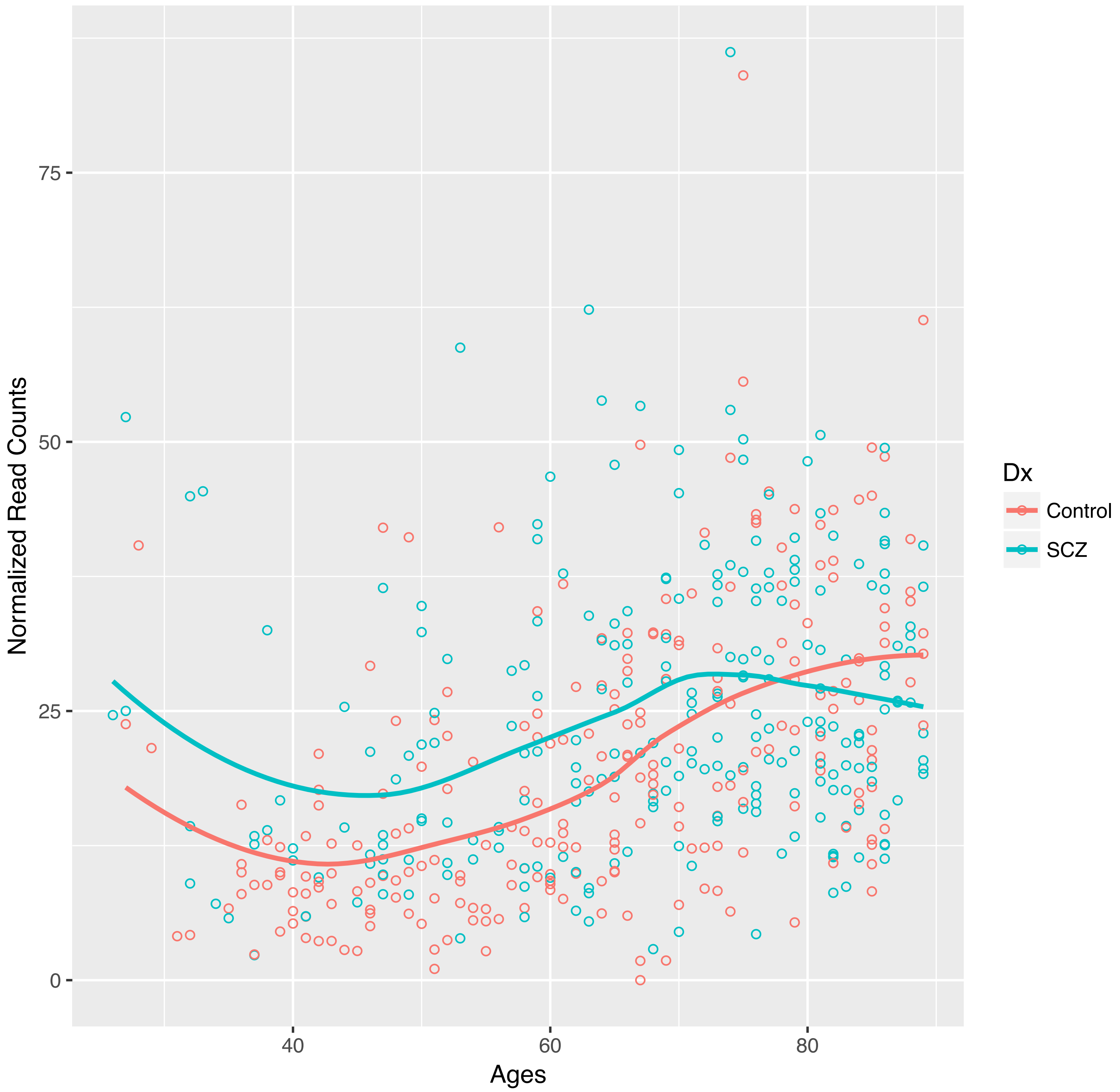

Normalized Read Counts
Ages
Dx
Control
SCZ
75
50
25
0
40
60
80

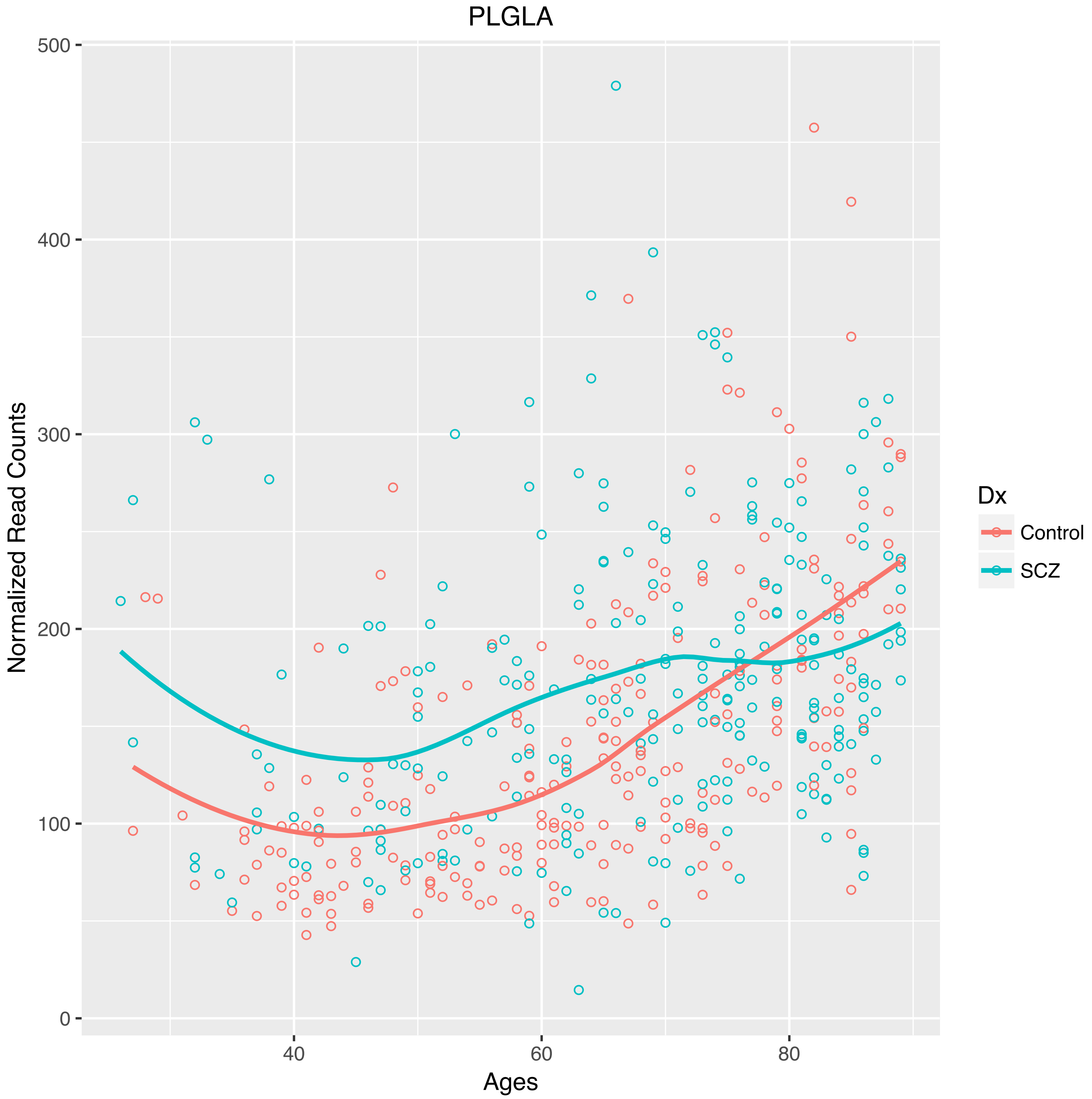

PLGLA
Normalized Read Counts
Ages
0
100
200
300
400
500
40
60
80
Dx
Control
SCZ

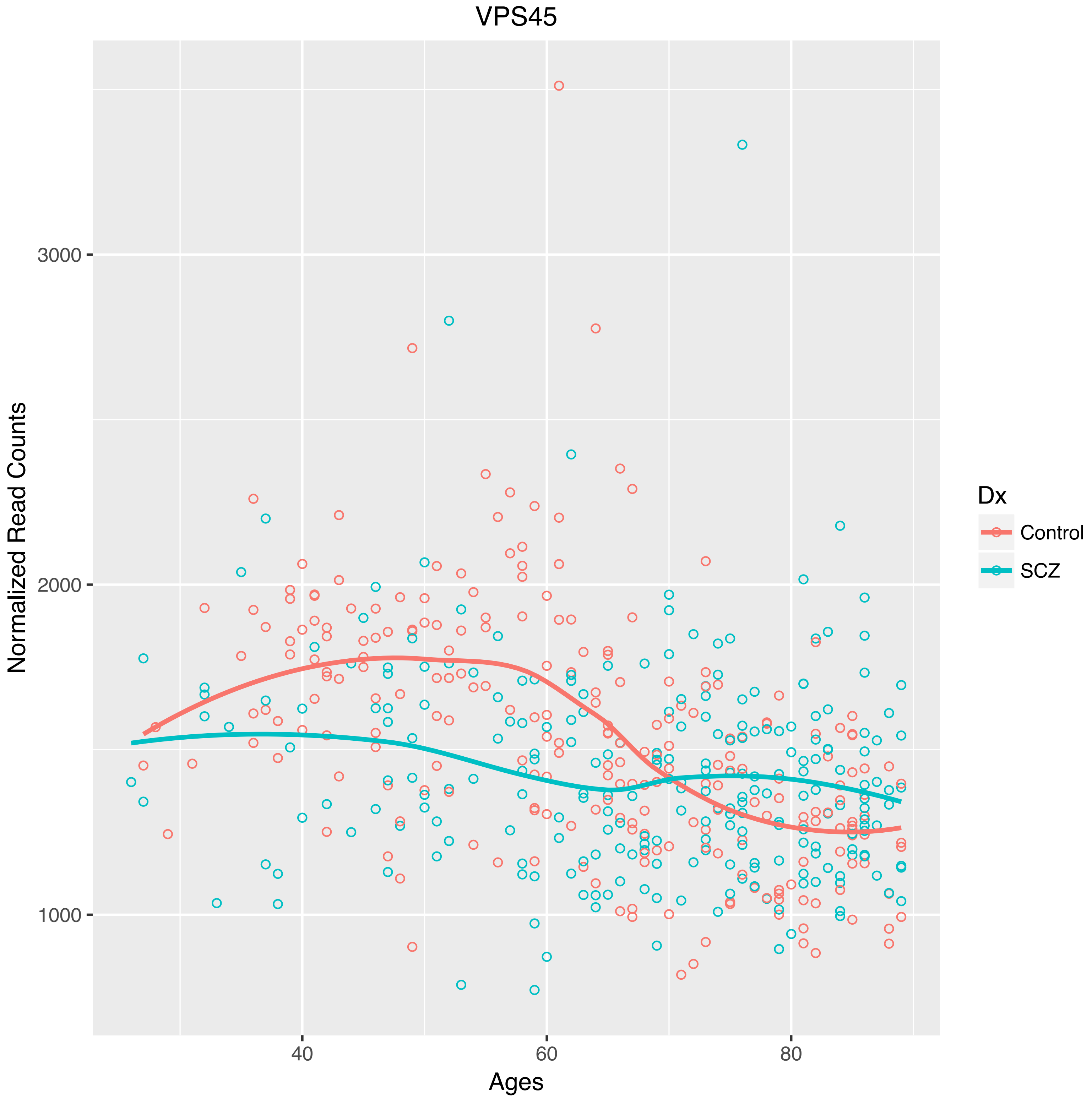

VPS45
Normalized Read Counts
3000
2000
1000
40
60
80
Ages
Dx
Control
SCZ

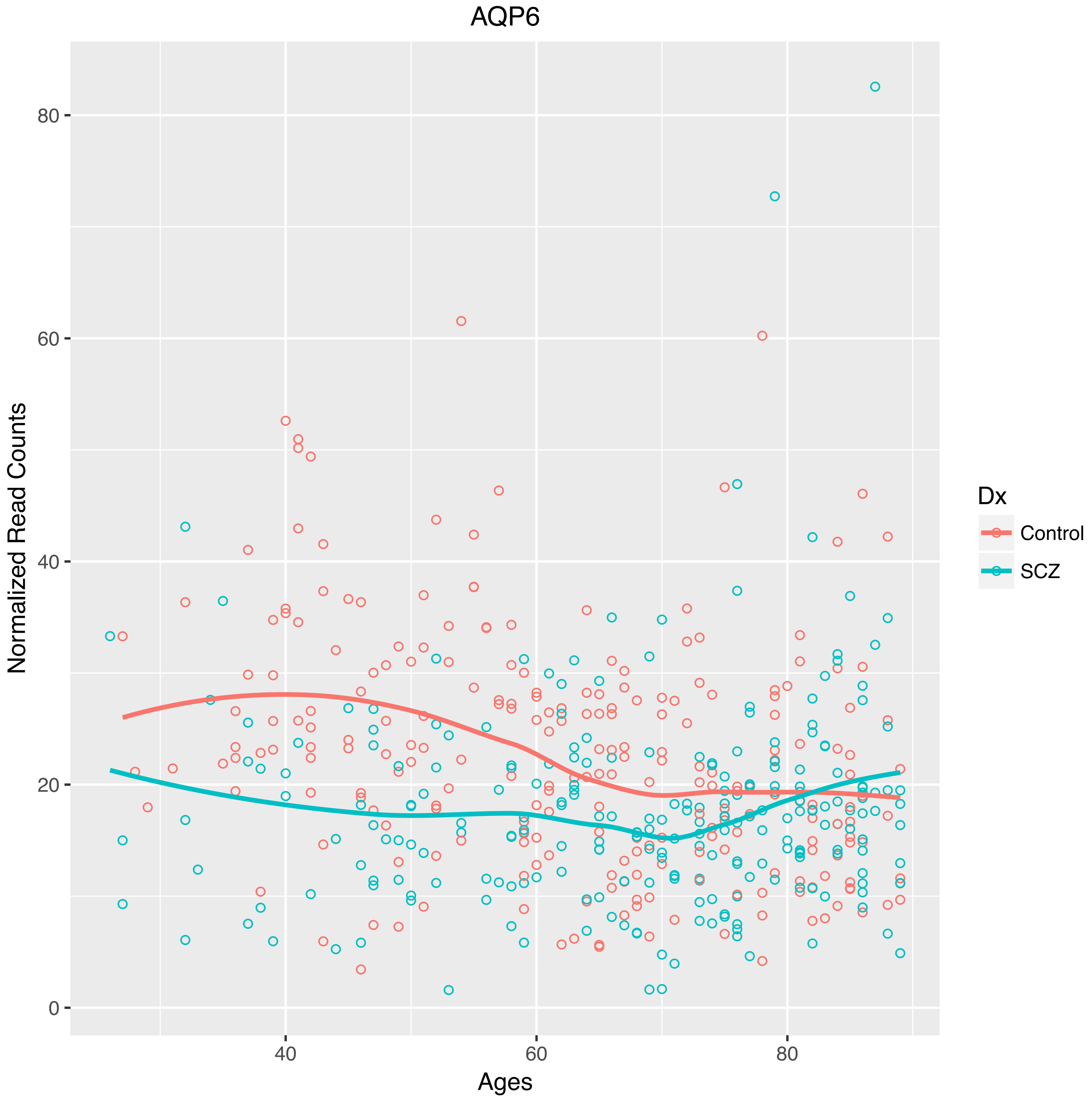
AQP6
Normalized Read Counts
0
20
40
60
80
Ages
40
60
80
Dx
Control
SCZ

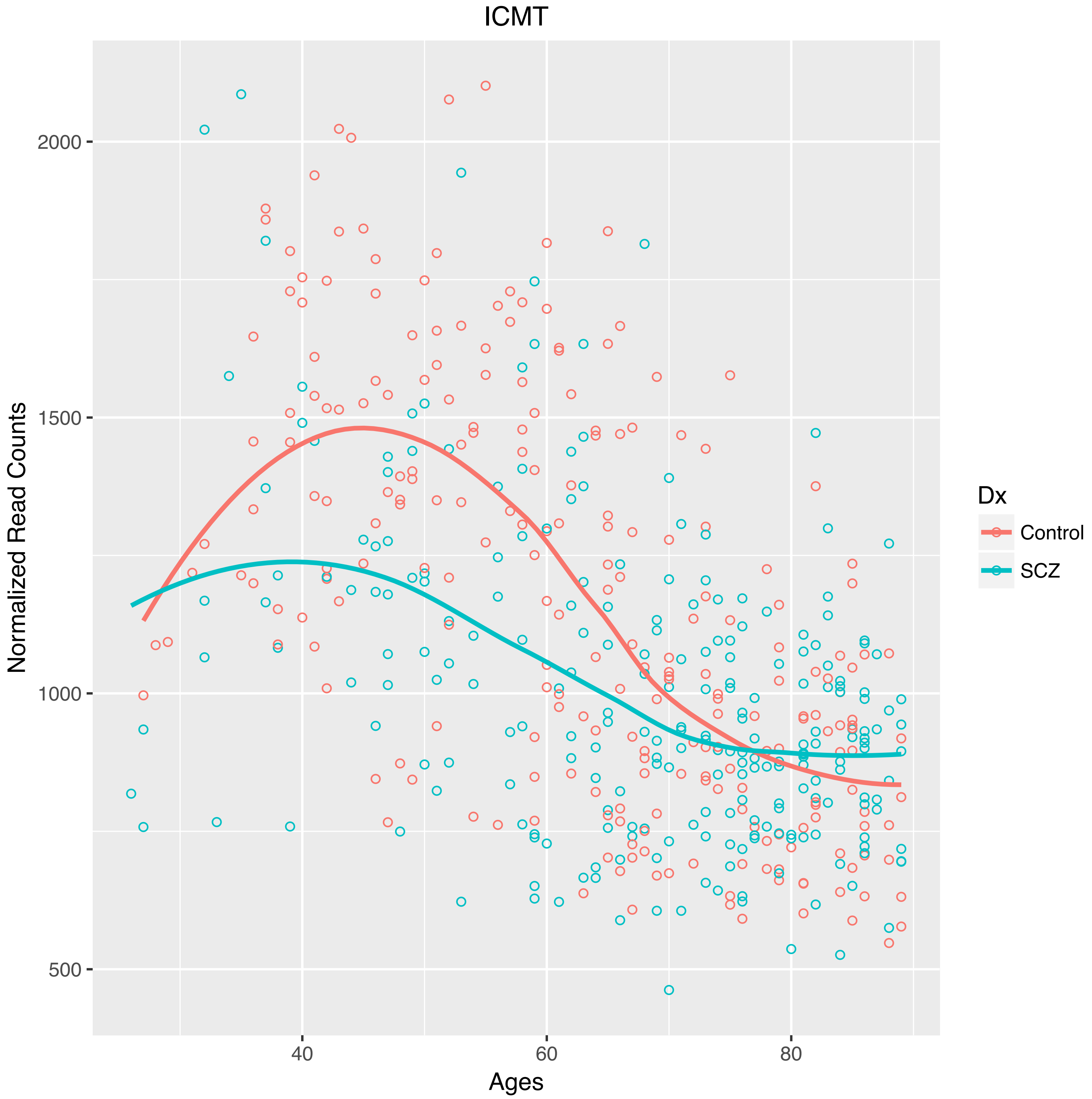
ICMT
Normalized Read Counts
2000
1500
1000
500
40
60
80
Ages
Dx
Control
SCZ

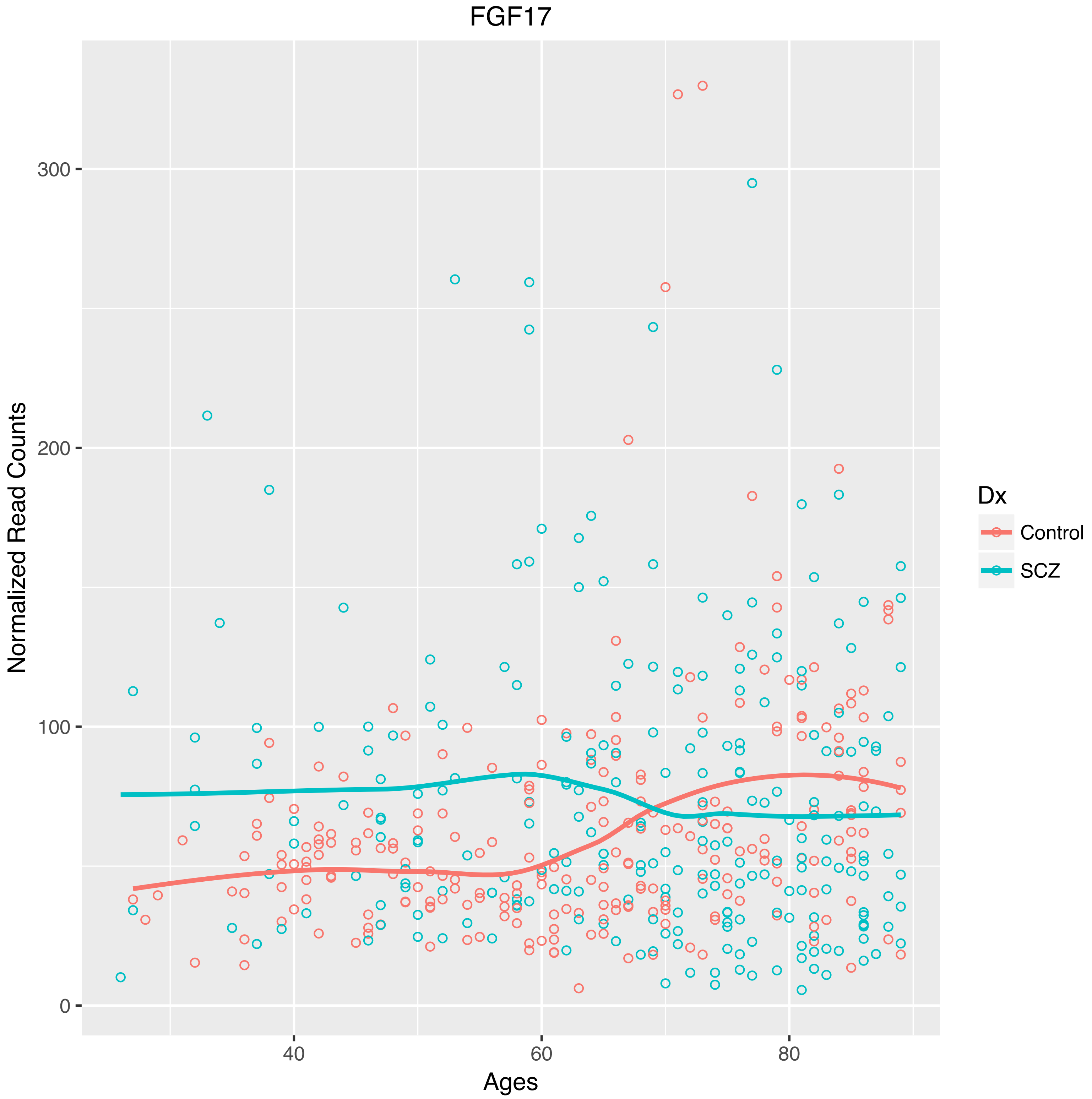
FGF17
Normalized Read Counts
300
200
100
0
40
60
80
Ages
Dx
Control
SCZ

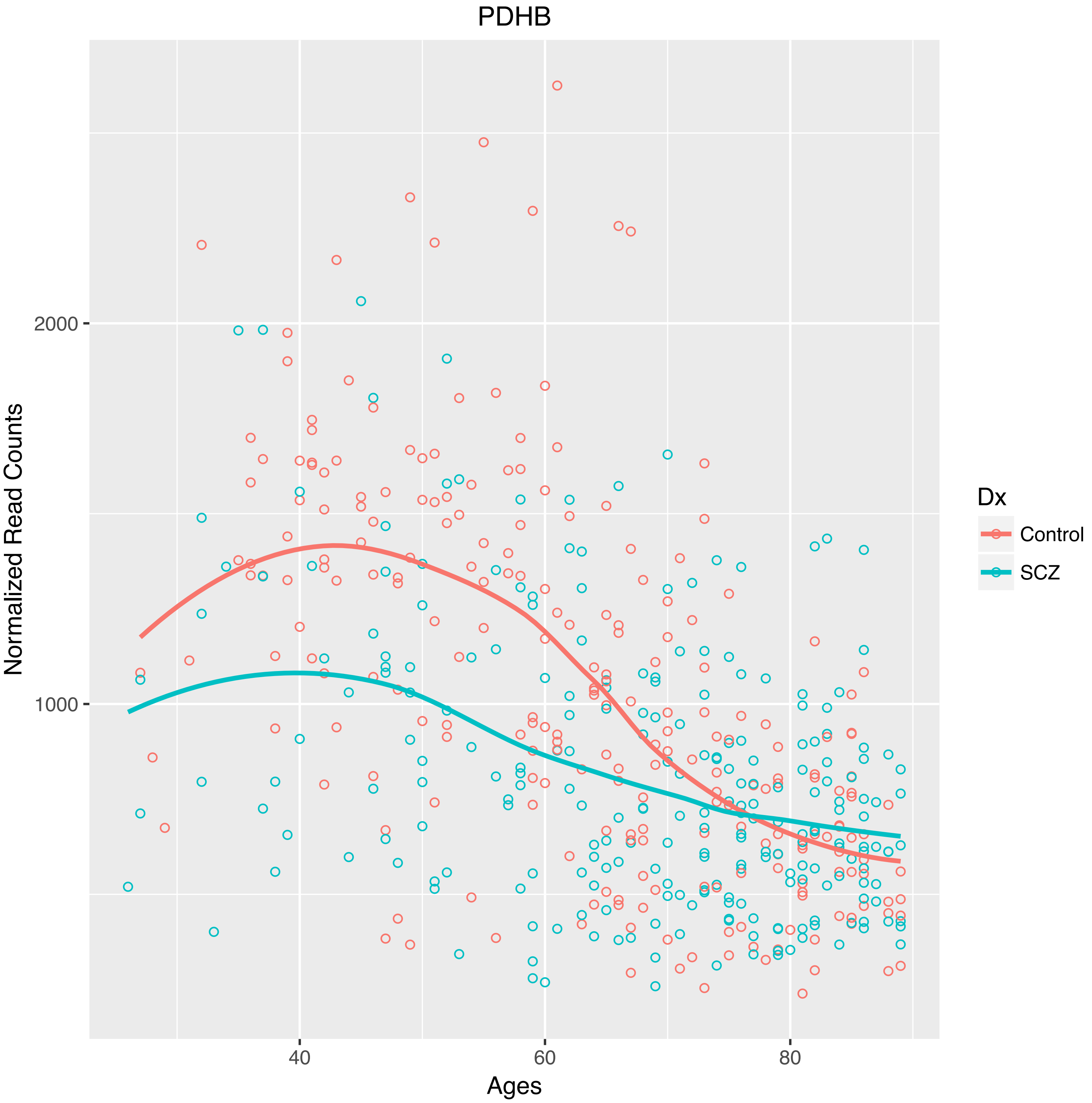
PDHB
Normalized Read Counts
2000
1000
40
60
80
Ages
Dx
Control
SCZ

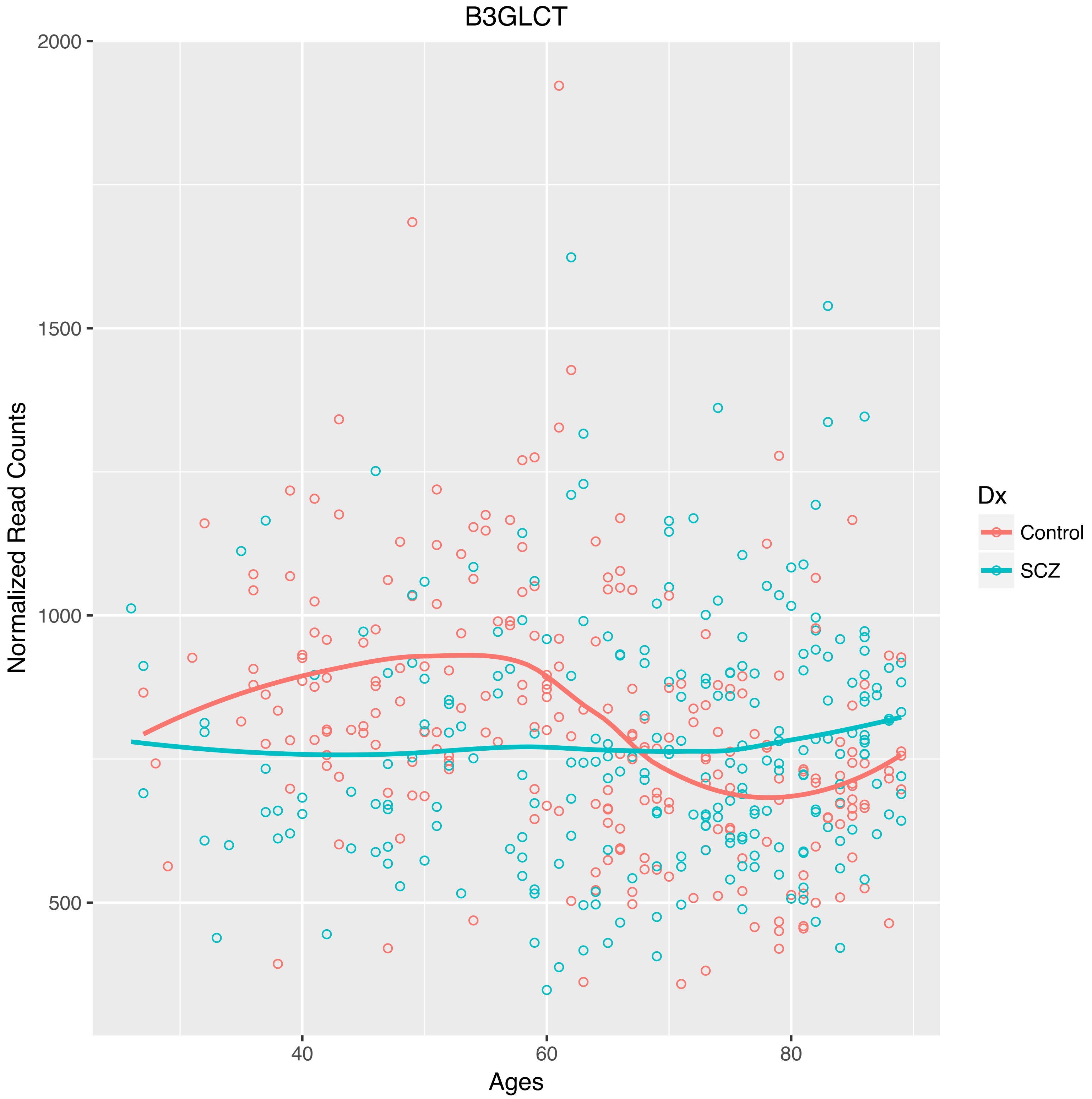

B3GLCT
2000
1500
1000
500
Normalized Read Counts
40
60
80
Ages
Dx
Control
SCZ

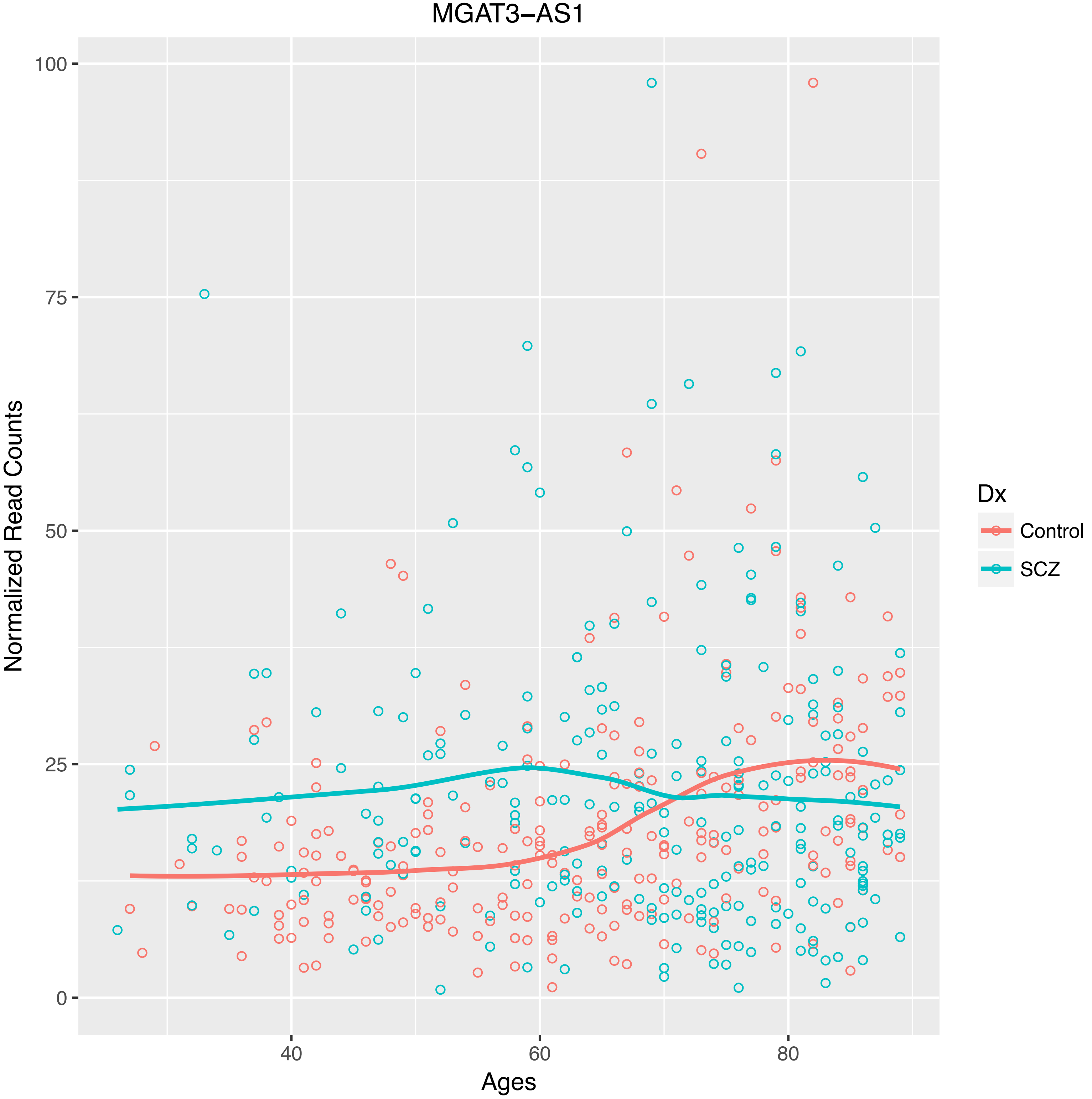
MGAT3-AS1
Normalized Read Counts
100
75
50
25
0
40
60
80
Ages
Dx
Control
SCZ

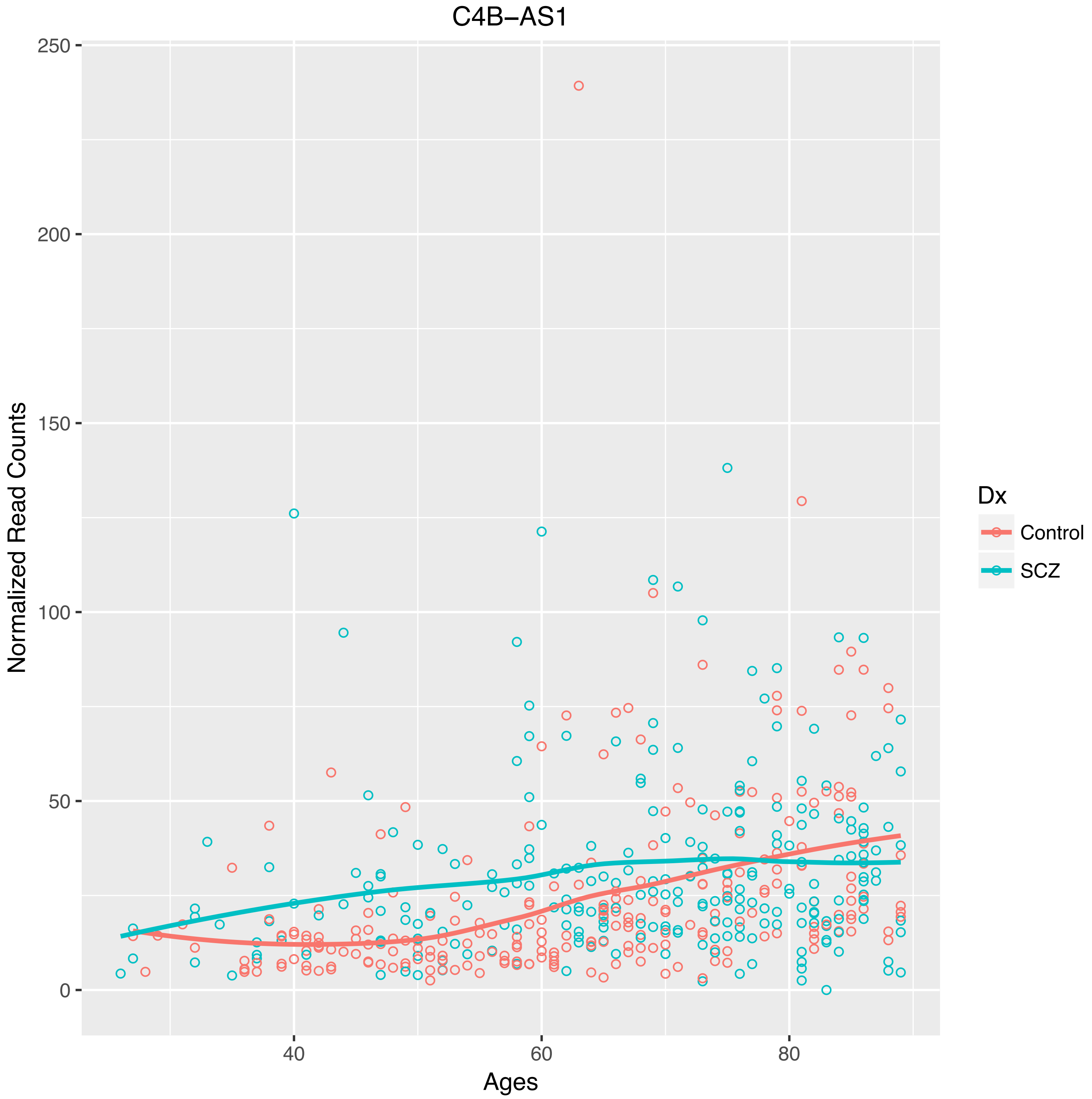
C4B−AS1
Normalized Read Counts
250
200
150
100
50
0
40
60
80
Ages
Dx
Control
SCZ

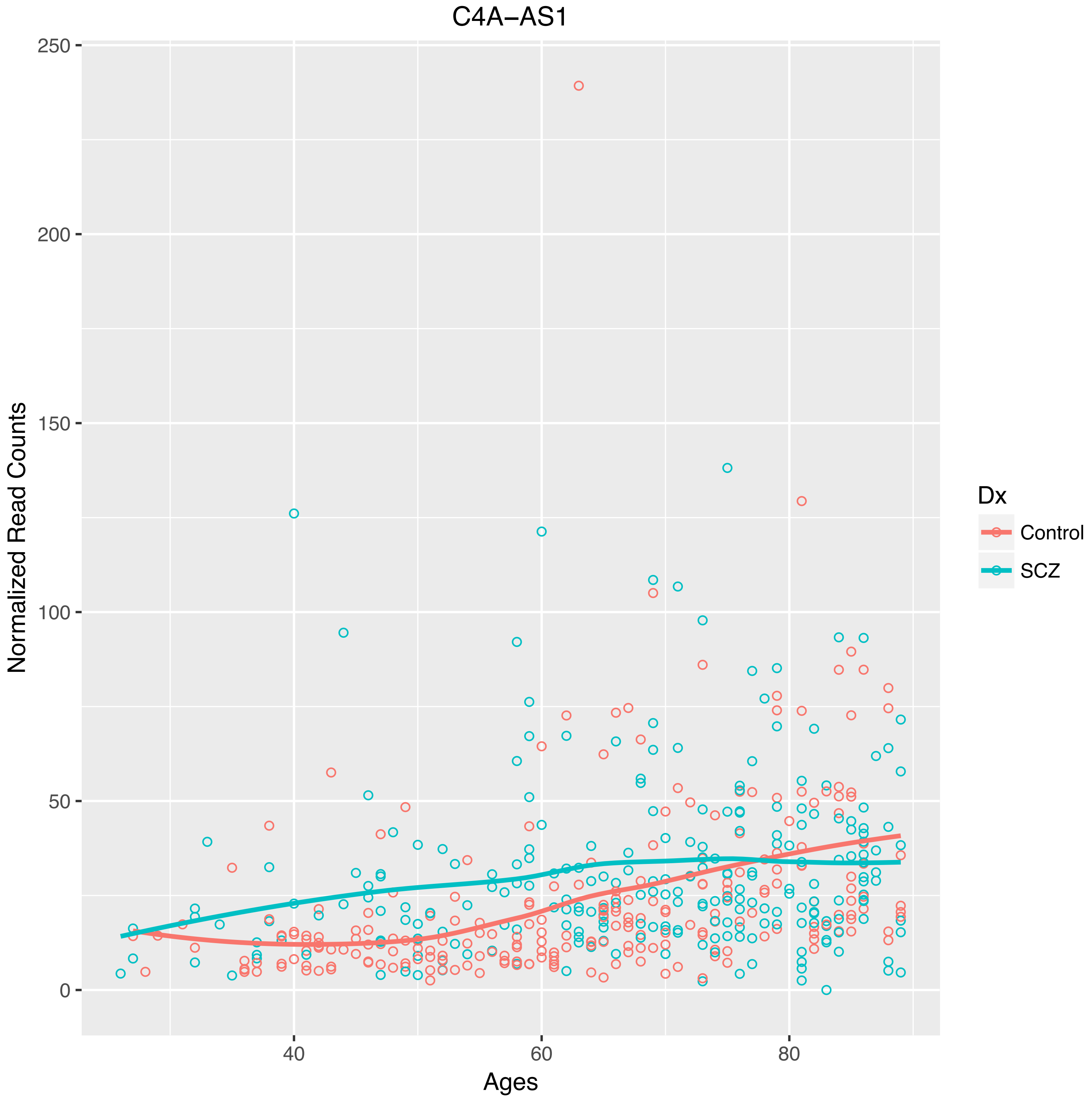

C4A−AS1
Normalized Read Counts
250
200
150
100
50
0
40
60
80
Ages
Dx
Control
SCZ

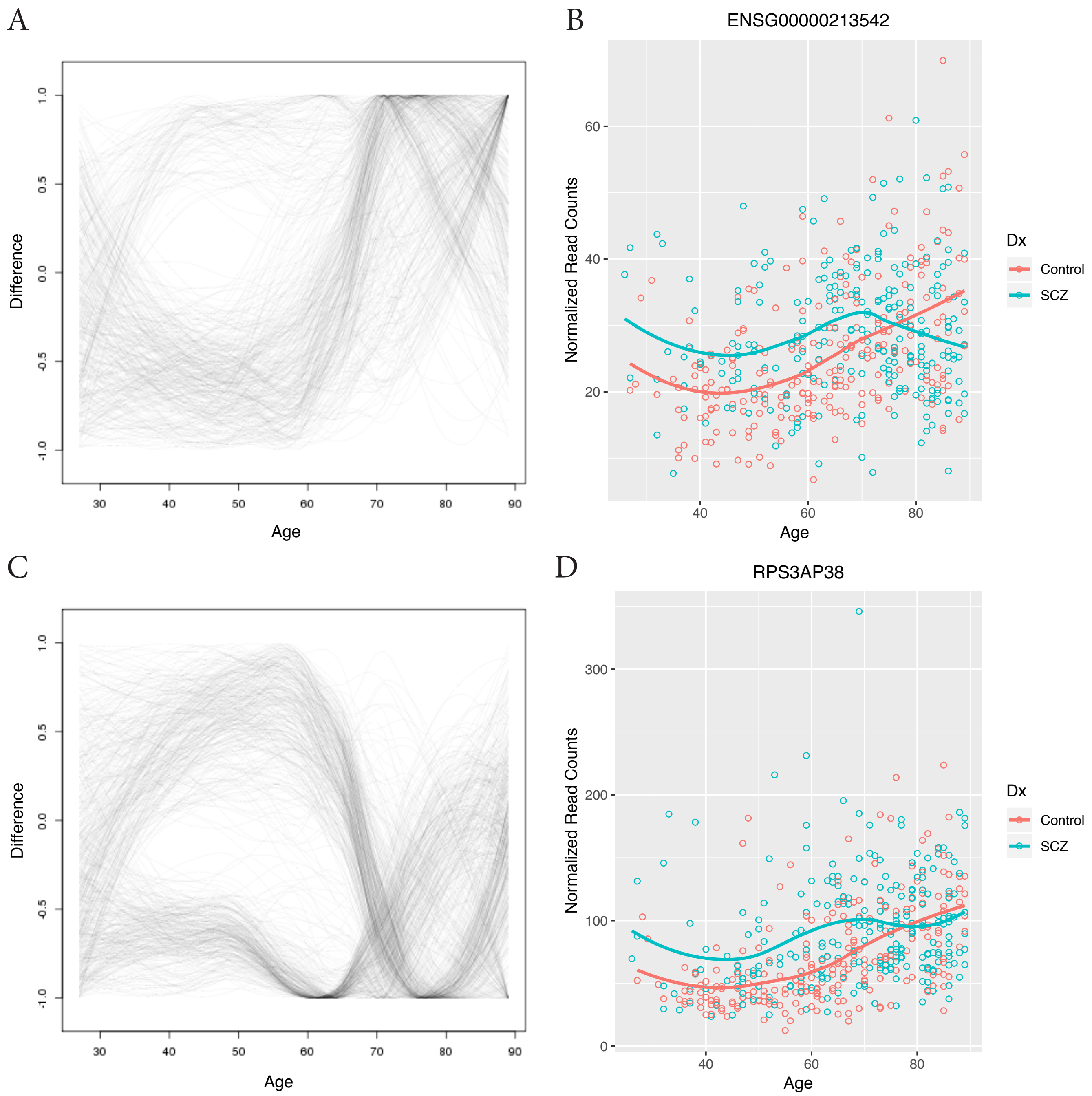

Supplementary Figure2: Gene Expression Differences Between Schizophrenia and Control Brains During Aging The normalized difference in gene expression between schizophrenia and control samples is plotted for ages 25 to 90 for genes identified to be differentially expressed in the CMC (see methods). As the values at leach locus are normalized to the observed maximum difference, only values between 1 and -1 are

possible. In order to avoid over plotting, the loci were grouped into those with **A)** positive maximum difference occurring at age 61 or more (405 loci) and **C)** negative maximum difference occurring at age 61 or more (546 loci). **B)** and **D)** are corresponding representative plots of normalized read counts vs age for individual loci in each category with the lowest *p*-values. The loess function was used to fit the best line for the data points.

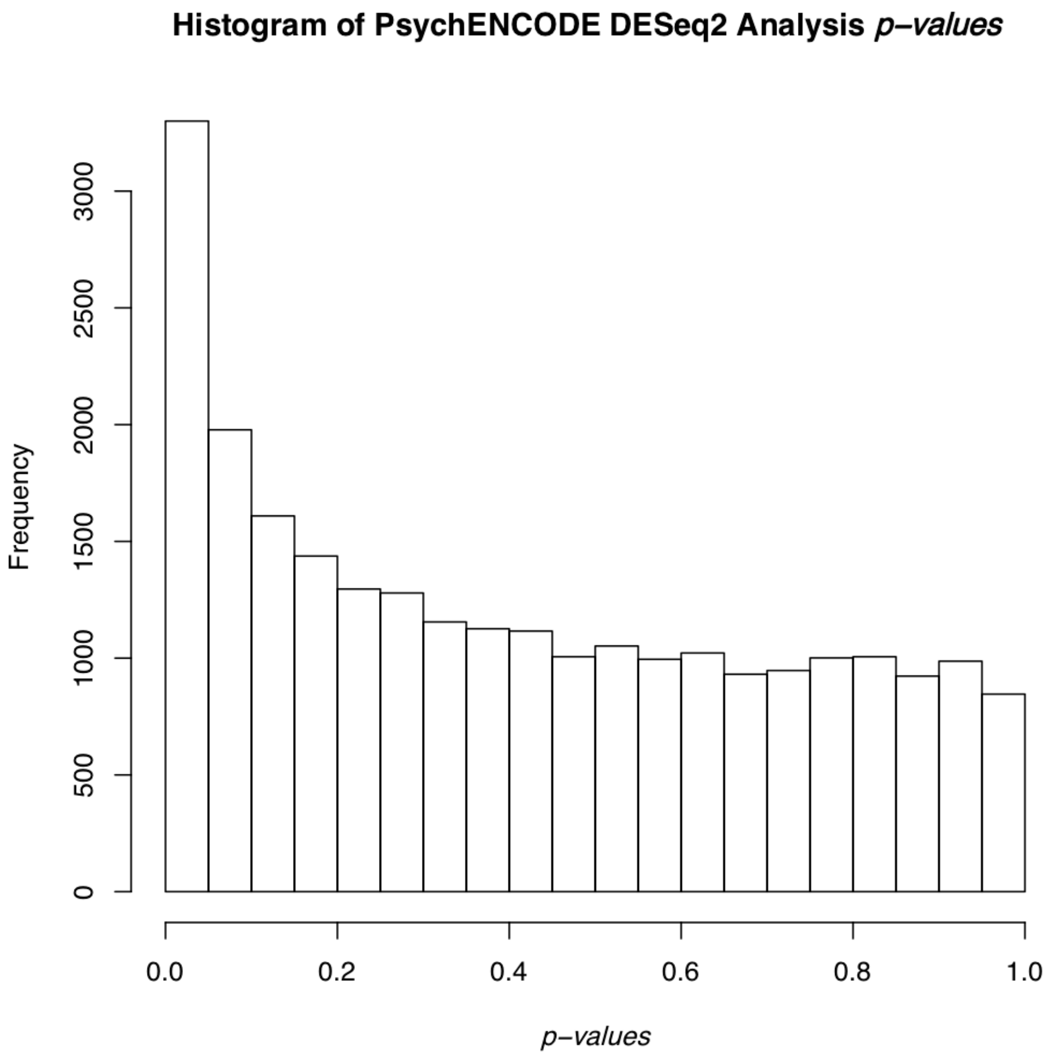

Supplementary Figure 3: Histogram of PsychEncode DESeq2 Analysis *p*-values. All p-values from the differential expression analysis including those that are not significant are plotted.

Supplementary Table 1: Differentially Expressed Genes Common to CMC and PE in the 40 to 60 Age Groups					
	CMC_log2FC	CMC_padj	PE_log2FC	PE_padj	Gene
ENSG00000107821	-0.53	1.25E-05	-0.40	3.43E-04	KAZALD1
ENSG00000103196	0.59	1.06E-04	0.39	1.93E-02	CRISPLD2
ENSG00000229776	0.80	4.38E-04	0.47	2.08E-03	C4B-AS1
ENSG00000233627	0.80	4.38E-04	0.47	2.22E-03	C4A-AS1
ENSG00000101187	0.56	4.63E-04	0.49	1.53E-04	SLCO4A1
ENSG00000168404	0.64	4.63E-04	0.34	3.22E-02	MLKL
ENSG00000101276	0.74	4.68E-04	0.84	1.14E-05	SLC52A3
ENSG00000047617	0.38	5.86E-04	0.37	1.20E-03	ANO2
ENSG00000122863	0.46	8.00E-04	0.50	2.07E-05	CHST3
ENSG00000106648	-0.33	1.92E-03	-0.33	1.12E-02	GALNTL5
ENSG00000096060	0.67	2.50E-03	1.07	5.71E-08	FKBP5
ENSG00000213700	0.48	2.50E-03	0.29	7.82E-02	RPL17P50
ENSG00000167700	-0.30	2.64E-03	-0.18	2.71E-02	MFSD3
ENSG00000077238	0.70	2.70E-03	0.76	4.54E-06	IL4R
ENSG00000197021	-0.26	2.89E-03	-0.11	6.30E-02	CXorf40B
ENSG00000151718	0.13	3.51E-03	0.11	5.86E-03	WWC2
ENSG00000110799	0.54	3.53E-03	0.46	2.81E-04	VWF
ENSG00000197142	0.41	3.53E-03	0.45	3.34E-06	ACSL5
ENSG00000197345	-0.38	3.64E-03	-0.18	9.17E-02	MRPL21
ENSG00000183943	0.31	3.67E-03	0.36	1.82E-04	PRKX
ENSG00000108641	-0.22	4.01E-03	-0.11	7.96E-02	B9D1
ENSG00000141485	-0.43	4.01E-03	-0.27	2.18E-02	SLC13A5
ENSG00000157617	0.22	4.01E-03	0.21	3.05E-05	C2CD2
ENSG00000182916	-0.33	4.01E-03	-0.26	9.89E-04	TCEAL7
ENSG00000248455	-0.42	4.01E-03	-0.37	2.22E-03	LINC02217
ENSG00000272449	-0.42	4.01E-03	-0.33	3.83E-02	
ENSG00000174348	0.48	4.08E-03	0.27	3.48E-02	PODN
ENSG00000162929	-0.15	4.21E-03	-0.07	9.35E-02	KIAA1841
ENSG00000261716	0.33	4.55E-03	0.26	1.02E-03	
ENSG00000163879	0.25	4.62E-03	0.20	2.17E-02	DNALI1
ENSG00000241158	0.47	4.67E-03	0.54	3.50E-03	ADAMTS9-AS1
ENSG00000267270	0.38	4.68E-03	0.20	7.26E-02	PARD6G-AS1
ENSG00000249436	-0.41	5.20E-03	-0.30	5.47E-03	
ENSG00000171509	-0.31	5.20E-03	-0.19	2.20E-02	RXFP1
ENSG00000087087	0.22	5.23E-03	-0.12	2.84E-02	SRRT
ENSG00000164112	-0.33	5.23E-03	-0.24	3.24E-03	TMEM155

ENSG00000164258	-0.32	5.23E-03	-0.20	4.33E-02	NDUFS4
ENSG00000255916	-0.32	5.23E-03	-0.25	2.62E-03	
ENSG00000178607	0.19	5.31E-03	0.09	2.71E-02	ERN1
ENSG00000137507	0.52	5.45E-03	0.37	2.08E-02	LRRC32
ENSG00000241218	0.46	5.47E-03	0.22	7.02E-02	
ENSG00000161281	-0.50	5.87E-03	-0.38	2.84E-02	COX7A1
ENSG00000080573	-0.45	5.89E-03	-0.43	1.32E-04	COL5A3
ENSG00000127507	0.66	5.89E-03	0.44	6.23E-04	ADGRE2
ENSG00000073060	0.16	5.97E-03	-0.10	6.84E-02	SCARB1
ENSG00000261455	-0.43	5.97E-03	-0.22	4.61E-02	LINC01003
ENSG00000179776	0.42	6.18E-03	0.24	8.68E-02	CDH5
ENSG00000250582	0.50	6.18E-03	0.30	3.00E-02	SMAD1-AS2
ENSG00000130052	0.28	6.24E-03	0.27	1.57E-02	STARD8
ENSG00000162241	-0.24	6.71E-03	-0.21	2.21E-03	SLC25A45
ENSG00000249717	0.51	7.45E-03	0.25	4.20E-03	
ENSG00000001617	0.44	8.14E-03	0.58	1.31E-05	SEMA3F
ENSG00000158792	-0.35	8.36E-03	-0.44	1.09E-04	SPATA2L
ENSG00000115604	0.90	8.36E-03	1.06	1.79E-03	IL18R1
ENSG00000077150	0.46	8.78E-03	0.29	4.26E-02	NFKB2
ENSG00000103253	-0.30	8.89E-03	-0.22	1.08E-02	HAGHL
ENSG00000236841	-0.37	8.89E-03	-0.28	6.16E-02	
ENSG00000271824	-0.52	9.06E-03	-0.46	2.22E-03	SMIM32
ENSG00000105227	0.36	9.19E-03	0.32	1.42E-02	PRX
ENSG00000136297	-0.50	9.19E-03	-0.52	6.09E-06	MMD2
ENSG00000197620	-0.25	9.19E-03	-0.14	3.75E-02	CXorf40A
ENSG00000171502	-0.31	9.26E-03	-0.36	1.47E-04	COL24A1
ENSG00000184887	-0.21	9.52E-03	-0.16	2.06E-03	BTBD6
ENSG00000136011	0.49	9.78E-03	0.45	3.07E-03	STAB2
ENSG00000143198	-0.24	9.93E-03	-0.15	7.39E-02	MGST3
ENSG00000179774	-0.36	9.93E-03	-0.36	7.39E-03	ATOH7
ENSG00000100626	0.24	1.08E-02	0.20	7.26E-04	GALNT16
ENSG00000157778	-0.29	1.08E-02	-0.25	5.77E-04	PSMG3
ENSG00000115226	-0.22	1.10E-02	-0.21	1.51E-03	FNDC4
ENSG00000196465	-0.25	1.10E-02	-0.20	5.11E-04	MYL6B
ENSG00000103254	-0.40	1.10E-02	-0.20	5.01E-02	FAM173A
ENSG00000144635	-0.17	1.10E-02	-0.15	3.96E-04	DYNC1LI1
ENSG00000133131	0.19	1.11E-02	0.17	3.88E-02	MORC4
ENSG00000173566	-0.31	1.12E-02	-0.29	8.45E-04	NA
ENSG00000086506	-0.40	1.13E-02	-0.45	1.51E-02	HBQ1

ENSG00000089159	0.28	1.13E-02	0.15	7.36E-02	PXN
ENSG00000167515	-0.20	1.14E-02	-0.17	1.91E-02	TRAPPC2L
ENSG00000224431	0.49	1.20E-02	0.39	9.90E-03	
ENSG00000101222	-0.35	1.21E-02	-0.31	2.15E-04	SPEF1
ENSG00000182327	-0.33	1.21E-02	-0.23	7.32E-02	GLTPD2
ENSG00000065057	-0.25	1.21E-02	-0.15	5.45E-02	NTHL1
ENSG00000213753	-0.12	1.22E-02	-0.09	5.97E-02	CENPBD1P1
ENSG00000223401	0.60	1.23E-02	0.39	6.72E-02	NA
ENSG00000115073	-0.25	1.24E-02	-0.11	5.99E-02	ACTR1B
ENSG00000188690	-0.22	1.26E-02	-0.19	5.33E-03	UROS
ENSG00000059728	0.17	1.29E-02	0.10	5.34E-02	MXD1
ENSG00000121060	0.23	1.29E-02	0.20	2.35E-03	TRIM25
ENSG00000185608	-0.19	1.31E-02	-0.13	8.35E-02	MRPL40
ENSG00000102547	-0.17	1.37E-02	-0.12	2.44E-02	CAB39L
ENSG00000115361	0.23	1.41E-02	0.36	1.91E-04	ACADL
ENSG00000263874	-0.24	1.41E-02	-0.18	6.80E-03	LINC00672
ENSG00000173542	0.16	1.44E-02	0.16	2.84E-03	MOB1B
ENSG00000270300	0.33	1.45E-02	0.21	2.17E-02	PHACTR2P1
ENSG00000116237	-0.15	1.46E-02	-0.17	2.24E-04	ICMT
ENSG00000172465	-0.22	1.46E-02	-0.16	1.01E-02	TCEAL1
ENSG00000115514	-0.22	1.50E-02	-0.12	3.23E-02	TXNDC9
ENSG00000231752	-0.22	1.51E-02	-0.18	2.74E-02	EMBP1
ENSG00000103319	0.18	1.52E-02	0.22	6.49E-04	EEF2K
ENSG00000162302	-0.19	1.53E-02	-0.20	2.67E-03	RPS6KA4
ENSG00000105419	-0.23	1.65E-02	-0.20	2.64E-02	MEIS3
ENSG00000130589	0.40	1.65E-02	0.34	3.35E-02	HELZ2
ENSG00000172059	0.22	1.65E-02	0.21	1.50E-02	KLF11
ENSG00000138741	-0.24	1.67E-02	-0.24	6.69E-03	TRPC3
ENSG00000090266	-0.32	1.71E-02	-0.22	1.90E-02	NDUFB2
ENSG00000105393	-0.26	1.71E-02	-0.13	9.52E-03	BABAM1
ENSG00000164604	-0.18	1.77E-02	-0.24	3.98E-04	GPR85
ENSG00000035687	-0.18	1.77E-02	-0.11	6.04E-02	ADSS
ENSG00000074527	-0.20	1.77E-02	-0.20	1.27E-02	NTN4
ENSG00000166681	-0.27	1.78E-02	-0.18	4.72E-02	BEX3
ENSG00000100804	-0.24	1.80E-02	-0.18	1.85E-02	PSMB5
ENSG00000113083	0.23	1.80E-02	0.25	8.12E-04	LOX
ENSG00000224389	0.58	1.81E-02	0.32	7.71E-02	C4B
ENSG00000183864	0.23	1.81E-02	0.12	7.16E-02	TOB2
ENSG00000066697	-0.17	1.82E-02	-0.12	2.24E-02	MSANTD3

ENSG00000122884	0.27	1.82E-02	0.16	8.78E-02	P4HA1
ENSG00000178741	-0.22	1.82E-02	-0.18	4.55E-02	COX5A
ENSG00000074071	-0.23	1.82E-02	-0.14	8.61E-02	MRPS34
ENSG00000109906	0.19	1.82E-02	0.24	9.45E-03	ZBTB16
ENSG00000150457	0.30	1.82E-02	0.22	3.50E-02	LATS2
ENSG00000158806	-0.23	1.82E-02	-0.18	1.37E-06	NPM2
ENSG00000163832	-0.18	1.82E-02	-0.18	1.54E-04	ELP6
ENSG00000168477	0.21	1.82E-02	0.19	7.16E-02	TNXB
ENSG00000114423	0.17	1.83E-02	0.14	5.65E-04	CBLB
ENSG00000242173	-0.27	1.83E-02	-0.21	2.62E-02	ARHGDIG
ENSG00000100379	-0.19	1.84E-02	-0.20	6.92E-04	KCTD17
ENSG00000134375	-0.24	1.84E-02	-0.17	7.14E-02	TIMM17A
ENSG00000170745	-0.27	1.85E-02	-0.31	2.30E-04	KCNS3
ENSG00000188051	-0.30	1.87E-02	-0.26	1.29E-03	TMEM221
ENSG00000124205	-0.37	1.87E-02	-0.45	2.95E-04	EDN3
ENSG00000086504	-0.23	1.87E-02	-0.22	2.67E-03	MRPL28
ENSG00000184207	-0.22	1.87E-02	-0.12	5.46E-02	PGP
ENSG00000205704	-0.28	1.87E-02	-0.21	3.30E-02	LINC00634
ENSG00000214253	-0.25	1.87E-02	-0.26	3.91E-03	FIS1
ENSG00000090971	-0.25	1.88E-02	-0.14	5.20E-02	NAT14
ENSG00000174939	-0.27	1.89E-02	-0.22	3.69E-03	ASPHD1
ENSG00000105717	0.26	1.90E-02	0.23	7.94E-02	PBX4
ENSG00000164620	-0.25	1.90E-02	-0.23	3.31E-03	RELL2
ENSG00000149761	-0.23	1.91E-02	-0.15	3.54E-02	NUDT22
ENSG00000128185	-0.21	1.92E-02	-0.13	9.18E-02	DGCR6L
ENSG00000225684	0.48	1.92E-02	0.44	1.95E-02	FAM225B
ENSG00000131480	0.38	1.92E-02	0.21	5.53E-02	AOC2
ENSG00000125843	-0.18	1.92E-02	-0.13	4.16E-02	AP5S1
ENSG00000232374	0.35	1.95E-02	0.27	3.26E-02	GPR79
ENSG00000213888	-0.24	1.99E-02	-0.17	8.03E-02	LINC01521
ENSG00000103316	-0.31	2.00E-02	-0.29	3.36E-04	CRYM
ENSG00000125877	-0.23	2.00E-02	-0.16	5.63E-02	ITPA
ENSG00000113369	0.34	2.03E-02	0.37	1.76E-04	ARRDC3
ENSG00000089356	0.37	2.04E-02	0.38	5.22E-04	FXYD3
ENSG00000109255	-0.49	2.07E-02	-0.52	5.71E-03	NMU
ENSG00000142627	0.45	2.09E-02	0.52	7.06E-03	EPHA2
ENSG00000129355	-0.27	2.10E-02	-0.17	7.44E-03	CDKN2D
ENSG00000127540	-0.19	2.16E-02	-0.16	5.27E-03	UQCR11
ENSG00000154309	0.21	2.20E-02	0.12	4.73E-02	DISP1

ENSG00000162076	-0.19	2.20E-02	-0.18	5.87E-03	FLYWCH2
ENSG00000151655	-0.35	2.24E-02	-0.26	1.36E-02	ITIH2
ENSG00000272944	-0.29	2.24E-02	-0.35	3.69E-05	
ENSG00000258861	-0.36	2.25E-02	-0.27	2.43E-02	MIR381HG
ENSG00000129158	-0.17	2.27E-02	-0.07	6.96E-02	SERGEF
ENSG00000137133	-0.21	2.27E-02	-0.21	4.61E-03	HINT2
ENSG00000244731	0.55	2.27E-02	0.31	6.98E-02	C4A
ENSG00000170634	-0.16	2.27E-02	-0.14	7.00E-02	ACYP2
ENSG00000161642	-0.28	2.29E-02	-0.24	1.38E-04	ZNF385A
ENSG00000133169	-0.30	2.30E-02	-0.23	1.66E-02	BEX1
ENSG00000231528	0.45	2.30E-02	0.40	2.68E-02	FAM225A
ENSG00000241684	0.22	2.38E-02	0.38	7.18E-07	ADAMTS9-AS2
ENSG00000261668	0.34	2.38E-02	0.46	5.33E-03	
ENSG00000090376	0.37	2.39E-02	0.38	2.48E-03	IRAK3
ENSG00000153233	-0.15	2.40E-02	-0.13	5.04E-02	PTPRR
ENSG00000106404	0.23	2.41E-02	-0.18	6.95E-02	CLDN15
ENSG00000173436	-0.18	2.41E-02	-0.14	8.10E-03	MINOS1
ENSG00000166451	-0.20	2.42E-02	-0.12	5.95E-02	CENPN
ENSG00000125901	-0.20	2.42E-02	-0.13	2.99E-02	MRPS26
ENSG00000197261	-0.56	2.43E-02	-0.62	1.64E-03	C6orf141
ENSG00000158457	0.17	2.44E-02	0.24	5.78E-03	TSPAN33
ENSG00000231185	-0.31	2.44E-02	-0.35	1.28E-03	SPRY4-AS1
ENSG00000102595	-0.10	2.55E-02	0.11	2.01E-02	UGGT2
ENSG00000235944	-0.20	2.56E-02	-0.11	5.34E-02	ZNF815P
ENSG00000170365	0.21	2.56E-02	0.17	5.77E-04	SMAD1
ENSG00000140798	-0.32	2.73E-02	-0.41	2.13E-06	ABCC12
ENSG00000115641	-0.16	2.73E-02	-0.19	1.08E-03	FHL2
ENSG00000196976	-0.29	2.75E-02	-0.20	7.82E-02	LAGE3
ENSG00000108219	0.17	2.76E-02	0.11	4.52E-02	TSPAN14
ENSG00000138028	-0.22	2.76E-02	-0.14	3.38E-02	CGREF1
ENSG00000148334	-0.18	2.76E-02	-0.13	2.09E-02	PTGES2
ENSG00000034152	0.28	2.79E-02	0.19	6.21E-02	MAP2K3
ENSG00000107554	0.18	2.85E-02	0.13	4.04E-03	DNMBP
ENSG00000139083	0.22	2.85E-02	0.14	9.54E-02	ETV6
ENSG00000213619	-0.19	2.85E-02	-0.17	1.71E-02	NDUFS3
ENSG00000255568	-0.31	2.85E-02	-0.22	6.90E-02	BRWD1-AS2
ENSG00000175182	-0.23	2.85E-02	-0.22	2.72E-04	FAM131A
ENSG00000165916	-0.18	2.86E-02	-0.20	1.44E-03	PSMC3
ENSG00000250031	0.32	2.87E-02	0.14	5.98E-02	

ENSG00000091409	0.24	2.91E-02	0.14	4.28E-02	ITGA6
ENSG00000124702	-0.22	2.95E-02	-0.16	1.11E-02	KLHDC3
ENSG00000174775	-0.16	2.95E-02	-0.12	7.86E-02	HRAS
ENSG00000184515	-0.35	3.00E-02	-0.27	3.17E-02	BEX5
ENSG00000137731	0.40	3.00E-02	0.36	1.44E-02	FXYD2
ENSG00000167705	-0.21	3.00E-02	-0.18	5.95E-02	RILP
ENSG00000163297	0.17	3.01E-02	0.17	3.06E-02	ANTXR2
ENSG00000163964	-0.14	3.01E-02	-0.10	5.86E-02	PIGX
ENSG00000155959	-0.21	3.03E-02	-0.12	9.97E-02	VBP1
ENSG00000087116	0.36	3.08E-02	0.73	3.57E-07	ADAMTS2
ENSG00000167779	-0.23	3.08E-02	-0.33	1.75E-03	IGFBP6
ENSG00000198315	0.13	3.09E-02	0.10	2.50E-02	ZKSCAN8
ENSG00000104872	-0.19	3.09E-02	-0.23	2.46E-05	PIH1D1
ENSG00000126432	-0.22	3.09E-02	-0.17	3.35E-02	PRDX5
ENSG00000090539	-0.17	3.11E-02	-0.11	9.14E-02	CHRD
ENSG00000101189	-0.14	3.11E-02	-0.11	4.70E-02	MRGBP
ENSG00000004799	0.39	3.12E-02	0.45	1.16E-03	PDK4
ENSG00000171159	-0.20	3.15E-02	-0.18	8.20E-02	C9orf16
ENSG00000125503	-0.17	3.16E-02	-0.19	9.66E-03	PPP1R12C
ENSG00000138623	-0.17	3.16E-02	-0.24	3.81E-04	SEMA7A
ENSG00000186081	-0.51	3.16E-02	-0.42	2.90E-02	KRT5
ENSG00000158683	0.27	3.17E-02	0.21	2.78E-02	PKD1L1
ENSG00000130244	-0.19	3.20E-02	-0.14	8.94E-03	FAM98C
ENSG00000174521	-0.29	3.20E-02	-0.29	4.83E-04	TTC9B
ENSG00000169432	0.20	3.20E-02	0.26	5.49E-03	SCN9A
ENSG00000117682	-0.11	3.22E-02	-0.12	1.07E-03	DHDDS
ENSG00000144834	-0.28	3.23E-02	-0.22	3.53E-03	TAGLN3
ENSG00000185115	-0.15	3.23E-02	-0.15	9.58E-04	NSMCE3
ENSG00000138604	-0.16	3.25E-02	-0.12	4.78E-03	GLCE
ENSG00000178980	-0.23	3.25E-02	-0.18	2.18E-02	SELENOW
ENSG00000030419	0.20	3.28E-02	0.34	3.76E-05	IKZF2
ENSG00000003989	0.36	3.33E-02	0.55	9.26E-06	SLC7A2
ENSG00000082293	-0.16	3.33E-02	-0.14	2.72E-02	COL19A1
ENSG00000116771	-0.23	3.36E-02	-0.23	1.22E-02	AGMAT
ENSG00000180992	-0.24	3.39E-02	-0.17	3.58E-02	MRPL14
ENSG00000249915	-0.17	3.41E-02	-0.11	1.67E-02	PDCD6
ENSG00000105639	0.27	3.42E-02	0.27	6.37E-04	JAK3
ENSG00000127364	0.26	3.49E-02	0.20	2.48E-03	TAS2R4
ENSG00000163382	-0.18	3.49E-02	-0.13	1.72E-02	NAXE

ENSG00000130702	0.20	3.50E-02	0.16	8.61E-02	LAMA5
ENSG00000254656	-0.27	3.53E-02	-0.24	2.59E-03	RTL1
ENSG00000185885	0.47	3.53E-02	0.38	8.95E-02	IFITM1
ENSG00000099901	-0.20	3.54E-02	-0.10	4.11E-02	RANBP1
ENSG00000131873	0.17	3.58E-02	0.17	4.80E-03	CHSY1
ENSG00000133265	-0.19	3.58E-02	-0.23	6.60E-04	HSPBP1
ENSG00000187778	-0.13	3.58E-02	-0.13	1.58E-02	MCRS1
ENSG00000251348	0.30	3.58E-02	0.42	1.74E-05	HSPD1P11
ENSG00000154930	-0.28	3.62E-02	-0.18	8.96E-02	ACSS1
ENSG00000157833	-0.29	3.62E-02	-0.31	1.50E-03	GAREM2
ENSG00000145681	-0.25	3.64E-02	-0.25	3.76E-03	HAPLN1
ENSG00000106367	-0.26	3.64E-02	-0.23	6.63E-04	AP1S1
ENSG00000253457	-0.27	3.65E-02	-0.23	3.26E-02	SMIM18
ENSG00000235863	-0.17	3.67E-02	-0.22	1.77E-03	B3GALT4
ENSG00000252010	-0.65	3.68E-02	-0.30	2.87E-02	SCARNA5
ENSG00000102554	0.24	3.70E-02	0.30	5.22E-03	KLF5
ENSG00000136280	-0.13	3.71E-02	-0.13	1.67E-02	CCM2
ENSG00000057593	-0.29	3.76E-02	-0.27	2.11E-02	F7
ENSG00000068078	-0.26	3.76E-02	-0.24	2.49E-02	FGFR3
ENSG00000126088	-0.16	3.76E-02	-0.20	1.35E-02	UROD
ENSG00000197363	-0.13	3.78E-02	-0.12	4.01E-02	ZNF517
ENSG00000180616	-0.21	3.80E-02	-0.21	1.75E-02	SSTR2
ENSG00000182541	0.26	3.81E-02	0.21	4.77E-03	LIMK2
ENSG00000187094	-0.30	3.82E-02	-0.24	6.81E-02	CCK
ENSG00000172053	-0.13	3.83E-02	-0.19	8.70E-06	QARS
ENSG00000175906	-0.29	3.87E-02	-0.36	1.91E-04	ARL4D
ENSG00000143036	0.29	3.89E-02	0.36	1.10E-04	SLC44A3
ENSG00000119787	-0.11	3.90E-02	0.08	4.06E-02	ATL2
ENSG00000124733	-0.20	3.90E-02	-0.26	6.03E-05	MEA1
ENSG00000226530	0.22	3.92E-02	0.27	1.33E-02	
ENSG00000101052	-0.16	3.92E-02	-0.09	8.63E-02	IFT52
ENSG00000230551	0.16	4.01E-02	0.16	2.16E-02	
ENSG00000100625	0.20	4.01E-02	0.40	2.12E-04	SIX4
ENSG00000167434	-0.24	4.01E-02	-0.27	1.66E-03	CA4
ENSG00000134594	-0.22	4.02E-02	-0.26	3.45E-04	RAB33A
ENSG00000149131	0.29	4.02E-02	0.24	4.15E-03	SERPING1
ENSG00000063180	-0.22	4.03E-02	-0.37	9.47E-05	CA11
ENSG00000214941	-0.19	4.06E-02	-0.11	9.34E-02	ZSWIM7
ENSG00000114395	-0.21	4.08E-02	-0.17	2.28E-02	CYB561D2

ENSG00000104611	0.26	4.10E-02	0.23	1.30E-02	SH2D4A
ENSG00000175104	0.15	4.10E-02	0.14	1.60E-04	TRAF6
ENSG00000265996	-0.34	4.10E-02	-0.47	5.88E-03	MIR3671
ENSG00000187953	-0.12	4.14E-02	-0.10	3.72E-02	PMS2CL
ENSG00000164761	0.43	4.16E-02	0.49	2.40E-03	TNFRSF11B
ENSG00000053372	-0.17	4.16E-02	-0.15	5.73E-03	MRTO4
ENSG00000143494	0.21	4.18E-02	0.35	9.26E-06	VASH2
ENSG00000161981	-0.20	4.18E-02	-0.23	3.35E-03	SNRNP25
ENSG00000165806	0.28	4.23E-02	0.32	3.23E-03	CASP7
ENSG00000167733	-0.22	4.23E-02	-0.18	5.58E-03	HSD11B1L
ENSG00000185272	-0.17	4.25E-02	0.15	6.17E-02	RBM11
ENSG00000187601	-0.19	4.28E-02	-0.11	8.37E-02	MAGEH1
ENSG00000227544	-0.35	4.29E-02	-0.52	2.36E-05	
ENSG00000041357	-0.16	4.29E-02	-0.17	1.62E-02	PSMA4
ENSG00000175602	-0.30	4.30E-02	-0.18	8.37E-02	CCDC85B
ENSG00000172009	-0.14	4.34E-02	-0.10	5.39E-02	THOP1
ENSG00000221838	-0.13	4.35E-02	-0.17	1.95E-03	AP4M1
ENSG00000273270	-0.24	4.36E-02	-0.11	5.17E-02	
ENSG00000146411	0.15	4.41E-02	0.19	3.29E-03	SLC2A12
ENSG00000107738	0.29	4.44E-02	0.33	2.04E-03	VSIR
ENSG00000172115	-0.22	4.49E-02	-0.14	9.66E-02	CYCS
ENSG00000089123	-0.13	4.51E-02	-0.08	5.01E-02	TASP1
ENSG00000245532	0.45	4.55E-02	0.42	9.68E-03	NEAT1
ENSG00000187556	-0.25	4.57E-02	-0.25	8.92E-03	NANOS3
ENSG00000141469	0.48	4.57E-02	0.96	3.68E-06	SLC14A1
ENSG00000100276	-0.29	4.57E-02	-0.42	5.10E-06	RASL10A
ENSG00000168734	-0.16	4.57E-02	-0.13	2.97E-02	PKIG
ENSG00000081320	0.29	4.57E-02	0.55	2.71E-09	STK17B
ENSG00000130520	-0.18	4.58E-02	-0.12	6.23E-02	LSM4
ENSG00000129116	0.20	4.59E-02	0.20	1.40E-04	PALLD
ENSG00000178878	0.48	4.59E-02	0.54	5.35E-02	APOLD1
ENSG00000196262	-0.18	4.59E-02	-0.24	9.79E-03	PPIA
ENSG00000198517	-0.23	4.59E-02	-0.29	1.29E-02	MAFK
ENSG00000007866	0.30	4.60E-02	0.29	5.52E-02	TEAD3
ENSG00000136010	0.18	4.65E-02	0.24	1.92E-05	ALDH1L2
ENSG00000110583	-0.11	4.66E-02	-0.15	1.37E-06	NAA40
ENSG00000139291	-0.14	4.66E-02	-0.12	3.18E-02	TMEM19
ENSG00000107262	-0.16	4.67E-02	-0.10	5.30E-02	BAG1
ENSG00000174177	-0.15	4.67E-02	-0.16	9.06E-03	CTU2

ENSG00000175782	-0.14	4.67E-02	-0.09	4.32E-02	SLC35E3
ENSG00000184445	-0.12	4.67E-02	0.15	5.73E-03	KNTC1
ENSG00000196668	0.28	4.71E-02	0.35	2.02E-03	LINC00173
ENSG00000071655	-0.17	4.72E-02	-0.15	5.32E-02	MBD3
ENSG00000141504	-0.17	4.74E-02	-0.13	4.77E-02	SAT2
ENSG00000196449	-0.14	4.84E-02	-0.10	9.45E-02	YRDC
ENSG00000163884	0.23	4.84E-02	0.46	9.26E-06	KLF15
ENSG00000148814	-0.10	4.88E-02	-0.08	4.86E-02	LRRC27
ENSG00000101132	-0.21	4.96E-02	-0.15	3.48E-02	PFDN4
ENSG00000120519	0.13	4.98E-02	0.10	2.62E-02	SLC10A7
ENSG00000149573	0.61	4.99E-02	1.11	1.46E-05	MPZL2
ENSG00000215066	0.27	4.99E-02	0.22	2.32E-02	NA
ENSG00000100479	0.22	5.01E-02	0.22	4.33E-02	POLE2
ENSG00000214087	-0.15	5.01E-02	-0.12	4.78E-02	ARL16
ENSG00000165030	0.29	5.03E-02	0.28	1.63E-02	NFIL3
ENSG00000120693	0.18	5.04E-02	0.20	1.43E-02	SMAD9
ENSG00000253636	0.32	5.04E-02	0.18	2.07E-02	
ENSG00000270061	0.23	5.04E-02	0.17	8.48E-02	
ENSG00000138678	-0.21	5.04E-02	-0.34	8.84E-03	GPAT3
ENSG00000172380	0.20	5.05E-02	0.29	9.85E-05	GNG12
ENSG00000179431	-0.22	5.05E-02	-0.22	2.50E-02	FJX1
ENSG00000112964	0.16	5.06E-02	0.17	4.61E-02	GHR
ENSG00000169599	-0.19	5.07E-02	-0.14	3.31E-02	NFU1
ENSG00000176101	-0.17	5.07E-02	-0.16	2.82E-02	SSNA1
ENSG00000130307	-0.25	5.08E-02	-0.28	3.04E-02	USHBP1
ENSG00000102981	-0.18	5.10E-02	-0.16	5.22E-02	PARD6A
ENSG00000063176	-0.16	5.12E-02	-0.16	3.78E-02	SPHK2
ENSG00000188868	-0.15	5.12E-02	0.14	4.80E-02	ZNF563
ENSG00000124160	0.19	5.16E-02	-0.12	6.41E-02	NCOA5
ENSG00000119403	0.18	5.22E-02	0.18	7.89E-03	PHF19
ENSG00000168874	0.31	5.24E-02	0.22	4.49E-02	ATOH8
ENSG00000196456	-0.11	5.24E-02	-0.10	2.64E-02	ZNF775
ENSG00000204603	-0.39	5.24E-02	-0.60	1.33E-04	LINC01257
ENSG00000135070	-0.16	5.24E-02	-0.11	9.91E-02	ISCA1
ENSG00000072954	-0.16	5.30E-02	-0.20	2.47E-05	TMEM38A
ENSG00000019991	0.34	5.32E-02	0.38	8.33E-03	HGF
ENSG00000183250	-0.22	5.32E-02	-0.22	1.88E-02	LINC01547
ENSG00000179841	-0.20	5.36E-02	-0.16	2.62E-02	AKAP5
ENSG00000244556	0.26	5.36E-02	0.16	5.12E-02	ODCP

ENSG00000136141	0.13	5.38E-02	0.07	6.04E-02	LRCH1
ENSG00000101180	0.22	5.46E-02	0.17	4.61E-02	HRH3
ENSG00000165076	0.36	5.46E-02	0.51	8.94E-05	PRSS37
ENSG00000091831	0.29	5.59E-02	0.20	8.33E-03	ESR1
ENSG00000157593	-0.16	5.63E-02	-0.19	8.55E-04	SLC35B2
ENSG00000273081	-0.15	5.63E-02	-0.14	8.68E-02	NA
ENSG00000130224	0.08	5.65E-02	0.11	3.78E-02	LRCH2
ENSG00000109016	-0.16	5.65E-02	-0.14	1.45E-02	DHRS7B
ENSG00000129990	-0.16	5.69E-02	-0.28	4.59E-07	SYT5
ENSG00000183828	-0.21	5.69E-02	-0.26	6.15E-04	NUDT14
ENSG00000127947	0.12	5.71E-02	0.14	3.75E-04	PTPN12
ENSG00000189238	-0.33	5.71E-02	-0.36	9.52E-03	LINC00943
ENSG00000107623	0.23	5.74E-02	0.45	1.46E-05	NA
ENSG00000249767	0.28	5.74E-02	0.21	4.23E-02	ENPP7P10
ENSG00000162910	-0.19	5.76E-02	-0.15	9.13E-02	MRPL55
ENSG00000159674	-0.31	5.77E-02	-0.24	1.66E-02	SPON2
ENSG00000179833	0.20	5.79E-02	0.13	2.74E-02	SERTAD2
ENSG00000118520	0.32	5.81E-02	0.36	1.88E-02	ARG1
ENSG00000136950	-0.18	5.83E-02	-0.14	2.42E-02	ARPC5L
ENSG00000112425	-0.15	5.83E-02	-0.12	6.81E-03	EPM2A
ENSG00000243051	0.30	5.86E-02	0.23	5.96E-02	RN7SL269P
ENSG00000107833	-0.24	5.87E-02	-0.17	4.24E-02	NPM3
ENSG00000168291	-0.18	5.89E-02	-0.15	1.01E-02	PDHB
ENSG00000121904	0.20	5.96E-02	0.11	6.35E-02	CSMD2
ENSG00000198915	-0.12	6.00E-02	-0.09	7.20E-02	RASGEF1A
ENSG00000147454	0.17	6.04E-02	0.11	8.59E-02	SLC25A37
ENSG00000157734	-0.23	6.04E-02	-0.23	1.38E-03	SNX22
ENSG00000165782	-0.12	6.04E-02	-0.13	2.38E-03	PIP4P1
ENSG00000237720	-0.19	6.04E-02	-0.29	5.59E-05	
ENSG00000158234	-0.14	6.10E-02	-0.15	3.76E-02	FAIM
ENSG00000147684	-0.19	6.13E-02	-0.23	1.20E-04	NDUFB9
ENSG00000167550	-0.25	6.13E-02	-0.26	1.51E-02	RHEBL1
ENSG00000167588	-0.27	6.20E-02	-0.59	5.56E-07	GPD1
ENSG00000124508	0.26	6.24E-02	0.35	2.95E-02	BTN2A2
ENSG00000142530	-0.23	6.24E-02	-0.31	3.02E-03	FAM71E1
ENSG00000120798	-0.09	6.26E-02	0.08	3.58E-02	NR2C1
ENSG00000180332	-0.18	6.31E-02	-0.37	3.77E-03	KCTD4
ENSG00000114383	-0.16	6.32E-02	-0.12	9.57E-03	TUSC2
ENSG00000117500	0.17	6.35E-02	0.16	1.88E-03	TMED5

ENSG00000178860	-0.29	6.35E-02	-0.30	5.75E-02	MSC
ENSG00000248508	0.21	6.35E-02	0.18	9.93E-02	SRP14-AS1
ENSG00000163528	-0.14	6.43E-02	-0.12	7.27E-02	CHCHD4
ENSG00000161326	-0.18	6.43E-02	-0.22	1.35E-03	NA
ENSG00000010438	-0.23	6.47E-02	-0.19	8.54E-02	PRSS3
ENSG00000167112	-0.16	6.53E-02	-0.11	2.96E-02	TRUB2
ENSG00000205885	0.28	6.56E-02	0.19	1.16E-02	C1RL-AS1
ENSG00000170075	-0.20	6.57E-02	-0.18	4.61E-02	GPR37L1
ENSG00000147679	-0.11	6.60E-02	0.08	1.11E-02	UTP23
ENSG00000089693	-0.15	6.61E-02	-0.12	6.23E-02	MLF2
ENSG00000145246	0.12	6.61E-02	0.14	9.26E-03	ATP10D
ENSG00000255471	0.20	6.68E-02	0.21	1.67E-02	
ENSG00000175806	-0.11	6.68E-02	-0.07	7.27E-02	MSRA
ENSG00000109762	-0.12	6.69E-02	-0.13	2.98E-03	SNX25
ENSG00000100629	0.15	6.69E-02	0.11	5.88E-02	CEP128
ENSG00000006128	-0.39	6.75E-02	-0.46	4.39E-04	TAC1
ENSG00000135469	-0.15	6.84E-02	-0.12	8.48E-02	COQ10A
ENSG00000170379	0.31	6.84E-02	0.42	2.37E-04	TCAF2
ENSG00000233559	0.28	6.85E-02	0.14	6.43E-02	LINC00513
ENSG00000197982	-0.24	6.86E-02	-0.14	7.55E-02	C1orf122
ENSG00000129103	-0.14	6.87E-02	-0.13	3.26E-02	SUMF2
ENSG00000163449	-0.15	6.87E-02	-0.21	1.62E-04	TMEM169
ENSG00000265313	-0.26	6.87E-02	-0.26	1.90E-02	LINC01476
ENSG00000054803	-0.25	6.87E-02	-0.15	4.64E-02	CBLN4
ENSG00000144550	-0.18	6.87E-02	-0.31	3.54E-04	CPNE9
ENSG00000159199	-0.21	6.89E-02	-0.21	7.98E-02	ATP5MC1
ENSG00000145354	-0.16	6.90E-02	-0.12	4.86E-02	CISD2
ENSG00000249307	0.39	6.90E-02	0.45	4.25E-02	LINC01088
ENSG00000116096	0.23	6.92E-02	0.20	6.58E-02	SPR
ENSG00000148498	0.17	6.93E-02	0.25	2.48E-04	PARD3
ENSG00000154330	-0.21	6.93E-02	-0.36	2.84E-03	PGM5
ENSG00000178127	0.15	6.93E-02	0.12	7.59E-02	NDUFV2
ENSG00000185420	-0.09	6.95E-02	0.09	3.49E-02	SMYD3
ENSG00000169900	-0.34	7.05E-02	0.47	1.20E-02	PYDC1
ENSG00000153094	0.15	7.07E-02	0.18	1.24E-03	BCL2L11
ENSG00000129480	-0.16	7.08E-02	-0.12	3.15E-02	DTD2
ENSG00000188559	0.13	7.09E-02	0.08	7.74E-02	RALGAPA2
ENSG00000145832	-0.25	7.10E-02	0.33	1.29E-02	SLC25A48
ENSG00000088727	-0.14	7.14E-02	-0.12	2.97E-02	KIF9

ENSG00000047662	0.17	7.15E-02	0.19	2.23E-02	FAM184B
ENSG00000069206	0.39	7.15E-02	0.35	5.29E-03	ADAM7
ENSG00000083223	0.10	7.15E-02	0.08	4.18E-02	ZCCHC6
ENSG00000137806	-0.16	7.15E-02	-0.14	5.88E-02	NDUFAF1
ENSG00000006194	-0.10	7.16E-02	-0.08	6.38E-02	ZNF263
ENSG00000186017	-0.09	7.16E-02	0.07	9.79E-03	ZNF566
ENSG00000169313	-0.35	7.16E-02	-0.53	4.54E-03	P2RY12
ENSG00000197380	-0.15	7.16E-02	-0.09	9.93E-02	DACT3
ENSG00000237719	0.28	7.16E-02	0.21	5.81E-02	
ENSG00000171574	-0.12	7.25E-02	-0.10	6.44E-02	ZNF584
ENSG00000253882	0.33	7.28E-02	0.42	1.77E-03	
ENSG00000162614	0.24	7.31E-02	0.32	4.91E-03	NEXN
ENSG00000010256	-0.16	7.31E-02	-0.16	5.33E-03	UQCRC1
ENSG00000239002	-0.55	7.33E-02	-0.30	7.21E-02	SCARNA10
ENSG00000171817	-0.12	7.35E-02	0.09	2.49E-02	ZNF540
ENSG00000039650	-0.13	7.37E-02	-0.20	2.08E-03	PNKP
ENSG00000067955	0.19	7.37E-02	0.17	1.59E-02	CBFB
ENSG00000110013	-0.11	7.37E-02	-0.09	9.50E-02	SIAE
ENSG00000147155	-0.17	7.48E-02	-0.25	1.57E-03	EBP
ENSG00000235314	-0.18	7.48E-02	-0.14	8.67E-02	LINC00957
ENSG00000115137	-0.12	7.51E-02	-0.09	1.46E-02	DNAJC27
ENSG00000235478	-0.25	7.51E-02	-0.24	5.49E-03	LINC01664
ENSG00000130749	0.14	7.51E-02	-0.09	8.78E-02	ZC3H4
ENSG00000146469	-0.31	7.52E-02	-0.28	9.72E-03	VIP
ENSG00000198890	-0.13	7.52E-02	-0.10	9.46E-02	PRMT6
ENSG00000122574	0.22	7.56E-02	0.38	9.96E-09	WIPF3
ENSG00000168936	-0.17	7.56E-02	-0.14	5.05E-02	TMEM129
ENSG00000092531	0.17	7.61E-02	0.13	5.76E-02	SNAP23
ENSG00000139117	0.11	7.61E-02	0.17	1.90E-03	CPNE8
ENSG00000141098	-0.12	7.61E-02	-0.16	5.76E-05	GFOD2
ENSG00000204237	-0.15	7.61E-02	-0.14	6.23E-02	OXLD1
ENSG00000248228	0.34	7.61E-02	0.32	1.38E-03	SLIT2-IT1
ENSG00000169118	0.09	7.65E-02	0.08	3.12E-02	CSNK1G1
ENSG00000177054	-0.08	7.69E-02	0.15	3.67E-03	ZDHHC13
ENSG00000225205	0.22	7.71E-02	0.21	6.80E-02	
ENSG00000136052	-0.07	7.72E-02	0.08	3.04E-02	SLC41A2
ENSG00000139714	-0.22	7.73E-02	-0.24	5.18E-03	MORN3
ENSG00000086717	-0.30	7.73E-02	-0.28	9.56E-03	PPEF1
ENSG00000204131	0.21	7.79E-02	0.16	8.82E-02	NHSL2

ENSG00000158220	0.16	7.83E-02	0.25	1.36E-05	ESYT3
ENSG00000164292	0.18	7.84E-02	0.38	9.84E-08	RHOBTB3
ENSG00000101940	-0.13	7.89E-02	-0.15	1.60E-02	WDR13
ENSG00000237684	0.38	7.90E-02	0.38	5.95E-02	RPSAP35
ENSG00000246082	0.23	7.90E-02	0.40	1.23E-05	NUDT16P1
ENSG00000135605	0.25	7.98E-02	0.63	9.96E-09	TEC
ENSG00000139637	-0.13	7.98E-02	-0.11	6.33E-02	C12orf10
ENSG00000272121	-0.25	8.01E-02	-0.24	7.49E-02	
ENSG00000117533	-0.11	8.03E-02	0.09	2.78E-02	VAMP4
ENSG00000143199	0.25	8.04E-02	0.24	2.95E-02	ADCY10
ENSG00000130511	-0.18	8.05E-02	-0.27	8.81E-04	SSBP4
ENSG00000074695	0.10	8.09E-02	0.15	2.01E-04	LMAN1
ENSG00000106537	-0.16	8.10E-02	-0.12	4.18E-02	TSPAN13
ENSG00000138622	0.22	8.13E-02	0.31	2.22E-03	HCN4
ENSG00000214872	0.25	8.13E-02	0.39	4.21E-03	SMTNL1
ENSG00000260912	0.22	8.15E-02	0.24	2.07E-02	
ENSG00000116661	-0.16	8.33E-02	-0.16	1.18E-02	FBXO2
ENSG00000127362	0.25	8.34E-02	0.33	2.39E-05	TAS2R3
ENSG00000114054	-0.11	8.35E-02	-0.08	9.99E-02	PCCB
ENSG00000159023	0.14	8.40E-02	0.13	8.90E-02	EPB41
ENSG00000261087	0.21	8.42E-02	0.16	2.24E-02	
ENSG00000106080	0.11	8.42E-02	0.16	1.02E-02	FKBP14
ENSG00000095464	0.23	8.42E-02	0.28	1.91E-03	PDE6C
ENSG00000197321	0.15	8.43E-02	0.15	6.58E-02	SVIL
ENSG00000120437	-0.20	8.44E-02	-0.29	8.47E-04	ACAT2
ENSG00000182324	-0.27	8.45E-02	-0.35	4.47E-03	KCNJ14
ENSG00000165973	-0.17	8.46E-02	-0.20	9.79E-03	NELL1
ENSG00000197992	-0.42	8.48E-02	-0.66	9.25E-03	CLEC9A
ENSG00000180902	-0.16	8.49E-02	-0.24	6.92E-05	D2HGDH
ENSG00000186063	0.11	8.51E-02	0.15	7.00E-05	AIDA
ENSG00000152944	-0.10	8.54E-02	0.15	1.23E-03	MED21
ENSG00000179397	-0.14	8.57E-02	0.11	6.21E-02	CATSPERE
ENSG00000198732	0.20	8.58E-02	0.19	6.69E-02	SMOC1
ENSG00000144227	-0.26	8.62E-02	-0.25	2.36E-02	NXPH2
ENSG00000140365	-0.14	8.71E-02	-0.14	9.04E-03	COMMD4
ENSG00000088766	-0.11	8.72E-02	0.12	2.63E-03	CRLS1
ENSG00000071054	0.19	8.73E-02	-0.16	3.67E-03	MAP4K4
ENSG00000183780	-0.12	8.79E-02	-0.07	8.36E-02	SLC35F3
ENSG00000198729	-0.20	8.79E-02	-0.17	3.05E-02	PPP1R14C

ENSG00000261589	0.22	8.81E-02	0.31	2.30E-04	NA
ENSG00000112237	-0.11	8.82E-02	0.11	5.20E-03	CCNC
ENSG00000259619	0.34	8.82E-02	0.27	4.07E-02	
ENSG00000273274	0.19	8.82E-02	0.16	2.23E-02	ZBTB8B
ENSG00000177731	0.12	8.85E-02	-0.13	1.38E-02	FLII
ENSG00000173227	-0.19	8.89E-02	-0.34	1.34E-05	SYT12
ENSG00000135097	-0.23	8.90E-02	-0.18	4.04E-02	MSI1
ENSG00000100906	0.23	8.95E-02	0.27	5.25E-02	NFKBIA
ENSG00000175895	0.15	8.96E-02	0.20	4.25E-03	PLEKHF2
ENSG00000173511	-0.11	8.97E-02	-0.14	7.18E-03	VEGFB
ENSG00000254999	-0.14	8.97E-02	-0.17	5.64E-02	BRK1
ENSG00000188612	-0.13	8.99E-02	-0.13	8.98E-02	SUMO2
ENSG00000159860	0.32	8.99E-02	0.44	2.15E-03	TCAF2P1
ENSG00000007062	-0.18	9.08E-02	-0.37	1.69E-05	PROM1
ENSG00000100652	0.26	9.10E-02	0.44	2.33E-03	SLC10A1
ENSG00000163629	0.11	9.10E-02	0.14	3.31E-02	PTPN13
ENSG00000117410	-0.20	9.12E-02	-0.20	4.49E-02	ATP6V0B
ENSG00000121210	0.14	9.15E-02	0.13	9.71E-03	TMEM131L
ENSG00000175318	0.21	9.15E-02	0.22	4.98E-02	GRAMD2A
ENSG00000186073	0.12	9.15E-02	0.14	1.12E-02	C15orf41
ENSG00000050130	-0.09	9.18E-02	-0.08	8.11E-02	JKAMP
ENSG00000196189	-0.13	9.22E-02	-0.21	9.29E-03	SEMA4A
ENSG00000271971	0.18	9.22E-02	0.19	1.59E-02	
ENSG00000249790	0.22	9.25E-02	0.46	8.63E-02	
ENSG00000139597	-0.10	9.26E-02	0.10	4.86E-02	N4BP2L1
ENSG00000119688	0.11	9.27E-02	0.10	5.92E-02	ABCD4
ENSG00000177692	-0.17	9.29E-02	-0.18	4.81E-03	DNAJC28
ENSG00000104231	-0.11	9.31E-02	0.11	2.78E-02	ZFAND1
ENSG00000066382	-0.10	9.32E-02	-0.11	3.50E-03	MPPED2
ENSG00000152413	-0.16	9.32E-02	-0.29	1.78E-05	HOMER1
ENSG00000158552	-0.12	9.33E-02	-0.11	4.56E-02	ZFAND2B
ENSG00000251379	0.21	9.33E-02	0.57	5.49E-03	
ENSG00000171490	-0.09	9.45E-02	-0.08	3.36E-02	RSL1D1
ENSG00000179294	-0.23	9.50E-02	-0.42	5.96E-06	NA
ENSG00000101463	-0.11	9.52E-02	-0.21	3.41E-04	SYNDIG1
ENSG00000124787	-0.15	9.52E-02	-0.18	3.15E-02	RPP40
ENSG00000165449	0.24	9.52E-02	0.42	1.94E-05	SLC16A9
ENSG00000243715	0.27	9.55E-02	0.36	1.23E-05	CACNA2D3-AS1
ENSG00000228653	0.20	9.68E-02	0.15	7.28E-02	HNRNPCP7

ENSG00000161912	0.14	9.74E-02	0.12	8.27E-02	ADCY10P1
ENSG00000170955	-0.26	9.75E-02	-0.39	1.25E-02	CAVIN3
ENSG00000183255	0.19	9.75E-02	0.12	2.66E-02	PTTG1IP
ENSG00000160194	-0.13	9.75E-02	-0.13	4.69E-02	NDUFV3
ENSG00000226137	-0.17	9.77E-02	-0.21	5.98E-04	BAIAP2-AS1
ENSG00000269956	0.20	9.77E-02	0.24	2.28E-03	MKNK1-AS1
ENSG00000152229	0.35	9.92E-02	0.50	2.09E-02	PSTPIP2
ENSG00000273472	-0.23	9.94E-02	-0.26	3.09E-02	
ENSG00000149809	-0.14	9.96E-02	-0.25	2.42E-04	TM7SF2
ENSG00000181104	0.18	9.96E-02	0.26	3.32E-02	F2R
ENSG00000100023	0.09	9.96E-02	-0.07	8.42E-02	PPIL2
ENSG00000152952	0.19	9.96E-02	0.24	3.03E-03	PLOD2
ENSG00000172671	0.13	1.00E-01	0.11	8.59E-02	ZFAND4

Supplementary Table 2: Differentially Expressed Genes in PsychENCODE				
	log2Fold Change	padj	Gene	Sig. in CMC
ENSG00000107821	-2.13	4.17E-03	KAZALD1	Y
ENSG00000057593	-1.90	1.55E-02	F7	Y
ENSG00000114739	0.67	1.55E-02	ACVR2B	Y
ENSG00000162600	1.44	1.55E-02	OMA1	N
ENSG00000185823	2.55	2.29E-02	NPAP1	Y
ENSG00000118503	-4.14	2.78E-02	TNFAIP3	N
ENSG00000128039	1.11	2.78E-02	SRD5A3	Y
ENSG00000249348	1.23	2.78E-02	UGDH-AS1	Y
ENSG00000152229	-4.04	3.06E-02	PSTPIP2	Y
ENSG00000175832	-1.60	3.66E-02	ETV4	N
ENSG00000263477	1.37	3.69E-02		Y
ENSG00000233421	-3.36	4.21E-02	LINC01783	N
ENSG00000144036	0.53	5.19E-02	EXOC6B	N
ENSG00000083290	0.47	5.28E-02	ULK2	N
ENSG00000143442	0.55	5.28E-02	POGZ	Y
ENSG00000145824	-1.51	6.15E-02	CXCL14	N
ENSG00000167103	-1.50	6.15E-02	PIP5KL1	Y
ENSG00000108375	-1.64	7.27E-02	RNF43	N
ENSG00000140386	0.49	7.56E-02	SCAPER	N
ENSG00000250366	-1.61	7.56E-02	TUNAR	N
ENSG00000272541	1.34	7.56E-02		N
ENSG00000186162	-0.77	8.36E-02	CIDECP	Y
ENSG00000107165	1.78	8.56E-02	TYRP1	Y
ENSG00000140511	-3.04	8.56E-02	HAPLN3	N
ENSG00000130208	-2.34	8.62E-02	APOC1	N
ENSG00000185104	0.40	8.62E-02	FAF1	Y
ENSG00000253161	-2.08	8.62E-02	LINC01605	N
ENSG00000127423	-1.17	9.24E-02	AUNIP	Y
ENSG00000080573	-1.53	9.41E-02	COL5A3	Y

Supplementary Table 3: Gene Set Enrichment Analysis of Differentially Expressed Genes in CommonMind				
Category	Term	Gene Count	Fold Enrichment	Bonferroni
SP_PIR_KEYWORDS	acetylation	1070	1.50	4.75E-57
SP_PIR_KEYWORDS	phosphoprotein	2375	1.21	3.55E-40
GOTERM_CC_FAT	GO:0005739~mitochondrion	458	1.58	6.12E-28
GOTERM_CC_FAT	GO:0030529~ribonucleoprotein complex	250	1.82	3.61E-24
SP_PIR_KEYWORDS	mitochondrion	359	1.60	9.83E-22
SP_PIR_KEYWORDS	alternative splicing	2325	1.15	4.17E-21
GOTERM_CC_FAT	GO:0070013~intracellular organelle lumen	647	1.36	1.38E-19
GOTERM_CC_FAT	GO:0044429~mitochondrial part	266	1.67	3.02E-19
GOTERM_CC_FAT	GO:0005840~ribosome	125	2.18	3.11E-19
SP_PIR_KEYWORDS	ribonucleoprotein	152	2.01	3.53E-19
GOTERM_CC_FAT	GO:0031974~membrane-enclosed lumen	668	1.35	4.84E-19
UP_SEQ_FEATURE	splice variant	2319	1.14	7.64E-19
GOTERM_CC_FAT	GO:0043233~organelle lumen	653	1.34	3.91E-18
SP_PIR_KEYWORDS	ribosomal protein	112	2.20	1.38E-17
GOTERM_BP_FAT	GO:0006412~translation	172	1.89	1.67E-17
SP_PIR_KEYWORDS	transit peptide	217	1.69	1.43E-15
SP_PIR_KEYWORDS	nucleus	1381	1.19	1.58E-15
GOTERM_CC_FAT	GO:0031090~organelle membrane	414	1.41	1.93E-14
GOTERM_CC_FAT	GO:0031981~nuclear lumen	523	1.35	2.52E-14
UP_SEQ_FEATURE	transit peptide:Mitochondrion	213	1.68	6.29E-14
KEGG_PATHWAY	hsa05012:Parkinson's disease	79	2.27	1.07E-13
GOTERM_CC_FAT	GO:0005829~cytosol	485	1.37	1.84E-13
GOTERM_CC_FAT	GO:0033279~ribosomal subunit	79	2.31	2.76E-13
GOTERM_CC_FAT	GO:0019866~organelle inner membrane	156	1.78	6.45E-13
GOTERM_MF_FAT	GO:0003735~structural constituent of ribosome	97	2.11	6.48E-13
KEGG_PATHWAY	hsa05016:Huntington's disease	99	2.02	6.61E-13
GOTERM_CC_FAT	GO:0005740~mitochondrial envelope	185	1.65	7.19E-12
GOTERM_CC_FAT	GO:0005743~mitochondrial inner membrane	145	1.77	7.92E-12

SP_PIR_KEYWORDS	cytoplasm	1080	1.20	2.44E-11
SP_PIR_KEYWORDS	respiratory chain	51	2.66	2.50E-11
GOTERM_CC_FAT	GO:0031966~mitochondrial membrane	174	1.65	4.75E-11
SP_PIR_KEYWORDS	mitochondrion inner membrane	102	1.95	5.70E-11
GOTERM_CC_FAT	GO:0031975~envelope	248	1.49	2.54E-10
GOTERM_CC_FAT	GO:0031967~organelle envelope	247	1.49	3.16E-10
KEGG_PATHWAY	hsa00190:Oxidative phosphorylation	74	2.09	3.27E-10
GOTERM_MF_FAT	GO:0000166~nucleotide binding	755	1.23	9.98E-10
SP_PIR_KEYWORDS	zinc-finger	591	1.27	1.34E-09
SP_PIR_KEYWORDS	protein biosynthesis	97	1.91	1.58E-09
KEGG_PATHWAY	hsa05010:Alzheimer's disease	85	1.92	3.44E-09
GOTERM_BP_FAT	GO:0044265~cellular macromolecule catabolic process	286	1.43	5.59E-09
KEGG_PATHWAY	hsa03010:Ribosome	54	2.28	5.81E-09
GOTERM_BP_FAT	GO:0051603~proteolysis involved in cellular protein catabolic process	243	1.47	1.44E-08
GOTERM_BP_FAT	GO:0044257~cellular protein catabolic process	243	1.46	2.66E-08
GOTERM_BP_FAT	GO:0070647~protein modification by small protein conjugation or removal	86	1.95	2.90E-08
GOTERM_CC_FAT	GO:0005654~nucleoplasm	322	1.37	2.97E-08
GOTERM_BP_FAT	GO:0030163~protein catabolic process	249	1.45	3.20E-08
GOTERM_CC_FAT	GO:0044455~mitochondrial membrane part	69	2.07	3.54E-08
GOTERM_BP_FAT	GO:0009057~macromolecule catabolic process	300	1.39	5.85E-08
GOTERM_CC_FAT	GO:0043228~non-membrane-bounded organelle	825	1.19	6.46E-08
GOTERM_CC_FAT	GO:0043232~intracellular non-membrane-bounded organelle	825	1.19	6.46E-08
SP_PIR_KEYWORDS	metal-binding	948	1.18	9.35E-08
GOTERM_CC_FAT	GO:0070469~respiratory chain	47	2.35	2.41E-07

GOTERM_BP_FAT	GO:0006414~translational elongation	60	2.16	3.27E-07
GOTERM_BP_FAT	GO:0043632~modification-dependent macromolecule catabolic process	229	1.45	3.61E-07
GOTERM_BP_FAT	GO:0019941~modification-dependent protein catabolic process	229	1.45	3.61E-07
GOTERM_CC_FAT	GO:0022626~cytosolic ribosome	49	2.27	5.34E-07
GOTERM_MF_FAT	GO:0003723~RNA binding	272	1.38	5.92E-07
SP_PIR_KEYWORDS	protein transport	193	1.47	6.97E-07
GOTERM_CC_FAT	GO:0000313~organellar ribosome	34	2.65	1.11E-06
GOTERM_CC_FAT	GO:0005761~mitochondrial ribosome	34	2.65	1.11E-06
SP_PIR_KEYWORDS	zinc	711	1.20	1.32E-06
SP_PIR_KEYWORDS	ubl conjugation pathway	199	1.45	2.06E-06
SP_PIR_KEYWORDS	nucleotide-binding	561	1.23	2.11E-06
GOTERM_BP_FAT	GO:0006396~RNA processing	217	1.44	2.19E-06
GOTERM_CC_FAT	GO:0015934~large ribosomal subunit	42	2.35	2.46E-06
SP_PIR_KEYWORDS	ligase	130	1.58	4.14E-06
KEGG_PATHWAY	hsa04120:Ubiquitin mediated proteolysis	68	1.82	6.36E-06
SP_PIR_KEYWORDS	rna-binding	207	1.42	6.37E-06
GOTERM_CC_FAT	GO:0005746~mitochondrial respiratory chain	40	2.34	7.07E-06
SP_PIR_KEYWORDS	ribosome	44	2.23	8.44E-06
SP_PIR_KEYWORDS	atp-binding	448	1.25	1.36E-05
GOTERM_BP_FAT	GO:0015031~protein transport	283	1.35	1.43E-05
GOTERM_CC_FAT	GO:0031980~mitochondrial lumen	100	1.65	1.83E-05
GOTERM_CC_FAT	GO:0005759~mitochondrial matrix	100	1.65	1.83E-05
GOTERM_MF_FAT	GO:0032555~purine ribonucleotide binding	605	1.20	1.84E-05
GOTERM_MF_FAT	GO:0032553~ribonucleotide binding	605	1.20	1.84E-05
GOTERM_BP_FAT	GO:0045184~establishment of protein localization	284	1.34	2.49E-05

GOTERM_MF_FAT	GO:0017076~purine nucleotide binding	628	1.20	2.62E-05
GOTERM_MF_FAT	GO:0019787~small conjugating protein ligase activity	80	1.76	3.60E-05
GOTERM_BP_FAT	GO:0006119~oxidative phosphorylation	55	2.04	3.80E-05
GOTERM_BP_FAT	GO:0008104~protein localization	318	1.31	5.15E-05
SP_PIR_KEYWORDS	mrna processing	111	1.58	5.35E-05
GOTERM_CC_FAT	GO:0045271~respiratory chain complex I	29	2.59	5.65E-05
GOTERM_CC_FAT	GO:0005747~mitochondrial respiratory chain complex I	29	2.59	5.65E-05
GOTERM_CC_FAT	GO:0030964~NADH dehydrogenase complex	29	2.59	5.65E-05
GOTERM_BP_FAT	GO:0032446~protein modification by small protein conjugation	68	1.87	5.82E-05
UP_SEQ_FEATURE	mutagenesis site	667	1.20	6.73E-05
GOTERM_MF_FAT	GO:0008270~zinc ion binding	740	1.17	6.73E-05
GOTERM_CC_FAT	GO:0015935~small ribosomal subunit	38	2.26	6.75E-05
GOTERM_CC_FAT	GO:0044445~cytosolic part	72	1.77	6.89E-05
GOTERM_MF_FAT	GO:0008137~NADH dehydrogenase (ubiquinone) activity	30	2.55	9.48E-05
GOTERM_MF_FAT	GO:0050136~NADH dehydrogenase (quinone) activity	30	2.55	9.48E-05
GOTERM_MF_FAT	GO:0003954~NADH dehydrogenase activity	30	2.55	9.48E-05
GOTERM_MF_FAT	GO:0016879~ligase activity, forming carbon-nitrogen bonds	102	1.61	1.05E-04
GOTERM_CC_FAT	GO:0005730~nucleolus	248	1.33	1.05E-04
GOTERM_MF_FAT	GO:0046914~transition metal ion binding	875	1.15	1.20E-04
SP_PIR_KEYWORDS	transport	543	1.20	1.36E-04
GOTERM_MF_FAT	GO:0032559~adenyl ribonucleotide binding	498	1.22	1.42E-04
GOTERM_MF_FAT	GO:0030554~adenyl nucleotide binding	521	1.21	1.80E-04
GOTERM_MF_FAT	GO:0005524~ATP binding	491	1.21	1.94E-04

GOTERM_BP_FAT	GO:0043161~proteasomal ubiquitin-dependent protein catabolic process	55	1.96	2.40E-04
GOTERM_BP_FAT	GO:0010498~proteasomal protein catabolic process	55	1.96	2.40E-04
SP_PIR_KEYWORDS	electron transport	52	1.92	2.44E-04
GOTERM_MF_FAT	GO:0001883~purine nucleoside binding	527	1.20	2.54E-04
GOTERM_MF_FAT	GO:0001882~nucleoside binding	530	1.20	2.74E-04
GOTERM_BP_FAT	GO:0031396~regulation of protein ubiquitination	54	1.96	3.04E-04
GOTERM_MF_FAT	GO:0016881~acid-amino acid ligase activity	90	1.64	3.20E-04
GOTERM_BP_FAT	GO:0000398~nuclear mRNA splicing, via spliceosome	74	1.76	3.66E-04
GOTERM_BP_FAT	GO:0000377~RNA splicing, via transesterification reactions with bulged adenosine as nucleophile	74	1.76	3.66E-04
GOTERM_BP_FAT	GO:0000375~RNA splicing, via transesterification reactions	74	1.76	3.66E-04
GOTERM_MF_FAT	GO:0016651~oxidoreductase activity, acting on NADH or NADPH	46	2.02	4.02E-04
GOTERM_MF_FAT	GO:0016887~ATPase activity	135	1.48	4.31E-04
GOTERM_BP_FAT	GO:0016071~mRNA metabolic process	149	1.46	4.99E-04
GOTERM_MF_FAT	GO:0004842~ubiquitin-protein ligase activity	70	1.74	5.45E-04
GOTERM_BP_FAT	GO:0006120~mitochondrial electron transport, NADH to ubiquinone	29	2.51	6.98E-04
GOTERM_BP_FAT	GO:0006397~mRNA processing	132	1.49	7.08E-04
GOTERM_BP_FAT	GO:0045333~cellular respiration	52	1.95	7.51E-04
GOTERM_MF_FAT	GO:0046872~metal ion binding	1251	1.10	9.44E-04
GOTERM_BP_FAT	GO:0042773~ATP synthesis coupled electron transport	35	2.27	9.90E-04
GOTERM_BP_FAT	GO:0042775~mitochondrial ATP synthesis coupled electron transport	35	2.27	9.90E-04

GOTERM_MF_FAT	GO:0016655~oxidoreductase activity, acting on NADH or NADPH, quinone or similar compound as acceptor	31	2.31	1.35E-03
GOTERM_MF_FAT	GO:0043169~cation binding	1260	1.10	1.38E-03
GOTERM_CC_FAT	GO:0000151~ubiquitin ligase complex	46	1.91	1.60E-03
GOTERM_BP_FAT	GO:0006091~generation of precursor metabolites and energy	128	1.48	1.60E-03
GOTERM_BP_FAT	GO:0022904~respiratory electron transport chain	38	2.16	1.69E-03
GOTERM_BP_FAT	GO:0016568~chromatin modification	114	1.51	2.69E-03
GOTERM_MF_FAT	GO:0043167~ion binding	1274	1.10	2.83E-03
GOTERM_MF_FAT	GO:0042623~ATPase activity, coupled	111	1.49	3.28E-03
KEGG_PATHWAY	hsa03040:Spliceosome	57	1.66	3.58E-03
GOTERM_BP_FAT	GO:0022613~ribonucleoprotein complex biogenesis	81	1.63	3.72E-03
GOTERM_BP_FAT	GO:0015980~energy derivation by oxidation of organic compounds	68	1.71	3.95E-03
GOTERM_BP_FAT	GO:0006974~response to DNA damage stimulus	146	1.42	4.86E-03
SP_PIR_KEYWORDS	mrna splicing	87	1.54	5.30E-03
GOTERM_CC_FAT	GO:0000775~chromosome, centromeric region	57	1.72	5.65E-03
SP_PIR_KEYWORDS	cell division	105	1.47	6.31E-03
GOTERM_BP_FAT	GO:0031397~negative regulation of protein ubiquitination	41	2.01	6.35E-03
GOTERM_BP_FAT	GO:0008380~RNA splicing	116	1.48	6.39E-03
SMART	SM00355:ZnF_C2H2	264	1.25	7.31E-03
SP_PIR_KEYWORDS	oxidative phosphorylation	23	2.43	7.98E-03
SP_PIR_KEYWORDS	coiled coil	629	1.15	8.11E-03
GOTERM_BP_FAT	GO:0016567~protein ubiquitination	58	1.77	8.14E-03
GOTERM_CC_FAT	GO:0000123~histone acetyltransferase complex	29	2.17	9.03E-03
GOTERM_BP_FAT	GO:0022900~electron transport chain	56	1.78	9.28E-03

GOTERM_BP_FAT	GO:0031145~anaphase-promoting complex-dependent proteasomal ubiquitin-dependent protein catabolic process	37	2.07	9.94E-03
GOTERM_BP_FAT	GO:0051443~positive regulation of ubiquitin-protein ligase activity	39	2.02	1.01E-02
GOTERM_BP_FAT	GO:0046907~intracellular transport	235	1.30	1.03E-02
GOTERM_CC_FAT	GO:0005681~spliceosome	59	1.67	1.09E-02
GOTERM_BP_FAT	GO:0006281~DNA repair	115	1.47	1.17E-02
GOTERM_BP_FAT	GO:0051438~regulation of ubiquitin-protein ligase activity	42	1.95	1.22E-02
GOTERM_BP_FAT	GO:0051351~positive regulation of ligase activity	40	1.99	1.26E-02
GOTERM_BP_FAT	GO:0051340~regulation of ligase activity	43	1.93	1.47E-02
GOTERM_CC_FAT	GO:0044431~Golgi apparatus part	112	1.43	1.47E-02
GOTERM_BP_FAT	GO:0051439~regulation of ubiquitin-protein ligase activity during mitotic cell cycle	39	1.99	1.60E-02
GOTERM_BP_FAT	GO:0031398~positive regulation of protein ubiquitination	44	1.90	1.74E-02
GOTERM_CC_FAT	GO:0044451~nucleoplasm part	191	1.29	3.05E-02
GOTERM_CC_FAT	GO:0022627~cytosolic small ribosomal subunit	24	2.25	3.07E-02
GOTERM_BP_FAT	GO:0016569~covalent chromatin modification	59	1.70	3.17E-02
GOTERM_BP_FAT	GO:0051436~negative regulation of ubiquitin-protein ligase activity during mitotic cell cycle	36	2.01	3.20E-02
SP_PIR_KEYWORDS	dna repair	78	1.51	3.60E-02
GOTERM_CC_FAT	GO:0022625~cytosolic large ribosomal subunit	23	2.27	3.90E-02
UP_SEQ_FEATURE	zinc finger region:C2H2-type 9	143	1.40	3.95E-02
GOTERM_BP_FAT	GO:0051437~positive regulation of ubiquitin-protein ligase activity during mitotic cell cycle	37	1.98	3.95E-02

GOTERM_BP_FAT	GO:0000278~mitotic cell cycle	141	1.38	3.96E-02
GOTERM_CC_FAT	GO:0031903~microbody membrane	20	2.42	4.33E-02
GOTERM_CC_FAT	GO:0005778~peroxisomal membrane	20	2.42	4.33E-02
KEGG_PATHWAY	hsa04260:Cardiac muscle contraction	37	1.74	4.34E-02
UP_SEQ_FEATURE	zinc finger region:C2H2-type 10	129	1.42	4.95E-02
GOTERM_MF_FAT	GO:0015078~hydrogen ion transmembrane transporter activity	44	1.79	5.22E-02
SP_PIR_KEYWORDS	cell cycle	163	1.31	5.43E-02
GOTERM_MF_FAT	GO:0015077~monovalent inorganic cation transmembrane transporter activity	49	1.72	5.73E-02
UP_SEQ_FEATURE	zinc finger region:C2H2-type 8	155	1.37	6.72E-02
GOTERM_BP_FAT	GO:0006511~ubiquitin-dependent protein catabolic process	98	1.47	6.73E-02
SP_PIR_KEYWORDS	Spliceosome	53	1.63	6.76E-02
GOTERM_BP_FAT	GO:0006259~DNA metabolic process	183	1.31	7.17E-02
KEGG_PATHWAY	hsa03050:Proteasome	25	1.96	7.50E-02
GOTERM_BP_FAT	GO:0051352~negative regulation of ligase activity	36	1.95	7.58E-02
GOTERM_BP_FAT	GO:0051444~negative regulation of ubiquitin-protein ligase activity	36	1.95	7.58E-02
SP_PIR_KEYWORDS	mitosis	74	1.50	8.14E-02
SP_PIR_KEYWORDS	host-virus interaction	107	1.39	8.24E-02
SP_PIR_KEYWORDS	magnesium	156	1.31	8.52E-02
SMART	SM00651:Sm	13	2.88	8.72E-02
SP_PIR_KEYWORDS	DNA damage	81	1.46	9.28E-02
GOTERM_CC_FAT	GO:0015630~microtubule cytoskeleton	186	1.27	9.85E-02

Supplementary Table 4: Inrich Analysis of Overlap between Differentially Expressed Genes in CMC and 108 GWAS Regions		
Chr	Position	Gene
chr1	8411211..8638666	ENSG00000232912
chr1	44029353..44128047	KDM4A
chr1	97792597..98559093	DPYD-IT1,DPYD-AS2,ENSG00000259946
chr1	149998923..150242595	VPS45
chr1	177247854..177300809	BRINP2
chr1	207912179..208024062	MIR29B2CHG
chr1	243503764..244002773	SDCCAG8,AKT3,FABP7P1,AKT3-IT1,ENSG00000232184,ENSG00000236031
chr2	72357336..72368190	CYP26B1
chr2	149390792..149520186	EPC2
chr2	198148570..198835702	ANKRD44,SF3B1,COQ10B,MARS2
chr2	200161427..200309455	ENSG00000257045
chr2	200715272..200847990	FTCDNL1,TYW5,MAIP1
chr2	233559312..233753340	KCNJ13,SNORC
chr3	36843149..36945794	TRANK1
chr3	52541253..52903446	GLT8D1,SPCS1,GNL3,ITIH3

chr3	63792668..64004101	C3orf49,THOC7,ENSG00000224479,ATXN7,PSMD6,PSMD6-AS2,ENSG00000271843
chr3	135807609..136615413	MSL2,PCCB,STAG1
chr3	180588841..181205593	DNAJC19,SOX2-OT
chr4	170357792..170646088	CLCN3
chr4	176851045..176875795	GPM6A
chr5	60499131..60843553	ZSWIM6,ENSG00000251682
chr5	109030041..109208991	MAN2A1
chr5	137598340..137948140	REEP2,ETF1
chr5	140023818..140222641	TMCO6,NDUFA2,HARS,ZMAT2
chr6	28303243..28712247	ZBED9
chr7	24619556..24832129	GSDME
chr7	86403211..86459347	GRM3
chr7	104598021..105063238	LINC01004,ENSG00000272918,RWDD4P1
chr7	110843795..111205894	ENSG00000227948
chr8	4177791..4192528	CSMD1,PAICSP4
chr10	104423800..105165256	ARL3,BORCS7,NT5C2,CNNM2,INA,PCGF6,ATP5MD

chr11	46342942..46751184	ATG13,ARHGAP1
chr11	57386178..57682011	CLP1,MED19,SELENOH,BTBD18,CTNND1
chr11	124610011..124620147	VSIG2,ESAM
chr11	130714613..130749351	SNX19
chr11	133808038..133852957	IGSF9B
chr12	2321868..2523697	DCP1B,CACNA1C,CACNA1C-AS1,CACNA1C-AS4,CACNA1C-IT3
chr12	57428302..57682980	NEMP1,STAT6,NDUFA4L2
chr12	123447928..123908948	PITPNM2
chr14	103996205..104184737	APOPT1,ENSG00000246451
chr15	78803057..78926726	PSMA4
chr15	84661145..85153716	EFL1P1,UBE2Q2L,CSPG4P11,GOLGA2P7,GOLGA6L4,ENSG00000259683,DNM1P51,ENSG00000259774,GOLGA6L5P,UBE2Q2P1
chr15	91416565..91429042	FURIN

chr16	29924422..30144379	ASPHD1,ENSG00000260114,TMEM219,TAOK2,ENSG00000250616
chr16	67709262..68311260	GFOD2,RANBP10,THAP11,DDX28,SLC7A6
chr17	2095954..2220807	SMG6,SRR
chr17	17721979..18030240	DRC3,MYO15A
chr18	53453353..53804156	ENSG00000267284,LINC01415
chr19	19374061..19657632	MAU2
chr19	50067508..50135406	NOSIP
chr22	39975307..40016767	CACNA1I
chr22	41408693..41675126	EP300
chr22	42315706..42689370	LINC00634,SMDT1,NDUFA6,CYP2D6,CYP2D7,TCF20
chrX	5916759..6032730	NLGN4X

Supplementary Table 5: Inrich Analysis of Overlap between Differentially Expressed Genes in PsychENCODE and 108 GWAS Regions		
Chr	Position	Gene
chr1	97792597..98559093	DPYD,SEC63P1,DPYD-AS2,ENSG00000270911,MIR137HG
chr1	149998923..150242595	CA14
chr1	177247854..177300809	BRINP2
chr2	57943567..58502233	VRK2,FANCL
chr2	198148570..198835702	ANKRD44,HSPD1,RFTN2,PLCL1
chr2	225334104..225467840	ENSG00000228446
chr2	233559312..233753340	KCNJ13
chr3	2532788..2561691	CNTN4,CNTN4-AS2
chr3	17221252..17888256	TBC1D5
chr3	135807609..136615413	PPP2R3A
chr3	180588841..181205593	SOX2-OT
chr5	60499131..60843553	ZSWIM6
chr5	109030041..109208991	MAN2A1
chr5	137598340..137948140	REEP2,CTNNA1
chr5	140023818..140222641	NDUFA2,WDR55,ZMAT2
chr5	151941138..152797656	LINC01470
chr7	86403211..86459347	GRM3
chr7	104598021..105063238	KMT2E,SRPK2,ENSG00000272918
chr10	104423800..105165256	WBP1L,NT5C2
chr11	46342942..46751184	ARHGAP1
chr11	57386178..57682011	ENSG00000254602
chr11	124610011..124620147	VSIG2,ESAM
chr12	57428302..57682980	SHMT2,NDUFA4L2

chr12	123447928..123908948	OGFOD2,ABCB9,KMT5A,RILPL2
chr14	99707933..99719219	BCL11B
chr14	103996205..104184737	TRMT61A,ENSG00000246451
chr15	84661145..85153716	ADAMTSL3,ENSG00000259694,UBE2Q2P11
chr15	91416565..91429042	FES
chr16	29924422..30144379	ASPHD1,PPP4C
chr16	67709262..68311260	DUS2,NFATC3
chr19	30981639..31038995	ZNF536
chr22	41408693..41675126	L3MBTL2,ENSG00000235513
chr22	42315706..42689370	LINC00634,FAM109B
